# Supplementary material for: How prevalent is COVID-19 vaccine hesitancy in low-income and middle-income countries and what are the key drivers of hesitancy? Results from 53 countries
Source: BMJ Open. 2023 Nov 6;13(11):e069152. doi: 10.1136/bmjopen-2022-069152 (PMC10632876; doi:10.1136/bmjopen-2022-069152)
Supplement: Supplementary data [file bmjopen-2022-069152supp001.pdf]

Supplemental materials, COVID-19 Vaccine Hesitancy in 53 Countries

1. **Supplements**

Supplement 1: Detailed Description of Methods

2. **Supplemental Tables**

Supplemental Table S1: Description of 53 HFPS surveys

Supplemental Table S2a: Survey response options for COVID-19 vaccine hesitancy: distribution of countries by questionnaire type

Supplemental Table S2b: Survey response options for COVID-19 vaccine hesitancy: Harmonized answers by questionnaire type

Supplemental Table S3: Percent of population hesitant to take the COVID-19 vaccine, HFPS

Supplemental Table S4: Tukey's tests of significance of differences across groups in percent of population hesitant to take the COVID-19 vaccine

Supplemental Table S5a: Correlates of vaccine hesitancy (Logit model, marginal effects)

Supplemental Table S5b: Correlates of vaccine hesitancy (Ordinal logit regression, marginal effects)

Supplemental Table S6: Percent of population hesitant to take the COVID-19 vaccine, 14 countries with multiple rounds of survey results (95% confidence intervals in parentheses).

Supplemental Table S7: Comparison of results on vaccine hesitancy from the HFPS and other studies

3. **Supplemental Figures**

Supplemental Figure S1: Vaccination rates by income group

Supplemental Figure S2: Vaccine rollout rates by income group

Supplemental Figure S3: Comparison of share of population COVID-19 that is hesitant to take the COVID-19 vaccine, HFPS with other studies

Supplemental Figure S4: Comparison of COVID-19 Vaccine Hesitancy with pre-COVID sentiment about vaccines from 2019 Global Monitor, 45 countries

Supplemental Figure S5: Reason for "definitely not" (upper panel) or "probably not" (lower panel) taking the COVID-19 vaccine, Facebook data for month of HFPS)

4. **References**

Dayton Eberwein J, *et al. BMJ Open* 2023; 13:e069152. doi: 10.1136/bmjopen-2022-069152

Supplement 1: Detailed Description of Methods

Data

This analysis describes the levels of vaccine hesitancy and its reasons in 53 developing countries between October 2020 August 2021 using data from the World Bank's COVID-19 high-frequency phone surveys (HFPS) which were implemented to monitor the impact of COVID-19 on households around the world (World Bank 2020). Data are available for one survey round from 39 countries and two or more survey rounds for 14 countries. The countries in the pooled database represent five out of six regions defined by the World Bank: Latin America and the Caribbean (24 countries), Sub-Saharan Africa (14), East Asia and Pacific (7), Europe and Central Asia (6) and the Middle East and North Africa (2).[1] By income group, the sample includes 12 low-income countries, 13 lower-middle income countries, 23 upper middle-income countries and five high-income countries (Supplemental Table 1 provides a list of countries with survey month, sample size, region and country income group).

A global core questionnaire served as the basis for each country's survey but was then customized to fit the local context. As a result, the exact questions may vary across countries, but topics typically included knowledge and concerns about COVID-19, access to food, health care and education, employment and income loss, and safety nets and coping strategies. The survey data was then harmonized ex-post to enable cross-country comparability. The questionnaire was flexible and adapted over time to the pandemic context. The vaccine module, for example, was included more recently.

The sampling frame was drawn from pre-existing nationally representative household surveys in 19 countries, random digit dialing (RDD) in 29 countries, and a list of phone numbers typically obtained from mobile phone operators in 5 countries. The RDD design samples from all active mobile and landline phone numbers, such that RDD surveys are representative of the population aged 18 and over with an active phone number, conditional on response and survey completion. Sampling weights were then constructed to correct for selection bias due to the inability to contact households that did not participate in the survey either due to non-response or lack of access to a phone, with the goal of obtaining estimates that are as close to being nationally representative as possible. Information was collected from one respondent per household. In the case of countries where the sampling frame was derived from a previous survey, this was typically the household head. In other cases, such as when the sample utilized RDD, the respondent was more representative of individuals within the household. While the non-random selection of individuals in the former cases is not ideal, in an earlier analysis of labor market indicators derived from phone surveys, Kugler et al. (2021) find little evidence of differences in employment outcomes or trends over time being affected by the oversampling of household heads with respect to variables such as level of education, gender or location, the main source of bias being for age comparisons. Within the pooled data set, the household sampling weights are scaled such that countries are weighted equally, such that estimates of vaccine hesitancy reported in this study are averages of country averages.[2]

Outcome measures

The questions asked about vaccine hesitancy in the surveys varied depending on whether a vaccine was available in the country at the time of the survey. In surveys that took place before the vaccine rollout had begun in that country, the question asked was "*If an approved vaccine to prevent COVID-19 was to become*

[1] South Asia is not represented due to lack of available data. The Middle East and North Africa includes two countries—Iraq and Lebanon.
[2] Using population weights would have resulted in the results being driven by a small number of large countries.

available at no cost, would you agree to be vaccinated?" In surveys that took place after the vaccine rollout had begun, the wording of the question was either "*Are you planning to be vaccinated?*" or "*When a vaccine to prevent COVID-19 is available to you, are you planning to be vaccinated?"* In the sample, 47 countries offered three answer options (yes, not sure, and no), while the other six offered only two categories (yes and no). For this analysis, we combine the "no" and "not sure" answers to obtain the measure of vaccine hesitancy.

To obtain the respondent's reason for vaccine hesitancy, survey respondents who answered "no" or "not sure" were asked "*What is your (main) reason/concern for not wanting to be vaccinated / not being sure if you want to be vaccinated*?" The answer categories varied widely across surveys. To make these more comparable across countries, answers were remapped into the following nine most common categories (see Supplemental Tables 2a and 2b for the original answer options in each survey and a mapping of how they were harmonized):

1. Safety, which includes concerns about side effects
2. Efficacy
3. Distrust of government, pharmaceutical industry, international community
4. Dislike of vaccines in general
5. Preference for natural immunity, which included perceiving self as low risk and already having had COVID-19 infection
6. Lack of knowledge or access, which included "I do not have enough information about the vaccine," "too hard to get," "health facility to far" and "I don't have the time"
7. Religious reasons
8. Not eligible, which included "counter-indication," "recent medical discharge, "have an underlying health condition and believe taking the vaccine will make it worse"
9. Other

The surveys also differed across countries in terms of whether a single concern or multiple concerns were collected. To account for the difference in the number of response options, the results are presented separately.

Contextual data

Country-level contextual data were drawn from other sources. New COVID-19 cases per million, measured as a 7-day rolling average prior to the midpoint of the month household survey data was collected in each country, were obtained from Dong and colleagues (2022), and the Oxford Stringency Index was drawn from Hale et al. 2022. The latter is an index, ranging from 0–100, that indicates the degree to which various restrictions were put in place by governments to control the pandemic (for example, school closures and shelter-in-place requirements). Confidence in the press, the government, and the WHO was drawn from the World Values Survey Wave 7, fielded in 2017-2020, and aggregated up to the country level (Inglehart 2022). Excess deaths due to COVID-19 draw on WHO data (WHO 2022). Country geographic region and income group are based on the World Bank classifications.

Analytical strategy

Data from 53 countries was pooled into a single data set. First, we report point estimates and 95% confidence intervals for each country based on standard errors clustered at the state or province level within each country. We also report the simple average across all countries and stratified by: (i) World Bank region, (ii) World Bank country income group, (iii) urban vs. rural residence, (iv) gender of the respondent, (v) whether the respondent is head of household, (vi) age of the respondent (under 35 years,

35-64 years, and 65 years and older, and (vii) educational attainment of the respondent (some primary school, some secondary school, some tertiary). Tukey's test of multiple comparisons is used to test the significance of differences in rates of vaccine hesitancy across the above-mentioned groups. Second, we use multivariate regression analysis to assess the relative association of vaccine hesitancy and the correlates and contextual variables. Trends over time in levels of vaccine hesitancy are examined in 14 countries with more than one wave of results available. Finally, the analysis describes the reasons for vaccine hesitancy.

Dayton Eberwein J, *et al. BMJ Open* 2023; 13:e069152. doi: 10.1136/bmjopen-2022-069152

Supplemental Table S1: Description of 53 HFPS surveys

Country	Survey Months	Total Number of households	Sample frame
**Antigua and Barbuda**	06/2021	790	RDD
**Argentina**	06/2021	1216	RDD
**Belize**	06/2021	816	RDD
**Bolivia**	05/2021	1272	RDD
**Brazil**	08/2021	2166	RDD
**Bulgaria**	07/2021	1000	RDD
**Burkina Faso**	12/2020	1944	Previous survey
**Chile**	06/2021	1212	RDD
**Colombia**	06/2021	1221	RDD
**Congo, Dem. Rep.**	12/2020	986	Previous Survey
**Congo, Rep.**	12/2020, 03/2021	578, 1495	Previous Survey
**Costa Rica**	06/2021	802	RDD
**Croatia**	03/2021	1217	Non-survey list
**Dominica**	06/2021	861	RDD
**Dominican Republic**	06/2021	1205	RDD
**Ecuador**	05/2021	1352	RDD
**El Salvador**	06/2021	816	RDD
**Ethiopia**	10/2020, 02/2021	2704, 2178	Previous Survey
**The Gambia**	12/2020, 04/2021, 08/2021	1334, 1287, 1059	Previous Survey
**Georgia**	01/2021, 03/2021, 06/2021	2033, 2100, 1936	RDD
**Guinea**	11/2020	1334	Previous Survey
**Guatemala**	06/2021	1206	RDD
**Guyana**	06/2021	785	RDD
**Haiti**	07/2021	2813	Non-Survey list
**Honduras**	07/2021	1021	RDD
**Indonesia**	11/2020, 03/2021	3953, 3555	Previous Survey
**Iraq**	12/2020, 01/2021,06/2021, 07/2021, 08/2021	1614,1651,1378, 1297,1141	Non-Survey list
**Jamaica**	06/2021	828	RDD
**Kazakhstan**	02/2021, 05/2021, 06/2021	917, 1732, 1610	Previous Survey
**Kenya**	03/2021	6730	Previous Survey

Country	Survey Months	Total Number of households	Sample frame
**Lao PDR**	03/2021	2153	RDD
**Lebanon**	03/2021	5113	RDD
**Mali**	01/2021	1884	Previous Survey
**Malawi**	11/2020, 03/2021, 04/2021	1589, 1549,1338	Previous Survey
**Malaysia**	06/2021	2210	RDD
**Mexico**	06/2021	2624	RDD
**Mongolia**	12/2020	1147	Previous Survey
**Nicaragua**	06/2021	833	RDD
**Nigeria**	02/2021, 10/2020	1699, 1762	Previous Survey
**Panama**	06/2021	815	RDD
**Paraguay**	06/2021	1076	RDD
**Peru**	06/2021	1210	RDD
**Philippines**	12/2020, 05/2021	1805, 2122	Non-survey list
**Saint Lucia**	06/2021	835	RDD
**Sierra Leone**	11/2020	1198	Previous Survey
**Sudan**	03/2021	2545	Non-Survey list
**Tajikistan**	05-08/2021	232	Previous Survey
**Thailand**	05/2021	1786	RDD
**Uganda**	11/2020, 02/2021	2135, 2121	Previous Survey
**Uruguay**	06/2021	816	RDD
**Uzbekistan**	04-06/2021	1496, 1356, 1300	Previous Survey
**Vietnam**	01/2021	3940	Previous Survey
**Zimbabwe**	12/2020	1227	Previous Survey

Dayton Eberwein J, *et al. BMJ Open* 2023; 13:e069152. doi: 10.1136/bmjopen-2022-069152

Supplemental Table S2a: Survey response options for COVID-19 vaccine hesitancy: distribution of countries by questionnaire type

Questionnaire type 1	Questionnaire type 2	Questionnaire type 3	Questionnaire type 4	Questionnaire type 5	Questionnaire type 6	Questionnaire type 7	Questionnaire type 8	Questionnaire type 9	Questionnaire type 10	Questionnaire LAC	Questionnaire type 11	Questionnaire type 12
Kenya	*Philippines*	*Lebanon*	*Congo, Democratic Republic*	*Guinea*	*Malaysia*	*Mali*	*Gambia, The*	*Lao PDR*	*Cambodia*	*Argentina*	*Kazakhstan*	*Croatia*
Thailand	*Indonesia*		*Republic of Congo*	*Sudan*		*Burkina Faso*		*Mongolia*		*Antigua and Barbuda*	*Kyrgyzstan*	
	Djibouti			*Sierra Leone*		*Uganda*				*Belize*	*Uzbekistan*	
						Ethiopia				*Bolivia*		
						Malawi				*Brazil*		
						Nigeria				*Chile*		
										Colombia		
										Costa Rica		
										Dominica		
										Dominican Republic		
										Ecuador		
										Guatemala		
										Guyana		
										Honduras		
										Haiti		
										Jamaica		
										Mexico		
										Nicaragua		
										Panama		
										Peru		
										Paraguay		
										El Salvador		
										Uruguay		

Dayton Eberwein J, *et al. BMJ Open* 2023; 13:e069152. doi: 10.1136/bmjopen-2022-069152

Supplemental Table S2b: Survey response options for COVID-19 vaccine hesitancy: Harmonized answers by questionnaire type

Answer categories	Quest type 1	Quest type 2	Quest type 3	Quest type 4	Quest type 5	Quest type 6	Quest type 7	Quest type 8	Quest type 9	Quest type 10	Quest LAC	Quest type 11	Quest type 12
**1. Efficacy**	I don't think it will work	I don't think COVID-19 vaccines effective/work	I don't think coronavirus vaccines are effective/work		I don't think vaccines work	I don't think it will work	I don't think it will work	I do not think the vaccine would work against COVID	I don't think COVID-19 vaccines effective/work		I do not think they are effective, that they work		It may not work
						I don't think the vaccines available in my country are effective							
**2 Safety (includes side effects)**	I don't think it will be safe	I am worried about the safety of the vaccine		Safety of the vaccine	I am worried about the vaccine safety		I don't think it will be safe	I fear the unforeseen future negative side effects from the COVID-19 vaccine	I am worried about the safety of the vaccine	I don't think it is safe		The risk of vaccinating is higher than the benefits	Health risks
	I am worried about the side effects	I am worried about the side effects	I am worried about the side effects of the vaccine			I am worried about the side effects	I'm worried about side effects	I have heard the vaccine has negative side effects	I am worried about the side effects		I don't think it's safe, because of the side effects		
**3. Perceive self as low risk**	I am not enough at risk of contracting COVID-19	I am strong, I never got any disease in the past	I am not enough at risk of contracting coronavirus			I am not enough at risk of contracting COVID-19	I am not at enough risk of contracting covid-19	I believe I do not need to be vaccinated			I am not at risk of getting covid-19		I am not worried about COVID-19
**4.Dislike vaccines in general**	I am against vaccines in general	I am against vaccines in general	I am against vaccines in general	General distrust of vaccines	I don't trust the vaccines in general	In general, I don't trust vaccines	I am against the vaccine in general		I am against vaccines in general		I am against vaccines in general	Negative past experiences with vaccines	I am against vaccines in general
**5 Religious**	It is against my religion	Religious reasons	Religious reasons		I have religious reasons/concerns	It is against my religion.	It's against my religion		Religious reasons		Religious reasons	Personal or religious beliefs	
		I am concerned about its											

Answer categories	Quest type 1	Quest type 2	Quest type 3	Quest type 4	Quest type 5	Quest type 6	Quest type 7	Quest type 8	Quest type 9	Quest type 10	Quest LAC	Quest type 11	Quest type 12
		halal certification											
**6 Concerned about getting covid at facility**	I'm worried to get infected with COVID-19 at the health facility	I am worried of getting infected with COVID-19 at the health facility	I am worried of getting infected with coronavirus at the health facility			I am worried to get infected with COVID-19 at the health facility.			I am worried to get infected with COVID-19 at the health facility		I am concerned of being infected with covid-19 at the health center		
**7 Supply /access barrier**	Health facility is too far or too hard to get to		Health facility too far or too hard to get to		It does not suit me (will be given too far or difficult to find)	Health facility too far or too hard to get to		There is shortage of vaccines in the country		I don't know how to access the vaccine	Health center too far or hard to reach		
	I don't have time to get vaccinated/ It will take too long to get vaccinated					It will take too long to get vaccinated/ I don't have time to get vaccinated		There is no vaccination center near my place		I am not the priority group to get the vaccine	I don't have time to go to get vaccinated		
**8 Prefer natural immunity (includes already had COVID-19)**			I already had coronavirus		I believe that the remedies natural or traditional	I already had COVID-19		I prefer to build immunity against COVID-19 naturally by having the disease			I already had covid-19 / I no longer need it		I already had COVID-19 and do''t need a vaccine
					It is better to leave nature take its course; the COVID symptoms are mostly light								

Answer categories	Quest type 1	Quest type 2	Quest type 3	Quest type 4	Quest type 5	Quest type 6	Quest type 7	Quest type 8	Quest type 9	Quest type 10	Quest LAC	Quest type 11	Quest type 12
**9 Distrust (of10overnmentt/pharmaceutical industry/international community)**				Be wary of international community	I do''t trust the government/ to the organization who gives the vaccine			I do not trust pharmaceutical companies				Lack of trust to producers of vaccines	I do''t trust the pharmaceutical industry
								I do not trust the government				Lack of trust to heath system or healthcare providers	I do''t trust the government
								I do not trust the COVID-19 vaccines					
								I heard the vaccine is meant to control population growth					
								I do not believe in COVID-19					
**10 Not eligible / conterindication**								I have underlying health conditions and I believe taking the vaccine will make it worse		I am not eligible to get the vaccine			Counter-indication or prolonged medical discharge
**11 Lack of knowledge about COVID 19 Vaccine**								I do not have enough information about the vaccine					

Answer categories	Quest type 1	Quest type 2	Quest type 3	Quest type 4	Quest type 5	Quest type 6	Quest type 7	Quest type 8	Quest type 9	Quest type 10	Quest LAC	Quest type 11	Quest type 12
								I did not know that a vaccine exists against COVID-19					
**12 Other**	Other (specify)	Other (specify)	Some other reason	other	Others	Other (Specify)	other, specify)	Everyday stresses are overwhelming to think about getting vaccinated	Others (specify)	Other, specify	Other (specify)	Other	
		I will wait till more people are vaccinated											
		No one in my neighborhood got it				I am not sure I will get the vaccine I want							

Supplemental Table S3: Percent of population hesitant to take the COVID-19 vaccine, HFPS

Average across countries	Percent (%) hesitant to take the COVID-19 vaccine	Confidence Intervals
All countries	20.0	[17.24 , 22.67]
East Asia & Pacific	26.2	[21.38 , 31.02]
Europe & Central Asia	58.8	[55.04 , 62.56]
Latin America & Caribbean	8.0	[6.45 , 9.47]
Middle East & North Africa	47.4	[38.80 , 55.96]
Sub-Saharan Africa	15.5	[11.81 , 19.19]
Low income countries	14.6	[7.82 , 21.39]
Lower middle income countries	27.7	[23.78 , 31.65]
Upper middle income countries	12.7	[9.77 , 15.61]
High income countries	5.9	[3.40 , 8.36]
Urban	17.6	[14.60 , 20.75]
Rural	23.2	[19.83 , 26.64]
Male	17.3	[15.02 , 19.63]
Female	22.5	[18.77 , 26.21]
Head of household	10.8	[12.82 , 18.53]
Non-Head of household	9.4	[17.37 , 26.54]
No education	22.8	[15.36 , 30.31]
Any Primary	19.8	[15.80 , 23.78]
Any Secondary	19.0	[14.64 , 23.42]
Any Tertiary	13.7	[10.56 , 16.83]
Age 34 and younger	20.3	[17.29 , 23.34]
Age 35— 64	20.1	[17.34 , 22.84]
Ages 65 and older	17.8	[13.70 , 21.80]
Antigua and Barbuda	24.5	[22.51 , 26.49]
Argentina	10.1	[8.66 , 11.53]
Belize	28.4	[26.10 , 30.64]
Bolivia	24.3	[22.47 , 26.04]
Brazil	3.1	[2.15 , 4.04]
Bulgaria	66.2	[59.48 , 72.90]
Burkina Faso	23.6	[16.60 , 30.53]
Chile	3.4	[2.22 , 4.57]
Colombia	11.2	[8.91 , 13.51]
Congo, Dem. Rep.	61.2	[61.23 , 61.23]
Congo, Rep.	13.0	[11.20 , 14.79]

Dayton Eberwein J, *et al. BMJ Open* 2023; 13:e069152. doi: 10.1136/bmjopen-2022-069152

Average across countries	Percent (%) hesitant to take the COVID-19 vaccine	Confidence Intervals
Costa Rica	11.9	[9.66 , 14.22]
Croatia	33.2	[29.21 , 37.25]
Dominica	35.9	[32.63 , 39.14]
Dominican Republic	5.0	[2.25 , 7.81]
Ecuador	19.4	[15.54 , 23.21]
El Salvador	8.2	[6.91 , 9.44]
Ethiopia (excludes Eritrea)	3.5	[1.81 , 5.09]
Fm Sudan	23.7	[19.19 , 28.11]
Gambia, The	33.1	[27.44 , 38.70]
Georgia	65.2	[59.62 , 70.72]
Guatemala	29.6	[25.09 , 34.05]
Guinea	20.2	[15.74 , 24.61]
Guyana	20.0	[15.75 , 24.23]
Haiti	58.1	[55.31 , 60.86]
Honduras	13.9	[10.31 , 17.51]
Indonesia	21.4	[17.71 , 25.07]
Iraq	47.4	[38.81 , 55.99]
Jamaica	50.6	[47.11 , 54.03]
Kazakhstan	75.3	[72.39 , 78.18]
Kenya	17.9	[17.11 , 18.74]
Lao PDR	13.0	[9.70 , 16.25]
Lebanon	32.2	[24.56 , 39.84]
Malawi	29.3	[25.03 , 33.54]
Malaysia	25.7	[22.37 , 29.10]
Mali	21.1	[13.14 , 29.03]
Mexico	6.2	[4.85 , 7.55]
Mongolia	19.3	[14.13 , 24.38]
Nicaragua	18.7	[16.04 , 21.40]
Nigeria	16.6	[12.27 , 20.84]
Panama	13.3	[11.12 , 15.49]
Paraguay	15.3	[10.71 , 19.79]
Peru	10.6	[7.55 , 13.59]
Philippines	53.6	[46.28 , 60.83]
Sierra Leone	21.5	[17.31 , 25.66]
St. Lucia	43.2	[38.46 , 47.93]
Tajikistan	26.7	[18.32 , 35.03]
Thailand	36.6	[31.25 , 42.00]
Uganda	11.6	[6.51 , 16.61]
Uruguay	9.1	[5.94 , 12.17]
Uzbekistan	54.6	[50.02 , 59.18]
Vietnam	15.9	[11.72 , 20.14]
Zimbabwe	15.8	[10.42 , 21.14]

Dayton Eberwein J, *et al. BMJ Open* 2023; 13:e069152. doi: 10.1136/bmjopen-2022-069152

Supplemental Table S4: Tukey's tests of significance of differences across groups in percent of population hesitant to take the COVID-19 vaccine

Group	Comparison	Difference	Std. Error	t-score	p-value	Signi-ficance
Gender	Female vs Male	0.052	0.022	2.316	0.021	**
Household Status	Head vs Non-Head	-0.063	0.026	-2.398	0.016	**
Age	Ages 65 and older vs Age 35 - 64	-0.023	0.025	-0.937	0.349	
	Ages 65 and older vs Age 34 and younger	-0.026	0.026	-0.994	0.320	
	Age 35 - 64 vs Age 34 and younger	-0.002	0.021	-0.107	0.543	
Education	Any Primary vs Any Secondary	0.008	0.030	0.250	0.599	
	Any Primary vs Any Tertiary	0.061	0.026	2.355	0.019	**
	Any Primary vs No Education	-0.030	0.043	-0.705	0.481	
	Any Secondary vs Any Tertiary	0.053	0.028	1.941	0.052	*
	Any Secondary vs No Education	-0.038	0.044	-0.860	0.390	
	Any Tertiary vs No Education	-0.091	0.041	-2.211	0.027	**
Sector	Rural vs Urban	0.056	0.023	2.375	0.018	**
Region	EAP vs ECA	-0.326	0.031	-10.450	0.000	***
	EAP vs LAC	0.182	0.026	7.077	0.000	***
	EAP vs MENA	-0.212	0.050	-4.219	0.000	***
	EAP vs SSA	0.107	0.031	3.454	0.001	***
	ECA vs LAC	0.508	0.021	24.579	0.000	***
	ECA vs MENA	0.114	0.048	2.390	0.017	**
	ECA vs SSA	0.433	0.027	16.103	0.000	***
	LAC vs MENA	-0.394	0.044	-8.870	0.000	***
	LAC vs SSA	-0.075	0.020	-3.705	0.000	***
	MENA vs SSA	0.319	0.048	6.691	0.000	***

Dayton Eberwein J, *et al. BMJ Open* 2023; 13:e069152. doi: 10.1136/bmjopen-2022-069152

Income Group	HIC vs LIC	-0.087	0.037	-2.368	0.018	**
	HIC vs LMIC	-0.218	0.024	-9.202	0.000	***
	HIC vs UMIC	-0.068	0.020	-3.481	0.001	***
	LIC vs LMIC	-0.131	0.037	-3.559	0.000	***
	LIC vs UMIC	0.019	0.024	0.808	0.419	
	LMIC vs UMIC	0.150	0.025	6.010	0.000	***

Significance levels: *** for p-values <= 0.01, ** for 0.01 < p-values <= 0.05, * for 0.05 < p-values <= 0.1

Supplemental Table S5a: Correlates of vaccine hesitancy (Logit model, marginal effects)

Dep. Var: Hesitancy	(1)	(2)	(3)	(4)	(5)	(6)
Male	-0.027*	-0.028*	-0.027*	-0.027*	-0.033**	-0.040***
	(0.013)	(0.013)	(0.013)	(0.012)	(0.012)	(0.011)
Head of HH	0.013	0.009	0.021	-0.001	0.014	0.007
	(0.014)	(0.014)	(0.013)	(0.016)	(0.015)	(0.012)
Education of respondent (ref – no education)						
No education	-0.062	-0.062	-0.055	-0.068*	-0.074*	-0.075*
	(0.032)	(0.032)	(0.031)	(0.034)	(0.036)	(0.035)
Any primary	-0.061	-0.060	-0.054	-0.072	-0.069	-0.086*
	(0.037)	(0.038)	(0.033)	(0.040)	(0.042)	(0.038)
Any secondary	-0.130***	-0.131***	-0.122***	-0.142***	-0.133**	-0.153***
	(0.036)	(0.037)	(0.033)	(0.040)	(0.044)	(0.040)
Age group (ref. -- 34 and younger)						
Working age (35-64)	-0.047***	-0.048***	-0.042**	-0.050***	-0.046**	-0.049***
	(0.014)	(0.013)	(0.015)	(0.013)	(0.015)	(0.012)
Retirement age (65+)	-0.103***	-0.102***	-0.096***	-0.101***	-0.103***	-0.102***
	(0.024)	(0.025)	(0.024)	(0.023)	(0.025)	(0.024)
Rural area	0.016	0.014	0.015	0.008	0.012	0.011
	(0.019)	(0.018)	(0.017)	(0.015)	(0.015)	(0.011)
Region (ref – LAC)						
EAP	0.189**	0.192*	0.222**	0.095	0.264*	0.188
	(0.067)	(0.078)	(0.077)	(0.069)	(0.130)	(0.130)
ECA	0.313***	0.315***	0.295***	0.328***	0.313***	0.295***
	(0.055)	(0.057)	(0.056)	(0.059)	(0.040)	(0.062)
MNA	0.208***	0.212***	0.245***	0.216***	0.282***	0.369***
	(0.037)	(0.040)	(0.062)	(0.032)	(0.059)	(0.051)
SSA	0.074	0.081	0.070	-0.022	0.047	0.022
	(0.075)	(0.077)	(0.070)	(0.105)	(0.083)	(0.104)
Income group (ref. – LIC)						
LMIC	-0.068	-0.065	-0.045	-0.088	-0.054	-0.071
	(0.122)	(0.119)	(0.112)	(0.123)	(0.097)	(0.069)
UMIC	-0.047	-0.038	-0.066	-0.095	-0.035	-0.137
	(0.149)	(0.148)	(0.130)	(0.150)	(0.116)	(0.087)
HIC	-0.173	-0.164	-0.190	-0.223	-0.202	-0.309***
	(0.163)	(0.160)	(0.143)	(0.164)	(0.130)	(0.090)
Survey month (ref -- Nov 2020 - Jan						
March - May 2021	-0.069	-0.060	-0.094	-0.056	-0.095	-0.083
	(0.095)	(0.108)	(0.103)	(0.098)	(0.104)	(0.095)
June - August 2021	-0.029	-0.025	-0.024	-0.052	-0.057	-0.055
	(0.088)	(0.098)	(0.100)	(0.087)	(0.092)	(0.075)
New COVID-19 cases per million, terciles (ref. – bottom tercile)						
Cases (middle tercile)		-0.017				0.026
		(0.067)				(0.060)
Cases (top tercile)		-0.019				-0.016
		(0.057)				(0.047)
Oxford stringency index terciles (ref. – bottom tercile)						
Stringency (middle tercile)			0.065			0.134
			(0.072)			(0.070)
Stringency (top tercile)			-0.008			0.027
			(0.076)			(0.068)
WHO excess deaths due to COVID-19 terciles (ref.- bottom tercile)						

Dep. Var: Hesitancy	(1)	(2)	(3)	(4)	(5)	(6)
Excess deaths (middle tercile)				-0.117*		-0.109
				(0.052)		(0.072)
Excess deaths (top tercile)				-0.069		0.040
				(0.072)		(0.108)
Confidence in government index tercile (ref.- top tercile)						
Confidence in government					0.048	0.070
					(0.053)	(0.057)
Confidence in government					0.024	0.053
					(0.097)	(0.103)
Pseudo R-squared	0.0745	0.0748	0.0794	0.0813	0.0869	0.1067
N	65088	65088	65088	65088	65088	65088

Notes: Weighted logit regressions. Marginal effects reported. Standard errors clustered at country level. Ref=reference group; EAP= East Asia and the Pacific; ECA= Europe and Central Asia; LAC=Latin America and the Caribbean; MNA= Middle East and North Africa; SSA= Sub-Saharan Africa; LIC=Low-income countries; LMIC=Lower middle-income countries, UMIC=Upper middle-income countries. *, **, *** indicates significance at the 95%, 99%, and 99.9% level.

Supplemental Table S5b: Correlates of vaccine hesitancy (Ordinal logit regression, marginal effects)

Dep. Var.:	Would you get the vaccine?		
	No	Not sure	Yes
Male	-0.025**	-0.007**	0.036**
	(0.01)	(0.002)	(0.011)
Head of HH	0.011	0.001	-0.006
	(0.012)	(0.002)	(0.011)
Education of respondent (ref – Tertiary)			
No education	0.119***	0.022**	-0.156***
	-0.028	(0.007)	(0.038)
Any primary	0.057**	0.013**	-0.080***
	-0.018	(0.004)	(0.022)
Any secondary	0.058***	0.012***	-0.068***
	(0.009)	(0.003)	(0.010)
Age group (ref. -- 34 and younger)			
Working age (35-64)	-0.040***	-0.009***	0.049***
	(0.010)	(0.002)	(0.012)
Retirement age (65+)	-0.079***	-0.018***	0.097***
	(0.020)	(0.005)	(0.025)
Rural area	0.010	0.002	-0.012
	(0.009)	(0.002)	(0.011)
Region (ref – LAC)			
EAP	0.149	0.034	-0.183
	(0.093)	(0.021)	(0.113)
ECA	0.238***	0.055***	-0.293***
	(0.044)	(0.016)	(0.058)
MNA	0.288***	0.066***	-0.354***
	(0.042)	(0.011)	(0.049)
SSA	0.015	0.004	-0.019
	(0.082)	(0.019)	(0.101)
Income group (ref. – LIC)			
LMIC	-0.074	-0.017	0.091
	(0.055)	(0.012)	(0.067)
UMIC	-0.109	-0.025	0.134
	(0.069)	(0.017)	(0.085)
HIC	-0.241***	-0.056**	0.297***
	(0.071)	(0.019)	(0.088)
Survey month (ref -- Nov 2020 - Jan 2021)			
March - May 2021	-0.054	-0.011	0.065
	(0.081)	(0.016)	(0.098)
June - August 2021	-0.046	-0.010	0.055
	(0.065)	(0.012)	(0.077)
New COVID-19 cases per million, terciles (ref. – top tercile)			
Cases (middle tercile)	0.016	0.003	-0.019
	(0.048)	(0.010)	(0.058)
Cases (bottom tercile)	-0.028	-0.006	0.034
	(0.037)	(0.008)	(0.045)
Oxford stringency index terciles (ref. – top tercile)			
Stringency (middle tercile)	0.089	0.020	-0.110
	(0.057)	(0.013)	(0.070)
Stringency (bottom tercile)	0.012	0.003	-0.015
	(0.041)	(0.011)	(0.052)

Dayton Eberwein J, *et al. BMJ Open* 2023; 13:e069152. doi: 10.1136/bmjopen-2022-069152

Dep. Var.:	Would you get the vaccine?		
	No	Not sure	Yes
WHO excess deaths due to COVID-19 terciles (ref.- bottom tercile)			
Excess deaths (middle tercile)	-0.075	-0.019	0.094
	(0.057)	(0.015)	(0.071)
Excess deaths (top tercile)	0.057	0.010	-0.067
	(0.096)	(0.015)	(0.112)
Confidence in government index tercile (ref.- top tercile)			
Confidence in government (middle tercile)	0.052	0.016	-0.069
	(0.041)	(0.013)	(0.054)
Confidence in government (bottom tercile)	0.040	0.013	-0.053
	(0.070)	(0.023)	(0.093)
R-squared		0.0866	
N		65088	

Notes: Weighted ordinal logit regressions. Categories ranked from no to not sure to yes. Marginal effects reported. Standard errors clustered at country level. Ref=reference group; EAP= East Asia and the Pacific; ECA= Europe and Central Asia; LAC=Latin America and the Caribbean; MNA= Middle East and North Africa; SSA= Sub-Saharan Africa; LIC=Low-income countries; LMIC=Lower middle-income countries, UMIC=Upper middle-income countries. *, **, *** indicates significance at the 95%, 99%, and 99.9% level.

Supplemental Table S6: Percent of population non-hesitant to take the COVID-19 vaccine, 14 countries with multiple rounds of survey results (95% confidence intervals in parentheses).

country	**Oct-2020**	**Nov-2020**	**Dec-2020**	**Jan-2021**	**Feb-2021**	**Mar-2021**	**Apr-2021**	**May-2021**	**Jun-2021**	**Jul-2021**	**Aug-2021**
Burkina Faso			0.79 (0.73, 0.86)						0.76 (0.67, 0.86)		
Congo, Rep.			0.84 (0.75, 0.94)			0.87 (0.81, 0.93)					
Ethiopia	0.98 (0.91, 1.00)				0.97 (0.89, 1.00)						
Gambia, The			0.65 (0.58, 0.71)				0.55 (0.49, 0.60)				0.67 (0.60, 0.74)
Georgia				0.36 (0.33, 0.38)		0.29 (0.27, 0.32)			0.35 (0.32, 0.38)		
Indonesia		0.79 (0.73, 0.85)				0.79 (0.72, 0.85)					
Iraq			0.66 (0.61, 0.72)	0.50 (0.45, 0.54)					0.45 (0.40, 0.50)	0.47 (0.42, 0.52)	0.53 (0.47, 0.58)
Kazakhstan					0.23 (0.17, 0.28)			0.32 (0.27, 0.37)	0.25 (0.21, 0.29)		
Malawi		0.83 (0.76, 0.89)				0.52 (0.47, 0.58)	0.71 (0.64, 0.78)				
Nigeria	0.86 (0.80, 0.93)				0.83 (0.77, 0.90)						
Philippines			0.44 (0.40, 0.48)					0.46 (0.42, 0.51)			
Tajikistan								0.73 (0.61, 0.84)	0.79 (0.67, 0.92)	0.80 (0.65, 0.95)	0.73 (0.57, 0.90)
Uganda		0.84 (0.79, 0.90)			0.88 (0.83, 0.94)						
Uzbekistan							0.55 (0.51, 0.60)	0.50 (0.46, 0.54)	0.45 (0.41, 0.49)		

Supplemental Table S7: Comparison of results on vaccine hesitancy from the HFPS and other studies

**Comparison with de Figueiredo and Larson (2021)**		
	**HFPS**	**de Figueiredo and Larson**
Argentina	10.1%	24.3%
Brazil	3.1%	17.0%
Chile	3.4%	27.9%
Croatia	33.2%	58.5%
Ecuador	19.4%	20.3%
Indonesia	21.3%	17.1%
Lebanon	32.2%	55.9%
Malaysia	25.8%	13.9%
Mexico	6.2%	18.0%
Nigeria	15.2%	35.9%
Paraguay	15.3%	48.5%
Peru	10.5%	28.3%
Vietnam	15.9%	3.2%
Average	*16.3%*	*28.4%*
**Comparison with Wouters et al. 2021**		
	**HFPS**	**Wouters et al.**
Argentina	10.1%	24.0%
Brazil	3.1%	12.0%
Chile	3.4%	28.0%
Croatia	33.2%	59.0%
Ecuador	19.4%	20.0%
Indonesia	42.6%	17.0%
Lebanon	32.2%	56.0%
Mexico	6.2%	18.0%
Nigeria	30.3%	36.0%
Paraguay	15.3%	49.0%
Peru	10.5%	28.0%
Vietnam	15.9%	2.0%
Average	*18.5%*	*29.1%*
**Comparison with Gallup (2021)**		
**Country**	**HFPS Hesitancy**	**Gallup**
Argentina	10.1%	37.0%
Bolivia	24.2%	35.0%
Brazil	3.1%	30.0%
Burkina Faso	22.2%	44.0%
Chile	3.4%	40.0%
Colombia	11.2%	30.0%
Congo, Dem. Rep.	61.2%	61.2%
Congo, Rep.	14.4%	48.0%

Costa Rica	11.9%	26.0%
Croatia	33.2%	57.0%
Dominican Republic	5.0%	35.0%
Ecuador	19.4%	28.0%
El Salvador	8.1%	25.0%
Ethiopia	2.8%	16.0%
Georgia	66.8%	44.0%
Guatemala	29.6%	29.0%
Guinea	20.1%	46.0%
Honduras	13.9%	30.0%
Indonesia	21.3%	30.0%
Iraq	47.8%	39.0%
Jamaica	50.6%	68.0%
Kenya	17.9%	27.0%
Lao PDR	12.9%	16.0%
Lebanon	32.2%	57.0%
Malaysia	25.8%	28.0%
Mali	21.1%	51.0%
Mexico	6.2%	25.0%
Mongolia	19.2%	39.0%
Nicaragua	18.8%	13.0%
Nigeria	15.2%	42.0%
Paraguay	15.3%	47.0%
Peru	10.5%	27.0%
Philippines	54.9%	49.0%
Tajikistan	23.7%	36.0%
Thailand	36.6%	39.0%
Uganda	13.6%	38.0%
Uruguay	9.1%	39.0%
Uzbekistan	49.8%	30.0%
Vietnam	15.9%	19.0%
Zimbabwe	15.8%	27.0%
Average	*22.4%*	*36.2%*
**Comparison with Facebook (2021)**		
**Country**	**HFPS**	**FB**
Argentina	10.1%	21.5%
Belize	28.4%	48.0%
Brazil	3.1%	25.8%
Chile	3.4%	43.0%
Colombia	11.2%	15.7%
Costa Rica	11.9%	17.1%
Ecuador	19.4%	17.1%

El Salvador	8.1%	15.8%
Guatemala	29.6%	18.6%
Guyana	20.0%	54.4%
Honduras	13.9%	13.7%
Indonesia	21.3%	19.2%
Iraq	47.8%	52.8%
Lebanon	32.2%	22.3%
Mexico	6.2%	13.0%
Nicaragua	18.8%	37.4%
Panama	13.3%	24.9%
Paraguay	15.3%	16.2%
Peru	10.5%	12.2%
Uruguay	9.1%	50.8%
Average	*16.7%*	*27.0%*

Supplemental Figure S1: Vaccination rates by income group

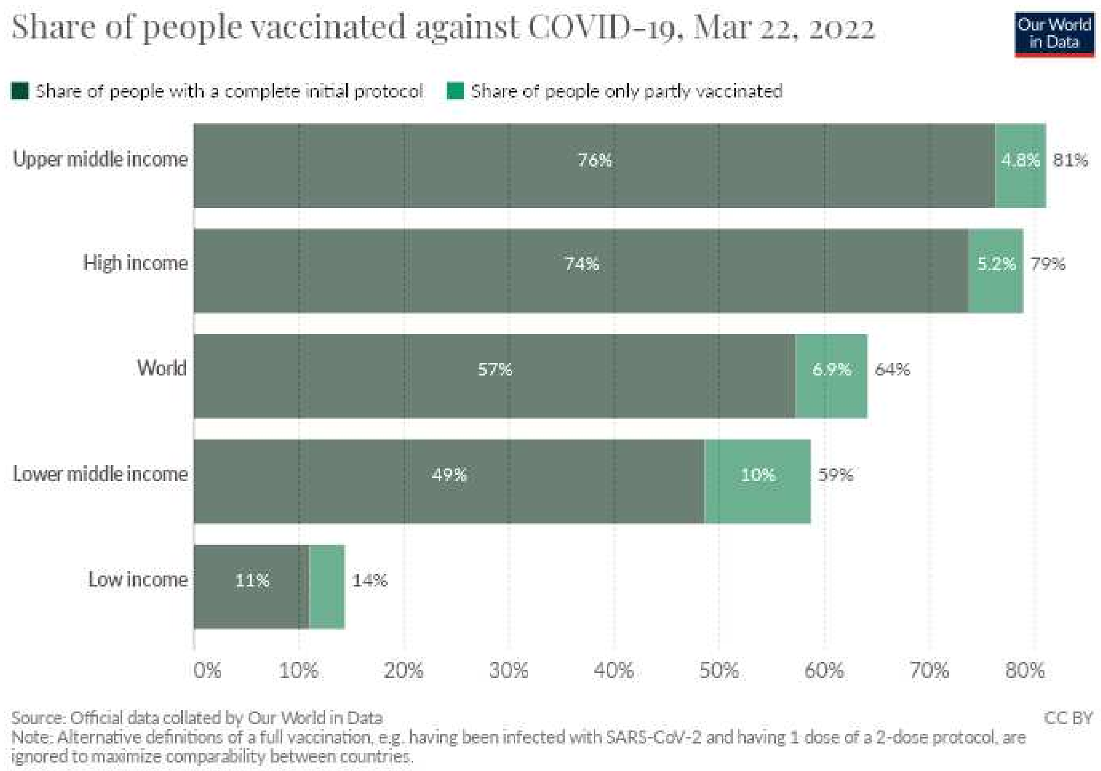

Data source: Ritchie et al. 2022 as of March 22, 2022.

Supplemental Figure S2: Vaccine rollout rates by income group

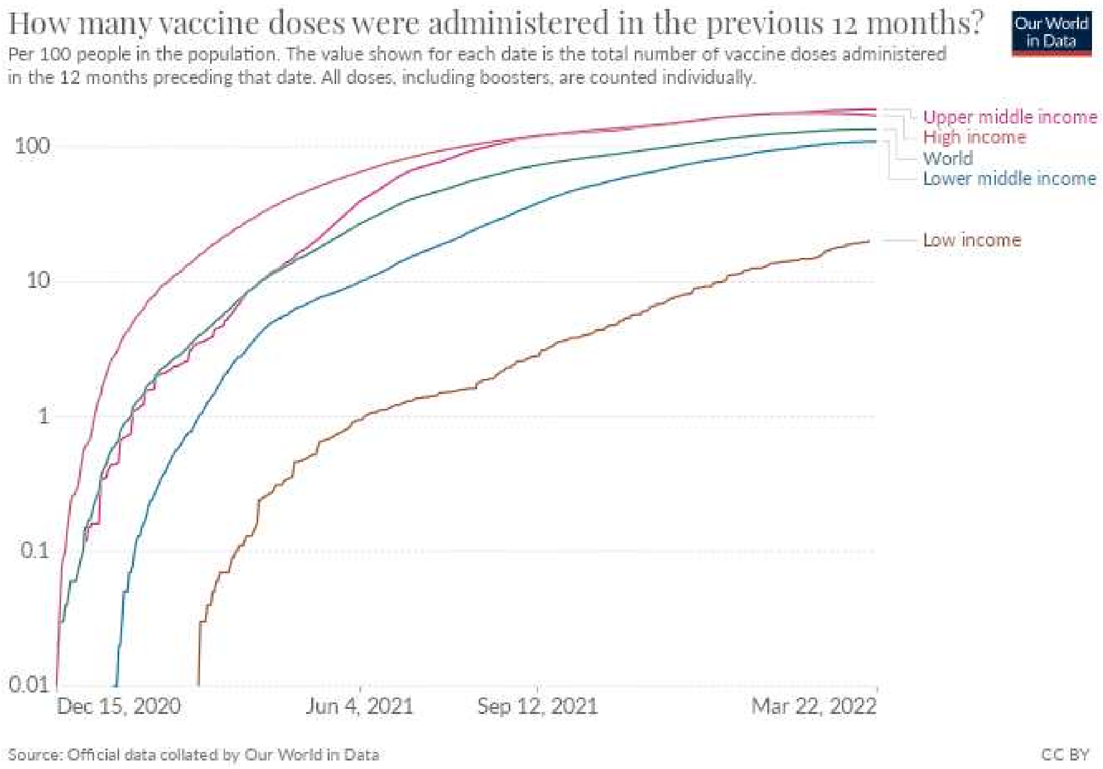

Data source: Ritchie et al. 2022 as of March 22, 2022.

Supplemental Figure S3: Comparison of share of population COVID-19 that is hesitant to take the COVID-19 vaccine, HFPS with other studies

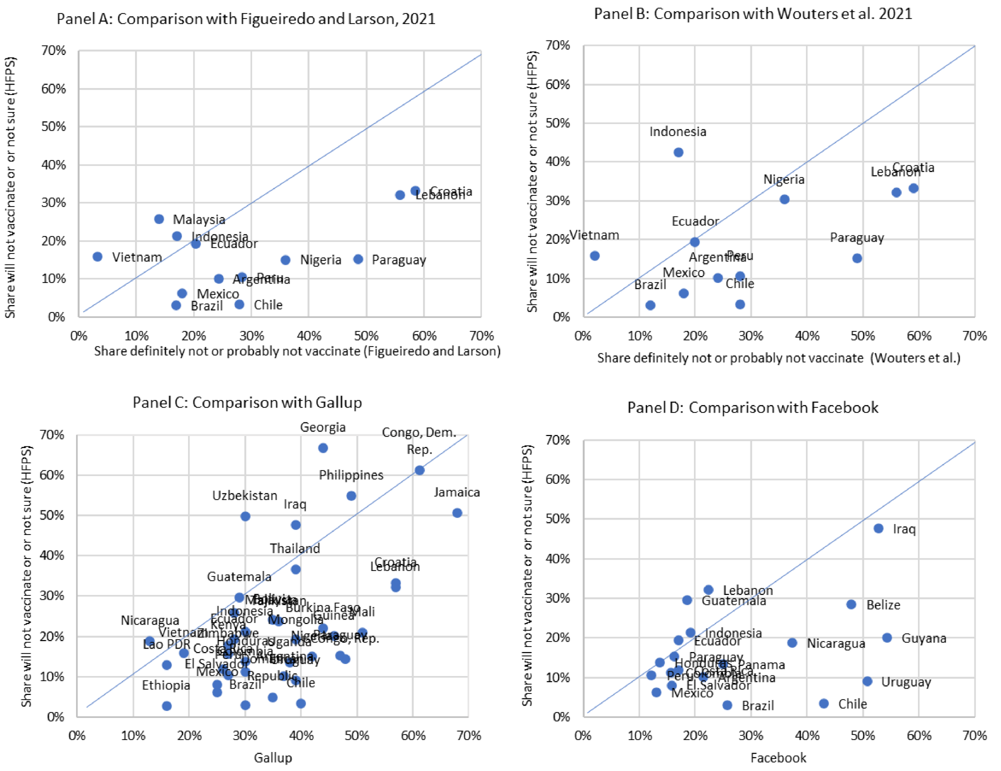

Notes: The y-axes indicate the share of the sample who reported not being willing or not sure if they will take the COVID-19 vaccine, as reported in the HFPS. The x-axes indicate the share of households in the survey that will definitely or probably not be vaccinated against COVID-19 from the following sources. Panel A: Figueiredo and Larson (2021). Panel B: Wouters et al. (2021). Panel C: Gallup (2021). Panel D: Facebook (2021).

Supplemental Figure S4: Comparison of COVID-19 Vaccine Hesitancy with pre-COVID sentiment about vaccines from 2019 Global Monitor, 45 countries

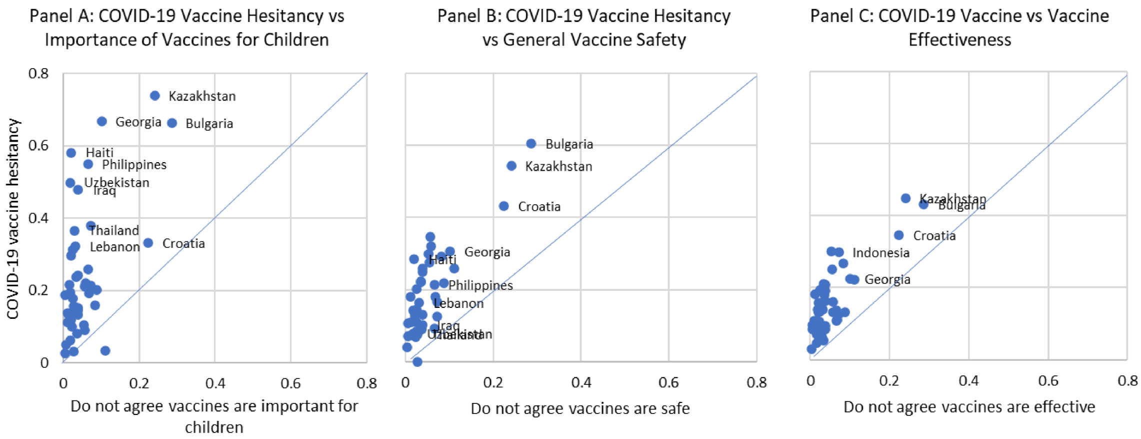

Notes: The y-axes indicate the share of the sample who report either not willing or not sure if they will vaccinate, as reported in the HFPS. The x-axes indicate the share of the 2019 Global Monitor Report sample that reported they do not agree vaccines are important for children (Panel A), do not agree that vaccines are safe (Panel B), and do not agree vaccines are effective.

Dayton Eberwein J, *et al. BMJ Open* 2023; 13:e069152. doi: 10.1136/bmjopen-2022-069152

Supplemental Figure S5: Reason for "definitely not" (upper panel) or "probably not" (lower panel) taking the COVID-19 vaccine, Facebook data for month of HFPS)

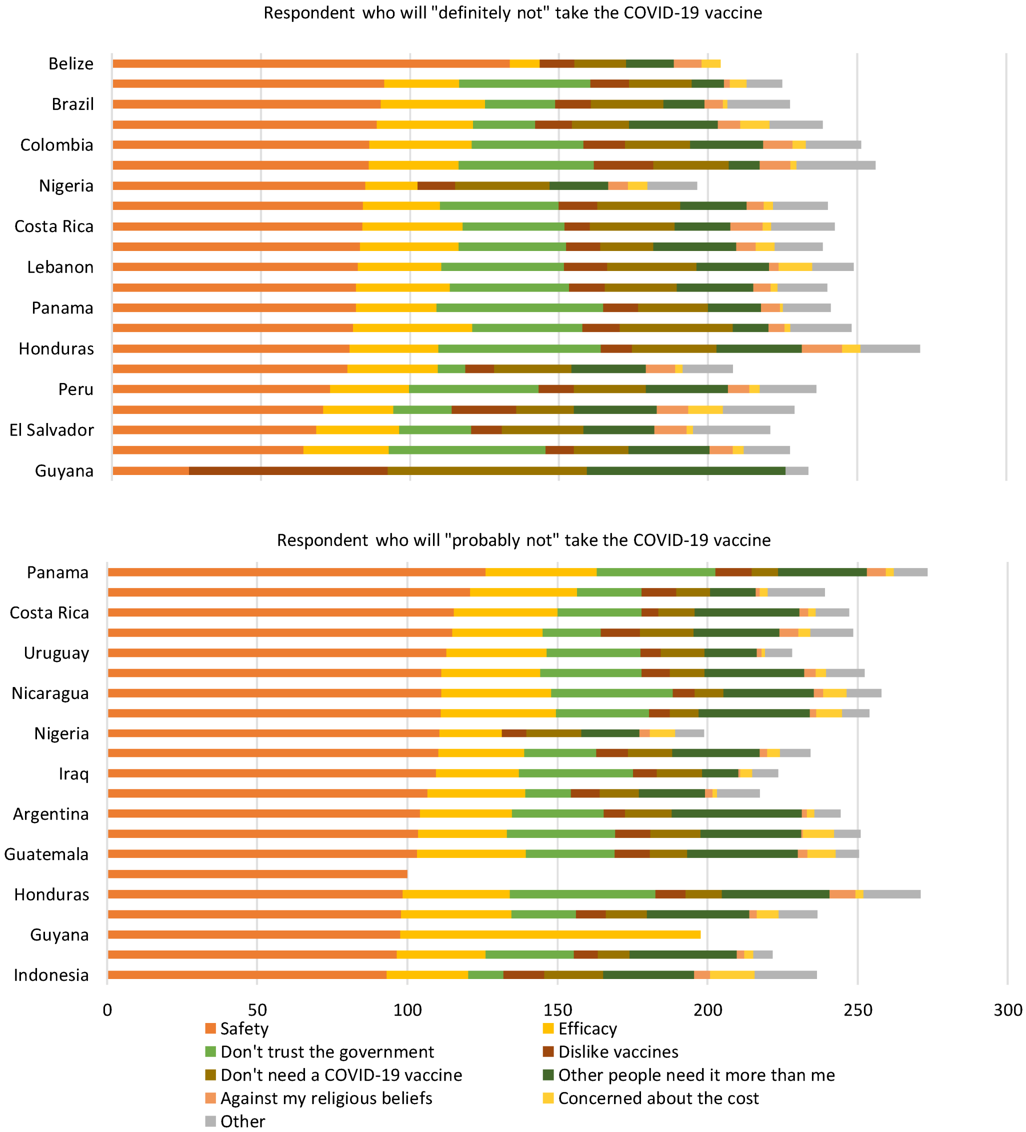

Notes: Y-axes indicate the share of survey respondents; multiple answers were allowed. Data source: Facebook (2021) for same month as reported in the HFPS. The month for each country is reported in Supplemental Table S1.

References- Supplemental Materials

de Figueiredo A, Larson HJ. Exploratory study of the global intent to accept COVID-19 vaccinations. Communications medicine. 2021 Sep 9;1(1):1-0.

Facebook. 2021. COVID-19 Trends and Impact Survey.

Gallup. 2019. Wellcome Global Monitor – First Wave Findings. https://wellcome.org/reports/wellcome-global-monitor/2018#downloads-4d1c.

Gallup. 2021. Gallup World Poll. https://www.gallup.com/178667/gallup-world-poll-work.aspx.

Hale T, Angrist N, Goldszmidt R, et al. 2022. A global panel database of pandemic policies (Oxford COVID-19 government response Tracker). Nat Hum Behav 2021;5:529–38. Database accessed Feb 28, 2022.

Inglehart, R, C. Haerpfer, A. Moreno et al. 2022. Would Values Survey: Round Seven – Country Pooled Datafile Version: https://www.worldvaluessurvey.org/WVSOnline.jsp. (accessed April 21, 2022) Madrid: JD Systems Institute.

Lazarus, JV, Ratzan SC , and Palayew A et al. 2021. A global survey of potential acceptance of a COVID-19 vaccine. Brief Communication. Nature Medicine 27: 225–228. https://doi.org/10.1038/s41591-020-1124-9.

Sallam M. COVID-19 Vaccine Hesitancy Worldwide: A Concise Systematic Review of Vaccine Acceptance Rates. Vaccines (Basel). 2021 Feb 16;9(2):160. doi: 10.3390/vaccines9020160. PMID: 33669441; PMCID: PMC7920465.

Solís Arce, J.S., Warren, S.S., Meriggi, N.F. *et al.* COVID-19 vaccine acceptance and hesitancy in low- and middle-income countries. *Nat Med* **27,** 1385–1394 (2021). https://doi.org/10.1038/s41591-021-01454-y.

World Bank. 2020. High Frequency Mobile Phone Surveys of Households to Assess the Impacts of COVID-19: Overview (English). Washington, D.C.: World Bank Group. http://documents.worldbank.org/curated/en/703571588695361920/Overview

World Bank. 2021. World Development Indicators. Washington, D.C.: The World Bank (producer and distributor). http://data.worldbank.org/data-catalog/world-development-indicators. Accessed March 12, 2022.

World Health Organization. 2022. Global Excess Deaths Associated with COVID-19 (Modelled Estimates). Downloaded on September 8, 2022 at https://www.who.int/data/sets/global-excess-deaths-associated-with-covid-19-modelled-estimates.

Wouters, O.J., Shadlen, K.C., Salcher-Konrad, M., Pollard, A.J., Larson, H.J., Teerawattananon, Y. and Jit, M., 2021. Challenges in ensuring global access to COVID-19 vaccines: production, affordability, allocation, and deployment. The Lancet, 397(10278), pp.1023-1034.

Dayton Eberwein J, *et al. BMJ Open* 2023; 13:e069152. doi: 10.1136/bmjopen-2022-069152
