# Supplementary material for: How prevalent is COVID-19 vaccine hesitancy in low-income and middle-income countries and what are the key drivers of hesitancy? Results from 53 countries
Source: BMJ Open. 2023 Nov 6;13(11):e069152. doi: 10.1136/bmjopen-2022-069152 (PMC10632876; doi:10.1136/bmjopen-2022-069152)
Supplement: Author's manuscript [file bmjopen-2022-069152.draft_revisions.pdf]

BMJ Open

BMJ Open is committed to open peer review. As part of this commitment we make the peer review history of every article we publish publicly available.

When an article is published we post the peer reviewers' comments and the authors' responses online. We also post the versions of the paper that were used during peer review. These are the versions that the peer review comments apply to.

The versions of the paper that follow are the versions that were submitted during the peer review process. They are not the versions of record or the final published versions. They should not be cited or distributed as the published version of this manuscript.

BMJ Open is an open access journal and the full, final, typeset and author-corrected version of record of the manuscript is available on our site with no access controls, subscription charges or pay-per-view fees (http://bmjopen.bmj.com).

If you have any questions on BMJ Open's open peer review process please email info.bmjopen@bmj.com

BMJ Open

COVID-19 Vaccine Hesitancy in 53 Developing Countries: Levels, Trends, and Reasons for Hesitancy

Journal:	*BMJ Open*
Manuscript ID	bmjopen-2022-069152
Article Type:	Original research
Date Submitted by the Author:	17-Oct-2022

Complete List of Authors:	Dayton Eberwein, Julia; World Bank Group, HNP GP<br>Edochie, Ifeanyi; World Bank Group<br>Newhouse, David; World Bank Group<br>Cojocaru, Alexandru; World Bank Group<br>Bopahbe, Gildas; World Bank Group<br>Kakietek, Jakub; World Bank Group<br>Kim, Yeon Soo; World Bank Group<br>Montes, Jose; World Bank Group

Keywords:	COVID-19, Health policy < HEALTH SERVICES ADMINISTRATION & MANAGEMENT, Public health < INFECTIOUS DISEASES

SCHOLARONE™
Manuscripts

I, the Submitting Author has the right to grant and does grant on behalf of all authors of the Work (as defined in the below author licence), an exclusive licence and/or a non-exclusive licence for contributions from authors who are: i) UK Crown employees; ii) where BMJ has agreed a CC-BY licence shall apply, and/or iii) in accordance with the terms applicable for US Federal Government officers or employees acting as part of their official duties; on a worldwide, perpetual, irrevocable, royalty-free basis to BMJ Publishing Group Ltd ("BMJ") its licensees and where the relevant Journal is co-owned by BMJ to the co-owners of the Journal, to publish the Work in this journal and any other BMJ products and to exploit all rights, as set out in our licence.

The Submitting Author accepts and understands that any supply made under these terms is made by BMJ to the Submitting Author unless you are acting as an employee on behalf of your employer or a postgraduate student of an affiliated institution which is paying any applicable article publishing charge ("APC") for Open Access articles. Where the Submitting Author wishes to make the Work available on an Open Access basis (and intends to pay the relevant APC), the terms of reuse of such Open Access shall be governed by a Creative Commons licence – details of these licences and which Creative Commons licence will apply to this Work are set out in our licence referred to above.

Other than as permitted in any relevant BMJ Author's Self Archiving Policies, I confirm this Work has not been accepted for publication elsewhere, is not being considered for publication elsewhere and does not duplicate material already published. I confirm all authors consent to publication of this Work and authorise the granting of this licence.

For peer review only

COVID-19 Vaccine Hesitancy in 53 Developing Countries: Levels, Trends, and Reasons for Hesitancy

Julia Dayton Eberwein,[1*] Ifeanyi Edochie,[1] David Newhouse, [1] Alexandru Cojocaru, [1] Gildas Deudibe, [1] Jakub Kakietek,[1] Yeon Soo Kim, [1] and Jose Montes[1]

September 2022

Abstract

Objectives: To estimate the levels of COVID-19 vaccine hesitancy in 53 developing countries, differences across population groups in hesitancy, and self-reported reasons for being hesitant to take the COVID-19 vaccine.

Methods: This paper presents new evidence on levels and trends of vaccine hesitancy in developing countries based on harmonized high-frequency phone surveys from more than 120,000 respondents in 53 low- and middle-income countries collected between October 2020 August 2021. These countries represent a combined 53 percent of the population of low- and middle-income countries excluding India and China.

Results: On average across countries, one in five adults reported being hesitant to take the COVID-19 vaccine, with the most cited reasons for hesitancy being concerns about the safety of the vaccine, followed by concerns about its efficacy. Between late 2020 and the first half of 2021, there tended to be little change in hesitancy rates in 11 of the 14 countries with available data, while hesitancy increased in Iraq, Malawi, and Uzbekistan. COVID-19 vaccine hesitancy was higher among female, younger adults, and less educated respondents, after controlling for selected observable characteristics.

Conclusions: Country estimates of vaccine hesitancy from the high-frequency phone surveys are correlated but lower than those from earlier studies, which often relied on less representative survey samples. The results suggest that vaccine hesitancy in developing countries, while less prevalent than previously thought, will be an important and enduring obstacle to recovery from the pandemic.

[1] The World Bank, 1818 H. Street NW, Washington, DC 20433

* Corresponding author: jdayton@worldbank.org.

For peer review only - http://bmjopen.bmj.com/site/about/guidelines.xhtml

Strengths and limitations of this study

- This study provides estimates of vaccine hesitancy from a large sample of households in 53 developing countries that are comparable across countries and largely based on household surveys that are representative of the national population. It fills a gap in the literature of COVID-19 vaccine hesitancy in developing countries, a part of the world that is under-represented in the literature.
- The study investigates heterogeneity in hesitancy across demographic characteristics of the respondent, as well as reasons for their hesitancy to take the COVID-19 vaccine, thus providing a rich set of results which can aid policy makers in the design of vaccination campaigns in developing countries.
- One potential limitation of this study is that it is based on phone survey data which exclude respondents who did not have access to a phone. However, in the countries in this sample, access to mobile phones was high, and sampling weights were used to correct for the biases resulting from non-random access to phones.

For peer review only

For peer review only - http://bmjopen.bmj.com/site/about/guidelines.xhtml

1. Introduction

The world is entering the third year of the global COVID-19 pandemic, which has caused enormous devastation to both people's health (450 million cases and 6 million deaths as of March 2022) and national economies, in the form of a global recession that has pushed millions into poverty (1,2). With the continued emergence of new variants and limited treatments available, it is commonly accepted that widespread vaccination is the world's best bet to contain the virus and it is also expected to play an important role in economic recovery, as widespread vaccinations would enable economic recovery (3,4). As of early March 2022, over 60 percent of the world's population had received at least one dose of a COVID-19 vaccine. However, there are stark disparities in vaccination rates across countries: only 14 percent of people in low-income countries had received at least one dose as of March 22, 2022, compared to 79 percent in high-income countries and 81 percent in upper-middle income countries (5)(supplemental figures S1 and S2). The lag in vaccine distribution in lower income countries had initially put the focus mainly on supply-side constraints until recently. However, with vaccine production now exceeding demand, it is increasingly important to understand the extent of vaccine hesitancy and the nature of the concerns.

Vaccine hesitancy refers to delay in acceptance or refusal to vaccinate despite availability of vaccination services (6). It is not a new phenomenon, and some level of hesitancy exists for most vaccines; even before the COVID-19 pandemic began, the World Health Organization (WHO) identified vaccine hesitancy as one of the top threats to global health (WHO 2019). Anecdotally, several factors likely contribute to COVID-19 vaccine hesitancy: the accelerated speed of development of the COVID-19 vaccine, its newness, concerns about longer-term side-effects, uncertainty about the duration of the vaccine's effectiveness, and the type of vaccine (e.g., mRNA) and country of origin of the vaccine (7-10).

The multi-country studies that exist show relatively high levels of hesitancy to take the COVID-19 vaccine, but mainly focus on high-income countries (11-12). Less is understood about COVID-19 vaccine hesitancy in low- and middle-income countries. A few studies reported multi-country survey results from developing countries (12-18) or systematic reviews of individual country surveys (19), but few of the studies were based on nationally representative samples of respondents or included comparable results for many countries. Taken together, the available literature from low- and middle-income countries suggests that vaccine acceptance is higher in East and South Asia and Latin America and lower in the Middle East and Eastern and Central Asia. Results from Africa were mixed, as were results for different subpopulations. Some studies indicated women were more hesitant than men, younger respondents were more hesitant than older respondents, and those with less education were more hesitant than persons with more education. The predominant reason for hesitancy was concern over side effects from the vaccine. Given the limited availability of randomly selected samples that are representative at the national level, an important contribution of this analysis will be to validate these findings.

This paper extends the existing literature by providing estimates of and reasons for vaccine hesitancy for 53 developing countries, which are comparable across countries and largely based on household surveys that are more representative of the national population. The 53 countries represent approximately 30% of the population of all low- and middle-income countries and 53% excluding India and China.

For peer review only - http://bmjopen.bmj.com/site/about/guidelines.xhtml

2. Data and Methods

2a. Data

This study describes the levels of vaccine hesitancy and its reasons in 53 developing countries between October 2020 August 2021 using data from the World Bank's COVID-19 high-frequency phone surveys (HFPS) which were implemented to monitor the impact of COVID-19 on households around the world (20). Data are available for one survey round from 39 countries and two or more survey rounds for 14 countries (supplemental table S1). The sampling frame was drawn from pre-existing nationally representative household surveys in 19 countries, random digit dialing (RDD) in 29 countries, and a list of phone numbers typically obtained from mobile phone operators in 5 countries (see supplement 1 for detailed description of methods).

2b. Outcome measures

The questions asked about vaccine hesitancy in the surveys varied depending on whether a vaccine was available in the country at the time of the survey. In surveys that took place before the vaccine rollout had begun in that country, the question asked was "If an approved vaccine to prevent COVID-19 was to become available at no cost, would you agree to be vaccinated?" In surveys that took place after the vaccine rollout had begun, the wording of the question was either "Are you planning to be vaccinated?" or "When a vaccine to prevent COVID-19 is available to you, are you planning to be vaccinated?" In the sample, 47 countries offered three answer options (yes, not sure, and no), while the other six offered only two categories (yes and no). We combine the "no" and "not sure" answers to obtain the measure of vaccine hesitancy.

To obtain the respondent's reason for vaccine hesitancy, survey respondents who answered "no" or "not sure" were asked "What is your (main) reason/concern for not wanting to be vaccinated / not being sure if you want to be vaccinated?" The answer categories varied widely across surveys (supplemental table S2a). To make these more comparable across countries, answers were remapped into the nine most common categories (supplemental table 2b). The surveys also differed across countries in terms of whether a single concern or multiple concerns were collected, and to account for this, the results are presented separately.

2c. Contextual data

Country-level contextual data were drawn from other sources: new COVID-19 cases per million, measured as a 7-day rolling average prior to the midpoint of the month prior to survey data collection in each country (1); and the Oxford Stringency Index, ranging from 0–100, that indicates the degree to which various restrictions were put in place by governments to control the pandemic (21); confidence in the press, the government, and the WHO (22); and estimated excess deaths due to COVID-19 (23).

2d. Analytical strategy

Data from 53 countries were pooled into a single data set. Sampling weights were scaled such that countries are weighted equally; in other words, the estimates of vaccine hesitancy are unweighted averages of population-weighted country averages. (Using population weights when aggregating across countries would have resulted in the results being driven by a small number of large countries.) First, we report point estimates and 95% confidence intervals for each country based on standard errors clustered at the state or province level within each country. We also report the simple average across all countries and stratified by: (i) World Bank region; (ii) World Bank country income group; (iii) urban vs. rural

For peer review only - http://bmjopen.bmj.com/site/about/guidelines.xhtml

residence; (iv) gender of respondent; (v) whether respondent is head of household; (vi) age of the respondent (under 35 years, 35-64 years, and 65 years and older); and (vii) educational attainment of the respondent (some primary school, some secondary school, some tertiary). Tukey's test of multiple comparisons is used to test differences in rates of vaccine hesitancy across groups. Second, we use multivariate regression analysis to assess the relative association of vaccine hesitancy and the correlates and contextual variables. Trends over time in levels of vaccine hesitancy are examined in 14 countries with more than one wave of results available. Finally, the analysis describes the reasons for vaccine hesitancy.

2e. Patient and public involvement

No patients were involved in this study. The results of the study will be disseminated to key policy- makers and relevant stakeholders involved in COVID- 19 vaccine deployment.

3. Results

3a. Levels of vaccine hesitancy across 53 countries

Overall, the average level of vaccine hesitancy across the most recent survey round in 53 countries was 20.0% (Confidence Intervals (CI) 17.2-22.7%). Across regions, average levels of hesitancy were highest in Europe and Central Asia (58.8%, CI 55.0-62.6%), followed by the Middle East and North Africa (47.4%, CI 38.8-56.0%), East Asia and Pacific (26.2%, CI 21.4-31.0%), Sub-Saharan Africa (15.5%, CI 11.8-19.2%), and Latin America and the Caribbean (8.0%, CI 6.5-9.5%) (Figure 1 and supplemental table S3). Differences between all regions were statistically significant at the 5% level (supplemental table S4). When considered by country income group, the highest average level of hesitancy was in lower middle-income countries (27.7%, CI 23.8-31.7%), followed by low-income countries (14.6%, CI 7.8-21.4%), upper middle-income countries (12.7%, CI 9.8-15.6%), and high-income countries (5.9%, CI 3.4-8.4). Differences in hesitancy across country income groups were significant at the 5% level across all pairs except for the comparison between low-income and upper-middle income groups.

3b. Bivariate correlates of vaccine hesitancy

Rural households reported significantly higher levels of vaccine hesitancy than urban households (23.2% vs. 17.7%, p-value=0.018), and female respondents (22.5%) were significantly more likely than male respondents (17.3%) to be vaccine hesitant (p-value=0.021). Among households in the 33 surveys with information on the educational attainment of the respondent, respondents with lower levels of education had on average higher levels of hesitancy: 22.8% of respondents with no education were hesitant, compared with 19.8% of those with any primary education, 19.0% of respondents with any secondary education, and 13.7% of those with some tertiary education. Those with at least some tertiary education were significantly less likely to be hesitant than those with no education (p-value=0.027), some primary (p-value=0.019), and some secondary (p-value=0.052). Younger respondents reported higher levels of hesitancy than older respondents (20.3% of respondents under 35 years; 20.1% of respondents aged 35-59 years, and 17.7% of over 60 years), but none of these differences were significant.

3c. Vaccine hesitancy by country

The highest levels of vaccine hesitancy were reported in Kazakhstan (75.3%), Bulgaria (66.2%) and Georgia (65.2%), and the lowest levels in Chile (3.4%), Ethiopia (3.4%), and Brazil (3.1%) (Figure 2). Strength of

For peer review only - http://bmjopen.bmj.com/site/about/guidelines.xhtml

vaccine hesitancy sentiment is shown for the 46 countries that included three answer options (yes, not sure, and no). In four countries (Philippines, Georgia, Jamaica, and Kazakhstan), over 20% of respondents reported being unsure of whether they would get vaccinated, and in another 11 countries, between 10% and 20% of the sample was unsure whether they would be willing to be vaccinated against COVID-19.

3c. Multivariate analysis of correlates of vaccine hesitancy

Results from a multivariate regression generally confirmed the bivariate results. Results from ordinary least squares (OLS) regression models are shown in Table 1: a baseline model with the correlates described above plus month of survey (expressed in terciles),[1] and extended models with additional contextual variables (expressed in terciles). Results were consistent across models, as are the marginal effects from logit regressions (supplemental Table S5a). Men were less COVID-19 vaccine hesitant than women, although the magnitude was small. Having less formal education was associated with being more hesitant and the differences were more substantial. Respondents with no formal education were 12.3 percentage points more likely to be hesitant than respondents with some tertiary education. Those with some primary or some secondary education were 6.1 and 6.4 percentage points more likely, respectively. Age is inversely correlated with hesitancy – adults over 65 years of age were 10.3 percentage points less likely to be hesitant compared with respondents under the age of 35 years, and respondents aged 35-64 years were 4.7 percentage points less likely. There were no statistically significant differences between hesitancy among rural and urban respondents. Differences across regions were statistically significant and, for some regions, quite large: those living in Europe and Central Asia were 36.3 percentage points more likely to be hesitant than those living in the reference region of Latin America and the Caribbean, those in the Middle East and North Africa were 23.1 percentage points more likely and those in East Asia and the Pacific, 21.1 percentage points more likely. Differences between Sub-Saharan Africa and Latin America and the Caribbean were not statistically significant, nor were differences by country income group. Over time, the probability of being hesitant to take the COVID-19 vaccine declined but not significantly.

For peer review only

---

[1] Time of survey was split into the following 3 groups: (i) November 2020 – January 2021; (ii) March – May 2021; and (iii) June – August 2021. There are no observations in the data for February and April of 2021.

For peer review only - http://bmjopen.bmj.com/site/about/guidelines.xhtml

Table 1: Multivariate analysis of vaccine hesitancy in 53 developing countries (Ordinary least squares)

Dep. Var: Hesitancy	(1)	(2)	(3)	(4)	(5)	(6)
Male	-0.028*	-0.027*	-0.028	-0.027*	-0.031*	-0.038**
	(0.013)	(0.013)	(0.014)	(0.013)	(0.013)	(0.012)
Head of HH	0.012	0.009	0.019	0.000	0.011	0.007
	(0.015)	(0.014)	(0.013)	(0.017)	(0.015)	(0.013)
Education of respondent (ref –						
No education	0.123***	0.125**	0.116**	0.134**	0.125**	0.143**
	(0.033)	(0.035)	(0.032)	(0.037)	(0.040)	(0.036)
Any primary	0.061**	0.063**	0.060**	0.067**	0.052*	0.070**
	(0.018)	(0.019)	(0.018)	(0.019)	(0.020)	(0.021)
Any secondary	0.064***	0.065**	0.062**	0.065**	0.058**	0.062**
	(0.011)	(0.011)	(0.011)	(0.012)	(0.011)	(0.011)
Age group (ref. -- 34 and						
Working age (35-64)	-0.047**	-	-0.042**	-	-0.045**	-
	(0.014)	(0.013)	(0.015)	(0.013)	(0.015)	(0.012)
Retirement age (65+)	-0.103***	-	-	-	-	-
	(0.023)	(0.024)	(0.023)	(0.022)	(0.024)	(0.023)
Rural area	0.016	0.014	0.013	0.010	0.012	0.011
	(0.019)	(0.018)	(0.018)	(0.015)	(0.015)	(0.011)
Region (ref – LAC)						
EAP	0.211**	0.218*	0.241**	0.127	0.299	0.249
	(0.076)	(0.086)	(0.084)	(0.076)	(0.155)	(0.137)
ECA	0.363***	0.364**	0.347**	0.376**	0.369**	0.345**
	(0.075)	(0.076)	(0.073)	(0.076)	(0.056)	(0.068)
MNA	0.231***	0.230**	0.262**	0.240**	0.288**	0.393**
	(0.043)	(0.043)	(0.062)	(0.036)	(0.059)	(0.050)
SSA	0.082	0.089	0.078	-0.005	0.072	0.057
	(0.075)	(0.079)	(0.074)	(0.101)	(0.086)	(0.103)
Income group (ref. – LIC)						
LMIC	-0.073	-0.071	-0.052	-0.090	-0.049	-0.053
	(0.127)	(0.124)	(0.122)	(0.129)	(0.113)	(0.081)
UMIC	-0.052	-0.050	-0.071	-0.096	-0.026	-0.130
	(0.154)	(0.154)	(0.146)	(0.154)	(0.135)	(0.107)
HIC	-0.161	-0.160	-0.183	-0.208	-0.175	-0.280*
	(0.159)	(0.162)	(0.153)	(0.162)	(0.136)	(0.108)
Survey month (ref -- Nov 2020 - Jan 2021)						
March - May 2021	-0.073	-0.062	-0.102	-0.056	-0.113	-0.110
	(0.095)	(0.108)	(0.104)	(0.101)	(0.112)	(0.095)
June - August 2021	-0.018	-0.012	-0.018	-0.028	-0.049	-0.026
	(0.090)	(0.099)	(0.099)	(0.089)	(0.097)	(0.079)
New COVID-19 cases per						
		-0.022				0.023
Cases (middle tercile)		(0.065)				(0.057)
		-0.009				-0.011
Cases (top tercile)		(0.054)				(0.049)
Oxford stringency index terciles (ref. – bottom tercile)						
Stringency (middle			0.061			0.148
			(0.073)			(0.077)
Stringency (top tercile)			-0.005			0.028
			(0.060)			(0.059)
WHO excess deaths due to COVID-19 (ref.- bottom tercile)						
Excess deaths (middle				-0.110*		-0.123
				(0.050)		(0.080)

For peer review only - http://bmjopen.bmj.com/site/about/guidelines.xhtml

Dep. Var: Hesitancy	(1)	(2)	(3)	(4)	(5)	(6)
Excess deaths (top				-0.059		0.045
				(0.067)		(0.106)
Confidence in government index tercile (ref.- top tercile)						
Confidence in					0.073	0.105
					(0.054)	(0.064)
Confidence in					0.016	0.055
					(0.131)	(0.135)
Constant	0.282*	0.286*	0.265	0.395**	0.206	0.190
	(0.136)	(0.140)	(0.141)	(0.143)	(0.149)	(0.145)
R-squared	0.090	0.090	0.095	0.097	0.102	0.123
N	65088	65088	65088	65088	65088	65088

Notes: Weighted logit regressions. Marginal effects reported. Standard errors clustered at country level. *, **, *** indicates significance at the 95%, 99%, and 99.9% level.

In the extended regression models, we tested two additional hypotheses. First, we expected that the severity of the pandemic (for which we use three different proxies: number of new cases of COVID-19 in the country in column 2, the degree of stringency in the government's policy measures as captured by the Oxford Stringency index in column 3, and estimated COVID-19 related excess deaths in column 4) would be negatively correlated with hesitancy. In our data, none of the three proxies for pandemic severity and/or visibility is statistically associated with vaccine hesitancy with the exception of COVID-19 excess deaths, where in countries in the second tercile of the excess deaths distribution vaccine hesitancy is, on average, lower, relative to the bottom tercile baseline. We also test whether trust in the government in general is associated with COVID-19 vaccine hesitancy, expecting a positive association due to widespread reporting of misinformation and antivax messages reported in the press, especially online. However, this association is not statistically significant in the regression model (column 5).

Finally, we also disaggregate the vaccine hesitant population into those who say that they would not get a COVID-19 vaccine and those who were not sure and consider whether the underlying characteristics of these two subgroups differ. To do so, we consider vaccine hesitancy as being a spectrum that spans from a rejection of the vaccine to the acceptance of the vaccine, with uncertainty being an intermediate category. This allows us to model the vaccine decision as an ordinal model, keeping the extended specification from table 1 (supplemental table S5b). When we examine the correlates of the "No" and the "Not sure" groups separately, we observe similar patterns, but they are much weaker in terms of magnitudes for the "Not sure" group. For instance, lower levels of education are associated with a higher probability of answering No, and this is true for the Not sure group, but the magnitude of the marginal effects is much smaller for the latter. Likewise, the probability of answering "Not sure" is also higher among the working age and older population, relative to those under the age of 35, but the strength of these negative correlations is considerably smaller. The Europe and Central Asia, Middle East and North Africa, and East Asia and Pacific regions have a higher probability of both No and Not sure answers relative to the Latin American and Caribbean region baseline.

3d. Changes in vaccine hesitancy over time

Estimates of vaccine hesitancy were available for two or more survey rounds in 14 countries in the sample (figure 3 and supplemental table S6). There were no clear patterns in terms of the changes in vaccine hesitancy in countries with multiple data points over the pandemic. COVID-19 vaccine hesitancy declined in half of the countries and increased in the other half, and changes in either direction were less than five

For peer review only - http://bmjopen.bmj.com/site/about/guidelines.xhtml

percentage points in all but three countries However, larger increases were observed in Iraq (30% increase), Malawi (36% increase), and Uzbekistan (18% increase). No patterns of change over time were observed across sub-populations either.

3e. Reasons for COVID-19 vaccine hesitancy

Overall, the most common concern pertained to the safety of the vaccine, including concerns about side effects (figure 4). Among the 42 countries with the single answer option, an average of 43% of respondents cited safety concerns as the primary reason for not planning to be vaccinated (figure 4, panel b). Among countries with multiple answer options, a similar pattern was observed: concerns about safety were the most common concern cited in five of the seven countries (figure 4, panel b). Other commonly cited concerns included efficacy of the vaccine (19%), dislike of vaccines in general (9%), a preference for natural immunity (which includes perceiving self as low risk and already having had COVID-19 illness) (8%), lack of knowledge about or access to the vaccine (6%), lack of trust in the government/pharmaceutical industry/international community (5%), religious reasons (4%), perceiving oneself as not eligible (which includes counterindication) (4%) and other reasons, which included concerns about getting COVID-19 at the facility and other unspecified reasons (9%). Supply-side concerns for not getting vaccinated only accounted for 6% on average across all surveys with the single answer option. One exception was seen in several Latin American countries where over 10% of respondents reported the concerns “health center too far or hard to reach” or “I don't have time to go to get vaccinated.”

These averages mask significant variation across countries. Concerns about vaccine efficacy were greater or equal to concerns about safety in five Latin American countries (Argentina, Chile, Dominica, Dominican Republic, and Mexico). Respondents in the Democratic Republic of Congo and the Republic of Congo were hesitant because of dislike for vaccines in general (40% and 44% in March 2021 of hesitant respondents, respectively) and distrust for government, pharmaceutical industry, and the international community (40% and 22% in March 2021, respectively). In Georgia, the only country in Europe and Central Asia with information on reasons for hesitancy, the most cited reason was “not eligible.”

Only one country, the Republic of Congo, reported reasons for two waves. Between December 2020 and March 2021, only small changes in reasons were seen: the share of respondents who cited distrust of the international community declined from 30% to 22%, and the share who cited concerns about safety and dislike of vaccines in general increased (from 27% to 31% and 42 to 44%, respectively).

Discussion

4a. Principal findings

On average across 53 countries, one in five adults were hesitant about getting a COVID-19 vaccine. The highest levels were observed in Eastern European and Central Asian countries and the lowest levels in Latin American and Caribbean countries. Among population groups, female respondents, younger adults, and those with less formal education reported higher levels of COVID-19 vaccine hesitancy than their respective counterparts. Between October 2020 and August 2021, there tended to be little change in levels of hesitancy except in Iraq, Malawi, and Uzbekistan, where hesitancy increased. The main self-reported reason for being hesitant was concerns over safety, especially worries about side effects, and to a lesser extent, concerns over vaccine efficacy and dislike of vaccines in general.

For peer review only - http://bmjopen.bmj.com/site/about/guidelines.xhtml

4b. Strengths and comparisons with other studies

This study has several advantages over the existing literature. It is based on a very large sample of respondents from national surveys in 53 developing countries, a part of the world that is under-represented in the literature. Over half of the surveys used random digit dialing, typically in upper middle-income countries in which a large share of the population uses mobile phones. In lower income contexts, most surveys, 35 percent overall, sampled based on previous face-to-face surveys which were in turn drawn from a census frame. In the remaining 10 percent of cases, sampling was carried out from lists provided by mobile phone operators. In each case, surveys drawn from pre-existing face-to-face surveys were reweighted using the baseline data to become more representative, while many random digit dialing surveys were also reweighted to make those samples more representative of the universe of phone numbers. While it is impossible to eliminate issues of representativeness in phone surveys, the HFPSs were typically carried out by National Statistics Offices and are more plausibly representative than convenience web surveys.

Overall, the estimates of COVID-19 vaccine hesitancy from the HFPSs were more conservative than other published estimates (supplemental figures S3). There are several possible explanations for the differences. The first relates to the timing of data collection. Most of the existing literature is based on surveys carried out in mid to late 2020 (12, 13, 15, 17-18), whereas the HFPS data included in our sample were collected from end-2020 through August 2021. It is plausible to believe COVID-19 vaccine hesitancy generally declined during this period as multiple vaccines became available and widespread vaccination was safely rolled out in high-income countries. Another possible reason for the differences is the variation in how the vaccine hesitancy question was framed and the response options available. For example, . some studies used a four or five point Likert scale of agreement (12, 14, 13, 18, 24), whereas the HFPS relied on two or three answer options. Finally, variations in survey modality may have created biases. Among the published studies included for comparison, many studies were based on data from commercial online sample providers, often using quotas sampling to ensure an appropriate distribution in terms of gender, age, and region (13, 24, 14, 15, 12, 18), while others were based on convenience samples (9,25-27), or a mix of methods (17, 19). There was also wide variety in the survey mode, including online, computer-assisted telephone, and face-to-face surveys.

It is not possible to know the extent to which each of the above-mentioned potential sources of bias affect the comparisons of the findings from the HFPS with the others. For example, it was possible to match the Facebook survey results for the month and year of the HFPS for 20 countries, thus taking away any differences in the timing of the survey. Nevertheless, the HFPS estimates were still lower than those reported by Facebook (supplemental figure S3, panel D), and this could be due to differences in question-and-answer wording and/or the sample frame.

Even though the HFPS estimates are lower than other sources, the levels of COVID-19 vaccine hesitancy reported here are nonetheless higher than the levels of vaccine hesitancy reported for other vaccines. We compared the average country levels of COVID-19 vaccine hesitancy from this study with results from 2019 Global Monitor for 45 countries with observations in both studies (28). Overall, the levels of COVID-19 vaccine hesitancy reported here are higher than concerns about vaccines in general (supplemental figure S4). However, there are also similarities in the pattern: countries in Europe and Central Asia and some Sub-Sahara African countries reported the highest levels of vaccine hesitancy even before

For peer review only - http://bmjopen.bmj.com/site/about/guidelines.xhtml

the pandemic, and the biggest reason for pre-COVID-19 vaccine hesitancy was concerns about safety.

Nevertheless, the higher levels of hesitancy reported for the COVID-19 vaccine suggest that respondents are more concerned about the COVID-19 vaccine than about childhood vaccines. The result is even more striking considering that the COVID-19 vaccine hesitancy is estimated rather conservatively in the HFPS, when compared against estimates from other sources. Possible reasons for the higher rates of COVID-19 vaccine hesitancy include that it is a very new vaccine that uses an innovative technology (mRNA), it was rapidly developed with a streamlined approval process, and may have unknown long-term effects. This is consistent with the reasons given by respondents, which are mostly around safety, and to a lesser extent efficacy.

Our findings that women, younger adults, and those with less education are more vaccine hesitant are largely consistent with results presented elsewhere (13, 17). Although Lazarus and colleagues found that men were slightly more hesitant than women, the gender difference was small (12). The findings with respect to age were also similar—the youngest adults were significantly less willing to be vaccinated than adults over 65 years of age (12-13). The low levels of vaccine hesitancy reported among the oldest adults provides an important opportunity for vaccine campaigns to target this demographic, especially given recent research that targeting vaccines to older age groups saves the most lives and is highly cost-effective (28-27). The findings on education are also consistent with other studies (12-13), although Solis-Arce and colleagues reported mixed results (17). Knowing that citizens who are younger and with less formal education are the most likely to be vaccine hesitant can help vaccination campaigns target these population groups.

Although we hypothesized that confidence in the government in general would be negatively associated with vaccine hesitancy, this association was not statistically significant in the regression analysis. This is likely because trust indicators were only available at the national level, making the estimates imprecise. Previous studies have shown mixed results (12-13, 19), and future analysis is needed to investigate this relationship, preferably using an individual level measure of trust in government.

With respect to our results on changes over time, which showed that changes were minimal in all countries except Iraq, Malawi, and Uzbekistan, where we report relatively large increases. It is difficult to identify the factors that led to increased hesitancy in these cases. The larger body on changes over time in vaccine hesitancy during the same timeframe as the HFPS data were collected is mostly from high-income countries and showed mixed results (30-34). Furthermore, this period was relatively short, when few countries experienced substantial increases in vaccination rates such that individuals would have few in-person experiences from which to model behavior.

The main reasons reported in the HFPS regarding COVID-19 vaccine hesitancy, which mainly revolve around safety and to a lesser degree efficacy, are consistent with the few studies that report reasons for COVID-19 vaccine hesitancy in low- and middle-income countries and with the main reasons for vaccine hesitancy historically (14, 17, 28). Most directly comparable, Facebook survey results for the same month were available for 21 countries and showed that concerns about safety (worded as “concern about side effects” or “wait and see if it’s safe”) were the primary concern, followed in most countries by either concerns about efficacy or lack of trust in the government (supplemental figure S5). Our findings from Georgia are consistent with results published elsewhere, confirming that counterindication has been a significant barrier to vaccination in Eastern Europe and Central Asia (35).

For peer review only - http://bmjopen.bmj.com/site/about/guidelines.xhtml

For peer review only

Although concerns have been raised about supply constraints being the greatest barrier to COVID-19 vaccination scale-up in developing countries, our study did not find this to be a major concern for respondents. However, the framing of the question may have influenced this, as respondents may have interpreted the question whether they would get vaccinated “when the vaccine becomes available,” as indicating a time when current access constraints get resolved. Furthermore, there was a set of small countries in Latin America where over 10% of respondents cited supply constraints as their main concern, specifically “health center too far or hard to reach” or “I don't have time to go to get vaccinated.” This finding reinforces the importance citizens place on having easy and quick access. In addition, with the estimates that about 20 percent of the population was hesitant to get a COVID-19 vaccine, but with only 1 in 5 individuals in low-income countries vaccinated to date, there is still a large share of the population who would get vaccinated if vaccines were made available to them, and alleviating access constraints will remain a policy priority in such contexts.

4c. Limitations of the study

One potential limitation of this study is that it is based on phone survey data which exclude respondents who did not have access to a phone. This method of data collection was necessary to collect information quickly during the early months of the COVID-19 pandemic while respecting local movement restrictions and minimizing the risk of COVID-19 transmission. Given that having a phone may be non-random, this might have created a risk that the results are representative only of the population with access to a phone. However, in the countries in this sample, access to mobile phones was high, and in countries where the sample was based on a previous survey, sampling weights were used in the analysis to correct for the biases resulting from non-random access to phones. A recent analysis using the same phone survey samples from four African countries demonstrated that the weighting procedures successfully minimized the selection bias in the phone surveys for a wide range of indicators (36). Another limitation is that country surveys were collected at different times, and some surveys were collected before a vaccine was available in the country while others were collected after the vaccine was available. During data collection (and the pandemic more generally), influential events occurred, including adverse news about side effects of certain vaccines, which could have changed respondents’ intentions. Furthermore, there are multiple vaccine manufacturers and types, and the type of vaccine available or anticipated to become available in each country may influence levels of hesitancy.

Conclusions

The main findings of this paper are that on average across countries, the level of COVID-19 vaccine hesitancy is approximately 20 percent and remained unchanged in most countries between late 2020 and the first half of 2021. Our results suggest that COVID-19 vaccine hesitancy in developing countries, while less prevalent than previously thought, will be an important and enduring obstacle to recovery from the pandemic. The sizeable discrepancies with other studies of COVID-19 vaccine hesitancy suggest that measures of hesitancy depend greatly on the framing of the hesitancy questions and the nature of the sample. The estimates of COVID-19 vaccine hesitancy are higher than levels of hesitancy reported towards other vaccines, indicating the challenges in scaling up COVID-19 vaccination campaigns may be even greater than for other diseases.

This study provides a rich set of results which can aid policy makers in the design of vaccination campaigns in developing countries. Overall, knowing that hesitancy is less prevalent than previous estimates may bolster the case for expanding vaccination interventions. Specifically, knowing that people over the age of 65 years are the least likely to be hesitant provides an important rationale for scaling up vaccine rollout

For peer review only - http://bmjopen.bmj.com/site/about/guidelines.xhtml

in this population group, which is at highest risk of severe disease and mortality from COVID-19. In many countries, a sizeable share of the hesitant reported being “not sure” about getting vaccinating (rather than definitely not being willing), likely providing greater opportunity to influence vaccination decisions in these countries. In addition, the findings point to the fact that it will be important to design vaccination campaigns that address concerns about safety, especially about side-effects, and that effectively reach the most hesitant, including women, younger adults, and less educated adults. This will likely call for multi-pronged strategies, given the heterogeneity in information sources across population groups.

For peer review only

Contributors All authors had full access to all the data in the study and take responsibility for the integrity of the data and the accuracy of the data analysis. All authors were responsible for its conception and design. IE, AC and GD were responsible for the preparation and analysis of the data and JD, DN, AC and YK contributed to their interpretation. JD drafted the manuscript, and AC, DN and YK made critical revision of the manuscript for important intellectual content. The corresponding author attests that all listed authors meet authorship criteria and that no others meeting criteria have been omitted.

Competing interests None declared.

Funding. This paper was funded by the World Bank (Project AA-P177656-GPLG-BB) and the Bill and Melinda Gates Foundation through the Umbrella Facility for Poverty and Equity, with support from the Global Financing Facility for Women, Children, and Adolescents (GFF).

Ethics approval statement: This study involves human participants. Each phone survey was implemented by the respective national statistical office or, in a few cases, a private firm. Informed consent was received from all survey respondents in each country. The World Bank does not require institutional ethics approval for household surveys that are partly or fully financed by the World Bank, including these national phone surveys that inform our research.

Data availability statement Data for most countries are available in a public, open access repository available from the World Bank's Microdata Library, High Frequency Phone Survey Catalog (https://microdata. worldbank.org/index.php/catalog/hfps).

For peer review only

References

(1) Dong E, Du H, Gardner L. An interactive web-based dashboard to track COVID-19 in real time. Lancet Infect Dis 2020; 20(5): 533-4. https://coronavirus.jhu.edu/ accessed March 1, 2022.
(2) World Bank. 2022. Global Economic Prospects, January 2022. Washington, DC: World Bank. doi: 10.1596/978-1-4648-1758-8
(3) International Monetary Fund. 2021. World Economic Outlook Update. July 2021. Fault Lines Widen in the Global Recovery.
(4) Hoogeveen, JG and G. Lopez-Acevedo, eds. 2021. Distributional Impacts of COVID-19 in the Middle East and North Africa Region. MENA Development Report;. Washington, DC: World Bank. © World Bank. https://openknowledge.worldbank.org/handle/10986/36618 License: CC BY 3.0 IGO.
(5) Ritchie, H., E. Mathieu, L. Rodés-Guirao, et al.2020. - "Coronavirus Pandemic (COVID-19)". Published online at OurWorldInData.org. Retrieved from: 'https://ourworldindata.org/coronavirus' on March 8, 2022.
(6) MacDonald, NE, and SAGE Working Group on Vaccine Hesitancy. Vaccine hesitancy: Definition, Scope, and determinants. Vaccine. Volume 33, Issue 34, 2015, Pages 4161-4164 https://doi.org/10.1016/j.vaccine.2015.04.036
(7) Gramacho WG, Turgeon M. 2021. When politics collides with public health: COVID-19 vaccine country of origin and vaccination acceptance in Brazil. Vaccine. May 6;39(19):2608-2612. doi: 10.1016/j.vaccine.2021.03.080.
(8) Bucci EM, Berkhof J, Gillibert A, Gopalakrishna G, Calogero RA, Bouter LM, Andreev K, Naudet F, Vlassov V. Data discrepancies and substandard reporting of interim data of Sputnik V phase 3 trial. Lancet. 2021 May 22;397(10288):1881-1883.
(9) Wong, L.P., Alias, H., Danaee, M., Ahmed, J., Lachyan, A., Cai, C.Z., Lin, Y., Hu, Z., Tan, S.Y., Lu, Y. and Cai, G., 2021. COVID-19 vaccination intention and vaccine characteristics influencing vaccination acceptance: a global survey of 17 countries. Infectious diseases of poverty, 10(1), pp.1-14.
(10) Husby, A. and Køber, L., 2022. COVID-19 mRNA vaccination and myocarditis or pericarditis. The Lancet, 399(10342), pp.2168-2169..
(11) Robinson, E., Jones, A., Lesser, I. and Daly, M., 2021. International estimates of intended uptake and refusal of COVID-19 vaccines: A rapid systematic review and meta-analysis of large nationally representative samples. Vaccine.
(12) Lazarus, JV, Ratzan SC , and Palayew A et al. 2021. A global survey of potential acceptance of a COVID-19 vaccine. Brief Communication. Nature Medicine 27: 225–228. https://doi.org/10.1038/s41591-020-1124-9.
(13) de Figueiredo A, Larson HJ. Exploratory study of the global intent to accept COVID-19 vaccinations. Communications medicine. 2021 Sep 9;1(1):1-0.
(14) Facebook. 2021. COVID-19 Trends and Impact Survey.
(15)Gallup. 2021. Gallup World Poll. https://www.gallup.com/178667/gallup-world-poll-work.aspx.
(16) Kanyanda, S., Markhof, Y., Wollburg, P. and Zezza, A., 2021. Acceptance of COVID-19 vaccines in sub-Saharan Africa: Evidence from six national phone surveys. BMJ open, 11(12), p.e055159.

For peer review only - http://bmjopen.bmj.com/site/about/guidelines.xhtml

(17)Solís Arce, J.S., Warren, S.S., Meriggi, N.F. et al. COVID-19 vaccine acceptance and hesitancy in low- and middle-income countries. Nat Med **27,** 1385–1394 (2021). https://doi.org/10.1038/s41591-021-01454-y.

(18)Wouters, O.J., Shadlen, K.C., Salcher-Konrad, M., Pollard, A.J., Larson, H.J., Teerawattananon, Y. and Jit, M., 2021. Challenges in ensuring global access to COVID-19 vaccines: production, affordability, allocation, and deployment. The Lancet, 397(10278), pp.1023-1034.

(19)Sallam M. COVID-19 Vaccine Hesitancy Worldwide: A Concise Systematic Review of Vaccine Acceptance Rates. Vaccines (Basel). 2021 Feb 16;9(2):160. doi: 10.3390/vaccines9020160. PMID: 33669441; PMCID: PMC7920465.

(20)World Bank. 2020. High Frequency Mobile Phone Surveys of Households to Assess the Impacts of COVID-19: Overview (English). Washington, D.C.: World Bank Group. http://documents.worldbank.org/curated/en/703571588695361920/Overview

(21)Hale T, Angrist N, Goldszmidt R, et al. 2022. A global panel database of pandemic policies (Oxford COVID-19 government response Tracker). Nat Hum Behav 2021;5:529–38. Database accessed Feb 28, 2022.

(22)Inglehart, R, C. Haerpfer, A. Moreno et al. 2022. Would Values Survey: Round Seven – Country Pooled Datafile Version: https://www.worldvaluessurvey.org/WVSOnline.jsp. (accessed April 21, 2022) Madrid: JD Systems Institute.

(23)World Health Organization. 2022. Global Excess Deaths Associated with COVID-19 (Modelled Estimates). Downloaded on September 8, 2022 at https://www.who.int/data/sets/global-excess-deaths-associated-with-covid-19-modelled-estimates.

(24)Africa CDC. 2021. COVID-19 vaccine perceptions: a 15-country study, 2021. Available: https://africacdc.org/download/covid-19-vaccineperceptions-a-15-country-study/ [Accessed 18 May 2021].

(25)Anjorin, A.A., Odetokun, I.A., Abioye, A.I., Elnadi, H., Umoren, M.V., Damaris, B.F., Eyedo, J., Umar, H.I., Nyandwi, J.B., Abdalla, M.M. and Tijani, S.O., 2021. Will Africans take COVID-19 vaccination? PloS one, 16(12), p.e0260575.

(26)Faezi, N.A., Gholizadeh, P., Sanogo, M., Oumarou, A., Mohamed, M.N., Cissoko, Y., Sow, M.S., Keita, B.S., Baye, Y.A.M., Pagliano, P. and Akouda, P., 2021. Peoples' attitude toward COVID-19 vaccine, acceptance, and social trust among African and Middle East countries. Health Promotion Perspectives, 11(2), p.171.

(27)Bono, S.A., Faria de Moura Villela, E., Siau, C.S., Chen, W.S., Pengpid, S., Hasan, M.T., Sessou, P., Ditekemena, J.D., Amodan, B.O., Hosseinipour, M.C. and Dolo, H., 2021. Factors affecting COVID-19 vaccine acceptance: an international survey among low-and middle-income countries. Vaccines, 9(5), p.515.Gallup. 2019. Wellcome Global Monitor – First Wave Findings. https://wellcome.org/reports/wellcome-global-monitor/2018#downloads-4d1c.

(28)Cheikh, N., J. Spitz and D. Wilson. 2022. Increase Vaccine Targeting and Uptake: lessons learned from Global COVID-19 Vaccine Implementation. Considerations for World Bank Task Teams Managing COVID-19 Fast Track Facility Operations. Washington, D.C.: World Bank Group.

(29)Orangi S, Ojal J, Brand SP, et al. 2022. Epidemiological impact and cost-effectiveness analysis of COVID-19 vaccination in Kenya. BMJ Global Health **7:**e009430.

(30)To QG, Stanton R, Khalesi S, Williams SL, Alley SJ, Thwaite TL, Fenning AS, Vandelanotte C. Willingness to Vaccinate against COVID-19 Declines in Australia, Except in Lockdown Areas.

For peer review only - http://bmjopen.bmj.com/site/about/guidelines.xhtml

Vaccines (Basel). 2021 May 10;9(5):479. doi: 10.3390/vaccines9050479. PMID: 34068479; PMCID: PMC8150580.

(31) Lavoie K, Gosselin-Boucher V, Stojanovic J, Gupta S, Gagné M, Joyal-Desmarais K, Séguin K, Gorin SS, Ribeiro P, Voisard B, Vallis M, Corace K, Presseau J, Bacon S; iCARE Study Team. Understanding national trends in COVID-19 vaccine hesitancy in Canada: results from five sequential cross-sectional representative surveys spanning April 2020-March 2021. BMJ Open. 2022 Apr 5;12(4):e059411. doi: 10.1136/bmjopen-2021-059411. PMID: 35383087; PMCID: PMC8983402.

(32) Szilagyi PG, Thomas K, Shah MD, Vizueta N, Cui Y, Vangala S, Kapteyn A. National Trends in the US Public's Likelihood of Getting a COVID-19 Vaccine-April 1 to December 8, 2020. JAMA. 2020 Dec 29;325(4):396–8. doi: 10.1001/jama.2020.26419. Epub ahead of print. PMID: 33372943; PMCID: PMC7772743.

(33) Biasio LR, Bonaccorsi G, Lorini C, Mazzini D, Pecorelli S. Italian Adults' Likelihood of Getting COVID-19 Vaccine: A Second Online Survey. Vaccines (Basel). 2021 Mar 17;9(3):268. doi: 10.3390/vaccines9030268. PMID: 33802873; PMCID: PMC8002675.

(34) Sypsa V, Roussos S, Engeli V, Paraskevis D, Tsiodras S, Hatzakis A. Trends in COVID-19 Vaccination Intent, Determinants and Reasons for Vaccine Hesitancy: Results from Repeated Cross-Sectional Surveys in the Adult General Population of Greece during November 2020-June 2021. Vaccines (Basel). 2022 Mar 18;10(3):470. doi: 10.3390/vaccines10030470. PMID: 35335102; PMCID: PMC8950863.

(35) World Bank, UNIFEF and JICA 2021. Counterindication: now the key driver of COVID-19 vaccine hesitancy in Central Asia.

(36) Ambel AA, Mcgee KR, Tsegay AH. 2021. Reducing Bias in Phone Survey Samples: Effectiveness of Reweighting Techniques using Face-to-Face Surveys as Frames. World Bank: Washington, DC.

For peer review only - http://bmjopen.bmj.com/site/about/guidelines.xhtml

Figure Legends and notes

Figure 1

Title: Share of households that were hesitant to be vaccinated against COVID-19 in 53 countries

Notes: Weighted estimates, with weights scales such that 53 countries are given equal weights, except for results by education which include only results from the 33 country surveys with information on the educational attainment of the respondent.

Figure 2

Title: Share of households that were hesitant to be vaccinated against COVID-19, by country

Panel A Title: Share of households that were hesitant to be vaccinated against COVID-19 (answering either “no” or “not sure”)

Panel B Title: Strength of sentiment of COVID-19 vaccine hesitancy: share of households in 46 countries reporting “yes,” “not sure,” or “no” when asked if they will take the COVID-19 vaccination when available

Notes: Weighted estimates, with weights scales such that 53 countries are given equal weights. Black bars indicate confidence intervals. Point estimates are given in supplemental table S3. Panel B includes the 46 countries with three response options (yes, not sure, no).

Figure 3

Title: Changes in levels of vaccine hesitancy in 14 countries, October 2020 to April 2021

Note: The shaded area around the line represents the confidence bands.

Figure 4

Title: Self-reported reason for vaccine hesitancy, HFPS

Panel A: Countries with single answer response

Panel B: Countries with multiple responses

Notes: The X axis represents share of vaccine hesitant households. In some cases, the share is larger than 100% because multiple responses were allowed. For the construction of harmonized response categories, see supplemental table S2b.

For peer review only - http://bmjopen.bmj.com/site/about/guidelines.xhtml

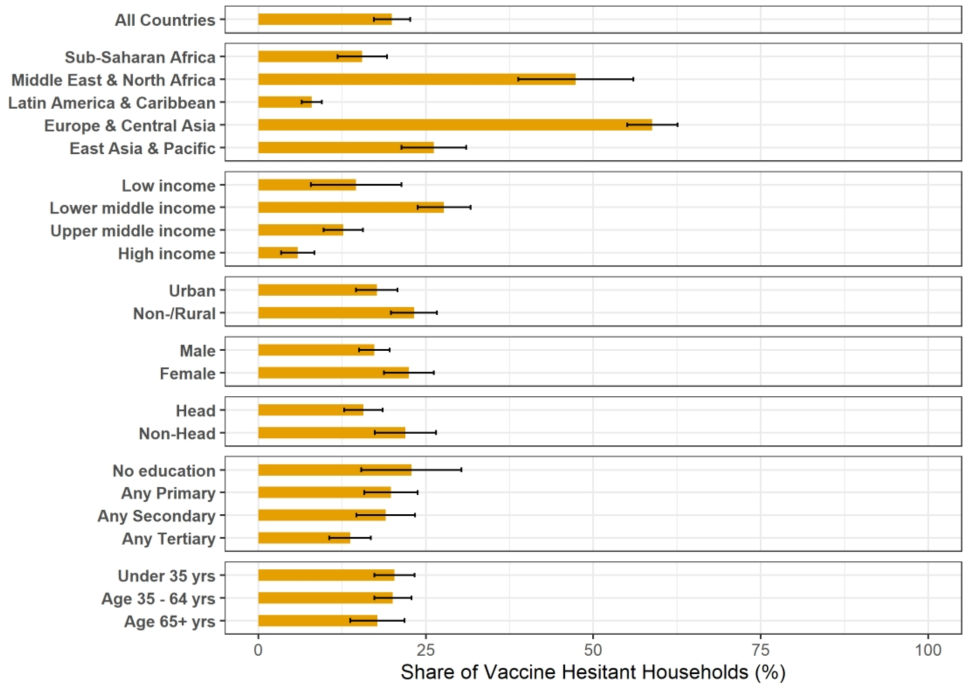

Figure 1: Share of households that were hesitant to be vaccinated against COVID-19 in 53 countries

490x342mm (118 x 118 DPI)

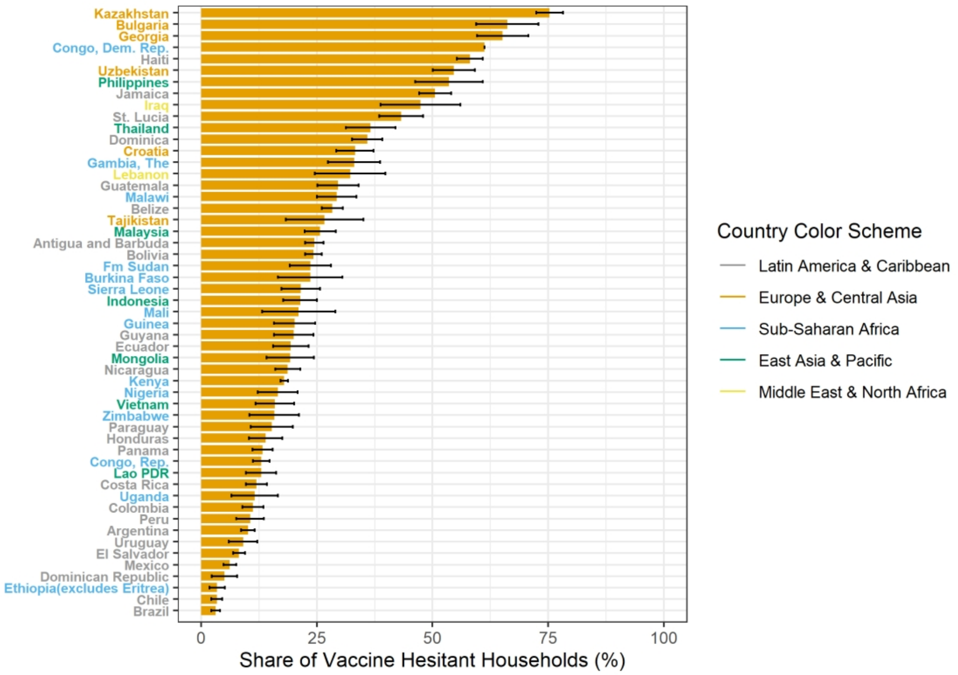

Figure 2: Share of households that were hesitant to be vaccinated against COVID-19, by countryPanel A: Share of households that were hesitant to be vaccinated against COVID-19 (answering either “no” or “not sure”)

490x336mm (118 x 118 DPI)

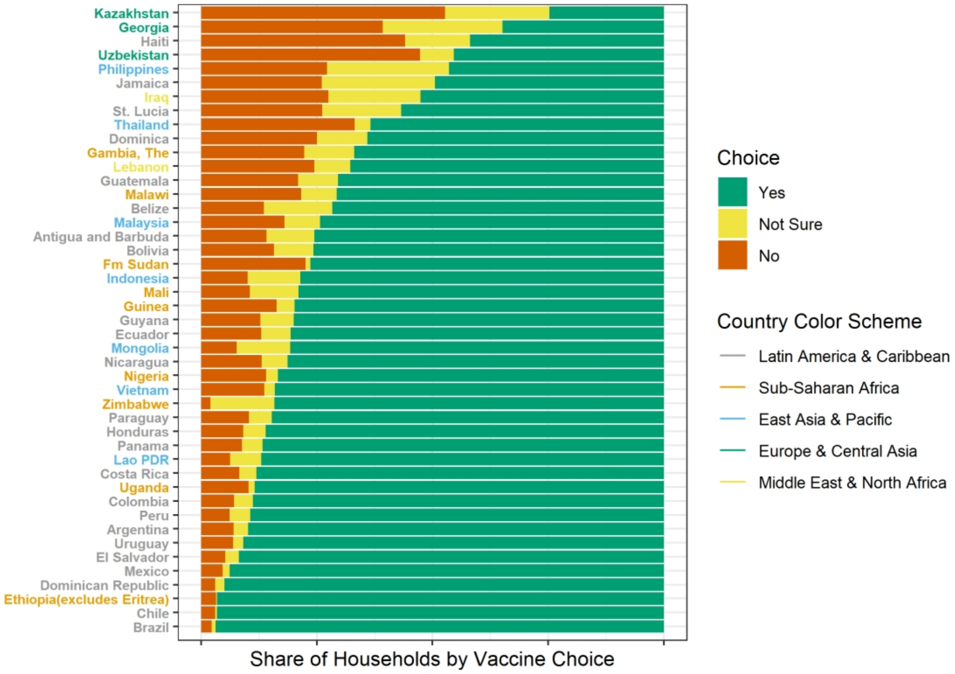

Panel B: Strength of sentiment of COVID-19 vaccine hesitancy: share of households in 46 countries reporting "yes," "not sure," or "no" when asked if they will take the COVID-19 vaccination when available

490x336mm (118 x 118 DPI)

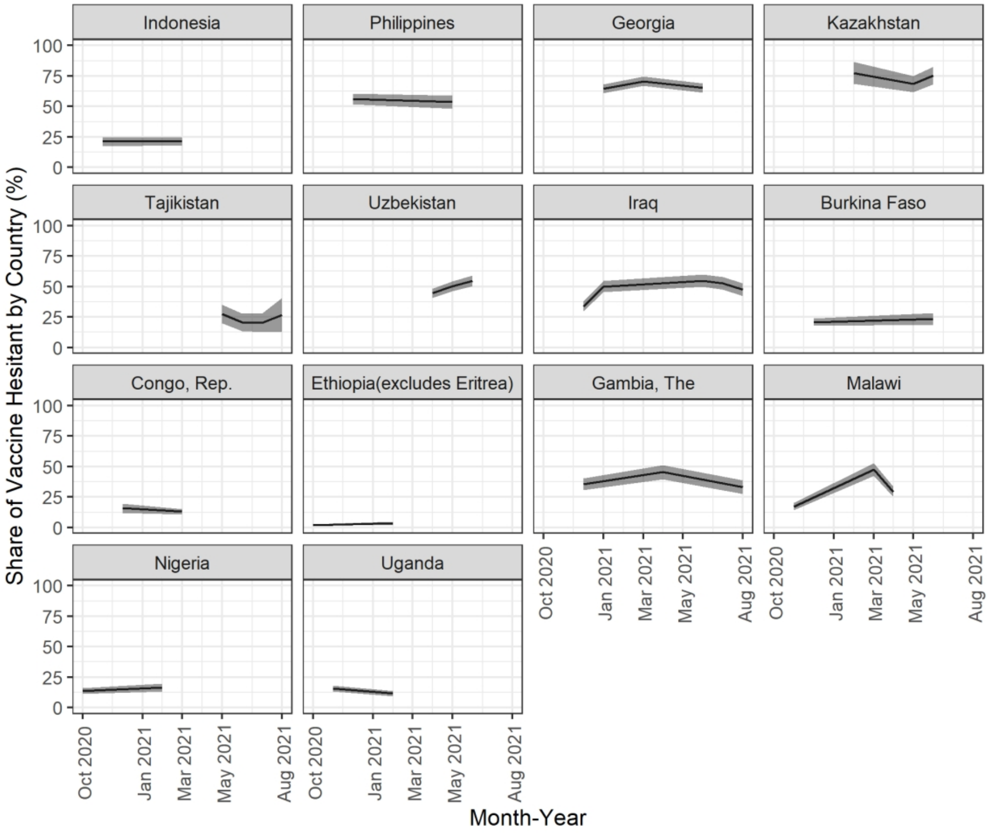

Figure 3: Changes in levels of vaccine hesitancy in 14 countries, October 2020 to April 2021

441x373mm (118 x 118 DPI)

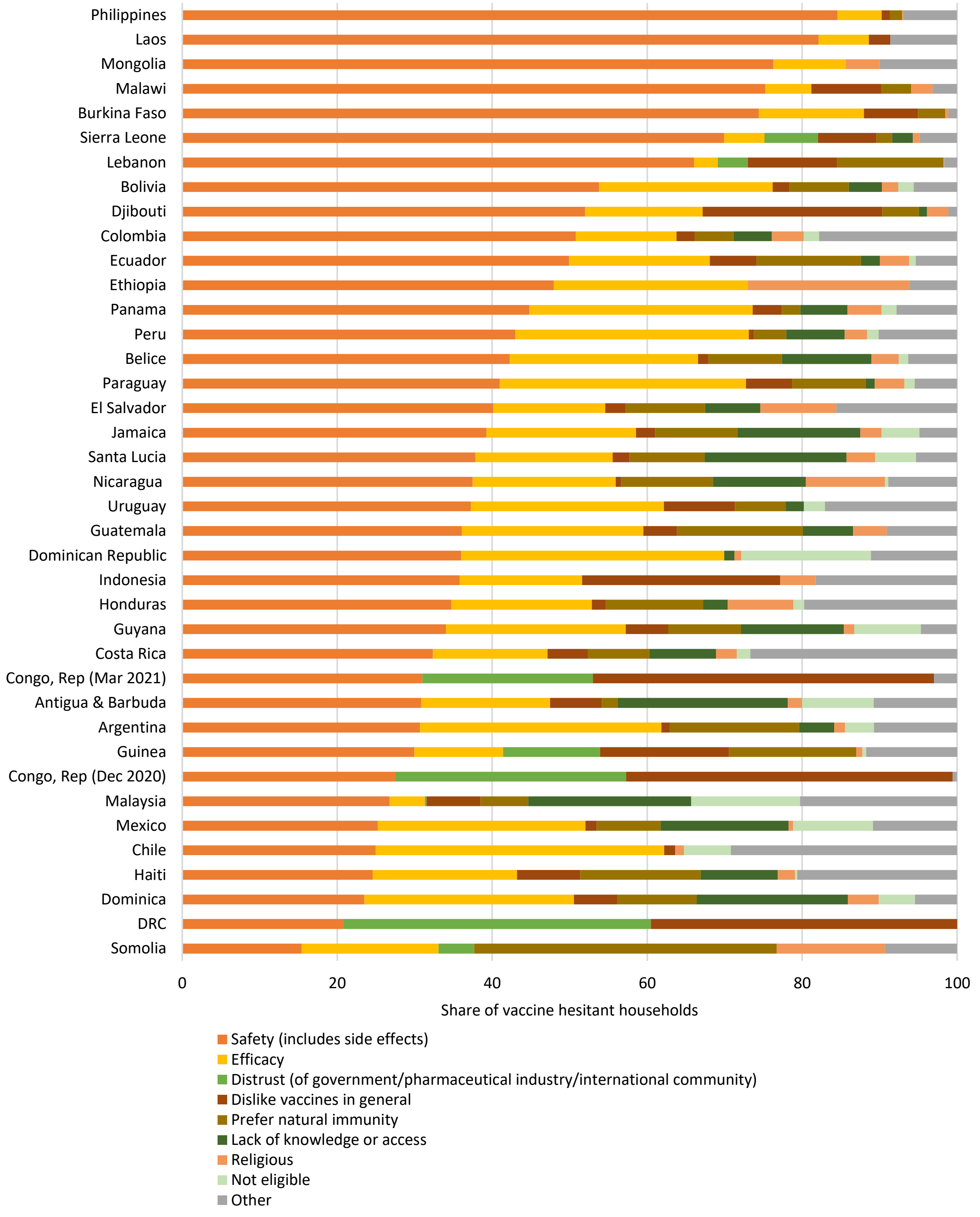
Philippines
Laos
Mongolia
Malawi
Burkina Faso
Sierra Leone
Lebanon
Bolivia
Djibouti
Colombia
Ecuador
Ethiopia
Panama
Peru
Belice
Paraguay
El Salvador
Jamaica
Santa Lucia
Nicaragua
Uruguay
Guatemala
Dominican Republic
Indonesia
Honduras
Guyana
Costa Rica
Congo, Rep (Mar 2021)
Antigua & Barbuda
Argentina
Guinea
Congo, Rep (Dec 2020)
Malaysia
Mexico
Chile
Haiti
Dominica
DRC
Somolia
0
20
40
60
80
100
Share of vaccine hesitant households
Safety (includes side effects)
Efficacy
Distrust (of government/pharmaceutical industry/international community)
Dislike vaccines in general
Prefer natural immunity
Lack of knowledge or access
Religious
Not eligible
Other

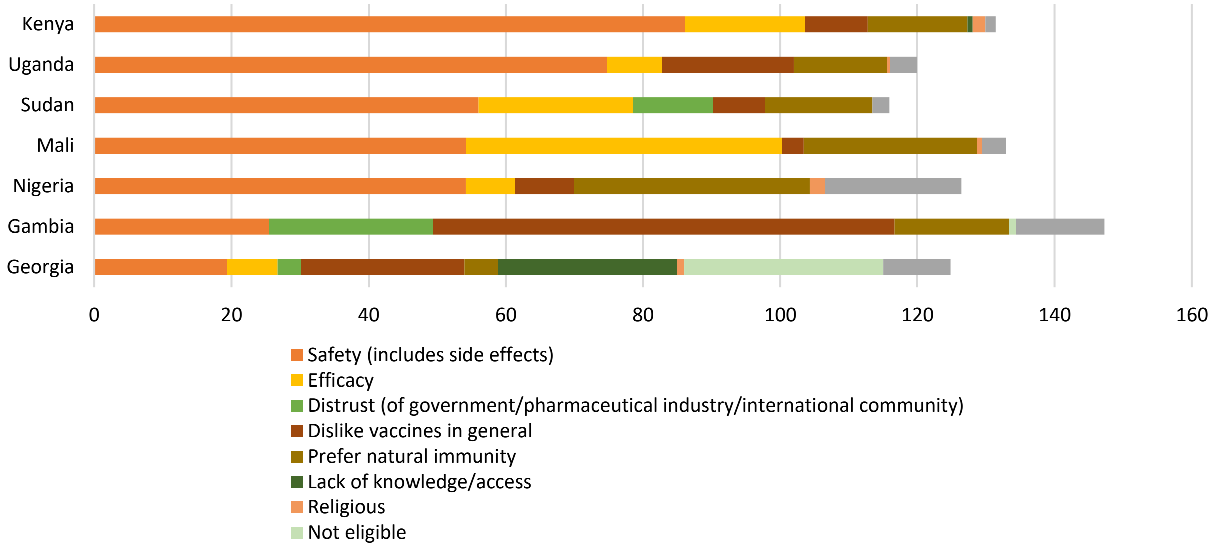

peer review only

Supplemental materials, COVID-19 Vaccine Hesitancy in 53 Countries

1. Supplements

Supplement 1: Detailed Description of Methods

2. Supplemental Tables

Supplemental Table S1: Description of 53 HFPS surveys

Supplemental Table S2a: Survey response options for COVID-19 vaccine hesitancy: distribution of countries by questionnaire type

Supplemental Table S2b: Survey response options for COVID-19 vaccine hesitancy: Harmonized answers by questionnaire type

Supplemental Table S3: Percent of population hesitant to take the COVID-19 vaccine, HFPS

Supplemental Table S4: Tukey's tests of significance of differences across groups in percent of population hesitant to take the COVID-19 vaccine

Supplemental Table S5a: Correlates of vaccine hesitancy (Logit model, marginal effects)

Supplemental Table S5b: Correlates of vaccine hesitancy (Ordinal logit regression, marginal effects)

Supplemental Table S6: Percent of population hesitant to take the COVID-19 vaccine, 14 countries with multiple rounds of survey results (95% confidence intervals in parentheses).

Supplemental Table S7: Comparison of results on vaccine hesitancy from the HFPS and other studies

3. Supplemental Figures

Supplemental Figure S1: Vaccination rates by income group

Supplemental Figure S2: Vaccine rollout rates by income group

Supplemental Figure S3: Comparison of share of population COVID-19 that is hesitant to take the COVID-19 vaccine, HFPS with other studies

Supplemental Figure S4: Comparison of COVID-19 Vaccine Hesitancy with pre-COVID sentiment about vaccines from 2019 Global Monitor, 45 countries

Supplemental Figure S5: Reason for "definitely not" (upper panel) or "probably not" (lower panel) taking the COVID-19 vaccine, Facebook data for month of HFPS)

4. References

For peer review only - http://bmjopen.bmj.com/site/about/guidelines.xhtml

For peer review only

Supplement 1: Detailed Description of Methods

Data

This analysis describes the levels of vaccine hesitancy and its reasons in 53 developing countries between October 2020 August 2021 using data from the World Bank's COVID-19 high-frequency phone surveys (HFPS) which were implemented to monitor the impact of COVID-19 on households around the world (World Bank 2020). Data are available for one survey round from 39 countries and two or more survey rounds for 14 countries. The countries in the pooled database represent five out of six regions defined by the World Bank: Latin America and the Caribbean (24 countries), Sub-Saharan Africa (14), East Asia and Pacific (7), Europe and Central Asia (6) and the Middle East and North Africa (2).[1] By income group, the sample includes 12 low-income countries, 13 lower-middle income countries, 23 upper middle-income countries and five high-income countries (Supplemental Table 1 provides a list of countries with survey month, sample size, region and country income group).

A global core questionnaire served as the basis for each country's survey but was then customized to fit the local context. As a result, the exact questions may vary across countries, but topics typically included knowledge and concerns about COVID-19, access to food, health care and education, employment and income loss, and safety nets and coping strategies. The survey data was then harmonized ex-post to enable cross-country comparability. The questionnaire was flexible and adapted over time to the pandemic context. The vaccine module, for example, was included more recently.

The sampling frame was drawn from pre-existing nationally representative household surveys in 19 countries, random digit dialing (RDD) in 29 countries, and a list of phone numbers typically obtained from mobile phone operators in 5 countries. The RDD design samples from all active mobile and landline phone numbers, such that RDD surveys are representative of the population aged 18 and over with an active phone number, conditional on response and survey completion. Sampling weights were then constructed to correct for selection bias due to the inability to contact households that did not participate in the survey either due to non-response or lack of access to a phone, with the goal of obtaining estimates that are as close to being nationally representative as possible. Information was collected from one respondent per household. In the case of countries where the sampling frame was derived from a previous survey, this was typically the household head. In other cases, such as when the sample utilized RDD, the respondent was more representative of individuals within the household. While the non-random selection of individuals in the former cases is not ideal, in an earlier analysis of labor market indicators derived from phone surveys, Kugler et al. (2021) find little evidence of differences in employment outcomes or trends over time being affected by the oversampling of household heads with respect to variables such as level of education, gender or location, the main source of bias being for age comparisons. Within the pooled data set, the household sampling weights are scaled such that countries are weighted equally, such that estimates of vaccine hesitancy reported in this study are averages of country averages.[2]

Outcome measures

The questions asked about vaccine hesitancy in the surveys varied depending on whether a vaccine was available in the country at the time of the survey. In surveys that took place before the vaccine rollout had begun in that country, the question asked was "*If an approved vaccine to prevent COVID-19 was to become*

[1] South Asia is not represented due to lack of available data. The Middle East and North Africa includes two countries—Iraq and Lebanon.

[2] Using population weights would have resulted in the results being driven by a small number of large countries.

For peer review only - http://bmjopen.bmj.com/site/about/guidelines.xhtml

available at no cost, would you agree to be vaccinated?" In surveys that took place after the vaccine rollout had begun, the wording of the question was either "*Are you planning to be vaccinated?*" or "*When a vaccine to prevent COVID-19 is available to you, are you planning to be vaccinated?"* In the sample, 47 countries offered three answer options (yes, not sure, and no), while the other six offered only two categories (yes and no). For this analysis, we combine the "no" and "not sure" answers to obtain the measure of vaccine hesitancy.

To obtain the respondent's reason for vaccine hesitancy, survey respondents who answered "no" or "not sure" were asked "*What is your (main) reason/concern for not wanting to be vaccinated / not being sure if you want to be vaccinated*?" The answer categories varied widely across surveys. To make these more comparable across countries, answers were remapped into the following nine most common categories (see Supplemental Tables 2a and 2b for the original answer options in each survey and a mapping of how they were harmonized):

1. Safety, which includes concerns about side effects
2. Efficacy
3. Distrust of government, pharmaceutical industry, international community
4. Dislike of vaccines in general
5. Preference for natural immunity, which included perceiving self as low risk and already having had COVID-19 infection
6. Lack of knowledge or access, which included "I do not have enough information about the vaccine," "too hard to get," "health facility to far" and "I don't have the time"
7. Religious reasons
8. Not eligible, which included "counter-indication," "recent medical discharge, "have an underlying health condition and believe taking the vaccine will make it worse"
9. Other

The surveys also differed across countries in terms of whether a single concern or multiple concerns were collected. To account for the difference in the number of response options, the results are presented separately.

Contextual data

Country-level contextual data were drawn from other sources. New COVID-19 cases per million, measured as a 7-day rolling average prior to the midpoint of the month household survey data was collected in each country, were obtained from Dong and colleagues (2022), and the Oxford Stringency Index was drawn from Hale et al. 2022. The latter is an index, ranging from 0–100, that indicates the degree to which various restrictions were put in place by governments to control the pandemic (for example, school closures and shelter-in-place requirements). Confidence in the press, the government, and the WHO was drawn from the World Values Survey Wave 7, fielded in 2017-2020, and aggregated up to the country level (Inglehart 2022). Excess deaths due to COVID-19 draw on WHO data (WHO 2022). Country geographic region and income group are based on the World Bank classifications.

Analytical strategy

Data from 53 countries was pooled into a single data set. First, we report point estimates and 95% confidence intervals for each country based on standard errors clustered at the state or province level within each country. We also report the simple average across all countries and stratified by: (i) World Bank region, (ii) World Bank country income group, (iii) urban vs. rural residence, (iv) gender of the respondent, (v) whether the respondent is head of household, (vi) age of the respondent (under 35 years,

For peer review only - http://bmjopen.bmj.com/site/about/guidelines.xhtml

35-64 years, and 65 years and older, and (vii) educational attainment of the respondent (some primary school, some secondary school, some tertiary). Tukey's test of multiple comparisons is used to test the significance of differences in rates of vaccine hesitancy across the above-mentioned groups. Second, we use multivariate regression analysis to assess the relative association of vaccine hesitancy and the correlates and contextual variables. Trends over time in levels of vaccine hesitancy are examined in 14 countries with more than one wave of results available. Finally, the analysis describes the reasons for vaccine hesitancy.

For peer review only

For peer review only - http://bmjopen.bmj.com/site/about/guidelines.xhtml

Supplemental Table S1: Description of 53 HFPS surveys

Country	Survey Months	Total Number of households	Sample frame
**Antigua and Barbuda**	06/2021	790	RDD
**Argentina**	06/2021	1216	RDD
**Belize**	06/2021	816	RDD
**Bolivia**	05/2021	1272	RDD
**Brazil**	08/2021	2166	RDD
**Bulgaria**	07/2021	1000	RDD
**Burkina Faso**	12/2020	1944	Previous survey
**Chile**	06/2021	1212	RDD
**Colombia**	06/2021	1221	RDD
**Congo, Dem. Rep.**	12/2020	986	Previous Survey
**Congo, Rep.**	12/2020, 03/2021	578, 1495	Previous Survey
**Costa Rica**	06/2021	802	RDD
**Croatia**	03/2021	1217	Non-survey list
**Dominica**	06/2021	861	RDD
**Dominican Republic**	06/2021	1205	RDD
**Ecuador**	05/2021	1352	RDD
**El Salvador**	06/2021	816	RDD
**Ethiopia**	10/2020, 02/2021	2704, 2178	Previous Survey
**The Gambia**	12/2020, 04/2021, 08/2021	1334, 1287, 1059	Previous Survey
**Georgia**	01/2021, 03/2021, 06/2021	2033, 2100, 1936	RDD
**Guinea**	11/2020	1334	Previous Survey
**Guatemala**	06/2021	1206	RDD
**Guyana**	06/2021	785	RDD
**Haiti**	07/2021	2813	Non-Survey list
**Honduras**	07/2021	1021	RDD
**Indonesia**	11/2020, 03/2021	3953, 3555	Previous Survey
**Iraq**	12/2020, 01/2021,06/2021, 07/2021, 08/2021	1614,1651,1378, 1297,1141	Non-Survey list
**Jamaica**	06/2021	828	RDD
**Kazakhstan**	02/2021, 05/2021, 06/2021	917, 1732, 1610	Previous Survey
**Kenya**	03/2021	6730	Previous Survey

For peer review only - http://bmjopen.bmj.com/site/about/guidelines.xhtml

Country	Survey Months	Total Number of households	Sample frame
**Lao PDR**	03/2021	2153	RDD
**Lebanon**	03/2021	5113	RDD
**Mali**	01/2021	1884	Previous Survey
**Malawi**	11/2020, 03/2021, 04/2021	1589, 1549,1338	Previous Survey
**Malaysia**	06/2021	2210	RDD
**Mexico**	06/2021	2624	RDD
**Mongolia**	12/2020	1147	Previous Survey
**Nicaragua**	06/2021	833	RDD
**Nigeria**	02/2021, 10/2020	1699, 1762	Previous Survey
**Panama**	06/2021	815	RDD
**Paraguay**	06/2021	1076	RDD
**Peru**	06/2021	1210	RDD
**Philippines**	12/2020, 05/2021	1805, 2122	Non-survey list
**Saint Lucia**	06/2021	835	RDD
**Sierra Leone**	11/2020	1198	Previous Survey
**Sudan**	03/2021	2545	Non-Survey list
**Tajikistan**	05-08/2021	232	Previous Survey
**Thailand**	05/2021	1786	RDD
**Uganda**	11/2020, 02/2021	2135, 2121	Previous Survey
**Uruguay**	06/2021	816	RDD
**Uzbekistan**	04-06/2021	1496, 1356, 1300	Previous Survey
**Vietnam**	01/2021	3940	Previous Survey
**Zimbabwe**	12/2020	1227	Previous Survey

For peer review only

Supplemental Table S2a: Survey response options for COVID-19 vaccine hesitancy: distribution of countries by questionnaire type

**Questionnaire type 1**	**Questionnaire type 2**	**Questionnaire type 3**	**Questionnaire type 4**	**Questionnaire type 5**	**Questionnaire type 6**	**Questionnaire type 7**	**Questionnaire type 8**	**Questionnaire type 9**	**Questionnaire type 10**	**Questionnaire LAC**	**Questionnaire type 11**	**Questionnaire type 12**
Kenya	*Philippines*	*Lebanon*	*Congo, Democratic Republic*	*Guinea*	*Malaysia*	*Mali*	*Gambia, The*	*Lao PDR*	*Cambodia*	*Argentina*	*Kazakhstan*	*Croatia*
Thailand	*Indonesia*		*Republic of Congo*	*Sudan*		*Burkina Faso*		*Mongolia*		*Antigua and Barbuda*	*Kyrgyzstan*	
	Djibouti			*Sierra Leone*		*Uganda*				*Belize*	*Uzbekistan*	
						Ethiopia				*Bolivia*		
						Malawi				*Brazil*		
						Nigeria				*Chile*		
										Colombia		
										Costa Rica		
										Dominica		
										Dominican Republic		
										Ecuador		
										Guatemala		
										Guyana		
										Honduras		
										Haiti		
										Jamaica		
										Mexico		
										Nicaragua		
										Panama		
										Peru		
										Paraguay		
										El Salvador		
										Uruguay		

For peer review only - http://bmjopen.bmj.com/site/about/guidelines.xhtml

For peer review only

Supplemental Table S2b: Survey response options for COVID-19 vaccine hesitancy: Harmonized answers by questionnaire type

Answer categories	Quest type 1	Quest type 2	Quest type 3	Quest type 4	Quest type 5	Quest type 6	Quest type 7	Quest type 8	Quest type 9	Quest type 10	Quest LAC	Quest type 11	Quest type 12
**1. Efficacy**	I don't think it will work	I don't think COVID-19 vaccines effective/wo rk	I don't think coronavirus vaccines are effective/wo rk		I don't think vaccines work	I don't think it will work	I don't think it will work	I do not think the vaccine would work against COVID	I don't think COVID-19 vaccines effective/wo rk		I do not think they are effective, that they work		It may not work
						I don't think the vaccines available in my country are effective							
**2 Safety (includes side effects)**	I don't think it will be safe	I am worried about the safety of the vaccine		Safety of the vaccine	I am worried about the vaccine safety		I don't think it will be safe	I fear the unforeseen future negative side effects from the COVID-19 vaccine	I am worried about the safety of the vaccine	I don't think it is safe		The risk of vaccinating is higher than the benefits	Health risks
	I am worried about the side effects	I am worried about the side effects	I am worried about the side effects of the vaccine			I am worried about the side effects	I'm worried about side effects	I have heard the vaccine has negative side effects	I am worried about the side effects		I don't think it's safe, because of the side effects		
**3. Perceive self as low risk**	I am not enough at risk of contracting COVID-19	I am strong, I never got any disease in the past	I am not enough at risk of contracting coronavirus			I am not enough at risk of contracting COVID-19	I am not at enough risk of contracting covid-19	I believe I do not need to be vaccinated			I am not at risk of getting covid-19		I am not worried about COVID-19
**4.Dislike vaccines in general**	I am against vaccines in general	I am against vaccines in general	I am against vaccines in general	General distrust of vaccines	I don't trust the vaccines in general	In general, I don't trust vaccines	I am against the vaccine in general		I am against vaccines in general		I am against vaccines in general	Negative past experiences with vaccines	I am against vaccines in general
**5 Religious**	It is against my religion	Religious reasons	Religious reasons		I have religious reasons/con cerns	It is against my religion.	It's against my religion		Religious reasons		Religious reasons	Personal or religious beliefs	
		I am concerned about its											

For peer review only - http://bmjopen.bmj.com/site/about/guidelines.xhtml

Answer categories	Quest type 1	Quest type 2	Quest type 3	Quest type 4	Quest type 5	Quest type 6	Quest type 7	Quest type 8	Quest type 9	Quest type 10	Quest LAC	Quest type 11	Quest type 12
		halal certification											
**6 Concerned about getting covid at facility**	I'm worried to get infected with COVID-19 at the health facility	I am worried of getting infected with COVID-19 at the health facility	I am worried of getting infected with coronavirus at the health facility			I am worried to get infected with COVID-19 at the health facility.			I am worried to get infected with COVID-19 at the health facility		I am concerned of being infected with covid-19 at the health center		
**7 Supply /access barrier**	Health facility is too far or too hard to get to		Health facility too far or too hard to get to		It does not suit me (will be given too far or difficult to find)	Health facility too far or too hard to get to		There is shortage of vaccines in the country		I don't know how to access the vaccine	Health center too far or hard to reach		
	I don't have time to get vaccinated/ It will take too long to get vaccinated					It will take too long to get vaccinated/ I don't have time to get vaccinated		There is no vaccination center near my place		I am not the priority group to get the vaccine	I don't have time to go to get vaccinated		
**8 Prefer natural immunity (includes already had COVID-19)**			I already had coronavirus		I believe that the remedies natural or traditional	I already had COVID-19		I prefer to build immunity against COVID-19 naturally by having the disease			I already had covid-19 / I no longer need it		I already had COVID-19 and do''t need a vaccine
					It is better to leave nature take its course; the COVID symptoms are mostly light								

For peer review only

Answer categories	Quest type 1	Quest type 2	Quest type 3	Quest type 4	Quest type 5	Quest type 6	Quest type 7	Quest type 8	Quest type 9	Quest type 10	Quest LAC	Quest type 11	Quest type 12
**9 Distrust (of government/pharmaceutical industry/international community)**				Be wary of international community	I do''t trust the government/ to the organization who gives the vaccine			I do not trust pharmaceutical companies				Lack of trust to producers of vaccines	I do''t trust the pharmaceutical industry
								I do not trust the government				Lack of trust to heath system or healthcare providers	I do''t trust the government
								I do not trust the COVID-19 vaccines					
								I heard the vaccine is meant to control population growth					
								I do not believe in COVID-19					
**10 Not eligible / conterindication**								I have underlying health conditions and I believe taking the vaccine will make it worse		I am not eligible to get the vaccine			Counter-indication or prolonged medical discharge
**11 Lack of knowledge about COVID 19 Vaccine**								I do not have enough information about the vaccine					

For peer review only - http://bmjopen.bmj.com/site/about/guidelines.xhtml

Answer categories	Quest type 1	Quest type 2	Quest type 3	Quest type 4	Quest type 5	Quest type 6	Quest type 7	Quest type 8	Quest type 9	Quest type 10	Quest LAC	Quest type 11	Quest type 12
								I did not know that a vaccine exists against COVID-19					
**12 Other**	Other (specify)	Other (specify)	Some other reason	other	Others	Other (Specify)	other, specify)	Everyday stresses are overwhelming to think about getting vaccinated	Others (specify)	Other, specify	Other (specify)	Other	
		I will wait till more people are vaccinated											
		No one in my neighborhood got it				I am not sure I will get the vaccine I want							

For peer review only

Supplemental Table S3: Percent of population hesitant to take the COVID-19 vaccine, HFPS

Average across countries	Percent (%) hesitant to take the COVID-19 vaccine	Confidence Intervals
All countries	20.0	[17.24 , 22.67]
East Asia & Pacific	26.2	[21.38 , 31.02]
Europe & Central Asia	58.8	[55.04 , 62.56]
Latin America & Caribbean	8.0	[6.45 , 9.47]
Middle East & North Africa	47.4	[38.80 , 55.96]
Sub-Saharan Africa	15.5	[11.81 , 19.19]
Low income countries	14.6	[7.82 , 21.39]
Lower middle income countries	27.7	[23.78 , 31.65]
Upper middle income countries	12.7	[9.77 , 15.61]
High income countries	5.9	[3.40 , 8.36]
Urban	17.6	[14.60 , 20.75]
Rural	23.2	[19.83 , 26.64]
Male	17.3	[15.02 , 19.63]
Female	22.5	[18.77 , 26.21]
Head of household	10.8	[12.82 , 18.53]
Non-Head of household	9.4	[17.37 , 26.54]
No education	22.8	[15.36 , 30.31]
Any Primary	19.8	[15.80 , 23.78]
Any Secondary	19.0	[14.64 , 23.42]
Any Tertiary	13.7	[10.56 , 16.83]
Age 34 and younger	20.3	[17.29 , 23.34]
Age 35— 64	20.1	[17.34 , 22.84]
Ages 65 and older	17.8	[13.70 , 21.80]
Antigua and Barbuda	24.5	[22.51 , 26.49]
Argentina	10.1	[8.66 , 11.53]
Belize	28.4	[26.10 , 30.64]
Bolivia	24.3	[22.47 , 26.04]
Brazil	3.1	[2.15 , 4.04]
Bulgaria	66.2	[59.48 , 72.90]
Burkina Faso	23.6	[16.60 , 30.53]
Chile	3.4	[2.22 , 4.57]
Colombia	11.2	[8.91 , 13.51]
Congo, Dem. Rep.	61.2	[61.23 , 61.23]
Congo, Rep.	13.0	[11.20 , 14.79]

For peer review only - http://bmjopen.bmj.com/site/about/guidelines.xhtml

Average across countries	Percent (%) hesitant to take the COVID-19 vaccine	Confidence Intervals
Costa Rica	11.9	[9.66 , 14.22]
Croatia	33.2	[29.21 , 37.25]
Dominica	35.9	[32.63 , 39.14]
Dominican Republic	5.0	[2.25 , 7.81]
Ecuador	19.4	[15.54 , 23.21]
El Salvador	8.2	[6.91 , 9.44]
Ethiopia (excludes Eritrea)	3.5	[1.81 , 5.09]
Fm Sudan	23.7	[19.19 , 28.11]
Gambia, The	33.1	[27.44 , 38.70]
Georgia	65.2	[59.62 , 70.72]
Guatemala	29.6	[25.09 , 34.05]
Guinea	20.2	[15.74 , 24.61]
Guyana	20.0	[15.75 , 24.23]
Haiti	58.1	[55.31 , 60.86]
Honduras	13.9	[10.31 , 17.51]
Indonesia	21.4	[17.71 , 25.07]
Iraq	47.4	[38.81 , 55.99]
Jamaica	50.6	[47.11 , 54.03]
Kazakhstan	75.3	[72.39 , 78.18]
Kenya	17.9	[17.11 , 18.74]
Lao PDR	13.0	[9.70 , 16.25]
Lebanon	32.2	[24.56 , 39.84]
Malawi	29.3	[25.03 , 33.54]
Malaysia	25.7	[22.37 , 29.10]
Mali	21.1	[13.14 , 29.03]
Mexico	6.2	[4.85 , 7.55]
Mongolia	19.3	[14.13 , 24.38]
Nicaragua	18.7	[16.04 , 21.40]
Nigeria	16.6	[12.27 , 20.84]
Panama	13.3	[11.12 , 15.49]
Paraguay	15.3	[10.71 , 19.79]
Peru	10.6	[7.55 , 13.59]
Philippines	53.6	[46.28 , 60.83]
Sierra Leone	21.5	[17.31 , 25.66]
St. Lucia	43.2	[38.46 , 47.93]
Tajikistan	26.7	[18.32 , 35.03]
Thailand	36.6	[31.25 , 42.00]
Uganda	11.6	[6.51 , 16.61]
Uruguay	9.1	[5.94 , 12.17]
Uzbekistan	54.6	[50.02 , 59.18]
Vietnam	15.9	[11.72 , 20.14]
Zimbabwe	15.8	[10.42 , 21.14]

For peer review only
For peer review only - http://bmjopen.bmj.com/site/about/guidelines.xhtml

Supplemental Table S4: Tukey's tests of significance of differences across groups in percent of population hesitant to take the COVID-19 vaccine

Group	Comparison	Difference	Std. Error	t-score	p-value	Signi-ficance
Gender	Female vs Male	0.052	0.022	2.316	0.021	**
Household Status	Head vs Non-Head	-0.063	0.026	-2.398	0.016	**
Age	Ages 65 and older vs Age 35 - 64	-0.023	0.025	-0.937	0.349	
	Ages 65 and older vs Age 34 and younger	-0.026	0.026	-0.994	0.320	
	Age 35 - 64 vs Age 34 and younger	-0.002	0.021	-0.107	0.543	
Education	Any Primary vs Any Secondary	0.008	0.030	0.250	0.599	
	Any Primary vs Any Tertiary	0.061	0.026	2.355	0.019	**
	Any Primary vs No Education	-0.030	0.043	-0.705	0.481	
	Any Secondary vs Any Tertiary	0.053	0.028	1.941	0.052	*
	Any Secondary vs No Education	-0.038	0.044	-0.860	0.390	
	Any Tertiary vs No Education	-0.091	0.041	-2.211	0.027	**
Sector	Rural vs Urban	0.056	0.023	2.375	0.018	**
Region	EAP vs ECA	-0.326	0.031	-10.450	0.000	***
	EAP vs LAC	0.182	0.026	7.077	0.000	***
	EAP vs MENA	-0.212	0.050	-4.219	0.000	***
	EAP vs SSA	0.107	0.031	3.454	0.001	***
	ECA vs LAC	0.508	0.021	24.579	0.000	***
	ECA vs MENA	0.114	0.048	2.390	0.017	**
	ECA vs SSA	0.433	0.027	16.103	0.000	***
	LAC vs MENA	-0.394	0.044	-8.870	0.000	***
	LAC vs SSA	-0.075	0.020	-3.705	0.000	***
	MENA vs SSA	0.319	0.048	6.691	0.000	***

For peer review only - http://bmjopen.bmj.com/site/about/guidelines.xhtml

Income Group	HIC vs LIC	-0.087	0.037	-2.368	0.018	**
	HIC vs LMIC	-0.218	0.024	-9.202	0.000	***
	HIC vs UMIC	-0.068	0.020	-3.481	0.001	***
	LIC vs LMIC	-0.131	0.037	-3.559	0.000	***
	LIC vs UMIC	0.019	0.024	0.808	0.419	
	LMIC vs UMIC	0.150	0.025	6.010	0.000	***

Significance levels: *** for p-values <= 0.01, ** for 0.01 < p-values <= 0.05, * for 0.05 < p-values <= 0.1

For peer review only

For peer review only - http://bmjopen.bmj.com/site/about/guidelines.xhtml

Supplemental Table S5a: Correlates of vaccine hesitancy (Logit model, marginal effects)

Dep. Var: Hesitancy	(1)	(2)	(3)	(4)	(5)	(6)
Male	-0.027*	-0.028*	-0.027*	-0.027*	-0.033**	-0.040***
	(0.013)	(0.013)	(0.013)	(0.012)	(0.012)	(0.011)
Head of HH	0.013	0.009	0.021	-0.001	0.014	0.007
	(0.014)	(0.014)	(0.013)	(0.016)	(0.015)	(0.012)
Education of respondent (ref –						
No education	0.130***	0.131***	0.122***	0.142***	0.133**	0.153***
	(0.036)	(0.037)	(0.033)	(0.040)	(0.044)	(0.040)
Any primary	0.068***	0.069**	0.067***	0.073***	0.059**	0.079***
	(0.020)	(0.021)	(0.020)	(0.020)	(0.022)	(0.023)
Any secondary	0.069***	0.070***	0.068***	0.070***	0.064***	0.067***
	(0.011)	(0.011)	(0.011)	(0.011)	(0.011)	(0.012)
Age group (ref. -- 34 and younger)						
Working age (35-64)	-0.047***	-0.048***	-0.042**	-0.050***	-0.046**	-0.049***
	(0.014)	(0.013)	(0.015)	(0.013)	(0.015)	(0.012)
Retirement age (65+)	-0.103***	-0.102***	-0.096***	-0.101***	-0.103***	-0.102***
	(0.024)	(0.025)	(0.024)	(0.023)	(0.025)	(0.024)
Rural area	0.016	0.014	0.015	0.008	0.012	0.011
	(0.019)	(0.018)	(0.017)	(0.015)	(0.015)	(0.011)
Region (ref – LAC)						
EAP	0.189**	0.192*	0.222**	0.095	0.264*	0.188
	(0.067)	(0.078)	(0.077)	(0.069)	(0.130)	(0.130)
ECA	0.313***	0.315***	0.295***	0.328***	0.313***	0.295***
	(0.055)	(0.057)	(0.056)	(0.059)	(0.040)	(0.062)
MNA	0.208***	0.212***	0.245***	0.216***	0.282***	0.369***
	(0.037)	(0.040)	(0.062)	(0.032)	(0.059)	(0.051)
SSA	0.074	0.081	0.070	-0.022	0.047	0.022
	(0.075)	(0.077)	(0.070)	(0.105)	(0.083)	(0.104)
Income group (ref. – LIC)						
LMIC	-0.068	-0.065	-0.045	-0.088	-0.054	-0.071
	(0.122)	(0.119)	(0.112)	(0.123)	(0.097)	(0.069)
UMIC	-0.047	-0.038	-0.066	-0.095	-0.035	-0.137
	(0.149)	(0.148)	(0.130)	(0.150)	(0.116)	(0.087)
HIC	-0.173	-0.164	-0.190	-0.223	-0.202	-0.309***
	(0.163)	(0.160)	(0.143)	(0.164)	(0.130)	(0.090)
Survey month (ref -- Nov 2020 - Jan						
March - May 2021	-0.069	-0.060	-0.094	-0.056	-0.095	-0.083
	(0.095)	(0.108)	(0.103)	(0.098)	(0.104)	(0.095)
June - August 2021	-0.029	-0.025	-0.024	-0.052	-0.057	-0.055
	(0.088)	(0.098)	(0.100)	(0.087)	(0.092)	(0.075)
New COVID-19 cases per million, terciles (ref. – bottom tercile)						
Cases (middle tercile)		-0.017				0.026
		(0.067)				(0.060)
Cases (top tercile)		-0.019				-0.016
		(0.057)				(0.047)
Oxford stringency index terciles (ref. – bottom tercile)						
Stringency (middle tercile)			0.065			0.134
			(0.072)			(0.070)
Stringency (top tercile)			-0.008			0.027
			(0.076)			(0.068)
WHO excess deaths due to COVID-19 terciles (ref.- bottom tercile)						
Excess deaths (middle tercile)				-0.117*		-0.109
				(0.052)		(0.072)

For peer review only - http://bmjopen.bmj.com/site/about/guidelines.xhtml

Dep. Var: Hesitancy	(1)	(2)	(3)	(4)	(5)	(6)
Excess deaths (top tercile)				-0.069		0.040
				(0.072)		(0.108)
Confidence in government index tercile (ref.- top tercile)						
Confidence in government					0.048	0.070
					(0.053)	(0.057)
Confidence in government					0.024	0.053
					(0.097)	(0.103)
Pseudo R-squared	0.0745	0.0748	0.0794	0.0813	0.0869	0.1067
N	65088	65088	65088	65088	65088	65088

Notes: Weighted logit regressions. Marginal effects reported. Standard errors clustered at country level. *, **, *** indicates significance at the 95%, 99%, and 99.9% level.

For peer review only

For peer review only - http://bmjopen.bmj.com/site/about/guidelines.xhtml

Supplemental Table S5b: Correlates of vaccine hesitancy (Ordinal logit regression, marginal effects)

Dep. Var.:	Would you get the vaccine?		
	No	Not sure	Yes
Male	-0.025**	-0.007**	0.036**
	(0.01)	(0.002)	(0.011)
Head of HH	0.011	0.001	-0.006
	(0.012)	(0.002)	(0.011)
Education of respondent (ref – Tertiary)			
No education	0.119***	0.022**	-0.156***
	-0.028	(0.007)	(0.038)
Any primary	0.057**	0.013**	-0.080***
	-0.018	(0.004)	(0.022)
Any secondary	0.058***	0.012***	-0.068***
	(0.009)	(0.003)	(0.010)
Age group (ref. -- 34 and younger)			
Working age (35-64)	-0.040***	-0.009***	0.049***
	(0.010)	(0.002)	(0.012)
Retirement age (65+)	-0.079***	-0.018***	0.097***
	(0.020)	(0.005)	(0.025)
Rural area	0.010	0.002	-0.012
	(0.009)	(0.002)	(0.011)
Region (ref – LAC)			
EAP	0.149	0.034	-0.183
	(0.093)	(0.021)	(0.113)
ECA	0.238***	0.055***	-0.293***
	(0.044)	(0.016)	(0.058)
MNA	0.288***	0.066***	-0.354***
	(0.042)	(0.011)	(0.049)
SSA	0.015	0.004	-0.019
	(0.082)	(0.019)	(0.101)
Income group (ref. – LIC)			
LMIC	-0.074	-0.017	0.091
	(0.055)	(0.012)	(0.067)
UMIC	-0.109	-0.025	0.134
	(0.069)	(0.017)	(0.085)
HIC	-0.241***	-0.056**	0.297***
	(0.071)	(0.019)	(0.088)
Survey month (ref -- Nov 2020 - Jan 2021)			
March - May 2021	-0.054	-0.011	0.065
	(0.081)	(0.016)	(0.098)
June - August 2021	-0.046	-0.010	0.055
	(0.065)	(0.012)	(0.077)
New COVID-19 cases per million, terciles (ref. – top tercile)			
Cases (middle tercile)	0.016	0.003	-0.019
	(0.048)	(0.010)	(0.058)
Cases (bottom tercile)	-0.028	-0.006	0.034
	(0.037)	(0.008)	(0.045)
Oxford stringency index terciles (ref. – top tercile)			
Stringency (middle tercile)	0.089	0.020	-0.110
	(0.057)	(0.013)	(0.070)
Stringency (bottom tercile)	0.012	0.003	-0.015
	(0.041)	(0.011)	(0.052)

For peer review only - http://bmjopen.bmj.com/site/about/guidelines.xhtml

Dep. Var.:	Would you get the vaccine?		
	No	Not sure	Yes
WHO excess deaths due to COVID-19 terciles (ref.- bottom tercile)			
Excess deaths (middle tercile)	-0.075	-0.019	0.094
	(0.057)	(0.015)	(0.071)
Excess deaths (top tercile)	0.057	0.010	-0.067
	(0.096)	(0.015)	(0.112)
Confidence in government index tercile (ref.- top tercile)			
Confidence in government (middle tercile)	0.052	0.016	-0.069
	(0.041)	(0.013)	(0.054)
Confidence in government (bottom tercile)	0.040	0.013	-0.053
	(0.070)	(0.023)	(0.093)
R-squared		0.0866	
N		65088	

Notes: Weighted ordinal logit regressions. Categories ranked from no to not sure to yes. Marginal effects reported. Standard errors clustered at country level. *, **, *** indicates significance at the 95%, 99%, and 99.9% level.

For peer review only

For peer review only - http://bmjopen.bmj.com/site/about/guidelines.xhtml

Supplemental Table S6: Percent of population non-hesitant to take the COVID-19 vaccine, 14 countries with multiple rounds of survey results (95% confidence intervals in parentheses).

country	**Oct-2020**	**Nov-2020**	**Dec-2020**	**Jan-2021**	**Feb-2021**	**Mar-2021**	**Apr-2021**	**May-2021**	**Jun-2021**	**Jul-2021**	**Aug-2021**
Burkina Faso			0.79 (0.73, 0.86)						0.76 (0.67, 0.86)		
Congo, Rep.			0.84 (0.75, 0.94)			0.87 (0.81, 0.93)					
Ethiopia	0.98 (0.91, 1.00)				0.97 (0.89, 1.00)						
Gambia, The			0.65 (0.58, 0.71)				0.55 (0.49, 0.60)				0.67 (0.60, 0.74)
Georgia				0.36 (0.33, 0.38)		0.29 (0.27, 0.32)			0.35 (0.32, 0.38)		
Indonesia		0.79 (0.73, 0.85)				0.79 (0.72, 0.85)					
Iraq			0.66 (0.61, 0.72)	0.50 (0.45, 0.54)					0.45 (0.40, 0.50)	0.47 (0.42, 0.52)	0.53 (0.47, 0.58)
Kazakhstan					0.23 (0.17, 0.28)			0.32 (0.27, 0.37)	0.25 (0.21, 0.29)		
Malawi		0.83 (0.76, 0.89)				0.52 (0.47, 0.58)	0.71 (0.64, 0.78)				
Nigeria	0.86 (0.80, 0.93)				0.83 (0.77, 0.90)						
Philippines			0.44 (0.40, 0.48)					0.46 (0.42, 0.51)			
Tajikistan								0.73 (0.61, 0.84)	0.79 (0.67, 0.92)	0.80 (0.65, 0.95)	0.73 (0.57, 0.90)
Uganda		0.84 (0.79, 0.90)			0.88 (0.83, 0.94)						
Uzbekistan							0.55 (0.51, 0.60)	0.50 (0.46, 0.54)	0.45 (0.41, 0.49)		

For peer review only

For peer review only - http://bmjopen.bmj.com/site/about/guidelines.xhtml

Supplemental Table S7: Comparison of results on vaccine hesitancy from the HFPS and other studies

**Comparison with de Figueiredo and Larson (2021)**		
	**HFPS**	**de Figueiredo and Larson**
Argentina	10.1%	24.3%
Brazil	3.1%	17.0%
Chile	3.4%	27.9%
Croatia	33.2%	58.5%
Ecuador	19.4%	20.3%
Indonesia	21.3%	17.1%
Lebanon	32.2%	55.9%
Malaysia	25.8%	13.9%
Mexico	6.2%	18.0%
Nigeria	15.2%	35.9%
Paraguay	15.3%	48.5%
Peru	10.5%	28.3%
Vietnam	15.9%	3.2%
Average	*16.3%*	*28.4%*
**Comparison with Wouters et al. 2021**		
	**HFPS**	**Wouters et al.**
Argentina	10.1%	24.0%
Brazil	3.1%	12.0%
Chile	3.4%	28.0%
Croatia	33.2%	59.0%
Ecuador	19.4%	20.0%
Indonesia	42.6%	17.0%
Lebanon	32.2%	56.0%
Mexico	6.2%	18.0%
Nigeria	30.3%	36.0%
Paraguay	15.3%	49.0%
Peru	10.5%	28.0%
Vietnam	15.9%	2.0%
Average	*18.5%*	*29.1%*
**Comparison with Gallup (2021)**		
**Country**	**HFPS Hesitancy**	**Gallup**
Argentina	10.1%	37.0%
Bolivia	24.2%	35.0%
Brazil	3.1%	30.0%
Burkina Faso	22.2%	44.0%
Chile	3.4%	40.0%
Colombia	11.2%	30.0%
Congo, Dem. Rep.	61.2%	61.2%
Congo, Rep.	14.4%	48.0%

For peer review only - http://bmjopen.bmj.com/site/about/guidelines.xhtml

Costa Rica	11.9%	26.0%
Croatia	33.2%	57.0%
Dominican Republic	5.0%	35.0%
Ecuador	19.4%	28.0%
El Salvador	8.1%	25.0%
Ethiopia	2.8%	16.0%
Georgia	66.8%	44.0%
Guatemala	29.6%	29.0%
Guinea	20.1%	46.0%
Honduras	13.9%	30.0%
Indonesia	21.3%	30.0%
Iraq	47.8%	39.0%
Jamaica	50.6%	68.0%
Kenya	17.9%	27.0%
Lao PDR	12.9%	16.0%
Lebanon	32.2%	57.0%
Malaysia	25.8%	28.0%
Mali	21.1%	51.0%
Mexico	6.2%	25.0%
Mongolia	19.2%	39.0%
Nicaragua	18.8%	13.0%
Nigeria	15.2%	42.0%
Paraguay	15.3%	47.0%
Peru	10.5%	27.0%
Philippines	54.9%	49.0%
Tajikistan	23.7%	36.0%
Thailand	36.6%	39.0%
Uganda	13.6%	38.0%
Uruguay	9.1%	39.0%
Uzbekistan	49.8%	30.0%
Vietnam	15.9%	19.0%
Zimbabwe	15.8%	27.0%
Average	*22.4%*	*36.2%*
**Comparison with Facebook (2021)**		
**Country**	**HFPS**	**FB**
Argentina	10.1%	21.5%
Belize	28.4%	48.0%
Brazil	3.1%	25.8%
Chile	3.4%	43.0%
Colombia	11.2%	15.7%
Costa Rica	11.9%	17.1%
Ecuador	19.4%	17.1%

For peer review only - http://bmjopen.bmj.com/site/about/guidelines.xhtml

El Salvador	8.1%	15.8%
Guatemala	29.6%	18.6%
Guyana	20.0%	54.4%
Honduras	13.9%	13.7%
Indonesia	21.3%	19.2%
Iraq	47.8%	52.8%
Lebanon	32.2%	22.3%
Mexico	6.2%	13.0%
Nicaragua	18.8%	37.4%
Panama	13.3%	24.9%
Paraguay	15.3%	16.2%
Peru	10.5%	12.2%
Uruguay	9.1%	50.8%
Average	*16.7%*	*27.0%*

For peer review only

For peer review only - http://bmjopen.bmj.com/site/about/guidelines.xhtml

Supplemental Figure S1: Vaccination rates by income group

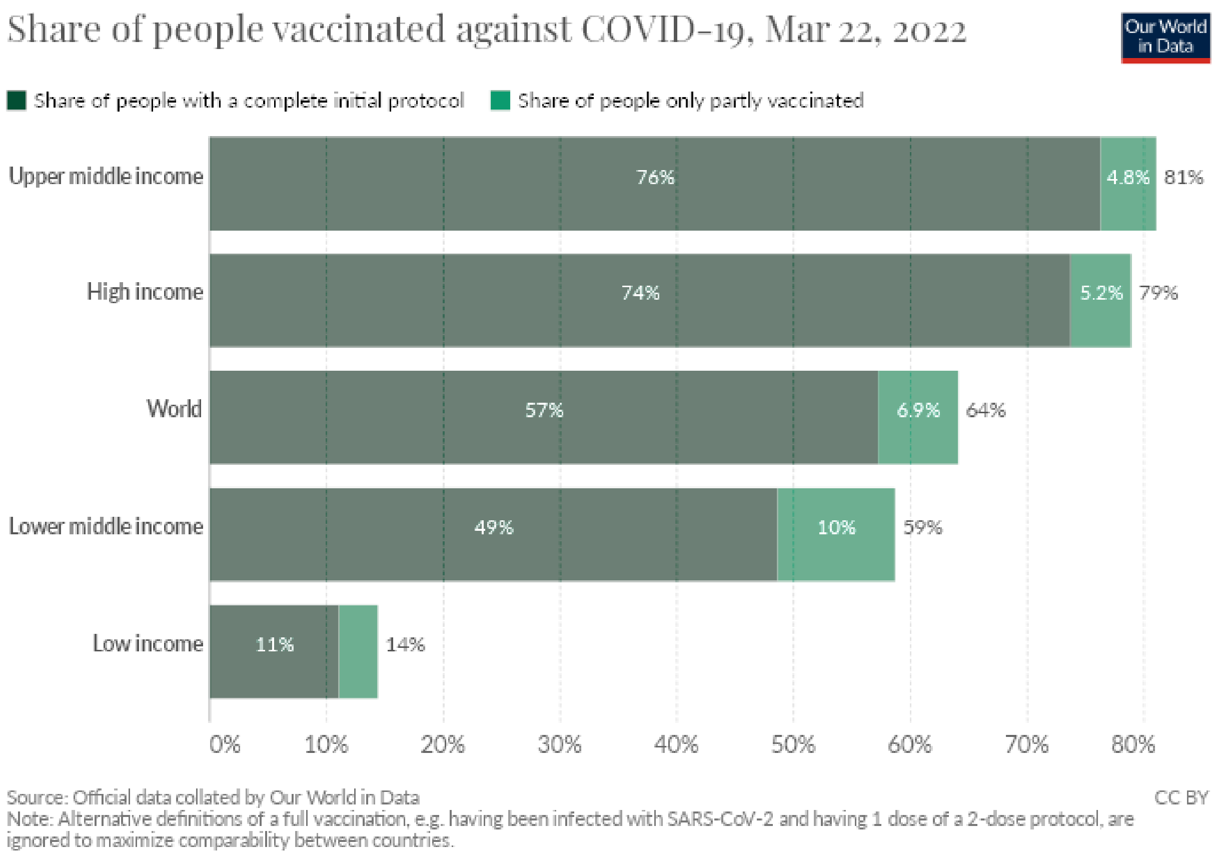

Data source: Ritchie et al. 2022 as of March 22, 2022.

w only

Supplemental Figure S2: Vaccine rollout rates by income group

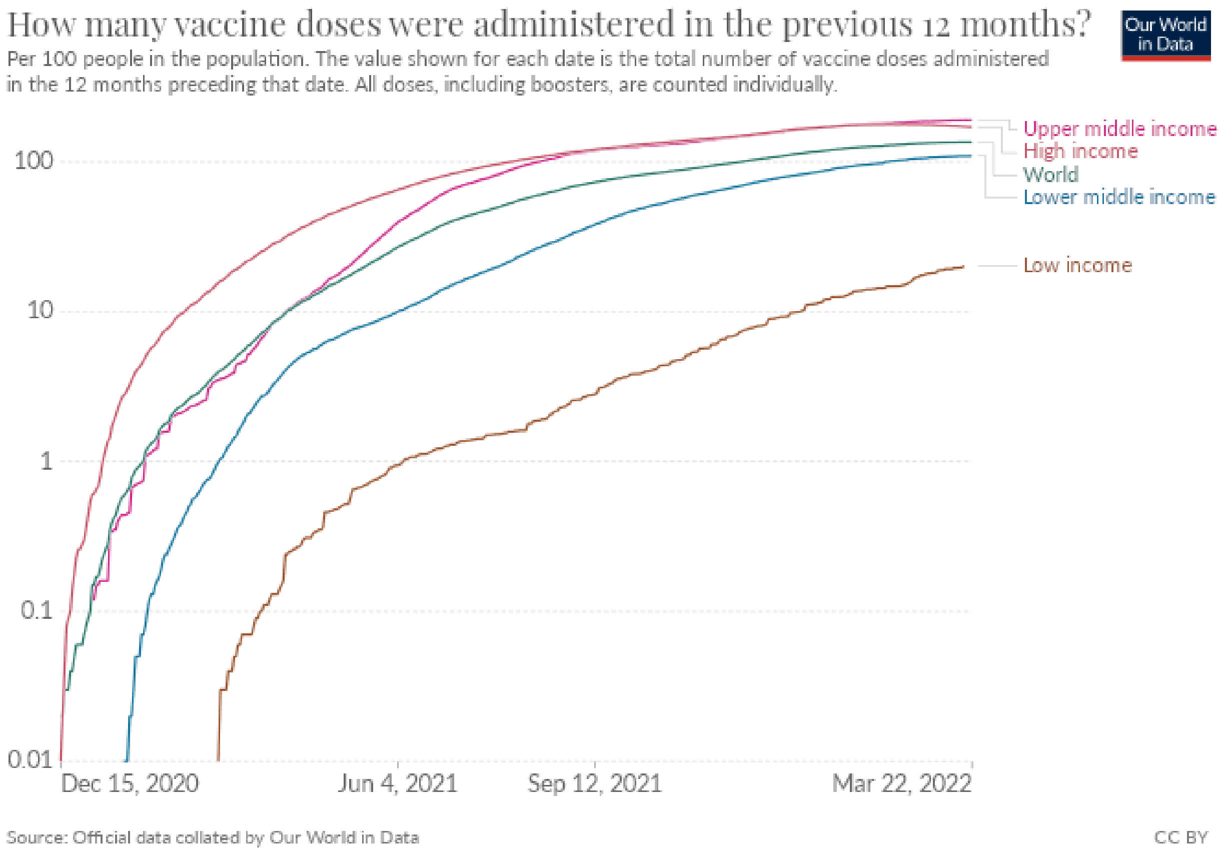

Data source: Ritchie et al. 2022 as of March 22, 2022.

For peer review only - http://bmjopen.bmj.com/site/about/guidelines.xhtml

Supplemental Figure S3: Comparison of share of population COVID-19 that is hesitant to take the COVID-19 vaccine, HFPS with other studies

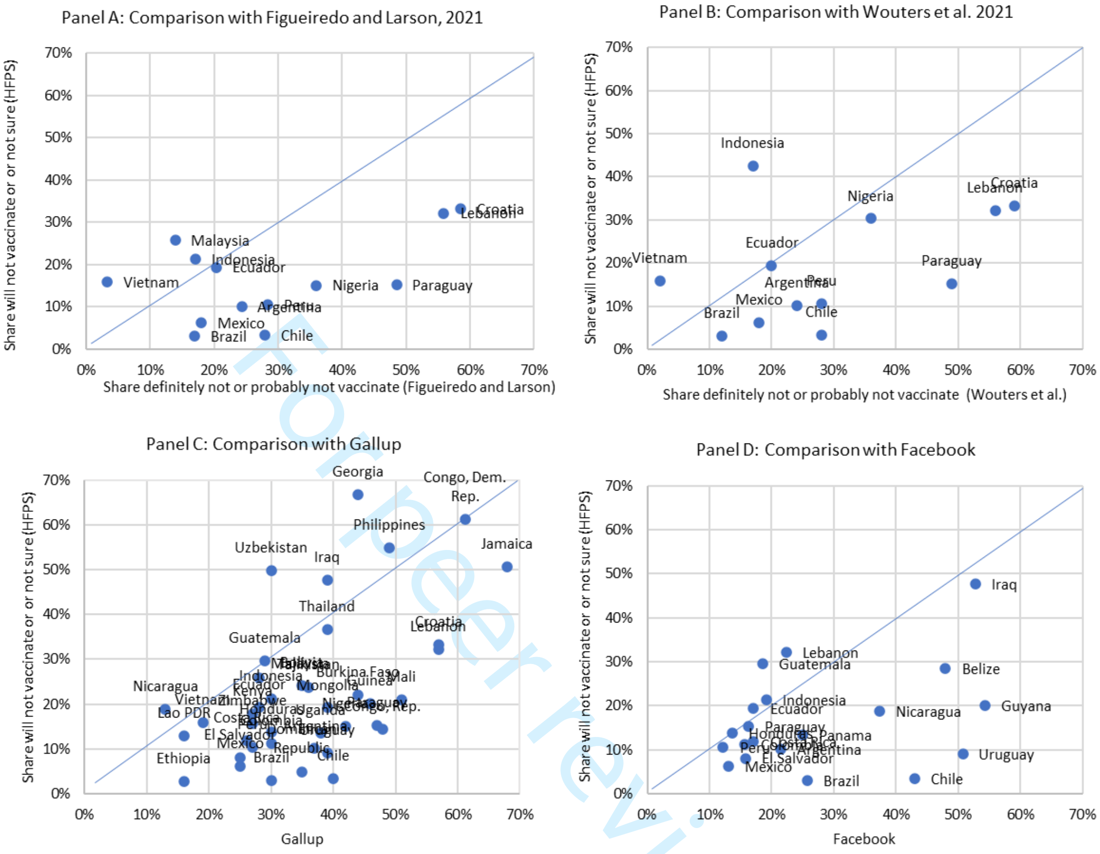

Notes: The y-axes indicate the share of the sample who reported not being willing or not sure if they will take the COVID-19 vaccine, as reported in the HFPS. The x-axes indicate the share of households in the survey that will definitely or probably not be vaccinated against COVID-19 from the following sources. Panel A: Figueiredo and Larson (2021). Panel B: Wouters et al. (2021). Panel C: Gallup (2021). Panel D: Facebook (2021).

For peer review only - http://bmjopen.bmj.com/site/about/guidelines.xhtml

Supplemental Figure S4: Comparison of COVID-19 Vaccine Hesitancy with pre-COVID sentiment about vaccines from 2019 Global Monitor, 45 countries

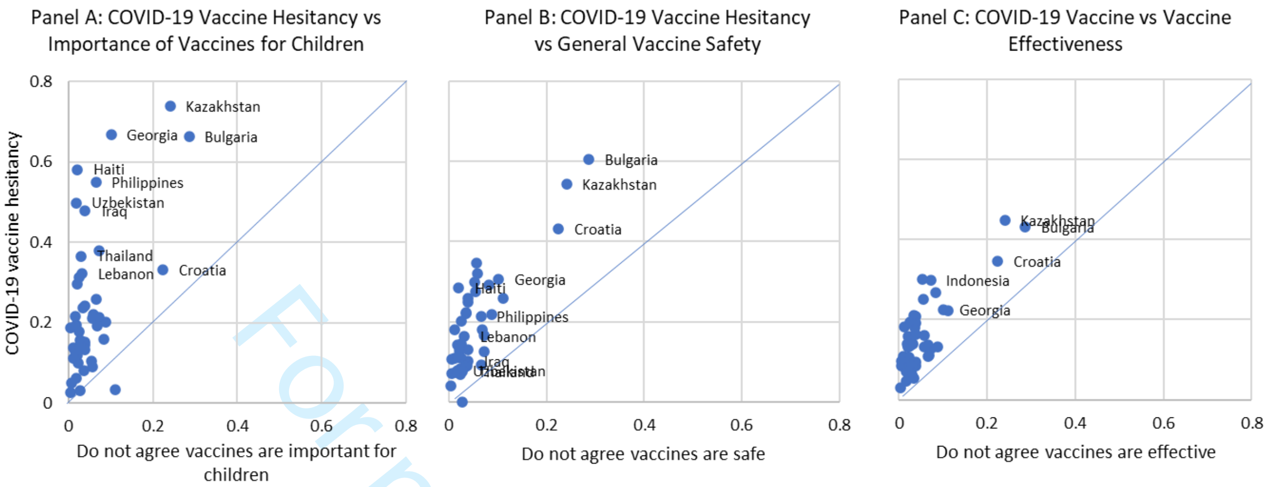

Notes: The y-axes indicate the share of the sample who report either not willing or not sure if they will vaccinate, as reported in the HFPS. The x-axes indicate the share of the 2019 Global Monitor Report sample that reported they do not agree vaccines are important for children (Panel A), do not agree that vaccines are safe (Panel B), and do not agree vaccines are effective.

For peer review only

Supplemental Figure S5: Reason for "definitely not" (upper panel) or "probably not" (lower panel) taking the COVID-19 vaccine, Facebook data for month of HFPS)

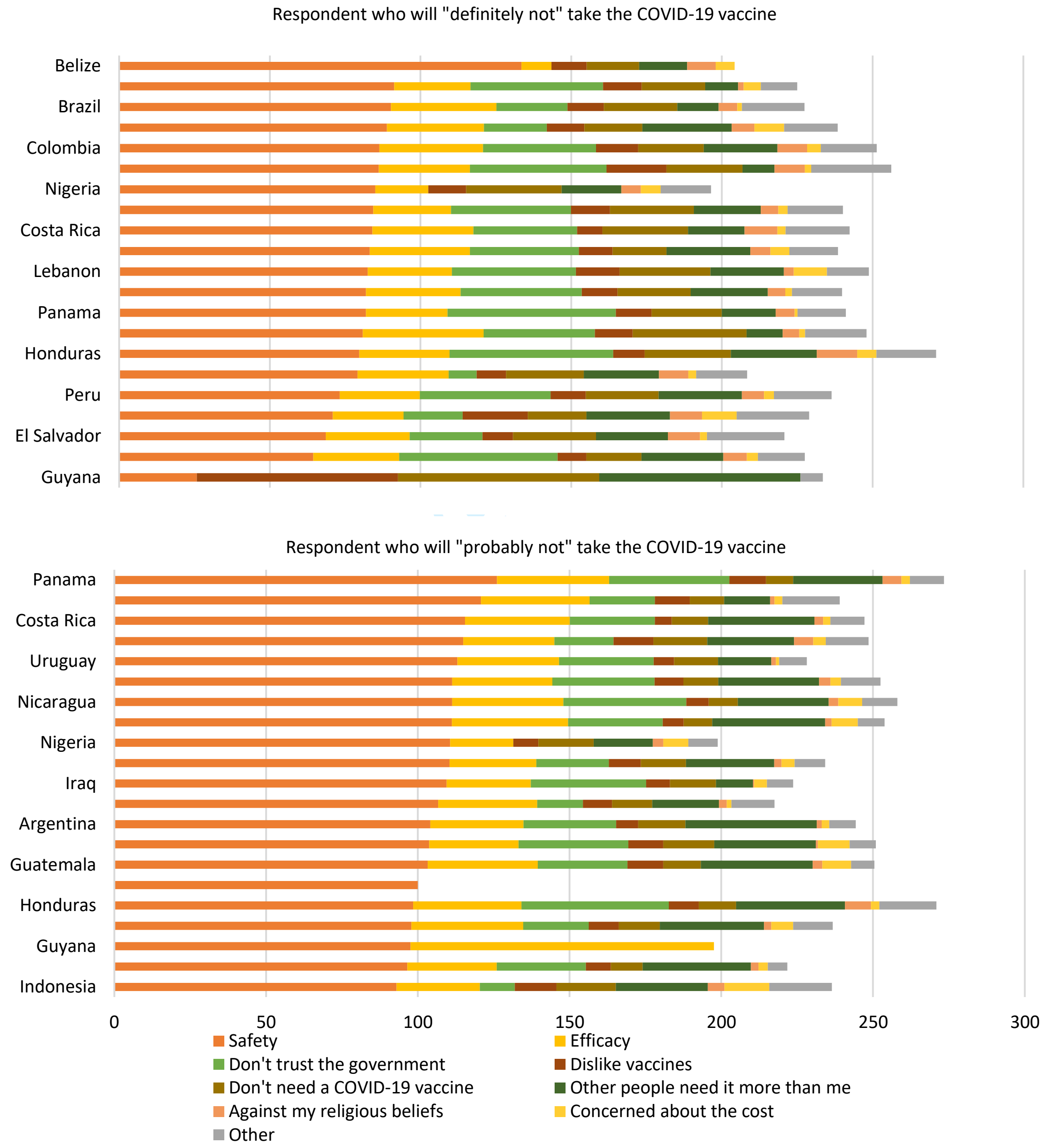

Notes: Y-axes indicate the share of survey respondents; multiple answers were allowed. Data source: Facebook (2021) for same month as reported in the HFPS. The month for each country is reported in Supplemental Table S1.

For peer review only - http://bmjopen.bmj.com/site/about/guidelines.xhtml

References- Supplemental Materials

Facebook. 2021. COVID-19 Trends and Impact Survey.

de Figueiredo A, Larson HJ. Exploratory study of the global intent to accept COVID-19 vaccinations. Communications medicine. 2021 Sep 9;1(1):1-0.

Gallup. 2019. Wellcome Global Monitor – First Wave Findings. https://wellcome.org/reports/wellcome-global-monitor/2018#downloads-4d1c.

Gallup. 2021. Gallup World Poll. https://www.gallup.com/178667/gallup-world-poll-work.aspx.

Hale T, Angrist N, Goldszmidt R, et al. 2022. A global panel database of pandemic policies (Oxford COVID-19 government response Tracker). Nat Hum Behav 2021;5:529–38. Database accessed Feb 28, 2022.

Inglehart, R, C. Haerpfer, A. Moreno et al. 2022. Would Values Survey: Round Seven – Country Pooled Datafile Version: https://www.worldvaluessurvey.org/WVSOnline.jsp. (accessed April 21, 2022) Madrid: JD Systems Institute.

Lazarus, JV, Ratzan SC , and Palayew A et al. 2021. A global survey of potential acceptance of a COVID-19 vaccine. Brief Communication. Nature Medicine 27: 225–228. https://doi.org/10.1038/s41591-020-1124-9.

Sallam M. COVID-19 Vaccine Hesitancy Worldwide: A Concise Systematic Review of Vaccine Acceptance Rates. Vaccines (Basel). 2021 Feb 16;9(2):160. doi: 10.3390/vaccines9020160. PMID: 33669441; PMCID: PMC7920465.

Solís Arce, J.S., Warren, S.S., Meriggi, N.F. *et al.* COVID-19 vaccine acceptance and hesitancy in low- and middle-income countries. *Nat Med* **27,** 1385–1394 (2021). https://doi.org/10.1038/s41591-021-01454-y.

World Bank. 2020. High Frequency Mobile Phone Surveys of Households to Assess the Impacts of COVID-19: Overview (English). Washington, D.C.: World Bank Group. http://documents.worldbank.org/curated/en/703571588695361920/Overview

World Bank. 2021. World Development Indicators. Washington, D.C.: The World Bank (producer and distributor). http://data.worldbank.org/data-catalog/world-development-indicators. Accessed March 12, 2022.

World Health Organization. 2022. Global Excess Deaths Associated with COVID-19 (Modelled Estimates). Downloaded on September 8, 2022 at https://www.who.int/data/sets/global-excess-deaths-associated-with-covid-19-modelled-estimates.

Wouters, O.J., Shadlen, K.C., Salcher-Konrad, M., Pollard, A.J., Larson, H.J., Teerawattananon, Y. and Jit, M., 2021. Challenges in ensuring global access to COVID-19 vaccines: production, affordability, allocation, and deployment. The Lancet, 397(10278), pp.1023-1034.

For peer review only - http://bmjopen.bmj.com/site/about/guidelines.xhtml

BMJ Open

How prevalent is COVID-19 Vaccine Hesitancy in Developing Countries and What Are the Key Drivers of Hesitancy? Results from 53 Countries

Journal:	*BMJ Open*
Manuscript ID	bmjopen-2022-069152.R1
Article Type:	Original research
Date Submitted by the Author:	18-Jul-2023
Complete List of Authors:	Dayton Eberwein, Julia; World Bank Group, HNP GP<br>Edochie, Ifeanyi; World Bank Group<br>Newhouse, David; World Bank Group<br>Cojocaru, Alexandru; World Bank Group<br>Bopahbe, Gildas; World Bank Group<br>Kakietek, Jakub; World Bank Group<br>Kim, Yeon Soo; World Bank Group<br>Montes, Jose; World Bank Group
**Primary Subject Heading**:	Global health
Secondary Subject Heading:	Health policy, Infectious diseases, Public health
Keywords:	COVID-19, Health policy < HEALTH SERVICES ADMINISTRATION & MANAGEMENT, Public health < INFECTIOUS DISEASES

SCHOLARONE™
Manuscripts

I, the Submitting Author has the right to grant and does grant on behalf of all authors of the Work (as defined in the below author licence), an exclusive licence and/or a non-exclusive licence for contributions from authors who are: i) UK Crown employees; ii) where BMJ has agreed a CC-BY licence shall apply, and/or iii) in accordance with the terms applicable for US Federal Government officers or employees acting as part of their official duties; on a worldwide, perpetual, irrevocable, royalty-free basis to BMJ Publishing Group Ltd ("BMJ") its licensees and where the relevant Journal is co-owned by BMJ to the co-owners of the Journal, to publish the Work in this journal and any other BMJ products and to exploit all rights, as set out in our licence.

The Submitting Author accepts and understands that any supply made under these terms is made by BMJ to the Submitting Author unless you are acting as an employee on behalf of your employer or a postgraduate student of an affiliated institution which is paying any applicable article publishing charge ("APC") for Open Access articles. Where the Submitting Author wishes to make the Work available on an Open Access basis (and intends to pay the relevant APC), the terms of reuse of such Open Access shall be governed by a Creative Commons licence – details of these licences and which Creative Commons licence will apply to this Work are set out in our licence referred to above.

Other than as permitted in any relevant BMJ Author's Self Archiving Policies, I confirm this Work has not been accepted for publication elsewhere, is not being considered for publication elsewhere and does not duplicate material already published. I confirm all authors consent to publication of this Work and authorise the granting of this licence.

For peer review only

How prevalent is COVID-19 Vaccine Hesitancy in Developing Countries and What Are the Key Drivers of Hesitancy? Results from 53 Countries

Julia Dayton Eberwein,[1*] Ifeanyi Edochie,[1] David Newhouse, [1] Alexandru Cojocaru, [1] Gildas Deudibe, [1] Jakub Kakietek,[1] Yeon Soo Kim, [1] and Jose Montes[1]

September 2022

For peer review only

Abstract

Objectives: To estimate the levels of COVID-19 vaccine hesitancy in 53 developing countries, differences across population groups in hesitancy, and self-reported reasons for being hesitant to take the COVID-19 vaccine.

Methods: This paper presents new evidence on levels and trends of vaccine hesitancy in developing countries based on harmonized high-frequency phone surveys from more than 120,000 respondents in 53 low- and middle-income countries collected between October 2020 August 2021. These countries represent a combined 53 percent of the population of low- and middle-income countries excluding India and China.

Results: On average across countries, one in five adults reported being hesitant to take the COVID-19 vaccine, with the most cited reasons for hesitancy being concerns about the safety of the vaccine, followed by concerns about its efficacy. Between late 2020 and the first half of 2021, there tended to be little change in hesitancy rates in 11 of the 14 countries with available data, while hesitancy increased in Iraq, Malawi, and Uzbekistan. COVID-19 vaccine hesitancy was higher among female, younger adults, and less educated respondents, after controlling for selected observable characteristics.

Conclusions: Country estimates of vaccine hesitancy from the high-frequency phone surveys are correlated with but lower than those from earlier studies, which often relied on less representative survey samples. The results suggest that vaccine hesitancy in developing countries, while less prevalent than previously thought, will be an important and enduring obstacle to recovery from the pandemic.

[1] The World Bank, 1818 H. Street NW, Washington, DC 20433

* Corresponding author: jdayton@worldbank.org.

Strengths and limitations of this study

- The greatest strength of the study is that is provides nationally representative and comparable estimates of COVID-19 vaccine hesitancy across over 50 developing countries, filling a critical evidence gap.
- Another strength of the study is how it extends our understanding of the heterogeneity in hesitancy across demographic characteristics of the respondent, as well as reasons for their hesitancy to take the COVID-19 vaccine, thus providing a rich set of results which can aid policy makers in the design of vaccination campaigns in developing countries.
- One potential limitation of this study is that it is based on phone survey data which exclude respondents who did not have access to a phone, although access to mobile phones was high in the countries in our sample and where possible sampling weights were used to correct for this bias.

For peer review only

1. Introduction

The world is entering the third year of the global COVID-19 pandemic, which has caused enormous devastation to both people's health (450 million cases and 6 million deaths as of March 2022) and national economies, in the form of a global recession that has pushed millions into poverty (1,2). With the continued emergence of new variants and limited treatments available, it is commonly accepted that widespread vaccination is the world's best bet to contain the virus and it is also expected to play an important role in economic recovery (3,4). As of early March 2022, over 60 percent of the world's population had received at least one dose of a COVID-19 vaccine. However, there are stark disparities in vaccination rates across countries: only 14 percent of people in low-income countries had received at least one dose as of March 22, 2022, compared to 79 percent in high-income countries and 81 percent in upper-middle income countries (5) (supplemental figures S1 and S2). The lag in vaccine distribution in lower income countries had until recently put the focus mainly on supply-side constraints. However, with vaccine production now exceeding demand, it is increasingly important to understand the extent of vaccine hesitancy and the nature of the concerns.

Vaccine hesitancy refers to the delay in acceptance or refusal to vaccinate despite availability of vaccination services (6). It is not a new phenomenon, and some level of hesitancy exists for most vaccines; even before the COVID-19 pandemic began, the World Health Organization (WHO) identified vaccine hesitancy as one of the top threats to global health (7). Brewer and colleagues established a conceptual framework of behavioral and social drivers (BeSD) of vaccination that has been widely adopted, including by the WHO (8). These drivers of vaccination can be grouped and measured in four domains: thinking and feeling about vaccines (including confidence in vaccine effectiveness and concern about safety); social processes that drive or inhibit vaccination; motivation (or hesitancy) to seek vaccination; and practical issues involved in seeking and receiving vaccination. This framework is focused on proximal factors affecting decisions to vaccinate, which are thought to be potentially changeable by programs. Several aspects of the thinking and feeling domain are thought to contribute substantially to COVID-19 vaccine hesitancy: the accelerated speed of development of the COVID-19 vaccine, its newness, concerns about longer-term side-effects, uncertainty about the duration of the vaccine's effectiveness, and the type of vaccine (e.g., mRNA) and country of origin of the vaccine (9,10-12).

The multi-country studies that exist show relatively high levels of hesitancy to take the COVID-19 vaccine, but mainly focus on high-income countries (13-14). Less is understood about COVID-19 vaccine hesitancy in low- and middle-income countries. A few studies reported multi-country survey results from developing countries (15-20) or systematic reviews of individual country surveys (21), but few of the studies were based on nationally representative samples of respondents or included comparable results for many countries. Given this limitation, an important contribution of this analysis will be to validate these findings.

This paper extends the existing literature by providing estimates of and reasons for vaccine hesitancy for 53 developing countries, which are comparable across countries and largely based on household surveys that are more representative of the national population. The 53 countries represent approximately 30% of the population of all low- and middle-income countries and 53% excluding India and China.

For peer review only - http://bmjopen.bmj.com/site/about/guidelines.xhtml

2. Data and Methods

2a. Data

This study describes the levels of vaccine hesitancy and its reasons in 53 developing countries between October 2020 August 2021 using data from the World Bank's COVID-19 high-frequency phone surveys (HFPS) which were implemented to monitor the impact of COVID-19 on households around the world (22, 23). Data are available for one survey round from 39 countries and two or more survey rounds for 14 countries (supplemental table S1). The sampling frame was drawn from pre-existing nationally representative household surveys in 19 countries, random digit dialing (RDD) in 29 countries, and a list of phone numbers typically obtained from mobile phone operators in 5 countries (see supplement 1 for detailed description of methods).

2b. Outcome measures

The question about vaccine hesitancy, "When a vaccine to prevent COVID-19 is available to you, are you planning to be vaccinated?" varied slightly across surveys depending on whether a vaccine was available in the country at the time of the survey (See supplement 1). In the sample, 47 countries offered three answer options (yes, not sure, and no), while the other six offered two categories (yes and no). We combine the "no" and "not sure" answers to obtain the measure of vaccine hesitancy.

To obtain the respondent's reason for vaccine hesitancy, survey respondents who answered "no" or "not sure" were asked "What is your (main) reason/concern for not wanting to be vaccinated / not being sure if you want to be vaccinated?" The answer categories varied widely across surveys (supplemental table S2a). To make these more comparable across countries, answers were remapped into the nine most common categories (supplemental table 2b). The surveys also differed across countries in terms of whether a single concern or multiple concerns were collected, and to account for this, the results are presented separately.

2c. Contextual data

Country-level contextual data were drawn from other sources: new COVID-19 cases per million, measured as a 7-day rolling average prior to the midpoint of the month prior to survey data collection in each country (1); and the Oxford Stringency Index, ranging from 0–100, that indicates the degree to which various restrictions were put in place by governments to control the pandemic (24); confidence in the press, the government (25); and estimated excess deaths due to COVID-19 (26).

2d. Analytical strategy

Data from 53 countries were pooled into a single data set. Sampling weights were scaled such that countries are weighted equally; in other words, the estimates of vaccine hesitancy are unweighted averages of population-weighted country averages. (Using population weights when aggregating across countries would have resulted in the results being driven by a small number of large countries.) First, we report point estimates and 95% confidence intervals for each country based on standard errors clustered at the state or province level within each country. We also report the simple average across all countries and stratified by: World Bank region; World Bank country income group; urban vs. rural residence; gender of respondent; whether respondent is head of household; age of the respondent; and educational attainment of the respondent. Tukey's test of multiple comparisons is used to test differences in rates of

For peer review only

vaccine hesitancy across groups. Second, we use multivariate regression analysis to assess the relative association of vaccine hesitancy and the correlates and contextual variables. Trends over time in levels of vaccine hesitancy are examined in 14 countries with more than one wave of results available. Finally, the analysis describes the reasons for vaccine hesitancy.

2e. Patient and public involvement

No patients were involved in this study. The results of the study will be disseminated to key policy- makers and relevant stakeholders involved in COVID- 19 vaccine deployment.

3. Results

3a. Levels of vaccine hesitancy across 53 countries

Overall, the average level of vaccine hesitancy across the most recent survey round in 53 countries was 20.0% (Confidence Intervals (CI) 17.2-22.7%). Across regions, average levels of hesitancy were highest in Europe and Central Asia (58.8%, CI 55.0-62.6%), followed by the Middle East and North Africa (47.4%, CI 38.8-56.0%), East Asia and Pacific (26.2%, CI 21.4-31.0%), Sub-Saharan Africa (15.5%, CI 11.8-19.2%), and Latin America and the Caribbean (8.0%, CI 6.5-9.5%) (Figure 1 and supplemental table S3). Differences between all regions were statistically significant at the 5% level (supplemental table S4). When considered by country income group, the highest average level of hesitancy was in lower middle-income countries (27.7%, CI 23.8-31.7%), followed by low-income countries (14.6%, CI 7.8-21.4%), upper middle-income countries (12.7%, CI 9.8-15.6%), and high-income countries (5.9%, CI 3.4-8.4). Differences in hesitancy across country income groups were significant at the 5% level across all pairs except for the comparison between low-income and upper-middle income groups.

3b. Bivariate correlates of vaccine hesitancy

Rural households reported significantly higher levels of vaccine hesitancy than urban households (23.2% vs. 17.7%, p-value=0.018), and female respondents (22.5%) were significantly more likely than male respondents (17.3%) to be vaccine hesitant (p-value=0.021). Among households in the 33 surveys with information on the educational attainment of the respondent, respondents with lower levels of education had on average higher levels of hesitancy. Younger respondents reported higher levels of hesitancy than older respondents, but the differences were not significant.

3c. Vaccine hesitancy by country

The highest levels of vaccine hesitancy were reported in Kazakhstan (75.3%), Bulgaria (66.2%) and Georgia (65.2%), and the lowest levels in Chile (3.4%), Ethiopia (3.4%), and Brazil (3.1%) (Figure 2). Strength of vaccine hesitancy was available for the 46 countries that included three answer options (yes, not sure, and no). In four countries (Philippines, Georgia, Jamaica, and Kazakhstan), over 20% of respondents reported being unsure of whether they would get vaccinated, and in another 11 countries, between 10% and 20% of the sample was unsure whether they would be willing to be vaccinated against COVID-19 (supplemental figure S3).

3c. Multivariate analysis of correlates of vaccine hesitancy

Results from a multivariate regression generally confirmed the bivariate results. Results from ordinary least squares (OLS) regression models are shown in Table 1: a baseline model with the correlates described

For peer review only

above plus month of survey (expressed in terciles),[1] and extended models with additional contextual variables (expressed in terciles). Results were consistent across models, as are the marginal effects from logit regressions (supplemental Table S5a). Men were less COVID-19 vaccine hesitant than women, although the magnitude was small. Having more formal education was associated with being less hesitant and the differences were more substantial. Age was inversely correlated with hesitancy. There were no statistically significant differences between hesitancy among rural and urban respondents. Differences across regions were statistically significant and, for some regions, quite large, but there were no significant differences by country income group.

Table 1: Multivariate analysis of vaccine hesitancy in 53 developing countries (Ordinary least squares)

Dep. Var: Hesitancy	(1)	(2)	(3)	(4)	(5)	(6)
Male	-0.028*	-0.027*	-0.028	-0.027*	-0.031*	-0.038**
	(0.013)	(0.013)	(0.014)	(0.013)	(0.013)	(0.012)
Head of HH	0.012	0.009	0.019	0.000	0.011	0.007
	(0.015)	(0.014)	(0.013)	(0.017)	(0.015)	(0.013)
Education of respondent (ref – No education)						
Any primary	-0.062	-0.062*	-0.056	-0.067*	-0.073*	-0.073*
	(0.031)	(0.031)	(0.030)	(0.032)	(0.034)	(0.032)
Any secondary	-0.059	-0.060	-0.053	-0.069	-0.067	-0.082*
	(0.035)	(0.036)	(0.032)	(0.037)	(0.039)	(0.035)
Any tertiary	-0.123***	-0.125***	-0.116***	-0.134***	-0.125**	-0.143***
	(0.033)	(0.035)	(0.032)	(0.037)	(0.040)	(0.036)
Age group (ref. – 34 and younger)						
Working age (35-64)	-0.047**	-0.048***	-0.042**	-0.050***	-0.045**	-0.049***
	(0.014)	(0.013)	(0.015)	(0.013)	(0.015)	(0.012)
Retirement age (65+)	-0.103***	-0.102***	-0.096***	-0.103***	-0.099***	-0.099***
	(0.023)	(0.024)	(0.023)	(0.022)	(0.024)	(0.023)
Rural area	0.016	0.014	0.013	0.010	0.012	0.011
	(0.019)	(0.018)	(0.018)	(0.015)	(0.015)	(0.011)
Region (ref – LAC)						
EAP	0.211**	0.218*	0.241**	0.127	0.299	0.249
	(0.076)	(0.086)	(0.084)	(0.076)	(0.155)	(0.137)
ECA	0.363***	0.364***	0.347***	0.376***	0.369***	0.345***
	(0.075)	(0.076)	(0.073)	(0.076)	(0.056)	(0.068)
MNA	0.231***	0.230***	0.262***	0.240***	0.288***	0.393***
	(0.043)	(0.043)	(0.062)	(0.036)	(0.059)	(0.050)
SSA	0.082	0.089	0.078	-0.005	0.072	0.057
	(0.075)	(0.079)	(0.074)	(0.101)	(0.086)	(0.103)
Country income group (ref. – LIC)						
LMIC	-0.073	-0.071	-0.052	-0.090	-0.049	-0.053
	(0.127)	(0.124)	(0.122)	(0.129)	(0.113)	(0.081)
UMIC	-0.052	-0.050	-0.071	-0.096	-0.026	-0.130
	(0.154)	(0.154)	(0.146)	(0.154)	(0.135)	(0.107)
HIC	-0.161	-0.160	-0.183	-0.208	-0.175	-0.280*
	(0.159)	(0.162)	(0.153)	(0.162)	(0.136)	(0.108)
Survey month (ref – Nov 2020 – Jan 2021)						

[1] Time of survey was split into the following 3 groups: (i) November 2020 – January 2021; (ii) March – May 2021; and (iii) June – August 2021. There are no observations in the data for February and April of 2021.

For peer review only

Dep. Var: Hesitancy	(1)	(2)	(3)	(4)	(5)	(6)
March – May 2021	-0.073	-0.062	-0.102	-0.056	-0.113	-0.110
	(0.095)	(0.108)	(0.104)	(0.101)	(0.112)	(0.095)
June – August 2021	-0.018	-0.012	-0.018	-0.028	-0.049	-0.026
	(0.090)	(0.099)	(0.099)	(0.089)	(0.097)	(0.079)
New COVID-19 cases per million, terciles						
Cases (middle tercile)		-0.022				0.023
		(0.065)				(0.057)
Cases (top tercile)		-0.009				-0.011
		(0.054)				(0.049)
Oxford stringency index terciles (ref. – bottom tercile)						
Stringency (middle tercile)			0.061			0.148
			(0.073)			(0.077)
Stringency (top tercile)			-0.005			0.028
			(0.060)			(0.059)
WHO excess deaths due to COVID-19 (ref.- bottom tercile)						
Excess deaths (middle tercile)				-0.110*		-0.123
				(0.050)		(0.080)
Excess deaths (top tercile)				-0.059		0.045
				(0.067)		(0.106)
Confidence in government index tercile (ref.- top tercile)						
Confidence in government (middle tercile)					0.073	0.105
					(0.054)	(0.064)
Confidence in government (top tercile)					0.016	0.055
					(0.131)	(0.135)
Constant	0.405**	0.411**	0.381*	0.529***	0.331*	0.333*
	(0.138)	(0.143)	(0.146)	(0.142)	(0.157)	(0.153)
R-squared	0.090	0.090	0.095	0.097	0.102	0.123
N	65088	65088	65088	65088	65088	65088

Notes: Weighted OLS regressions. Marginal effects reported. Standard errors clustered at country level. Ref=reference group; EAP= East Asia and the Pacific; ECA= Europe and Central Asia; LAC=Latin America and the Caribbean; MNA= Middle East and North Africa; SSA= Sub-Saharan Africa; LIC=Low-income countries; LMIC=Lower middle-income countries; UMIC=Upper middle-income countries.*, **, *** indicates significance at the 95%, 99%, and 99.9% level.

In the extended regression models, we tested two additional hypotheses. First, we expected that the severity of the pandemic (for which we use three different proxies: number of new cases of COVID-19 in the country in column 2, the degree of stringency in the government's policy measures as captured by the Oxford Stringency index in column 3, and estimated COVID-19 related excess deaths in column 4) would be negatively correlated with hesitancy. In our data, none of the three proxies for pandemic severity is statistically associated with vaccine hesitancy with the exception of COVID-19 excess deaths, where in countries in the second tercile of the excess deaths distribution vaccine hesitancy was, on average, lower, relative to the bottom tercile baseline. We also tested whether trust in the government in general was

For peer review only

associated with COVID-19 vaccine hesitancy, but this association was not statistically significant in the regression model (column 5). We also considered a regression model with vaccine hesitance as an ordinal variable, in case the underlying characteristics of those who answered “No” and “Not sure” differed. The pattern of the results was similar, but the associations were weaker for the “Not sure” group (supplemental table S5b).

3d. Changes in vaccine hesitancy over time

In the 14 countries for which estimates of vaccine hesitancy were available for two or more survey rounds (figure 3 and supplemental table S6), there were no clear patterns in terms of the changes in vaccine hesitancy in countries with multiple data points over the pandemic. COVID-19 vaccine hesitancy declined in half of the countries and increased in the other half, and changes in either direction were less than five percentage points in all but three countries (Iraq, Malawi, Uzbekistan). No patterns of change over time were observed across sub-populations either.

3e. Reasons for COVID-19 vaccine hesitancy

Overall, the most common concern pertained to the safety of the vaccine, including concerns about side effects (43% of respondents in counties with the single answer option) (figure 4). The second most common concern related to efficacy (19%) and all other reasons accounted for less than 10 percent overall. These averages mask significant variation across countries. Concerns about vaccine efficacy were greater or equal to concerns about safety in five Latin American countries (Argentina, Chile, Dominica, Dominican Republic, and Mexico). Respondents in the Democratic Republic of Congo and the Republic of Congo were mainly hesitant because of dislike for vaccines in general and (distrust of the international community). In Georgia, the only country in Europe and Central Asia with information on reasons for hesitancy, the most cited reason was “not eligible” (supplemental figure S4). Only one country, the Republic of Congo, reported reasons for two waves, and only small changes in reasons were observed.

4. Discussion

4a. Principal findings

On average across 53 countries, one in five adults were hesitant about getting a COVID-19 vaccine. The highest levels were observed in Eastern European and Central Asian countries and the lowest levels in Latin American and Caribbean countries. Female respondents, younger adults, and those with less formal education reported higher levels of COVID-19 vaccine hesitancy than their respective counterparts. Between October 2020 and August 2021, there tended to be little change in levels of hesitancy except in Iraq, Malawi, and Uzbekistan, where hesitancy increased. The main self-reported reason for being hesitant was concerns over safety, especially worries about side effects.

4b. Strengths and comparisons with other studies

This study has several advantages over existing literature. It is based on a very large sample of respondents from national surveys in 53 developing countries, a part of the world that is under-represented in the literature. Over half of the surveys used random digit dialing, typically in upper middle-income countries in which a large share of the population uses mobile phones. In lower income contexts, most surveys, 35 percent overall, sampled based on previous face-to-face surveys which were in turn drawn from a census frame. In the remaining 10 percent of cases, sampling was carried out from lists provided by mobile phone

For peer review only

operators. In each case, surveys drawn from pre-existing face-to-face surveys were reweighted using the baseline data to become more representative, while many random digit dialing surveys were also reweighted to make those samples more representative of the universe of phone numbers. While it is impossible to eliminate issues of representativeness in phone surveys, the HFPSs were typically carried out by National Statistics Offices and are more plausibly representative than convenience web surveys.

Overall, the estimates of COVID-19 vaccine hesitancy from the HFPSs were more conservative than other published estimates (supplemental figures S5). There are several possible explanations for the differences. The first relates to the timing of data collection. Most of the existing literature is based on surveys carried out in mid to late 2020 (14, 15, 17, 19-20), whereas the HFPS data included in our sample were collected from end-2020 through August 2021. It is plausible to believe COVID-19 vaccine hesitancy generally declined during this period as multiple vaccines became available, and widespread vaccination was safely rolled out in high-income countries. Another possible reason for the differences is the variation in how the vaccine hesitancy question was framed and the response options available. For example, some studies used a four- or five-point Likert scale of agreement (14-16, 20, 27), whereas the HFPS relied on two or three answer options. Finally, variations in survey modality may have created biases. Among the published studies included for comparison, many studies were based on data from commercial online sample providers, often using quotas sampling to ensure an appropriate distribution in terms of gender, age, and region (14-17,20, 27), while others were based on convenience samples (11, 28-30), or a mix of methods (19, 21). There was also wide variety in the survey mode, including online, computer-assisted telephone, and face-to-face surveys.

It is not possible to know the extent to which each of the above-mentioned potential sources of bias affect the comparisons of the findings from the HFPS with the others. For example, it was possible to match the Facebook survey results for the month and year of the HFPS for 20 countries, thus taking away any differences in the timing of the survey. Nevertheless, the HFPS estimates were still lower than those reported by Facebook (supplemental figure S5, panel D), and this could be due to differences in question-and-answer wording and/or the sample frame.

Even though the HFPS estimates are lower than other sources, the levels of COVID-19 vaccine hesitancy reported here are nonetheless higher than the levels of vaccine hesitancy reported for childhood vaccines. We compared the average country levels of COVID-19 vaccine hesitancy from this study with results from 2019 Global Monitor for 45 countries with observations in both studies (31). Overall, the levels of COVID-19 vaccine hesitancy reported here are higher than concerns about vaccines in general (supplemental figure S6). However, there are also similarities in the pattern: countries in Europe and Central Asia and some Sub-Sahara African countries reported the highest levels of vaccine hesitancy even before the pandemic, and the biggest reason for pre-COVID-19 vaccine hesitancy was concerns about safety.

Nevertheless, the higher levels of hesitancy reported for the COVID-19 vaccine suggest that respondents are more concerned about the COVID-19 vaccine than about childhood vaccines. The result is even more striking considering that the COVID-19 vaccine hesitancy is estimated rather conservatively in the HFPS, when compared against estimates from other sources. Possible reasons for the higher rates of COVID-19 vaccine hesitancy include the newness of a vaccine that employs an innovative technology (mRNA), it's rapid development and streamlined approval process, and unknown long-term effects. This is consistent with the reasons given by respondents, which are mainly around safety.

Our findings that women, younger adults, and those with less education are more vaccine hesitant are largely consistent with results presented elsewhere (15, 19). Although Lazarus and colleagues found that men were slightly more hesitant than women, the gender difference was small (14). The findings with respect to age were also similar (14-15). The low levels of vaccine hesitancy reported among the older adults provide an important opportunity for vaccine campaigns to target this demographic, especially given recent research that targeting vaccines to older age groups saves the most lives and is highly cost-effective (32-33). The findings on education are also consistent with other studies (14-15), although Solis-Arce and colleagues reported mixed results (19). Knowing that citizens who are younger and with less formal education are the most likely to be vaccine hesitant can help vaccination campaigns target these population groups. Although we hypothesized that confidence in the government in general would be negatively associated with vaccine hesitancy, this association was not statistically significant in the regression analysis. This is likely because trust indicators were only available at the national level, making the estimates imprecise. Previous studies have shown mixed results (14-15, 18, 21), and future analysis is needed to investigate this relationship, preferably using an individual level measure of trust in government.

Our results showed that changes over time were minimal in all countries except Iraq, Malawi, and Uzbekistan, where we report relatively large increases. It is difficult to identify the factors that led to increased hesitancy in these cases. The larger body on changes over time in vaccine hesitancy during the same timeframe as the HFPS data were collected is mostly from high-income countries and showed mixed results (34-38). Furthermore, this period was relatively short.

The main reasons reported in the HFPS regarding COVID-19 vaccine hesitancy, which mainly revolve around safety and to a lesser degree efficacy suggest that factors in the 'thinking and feeling' domain of the BeSD framework were predominant (8). This is consistent with the few studies that report reasons for COVID-19 vaccine hesitancy in low- and middle-income countries and with the main reasons for vaccine hesitancy historically (16, 19, 31).

The variation in COVID-19 vaccine hesitancy across regions, and especially in Eastern Europe and Central Asia where rates are highest, is not fully explained by results in this study. Self-reported reasons for being hesitant were only available for one country in Eastern Europe and Central Asia (Georgia). Although our findings from Georgia that counterindication was a common barrier to vaccination are consistent with results published elsewhere (39), more research is needed to understand differences in vaccine hesitancy across regions.

Although concerns have been raised about supply constraints being the greatest barrier to COVID-19 vaccination scale-up in developing countries, our study did not find this to be a major concern for most respondents. However, with only 1 in 5 individuals in low-income countries vaccinated to date, there was still a large share of the population who would get vaccinated if vaccines were made available to them, and alleviating access constraints will remain a policy priority in such contexts.

For peer review only

4c. Limitations of the study

The high-frequency surveys used in this analysis were designed to be nationally representative; however, there are potential limitations to phone surveys (40-41). Phone surveys exclude respondents without access to a phone. This method of data collection was necessary to collect information quickly during the early months of the COVID-19 pandemic while respecting local movement restrictions and minimizing the risk of COVID-19 transmission. Given that having a phone may be non-random, this might have created a risk that the results are representative only of the population with access to a phone. However, in the countries in this sample, access to mobile phones was high, and in countries where the sample was based on an existing nationally representative (pre-pandemic) survey, sampling weights were used in the analysis to correct for the biases resulting from non-random access to phones. Studies using phone survey from Africa also included in our sample have shown that although phone survey respondents tended to be older and better educated, the weighting procedures successfully minimized the selection bias in the phone surveys for a wide range of indicators (42-43). Another study on labor market participation during COVID-19 using the same phone survey data found that reweighting did not alter the main results (44).

Another limitation is that country surveys were collected at different times, and some surveys were collected before a vaccine was available in the country while others were collected after the vaccine was available. During data collection (and the pandemic more generally), influential events occurred, including adverse news about side effects of certain vaccines, which could have changed respondents' intentions. Furthermore, there are multiple vaccine manufacturers and types, and the type of vaccine available or anticipated to become available in each country may influence levels of hesitancy.

5. Conclusions

Between late 2020 and the first half of 2021, the level of COVID-19 vaccine hesitancy was on average about 20 percent across 53 developing countries. Although less prevalent than previously thought, COVID-19 vaccine hesitancy was higher than levels of hesitancy reported towards other vaccines, indicating the challenges in scaling up COVID-19 vaccination campaigns may be even greater than for other diseases.

This study provides a rich set of results which can aid policy makers in the design of vaccination campaigns in developing countries. Overall, knowing that hesitancy is less prevalent than previous estimates may bolster the case for expanding vaccination interventions. Specifically, knowing that people over the age of 65 years are the least likely to be hesitant provides an important rationale for scaling up vaccine rollout in this population group, which is at highest risk of severe disease and mortality from COVID-19. In many countries, a sizeable share of the hesitant reported being "not sure" about getting vaccinating (rather than definitely not willing), likely providing greater opportunity to influence vaccination decisions in these countries. In addition, the findings suggest the importance of designing vaccination campaigns that address concerns about safety, especially about side-effects and effectively reach the most hesitant, including women, younger adults, and less educated adults.

For peer review only

Contributors All authors had full access to all the data in the study and take responsibility for the integrity of the data and the accuracy of the data analysis. All authors were responsible for its conception and design. IE, AC and GD were responsible for the preparation and analysis of the data and JD, DN, AC and YK contributed to their interpretation. JD drafted the manuscript, and AC, DN and YK made critical revision of the manuscript for important intellectual content. The corresponding author attests that all listed authors meet authorship criteria and that no others meeting criteria have been omitted.

Competing interests None declared.

Funding. This paper was funded by the World Bank (Project AA-P177656-GPLG-BB) and the Bill and Melinda Gates Foundation through the Umbrella Facility for Poverty and Equity (TF0B7242), with support from the Global Financing Facility for Women, Children, and Adolescents (GFF).

Ethics approval statement: This study involves human participants. Each phone survey was implemented by the respective national statistical office or, in a few cases, a private firm. Informed consent was received from all survey respondents in each country. The World Bank does not require institutional ethics approval for household surveys that are partly or fully financed by the World Bank, including these national phone surveys that inform our research.

Data availability statement Data for most countries are available in a public, open access repository available from the World Bank's Microdata Library, High Frequency Phone Survey Catalog (https://microdata. Worldbank.org/index.php/catalog/hfps).

For peer review only

References

(1) Dong E, Du H, Gardner L. An interactive web-based dashboard to track COVID-19 in real time. Lancet Infect Dis 2020; 20(5): 533-4. https://coronavirus.jhu.edu/ accessed March 1, 2022.

(2) World Bank. 2022. Global Economic Prospects, January 2022. Washington, DC: World Bank. Doi: 10.1596/978-1-4648-1758-8

(3) International Monetary Fund. 2021. World Economic Outlook Update. July 2021. Fault Lines Widen in the Global Recovery.

(4) Hoogeveen, JG and G. Lopez-Acevedo, eds. 2021. Distributional Impacts of COVID-19 in the Middle East and North Africa Region. MENA Development Report;. Washington, DC: World Bank. © World Bank. https://openknowledge.worldbank.org/handle/10986/36618 License: CC BY 3.0 IGO.

(5) Ritchie, H., E. Mathieu, L. Rodés-Guirao, et al.2020. – "Coronavirus Pandemic (COVID-19)". Published online at OurWorldInData.org. Retrieved from: 'https://ourworldindata.org/coronavirus' on March 8, 2022.

(6) MacDonald, NE, and SAGE Working Group on Vaccine Hesitancy. Vaccine hesitancy: Definition, Scope, and determinants. Vaccine. Volume 33, Issue 34, 2015, Pages 4161-4164 https://doi.org/10.1016/j.vaccine.2015.04.036

(7) World Health Organization. 2019. Ten threats to global health in 2019. https://www.who.int/newsroom/spotlight/ten-threats-to-global-health-in-2019.

(8) Brewer, N.T., Chapman, G.B., Rothman, A.J., Leask, J. and Kempe, A., 2017. Increasing vaccination: putting psychological science into action. *Psychological Science in the Public Interest*, *18*(3), pp.149-207.

(9) Gramacho WG, Turgeon M. 2021. When politics collides with public health: COVID-19 vaccine country of origin and vaccination acceptance in Brazil. Vaccine. May 6;39(19):2608-2612. Doi: 10.1016/j.vaccine.2021.03.080.

(10) Bucci EM, Berkhof J, Gillibert A, Gopalakrishna G, Calogero RA, Bouter LM, Andreev K, Naudet F, Vlassov V. Data discrepancies and substandard reporting of interim data of Sputnik V phase 3 trial. Lancet. 2021 May 22;397(10288):1881-1883.

(11) Wong, L.P., Alias, H., Danaee, M., Ahmed, J., Lachyan, A., Cai, C.Z., Lin, Y., Hu, Z., Tan, S.Y., Lu, Y. and Cai, G., 2021. COVID-19 vaccination intention and vaccine characteristics influencing vaccination acceptance: a global survey of 17 countries. Infectious diseases of poverty, 10(1), pp.1-14.

(12) Husby, A. and Køber, L., 2022. COVID-19 mRNA vaccination and myocarditis or pericarditis. The Lancet, 399(10342), pp.2168-2169.

(13) Robinson, E., Jones, A., Lesser, I. and Daly, M., 2021. International estimates of intended uptake and refusal of COVID-19 vaccines: A rapid systematic review and meta-analysis of large nationally representative samples. Vaccine.

(14) Lazarus, JV, Ratzan SC , and Palayew A et al. 2021. A global survey of potential acceptance of a COVID-19 vaccine. Brief Communication. Nature Medicine 27: 225–228. https://doi.org/10.1038/s41591-020-1124-9.

(15) de Figueiredo A, Larson HJ. Exploratory study of the global intent to accept COVID-19 vaccinations. Communications medicine. 2021 Sep 9;1(1):1-0.

(16) Facebook. 2021. COVID-19 Trends and Impact Survey.

(17) Gallup. 2021. Gallup World Poll. https://www.gallup.com/178667/gallup-world-poll-work.aspx.
(18) Kanyanda, S., Markhof, Y., Wollburg, P. and Zezza, A., 2021. Acceptance of COVID-19 vaccines in sub-Saharan Africa: Evidence from six national phone surveys. BMJ open, 11(12), p.e055159.
(19) Solís Arce, J.S., Warren, S.S., Meriggi, N.F. et al. COVID-19 vaccine acceptance and hesitancy in low- and middle-income countries. Nat Med **27,** 1385–1394 (2021). https://doi.org/10.1038/s41591-021-01454-y.
(20) Wouters, O.J., Shadlen, K.C., Salcher-Konrad, M., Pollard, A.J., Larson, H.J., Teerawattananon, Y. and Jit, M., 2021. Challenges in ensuring global access to COVID-19 vaccines: production, affordability, allocation, and deployment. The Lancet, 397(10278), pp.1023-1034.
(21) Sallam M. COVID-19 Vaccine Hesitancy Worldwide: A Concise Systematic Review of Vaccine Acceptance Rates. Vaccines (Basel). 2021 Feb 16;9(2):160. Doi: 10.3390/vaccines9020160. PMID: 33669441; PMCID: PMC7920465.
(22) World Bank. 2020. High Frequency Mobile Phone Surveys of Households to Assess the Impacts of COVID-19: Overview (English). Washington, D.C.: World Bank Group. http://documents.worldbank.org/curated/en/703571588695361920/Overview
(23) [dataset] World Bank. 2020. High Frequency Mobile Phone Surveys Microdata. https://microdata.worldbank.org/index.php/catalog/hfps/?page=1&ps=15&repo=hfps.
(24) [dataset] Hale T, Angrist N, Goldszmidt R, et al. 2022. A global panel database of pandemic policies (Oxford COVID-19 government response Tracker). Nat Hum Behav 2021;5:529–38. Database accessed Feb 28, 2022.
(25) [dataset] Inglehart, R, C. Haerpfer, A. Moreno et al. 2022. Would Values Survey: Round Seven – Country Pooled Datafile Version: https://www.worldvaluessurvey.org/WVSOnline.jsp. (accessed April 21, 2022) Madrid: JD Systems Institute.
(26) [dataset] World Health Organization. 2022. Global Excess Deaths Associated with COVID-19 (Modelled Estimates). Downloaded on September 8, 2022 at https://www.who.int/data/sets/global-excess-deaths-associated-with-covid-19-modelled-estimates.
(27) Africa CDC. 2021. COVID-19 vaccine perceptions: a 15-country study, 2021. Available: https://africacdc.org/download/covid-19-vaccineperceptions-a-15-country-study/ [Accessed 18 May 2021].
(28) Anjorin, A.A., Odetokun, I.A., Abioye, A.I., Elnadi, H., Umoren, M.V., Damaris, B.F., Eyedo, J., Umar, H.I., Nyandwi, J.B., Abdalla, M.M. and Tijani, S.O., 2021. Will Africans take COVID-19 vaccination? PloS one, 16(12), p.e0260575.
(29) Faezi, N.A., Gholizadeh, P., Sanogo, M., Oumarou, A., Mohamed, M.N., Cissoko, Y., Sow, M.S., Keita, B.S., Baye, Y.A.M., Pagliano, P. and Akouda, P., 2021. Peoples' attitude toward COVID-19 vaccine, acceptance, and social trust among African and Middle East countries. Health Promotion Perspectives, 11(2), p.171.
(30) Bono, S.A., Faria de Moura Villela, E., Siau, C.S., Chen, W.S., Pengpid, S., Hasan, M.T., Sessou, P., Ditekemena, J.D., Amodan, B.O., Hosseinipour, M.C. and Dolo, H., 2021. Factors affecting COVID-19 vaccine acceptance: an international survey among low-and middle-income countries. Vaccines, 9(5), p.515.Gallup. 2019. Wellcome Global Monitor – First Wave Findings. https://wellcome.org/reports/wellcome-global-monitor/2018#downloads-4d1c.

For peer review only

(31) Gallup. 2019. Wellcome Global Monitor – First Wave Findings. https://wellcome.org/reports/wellcomeglobal-monitor/2018#downloads-4d1c.

(32) Cheikh, N., J. Spitz and D. Wilson. 2022. Increase Vaccine Targeting and Uptake: lessons learned from Global COVID-19 Vaccine Implementation. Considerations for World Bank Task Teams Managing COVID-19 Fast Track Facility Operations. Washington, D.C.: World Bank Group.

(33) Orangi S, Ojal J, Brand SP, et al. 2022. Epidemiological impact and cost-effectiveness analysis of COVID-19 vaccination in Kenya. BMJ Global Health **7:**e009430.

(34) To QG, Stanton R, Khalesi S, Williams SL, Alley SJ, Thwaite TL, Fenning AS, Vandelanotte C. Willingness to Vaccinate against COVID-19 Declines in Australia, Except in Lockdown Areas. Vaccines (Basel). 2021 May 10;9(5):479. doi: 10.3390/vaccines9050479. PMID: 34068479; PMCID: PMC8150580.

(35) Lavoie K, Gosselin-Boucher V, Stojanovic J, Gupta S, Gagné M, Joyal-Desmarais K, Séguin K, Gorin SS, Ribeiro P, Voisard B, Vallis M, Corace K, Presseau J, Bacon S; iCARE Study Team. Understanding national trends in COVID-19 vaccine hesitancy in Canada: results from five sequential cross-sectional representative surveys spanning April 2020-March 2021. BMJ Open. 2022 Apr 5;12(4):e059411. doi: 10.1136/bmjopen-2021-059411. PMID: 35383087; PMCID: PMC8983402.

(36) Szilagyi PG, Thomas K, Shah MD, Vizueta N, Cui Y, Vangala S, Kapteyn A. National Trends in the US Public's Likelihood of Getting a COVID-19 Vaccine-April 1 to December 8, 2020. JAMA. 2020 Dec 29;325(4):396–8. doi: 10.1001/jama.2020.26419. Epub ahead of print. PMID: 33372943; PMCID: PMC7772743.

(37) Biasio LR, Bonaccorsi G, Lorini C, Mazzini D, Pecorelli S. Italian Adults' Likelihood of Getting COVID-19 Vaccine: A Second Online Survey. Vaccines (Basel). 2021 Mar 17;9(3):268. doi: 10.3390/vaccines9030268. PMID: 33802873; PMCID: PMC8002675.

(38) Sypsa V, Roussos S, Engeli V, Paraskevis D, Tsiodras S, Hatzakis A. Trends in COVID-19 Vaccination Intent, Determinants and Reasons for Vaccine Hesitancy: Results from Repeated Cross-Sectional Surveys in the Adult General Population of Greece during November 2020-June 2021. Vaccines (Basel). 2022 Mar 18;10(3):470. doi: 10.3390/vaccines10030470. PMID: 35335102; PMCID: PMC8950863.

(39) World Bank, UNIFEF and JICA 2021. Counterindication: now the key driver of COVID-19 vaccine hesitancy in Central Asia.

(40) Gourlay, S., Kilic, T., Martuscelli, A., Wollburg, P. and Zezza, A., 2021. High-frequency phone surveys on COVID-19: good practices, open questions. *Food Policy*, *105*, p.102153.

(41) Bundervoet, T., Dávalos, M.E. and Garcia, N., 2022. The short-term impacts of COVID-19 on households in developing countries: An overview based on a harmonized dataset of high-frequency surveys. *World development*, p.105844.

(42) Brubaker J, Kilic T, Wollburg P. 2021. Representativeness of individual-level data in COVID-19 phone surveys: Findings from SubSaharan Africa. PLoS ONE 16(11): e0258877. https://doi.org/10.1371/journal.pone.0258877

(43) Ambel AA, Mcgee KR, Tsegay AH. 2021. Reducing Bias in Phone Survey Samples: Effectiveness of Reweighting Techniques using Face-to-Face Surveys as Frames. World Bank: Washington, DC.

For peer review only - http://bmjopen.bmj.com/site/about/guidelines.xhtml

(44) Kugler, M., Viollaz, M., Duque, D., Gaddis, I., Newhouse, D.L., Palacios-Lopez, A. and Weber, M., 2021. How Did the COVID-19 Crisis Affect Different Types of Workers in the Developing World?. IZA Discussion Paper No. 14519, Available at SSRN: https://ssrn.com/abstract=3883795 or http://dx.doi.org/10.2139/ssrn.3883795

For peer review only

Figure Legends and Notes

Figure 1

Title: Share of households that were hesitant to be vaccinated against COVID-19 in 53 countries

Notes: Weighted estimates, with weights scales such that 53 countries are given equal weights, except for results by education which include only results from the 33 country surveys with information on the educational attainment of the respondent.

Figure 2

Title: Share of households that were hesitant to be vaccinated against COVID-19 (answering either “no” or “not sure”), by country

Notes: Weighted estimates, with weights scales such that 53 countries are given equal weights. Black bars indicate confidence intervals. Point estimates are given in supplemental table S3.

Source: Authors’ calculations.

Figure 3

Title: Changes in levels of vaccine hesitancy in 14 countries, October 2020 to April 2021

Note: The shaded area around the line represents the confidence bands.

Source: Authors’ calculations.

Figure 4

Title: Self-reported reason for vaccine hesitancy in countries with single answer response, HFPS

Notes: The X axis represents share of vaccine hesitant households. In some cases, the share is larger than 100% because multiple responses were allowed. For the construction of harmonized response categories, see supplemental table S2b. Results for countries will multiple answer options are shown in supplemental figure S4.

Source: Authors’ calculations.

For peer review only

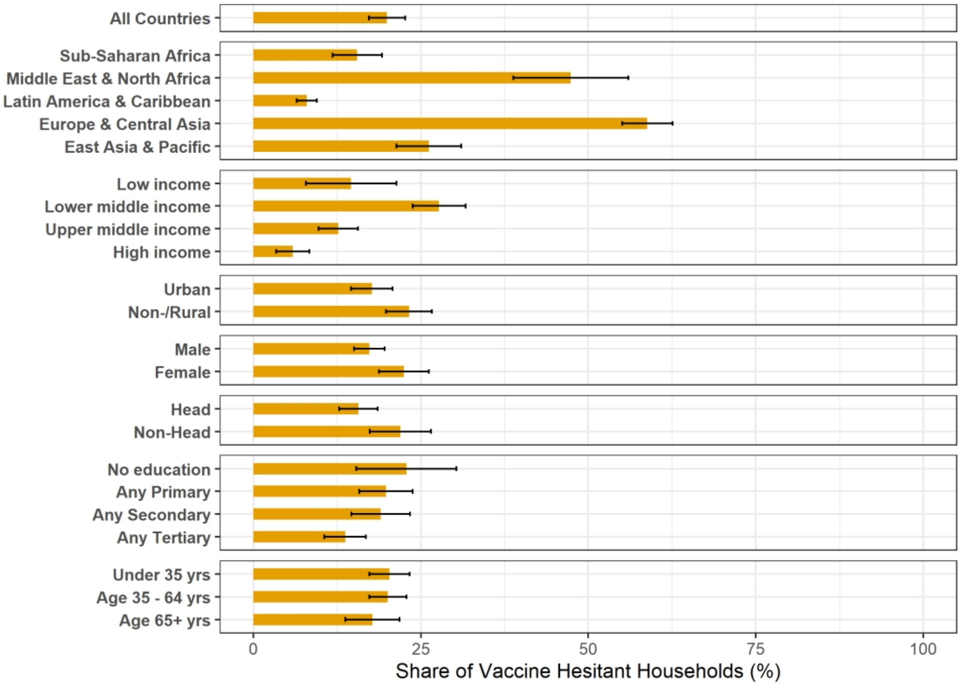

Figure 1: Share of households that were hesitant to be vaccinated against COVID-19 in 53 countries

490x342mm (118 x 118 DPI)

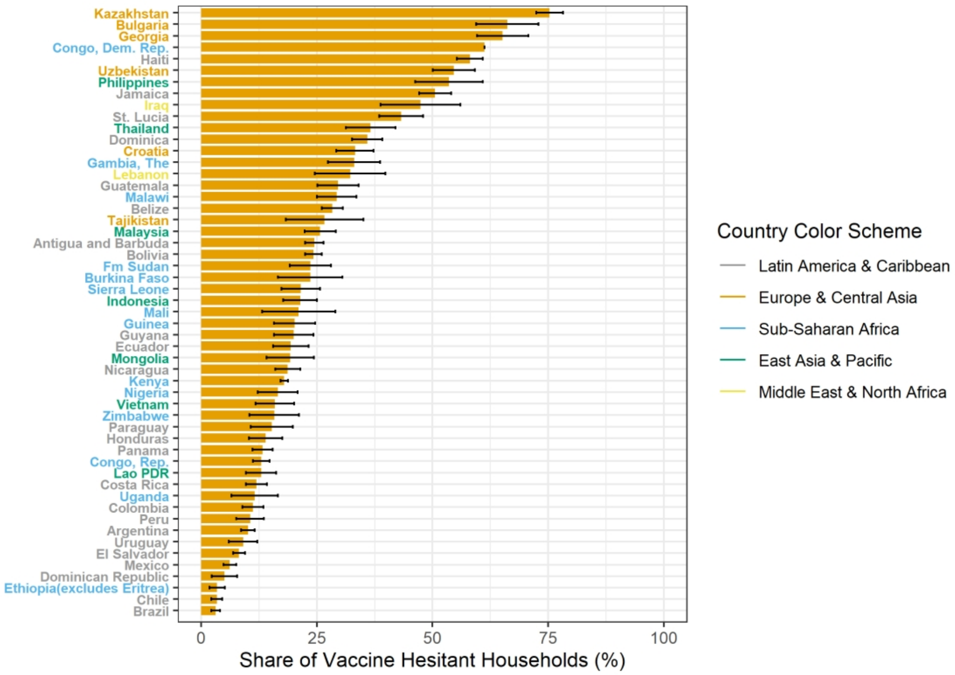

Share of households that were hesitant to be vaccinated against COVID-19 (answering either “no” or “not sure”), by country

490x336mm (118 x 118 DPI)

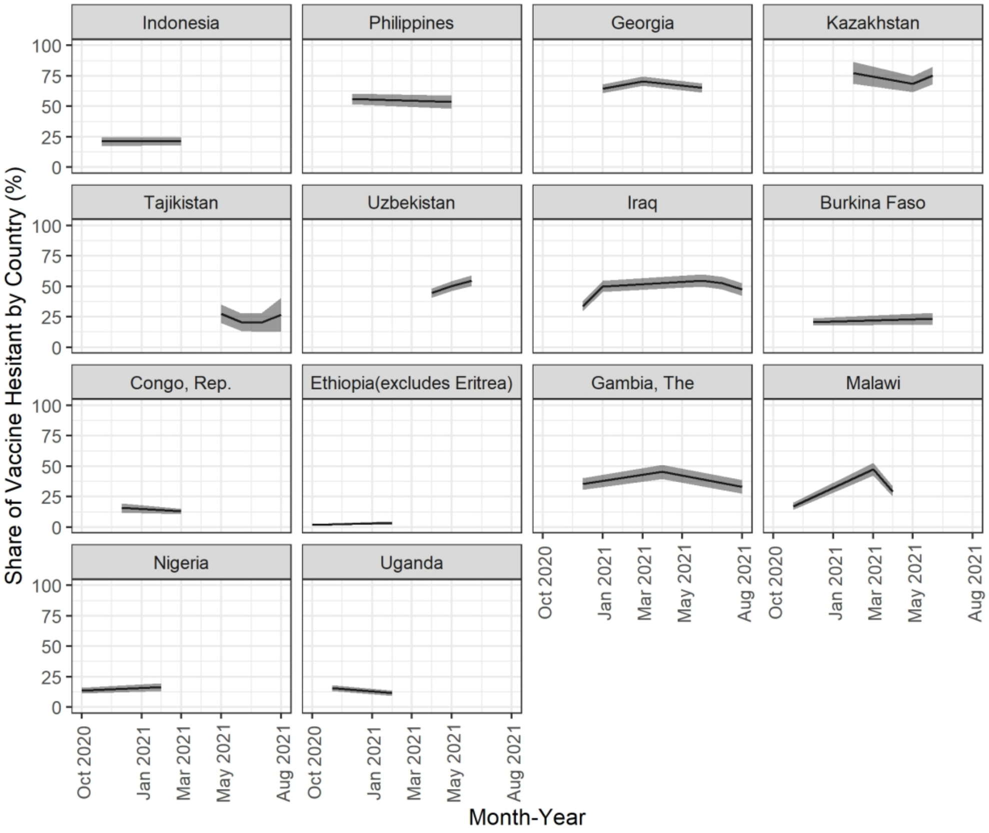

Figure 3: Changes in levels of vaccine hesitancy in 14 countries, October 2020 to April 2021

441x373mm (118 x 118 DPI)

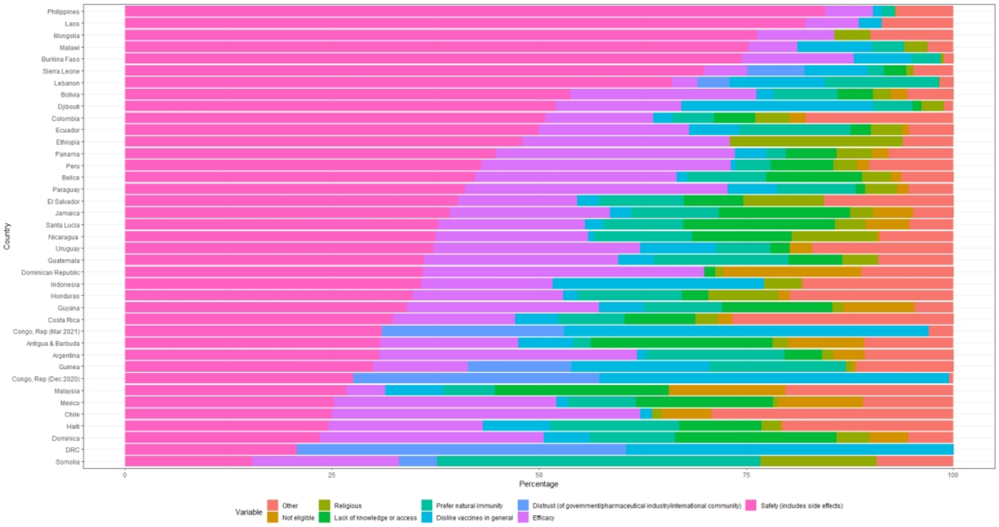

Self-reported reason for vaccine hesitancy in countries with single answer response, HFPS

1283x679mm (38 x 38 DPI)

Supplemental materials, COVID-19 Vaccine Hesitancy in 53 Countries

1. **Supplements**

Supplement 1: Detailed Description of Methods

2. **Supplemental Tables**

Supplemental Table S1: Description of 53 HFPS surveys

Supplemental Table S2a: Survey response options for COVID-19 vaccine hesitancy: distribution of countries by questionnaire type

Supplemental Table S2b: Survey response options for COVID-19 vaccine hesitancy: Harmonized answers by questionnaire type

Supplemental Table S3: Percent of population hesitant to take the COVID-19 vaccine, HFPS

Supplemental Table S4: Tukey's tests of significance of differences across groups in percent of population hesitant to take the COVID-19 vaccine

Supplemental Table S5a: Correlates of vaccine hesitancy (Logit model, marginal effects)

Supplemental Table S5b: Correlates of vaccine hesitancy (Ordinal logit regression, marginal effects)

Supplemental Table S6: Percent of population hesitant to take the COVID-19 vaccine, 14 countries with multiple rounds of survey results (95% confidence intervals in parentheses).

Supplemental Table S7: Comparison of results on vaccine hesitancy from the HFPS and other studies

3. **Supplemental Figures**

Supplemental Figure S1: Vaccination rates by income group

Supplemental Figure S2: Vaccine rollout rates by income group

Supplemental Figure S3: Strength of sentiment of COVID-19 vaccine hesitancy: share of households in 46 countries reporting "yes," "not sure," or "no" when asked if they will take the COVID-19 vaccination when available

Supplemental Figure S4: Self-reported reason for vaccine hesitancy in countries with multiple answer options, HFPS

Supplemental Figure S5: Comparison of share of population COVID-19 that is hesitant to take the COVID-19 vaccine, HFPS with other studies

Supplemental Figure S6: Comparison of COVID-19 Vaccine Hesitancy with pre-COVID sentiment about vaccines from 2019 Global Monitor, 45 countries

Supplemental Figure S7: Reason for "definitely not" (upper panel) or "probably not" (lower panel) taking the COVID-19 vaccine, Facebook data for month of HFPS)

4. **References**

For peer review only

Supplement 1: Detailed Description of Methods

Data

This analysis describes the levels of vaccine hesitancy and its reasons in 53 developing countries between October 2020 August 2021 using data from the World Bank's COVID-19 (HFPS) which were implemented to monitor the impact of COVID-19 on households around the world (World Bank 2020). Data are available for one survey round from 39 countries and two or more survey rounds for 14 countries. The countries in the pooled database represent five out of six regions defined by the World Bank: Latin America and the Caribbean (24 countries), Sub-Saharan Africa (14), East Asia and Pacific (7), Europe and Central Asia (6) and the Middle East and North Africa (2).[1] By income group, the sample includes 12 low-income countries, 13 lower-middle income countries, 23 upper middle-income countries and five high-income countries (Supplemental Table 1 provides a list of countries with survey month, sample size, region and country income group).

A global core questionnaire served as the basis for each country's survey but was then customized to fit the local context. As a result, the exact questions may vary across countries, but topics typically included knowledge and concerns about COVID-19, access to food, health care and education, employment and income loss, and safety nets and coping strategies. The survey data was then harmonized ex-post to enable cross-country comparability. The questionnaire was flexible and adapted over time to the pandemic context. The vaccine module, for example, was included more recently.

The sampling frame was drawn from pre-existing nationally representative household surveys in 19 countries, random digit dialing (RDD) in 29 countries, and a list of phone numbers typically obtained from mobile phone operators in 5 countries. The RDD design samples from all active mobile and landline phone numbers, such that RDD surveys are representative of the population aged 18 and over with an active phone number, conditional on response and survey completion. Sampling weights were then constructed to correct for selection bias due to the inability to contact households that did not participate in the survey either due to non-response or lack of access to a phone, with the goal of obtaining estimates that are as close to being nationally representative as possible. Information was collected from one respondent per household. In the case of countries where the sampling frame was derived from a previous survey, this was typically the household head. In other cases, such as when the sample utilized RDD, the respondent was more representative of individuals within the household. While the non-random selection of individuals in the former cases is not ideal, in an earlier analysis of labor market indicators derived from phone surveys, Kugler et al. (2021) find little evidence of differences in employment outcomes or trends over time being affected by the oversampling of household heads with respect to variables such as level of education, gender or location, the main source of bias being for age comparisons. Within the pooled data set, the household sampling weights are scaled such that countries are weighted equally, such that estimates of vaccine hesitancy reported in this study are averages of country averages.[2]

Outcome measures

The questions asked about vaccine hesitancy in the surveys varied depending on whether a vaccine was available in the country at the time of the survey. In surveys that took place before the vaccine rollout had begun in that country, the question asked was "*If an approved vaccine to prevent COVID-19 was to become*

[1] South Asia is not represented due to lack of available data. The Middle East and North Africa includes two countries—Iraq and Lebanon.

[2] Using population weights would have resulted in the results being driven by a small number of large countries.

available at no cost, would you agree to be vaccinated?" In surveys that took place after the vaccine rollout had begun, the wording of the question was either "*Are you planning to be vaccinated?*" or "*When a vaccine to prevent COVID-19 is available to you, are you planning to be vaccinated?"* In the sample, 47 countries offered three answer options (yes, not sure, and no), while the other six offered only two categories (yes and no). For this analysis, we combine the "no" and "not sure" answers to obtain the measure of vaccine hesitancy.

To obtain the respondent's reason for vaccine hesitancy, survey respondents who answered "no" or "not sure" were asked "*What is your (main) reason/concern for not wanting to be vaccinated / not being sure if you want to be vaccinated*?" The answer categories varied widely across surveys. To make these more comparable across countries, answers were remapped into the following nine most common categories (see Supplemental Tables 2a and 2b for the original answer options in each survey and a mapping of how they were harmonized):

1. Safety, which includes concerns about side effects
2. Efficacy
3. Distrust of government, pharmaceutical industry, international community
4. Dislike of vaccines in general
5. Preference for natural immunity, which included perceiving self as low risk and already having had COVID-19 infection
6. Lack of knowledge or access, which included "I do not have enough information about the vaccine," "too hard to get," "health facility to far" and "I don't have the time"
7. Religious reasons
8. Not eligible, which included "counter-indication," "recent medical discharge, "have an underlying health condition and believe taking the vaccine will make it worse"
9. Other

The surveys also differed across countries in terms of whether a single concern or multiple concerns were collected. To account for the difference in the number of response options, the results are presented separately.

Contextual data

Country-level contextual data were drawn from other sources. New COVID-19 cases per million, measured as a 7-day rolling average prior to the midpoint of the month household survey data was collected in each country, were obtained from Dong and colleagues (2022), and the Oxford Stringency Index was drawn from Hale et al. 2022. The latter is an index, ranging from 0–100, that indicates the degree to which various restrictions were put in place by governments to control the pandemic (for example, school closures and shelter-in-place requirements). Confidence in the press, the government, and the WHO was drawn from the World Values Survey Wave 7, fielded in 2017-2020, and aggregated up to the country level (Inglehart 2022). Excess deaths due to COVID-19 draw on WHO data (WHO 2022). Country geographic region and income group are based on the World Bank classifications.

Analytical strategy

Data from 53 countries was pooled into a single data set. First, we report point estimates and 95% confidence intervals for each country based on standard errors clustered at the state or province level within each country. We also report the simple average across all countries and stratified by: (i) World Bank region, (ii) World Bank country income group, (iii) urban vs. rural residence, (iv) gender of the respondent, (v) whether the respondent is head of household, (vi) age of the respondent (under 35 years,

For peer review only

35-64 years, and 65 years and older, and (vii) educational attainment of the respondent (some primary school, some secondary school, some tertiary). Tukey's test of multiple comparisons is used to test the significance of differences in rates of vaccine hesitancy across the above-mentioned groups. Second, we use multivariate regression analysis to assess the relative association of vaccine hesitancy and the correlates and contextual variables. Trends over time in levels of vaccine hesitancy are examined in 14 countries with more than one wave of results available. Finally, the analysis describes the reasons for vaccine hesitancy.

For peer review only

Supplemental Table S1: Description of 53 HFPS surveys

Country	Survey Months	Total Number of households	Sample frame
**Antigua and Barbuda**	06/2021	790	RDD
**Argentina**	06/2021	1216	RDD
**Belize**	06/2021	816	RDD
**Bolivia**	05/2021	1272	RDD
**Brazil**	08/2021	2166	RDD
**Bulgaria**	07/2021	1000	RDD
**Burkina Faso**	12/2020	1944	Previous survey
**Chile**	06/2021	1212	RDD
**Colombia**	06/2021	1221	RDD
**Congo, Dem. Rep.**	12/2020	986	Previous Survey
**Congo, Rep.**	12/2020, 03/2021	578, 1495	Previous Survey
**Costa Rica**	06/2021	802	RDD
**Croatia**	03/2021	1217	Non-survey list
**Dominica**	06/2021	861	RDD
**Dominican Republic**	06/2021	1205	RDD
**Ecuador**	05/2021	1352	RDD
**El Salvador**	06/2021	816	RDD
**Ethiopia**	10/2020, 02/2021	2704, 2178	Previous Survey
**The Gambia**	12/2020, 04/2021, 08/2021	1334, 1287, 1059	Previous Survey
**Georgia**	01/2021, 03/2021, 06/2021	2033, 2100, 1936	RDD
**Guinea**	11/2020	1334	Previous Survey
**Guatemala**	06/2021	1206	RDD
**Guyana**	06/2021	785	RDD
**Haiti**	07/2021	2813	Non-Survey list
**Honduras**	07/2021	1021	RDD
**Indonesia**	11/2020, 03/2021	3953, 3555	Previous Survey
**Iraq**	12/2020, 01/2021,06/2021, 07/2021, 08/2021	1614,1651,1378, 1297,1141	Non-Survey list
**Jamaica**	06/2021	828	RDD
**Kazakhstan**	02/2021, 05/2021, 06/2021	917, 1732, 1610	Previous Survey
**Kenya**	03/2021	6730	Previous Survey

For peer review only

Country	Survey Months	Total Number of households	Sample frame
**Lao PDR**	03/2021	2153	RDD
**Lebanon**	03/2021	5113	RDD
**Mali**	01/2021	1884	Previous Survey
**Malawi**	11/2020, 03/2021, 04/2021	1589, 1549,1338	Previous Survey
**Malaysia**	06/2021	2210	RDD
**Mexico**	06/2021	2624	RDD
**Mongolia**	12/2020	1147	Previous Survey
**Nicaragua**	06/2021	833	RDD
**Nigeria**	02/2021, 10/2020	1699, 1762	Previous Survey
**Panama**	06/2021	815	RDD
**Paraguay**	06/2021	1076	RDD
**Peru**	06/2021	1210	RDD
**Philippines**	12/2020, 05/2021	1805, 2122	Non-survey list
**Saint Lucia**	06/2021	835	RDD
**Sierra Leone**	11/2020	1198	Previous Survey
**Sudan**	03/2021	2545	Non-Survey list
**Tajikistan**	05-08/2021	232	Previous Survey
**Thailand**	05/2021	1786	RDD
**Uganda**	11/2020, 02/2021	2135, 2121	Previous Survey
**Uruguay**	06/2021	816	RDD
**Uzbekistan**	04-06/2021	1496, 1356, 1300	Previous Survey
**Vietnam**	01/2021	3940	Previous Survey
**Zimbabwe**	12/2020	1227	Previous Survey

Source: Authors' calculations.

For peer review only

Supplemental Table S2a: Survey response options for COVID-19 vaccine hesitancy: distribution of countries by questionnaire type

**Questionnaire type 1**	**Questionnaire type 2**	**Questionnaire type 3**	**Questionnaire type 4**	**Questionnaire type 5**	**Questionnaire type 6**	**Questionnaire type 7**	**Questionnaire type 8**	**Questionnaire type 9**	**Questionnaire type 10**	**Questionnaire LAC**	**Questionnaire type 11**	**Questionnaire type 12**
Kenya	*Philippines*	*Lebanon*	*Congo, Democratic Republic*	*Guinea*	*Malaysia*	*Mali*	*Gambia, The*	*Lao PDR*	*Cambodia*	*Argentina*	*Kazakhstan*	*Croatia*
Thailand	*Indonesia*		*Republic of Congo*	*Sudan*		*Burkina Faso*		*Mongolia*		*Antigua and Barbuda*	*Kyrgyzstan*	
	Djibouti			*Sierra Leone*		*Uganda*				*Belize*	*Uzbekistan*	
						Ethiopia				*Bolivia*		
						Malawi				*Brazil*		
						Nigeria				*Chile*		
										Colombia		
										Costa Rica		
										Dominica		
										Dominican Republic		
										Ecuador		
										Guatemala		
										Guyana		
										Honduras		
										Haiti		
										Jamaica		
										Mexico		
										Nicaragua		
										Panama		
										Peru		
										Paraguay		
										El Salvador		
										Uruguay		

For peer review only

For peer review only - http://bmjopen.bmj.com/site/about/guidelines.xhtml

Supplemental Table S2b: Survey response options for COVID-19 vaccine hesitancy: Harmonized answers by questionnaire type

Answer categories	Quest type 1	Quest type 2	Quest type 3	Quest type 4	Quest type 5	Quest type 6	Quest type 7	Quest type 8	Quest type 9	Quest type 10	Quest LAC	Quest type 11	Quest type 12
**1. Efficacy**	I don't think it will work	I don't think COVID-19 vaccines effective/work	I don't think coronavirus vaccines are effective/work		I don't think vaccines work	I don't think it will work	I don't think it will work	I do not think the vaccine would work against COVID	I don't think COVID-19 vaccines effective/work		I do not think they are effective, that they work		It may not work
						I don't think the vaccines available in my country are effective							
**2 Safety (includes side effects)**	I don't think it will be safe	I am worried about the safety of the vaccine		Safety of the vaccine	I am worried about the vaccine safety		I don't think it will be safe	I fear the unforeseen future negative side effects from the COVID-19 vaccine	I am worried about the safety of the vaccine	I don't think it is safe		The risk of vaccinating is higher than the benefits	Health risks
	I am worried about the side effects	I am worried about the side effects	I am worried about the side effects of the vaccine			I am worried about the side effects	I'm worried about side effects	I have heard the vaccine has negative side effects	I am worried about the side effects		I don't think it's safe, because of the side effects		
**3. Perceive self as low risk**	I am not enough at risk of contracting COVID-19	I am strong, I never got any disease in the past	I am not enough at risk of contracting coronavirus			I am not enough at risk of contracting COVID-19	I am not at enough risk of contracting covid-19	I believe I do not need to be vaccinated			I am not at risk of getting covid-19		I am not worried about COVID-19
**4.Dislike vaccines in general**	I am against vaccines in general	I am against vaccines in general	I am against vaccines in general	General distrust of vaccines	I don't trust the vaccines in general	In general, I don't trust vaccines	I am against the vaccine in general		I am against vaccines in general		I am against vaccines in general	Negative past experiences with vaccines	I am against vaccines in general
**5 Religious**	It is against my religion	Religious reasons	Religious reasons		I have religious reasons/concerns	It is against my religion.	It's against my religion		Religious reasons		Religious reasons	Personal or religious beliefs	
		I am concerned about its											

For peer review only

Answer categories	Quest type 1	Quest type 2	Quest type 3	Quest type 4	Quest type 5	Quest type 6	Quest type 7	Quest type 8	Quest type 9	Quest type 10	Quest LAC	Quest type 11	Quest type 12
		halal certification											
**6 Concerned about getting covid at facility**	I'm worried to get infected with COVID-19 at the health facility	I am worried of getting infected with COVID-19 at the health facility	I am worried of getting infected with coronavirus at the health facility			I am worried to get infected with COVID-19 at the health facility.			I am worried to get infected with COVID-19 at the health facility		I am concerned of being infected with covid-19 at the health center		
**7 Supply access barrier**	Health facility is too far or too hard to get to		Health facility too far or too hard to get to		It does not suit me (will be given too far or difficult to find)	Health facility too far or too hard to get to		There is shortage of vaccines in the country		I don't know how to access the vaccine	Health center too far or hard to reach		
	I don't have time to get vaccinated/ It will take too long to get vaccinated					It will take too long to get vaccinated/ I don't have time to get vaccinated		There is no vaccination center near my place		I am not the priority group to get the vaccine	I don't have time to go to get vaccinated		
**8 Prefer natural immunity (includes already had COVID-19)**			I already had coronavirus		I believe that the remedies natural or traditional	I already had COVID-19		I prefer to build immunity against COVID-19 naturally by having the disease			I already had covid-19 / I no longer need it		I already had COVID-19 and do''t need a vaccine
					It is better to leave nature take its course; the COVID symptoms are mostly light								

For peer review only

Answer categories	Quest type 1	Quest type 2	Quest type 3	Quest type 4	Quest type 5	Quest type 6	Quest type 7	Quest type 8	Quest type 9	Quest type 10	Quest LAC	Quest type 11	Quest type 12
**9 Distrust (of government/pharmaceutical industry/international community)**				Be wary of international community	I do''t trust the government/ to the organization who gives the vaccine			I do not trust pharmaceutical companies				Lack of trust to producers of vaccines	I do''t trust the pharmaceutical industry
								I do not trust the government				Lack of trust to heath system or healthcare providers	I do''t trust the government
								I do not trust the COVID-19 vaccines					
								I heard the vaccine is meant to control population growth					
								I do not believe in COVID-19					
**10 Not eligible / conterindication**								I have underlying health conditions and I believe taking the vaccine will make it worse		I am not eligible to get the vaccine			Counter-indication or prolonged medical discharge
**11 Lack of knowledge about COVID 19 Vaccine**								I do not have enough information about the vaccine					

For peer review only

**Answer categories**	**Quest type 1**	**Quest type 2**	**Quest type 3**	**Quest type 4**	**Quest type 5**	**Quest type 6**	**Quest type 7**	**Quest type 8**	**Quest type 9**	**Quest type 10**	**Quest LAC**	**Quest type 11**	**Quest type 12**
								I did not know that a vaccine exists against COVID-19					
**12 Other**	Other (specify)	Other (specify)	Some other reason	other	Others	Other (Specify)	other, specify)	Everyday stresses are overwhelming to think about getting vaccinated	Others (specify)	Other, specify	Other (specify)	Other	
		I will wait till more people are vaccinated											
		No one in my neighborhood got it				I am not sure I will get the vaccine I want							

Source: Authors' calculations.

For peer review only

Supplemental Table S3: Percent of population hesitant to take the COVID-19 vaccine, HFPS

Average across countries	Percent (%) hesitant to take the COVID-19 vaccine	Confidence Intervals
All countries	20.0	[17.24 , 22.67]
East Asia & Pacific	26.2	[21.38 , 31.02]
Europe & Central Asia	58.8	[55.04 , 62.56]
Latin America & Caribbean	8.0	[6.45 , 9.47]
Middle East & North Africa	47.4	[38.80 , 55.96]
Sub-Saharan Africa	15.5	[11.81 , 19.19]
Low income countries	14.6	[7.82 , 21.39]
Lower middle income countries	27.7	[23.78 , 31.65]
Upper middle income countries	12.7	[9.77 , 15.61]
High income countries	5.9	[3.40 , 8.36]
Urban	17.6	[14.60 , 20.75]
Rural	23.2	[19.83 , 26.64]
Male	17.3	[15.02 , 19.63]
Female	22.5	[18.77 , 26.21]
Head of household	10.8	[12.82 , 18.53]
Non-Head of household	9.4	[17.37 , 26.54]
No education	22.8	[15.36 , 30.31]
Any Primary	19.8	[15.80 , 23.78]
Any Secondary	19.0	[14.64 , 23.42]
Any Tertiary	13.7	[10.56 , 16.83]
Age 34 and younger	20.3	[17.29 , 23.34]
Age 35— 64	20.1	[17.34 , 22.84]
Ages 65 and older	17.8	[13.70 , 21.80]
Antigua and Barbuda	24.5	[22.51 , 26.49]
Argentina	10.1	[8.66 , 11.53]
Belize	28.4	[26.10 , 30.64]
Bolivia	24.3	[22.47 , 26.04]
Brazil	3.1	[2.15 , 4.04]
Bulgaria	66.2	[59.48 , 72.90]
Burkina Faso	23.6	[16.60 , 30.53]
Chile	3.4	[2.22 , 4.57]
Colombia	11.2	[8.91 , 13.51]
Congo, Dem. Rep.	61.2	[61.23 , 61.23]
Congo, Rep.	13.0	[11.20 , 14.79]

For peer review only

Average across countries	Percent (%) hesitant to take the COVID-19 vaccine	Confidence Intervals
Costa Rica	11.9	[9.66 , 14.22]
Croatia	33.2	[29.21 , 37.25]
Dominica	35.9	[32.63 , 39.14]
Dominican Republic	5.0	[2.25 , 7.81]
Ecuador	19.4	[15.54 , 23.21]
El Salvador	8.2	[6.91 , 9.44]
Ethiopia (excludes Eritrea)	3.5	[1.81 , 5.09]
Fm Sudan	23.7	[19.19 , 28.11]
Gambia, The	33.1	[27.44 , 38.70]
Georgia	65.2	[59.62 , 70.72]
Guatemala	29.6	[25.09 , 34.05]
Guinea	20.2	[15.74 , 24.61]
Guyana	20.0	[15.75 , 24.23]
Haiti	58.1	[55.31 , 60.86]
Honduras	13.9	[10.31 , 17.51]
Indonesia	21.4	[17.71 , 25.07]
Iraq	47.4	[38.81 , 55.99]
Jamaica	50.6	[47.11 , 54.03]
Kazakhstan	75.3	[72.39 , 78.18]
Kenya	17.9	[17.11 , 18.74]
Lao PDR	13.0	[9.70 , 16.25]
Lebanon	32.2	[24.56 , 39.84]
Malawi	29.3	[25.03 , 33.54]
Malaysia	25.7	[22.37 , 29.10]
Mali	21.1	[13.14 , 29.03]
Mexico	6.2	[4.85 , 7.55]
Mongolia	19.3	[14.13 , 24.38]
Nicaragua	18.7	[16.04 , 21.40]
Nigeria	16.6	[12.27 , 20.84]
Panama	13.3	[11.12 , 15.49]
Paraguay	15.3	[10.71 , 19.79]
Peru	10.6	[7.55 , 13.59]
Philippines	53.6	[46.28 , 60.83]
Sierra Leone	21.5	[17.31 , 25.66]
St. Lucia	43.2	[38.46 , 47.93]
Tajikistan	26.7	[18.32 , 35.03]
Thailand	36.6	[31.25 , 42.00]
Uganda	11.6	[6.51 , 16.61]
Uruguay	9.1	[5.94 , 12.17]
Uzbekistan	54.6	[50.02 , 59.18]
Vietnam	15.9	[11.72 , 20.14]
Zimbabwe	15.8	[10.42 , 21.14]

Source: Authors’ calculations.

For peer review only

Supplemental Table S4: Tukey's tests of significance of differences across groups in percent of population hesitant to take the COVID-19 vaccine

Group	Comparison	Difference	Std. Error	t-score	p-value	Signi-ficance
Gender	Female vs Male	0.052	0.022	2.316	0.021	**
Household Status	Head vs Non-Head	-0.063	0.026	-2.398	0.016	**
Age	Ages 65 and older vs Age 35 - 64	-0.023	0.025	-0.937	0.349	
	Ages 65 and older vs Age 34 and younger	-0.026	0.026	-0.994	0.320	
	Age 35 - 64 vs Age 34 and younger	-0.002	0.021	-0.107	0.543	
Education	Any Primary vs Any Secondary	0.008	0.030	0.250	0.599	
	Any Primary vs Any Tertiary	0.061	0.026	2.355	0.019	**
	Any Primary vs No Education	-0.030	0.043	-0.705	0.481	
	Any Secondary vs Any Tertiary	0.053	0.028	1.941	0.052	*
	Any Secondary vs No Education	-0.038	0.044	-0.860	0.390	
	Any Tertiary vs No Education	-0.091	0.041	-2.211	0.027	**
Sector	Rural vs Urban	0.056	0.023	2.375	0.018	**
Region	EAP vs ECA	-0.326	0.031	-10.450	0.000	***
	EAP vs LAC	0.182	0.026	7.077	0.000	***
	EAP vs MENA	-0.212	0.050	-4.219	0.000	***
	EAP vs SSA	0.107	0.031	3.454	0.001	***
	ECA vs LAC	0.508	0.021	24.579	0.000	***
	ECA vs MENA	0.114	0.048	2.390	0.017	**
	ECA vs SSA	0.433	0.027	16.103	0.000	***
	LAC vs MENA	-0.394	0.044	-8.870	0.000	***
	LAC vs SSA	-0.075	0.020	-3.705	0.000	***
	MENA vs SSA	0.319	0.048	6.691	0.000	***

For peer review only

Income Group	HIC vs LIC	-0.087	0.037	-2.368	0.018	**
	HIC vs LMIC	-0.218	0.024	-9.202	0.000	***
	HIC vs UMIC	-0.068	0.020	-3.481	0.001	***
	LIC vs LMIC	-0.131	0.037	-3.559	0.000	***
	LIC vs UMIC	0.019	0.024	0.808	0.419	
	LMIC vs UMIC	0.150	0.025	6.010	0.000	***

Significance levels: *** for p-values <= 0.01, ** for 0.01 < p-values <= 0.05, * for 0.05 < p-values <= 0.1

Source: Authors' calculations.

For peer review only

Supplemental Table S5a: Correlates of vaccine hesitancy (Logit model, marginal effects)

Dep. Var: Hesitancy	(1)	(2)	(3)	(4)	(5)	(6)
Male	-0.027*	-0.028*	-0.027*	-0.027*	-0.033**	-0.040***
	(0.013)	(0.013)	(0.013)	(0.012)	(0.012)	(0.011)
Head of HH	0.013	0.009	0.021	-0.001	0.014	0.007
	(0.014)	(0.014)	(0.013)	(0.016)	(0.015)	(0.012)
Education of respondent (ref – no education)						
No education	-0.062	-0.062	-0.055	-0.068*	-0.074*	-0.075*
	(0.032)	(0.032)	(0.031)	(0.034)	(0.036)	(0.035)
Any primary	-0.061	-0.060	-0.054	-0.072	-0.069	-0.086*
	(0.037)	(0.038)	(0.033)	(0.040)	(0.042)	(0.038)
Any secondary	-0.130***	-0.131***	-0.122***	-0.142***	-0.133**	-0.153***
	(0.036)	(0.037)	(0.033)	(0.040)	(0.044)	(0.040)
Age group (ref. -- 34 and younger)						
Working age (35-64)	-0.047***	-0.048***	-0.042**	-0.050***	-0.046**	-0.049***
	(0.014)	(0.013)	(0.015)	(0.013)	(0.015)	(0.012)
Retirement age (65+)	-0.103***	-0.102***	-0.096***	-0.101***	-0.103***	-0.102***
	(0.024)	(0.025)	(0.024)	(0.023)	(0.025)	(0.024)
Rural area	0.016	0.014	0.015	0.008	0.012	0.011
	(0.019)	(0.018)	(0.017)	(0.015)	(0.015)	(0.011)
Region (ref – LAC)						
EAP	0.189**	0.192*	0.222**	0.095	0.264*	0.188
	(0.067)	(0.078)	(0.077)	(0.069)	(0.130)	(0.130)
ECA	0.313***	0.315***	0.295***	0.328***	0.313***	0.295***
	(0.055)	(0.057)	(0.056)	(0.059)	(0.040)	(0.062)
MNA	0.208***	0.212***	0.245***	0.216***	0.282***	0.369***
	(0.037)	(0.040)	(0.062)	(0.032)	(0.059)	(0.051)
SSA	0.074	0.081	0.070	-0.022	0.047	0.022
	(0.075)	(0.077)	(0.070)	(0.105)	(0.083)	(0.104)
Income group (ref. – LIC)						
LMIC	-0.068	-0.065	-0.045	-0.088	-0.054	-0.071
	(0.122)	(0.119)	(0.112)	(0.123)	(0.097)	(0.069)
UMIC	-0.047	-0.038	-0.066	-0.095	-0.035	-0.137
	(0.149)	(0.148)	(0.130)	(0.150)	(0.116)	(0.087)
HIC	-0.173	-0.164	-0.190	-0.223	-0.202	-0.309***
	(0.163)	(0.160)	(0.143)	(0.164)	(0.130)	(0.090)
Survey month (ref -- Nov 2020 - Jan						
March - May 2021	-0.069	-0.060	-0.094	-0.056	-0.095	-0.083
	(0.095)	(0.108)	(0.103)	(0.098)	(0.104)	(0.095)
June - August 2021	-0.029	-0.025	-0.024	-0.052	-0.057	-0.055
	(0.088)	(0.098)	(0.100)	(0.087)	(0.092)	(0.075)
New COVID-19 cases per million, terciles (ref. – bottom tercile)						
Cases (middle tercile)		-0.017				0.026
		(0.067)				(0.060)
Cases (top tercile)		-0.019				-0.016
		(0.057)				(0.047)
Oxford stringency index terciles (ref. – bottom tercile)						
Stringency (middle tercile)			0.065			0.134
			(0.072)			(0.070)
Stringency (top tercile)			-0.008			0.027
			(0.076)			(0.068)
WHO excess deaths due to COVID-19 terciles (ref.- bottom tercile)						

For peer review only

Dep. Var: Hesitancy	(1)	(2)	(3)	(4)	(5)	(6)
Excess deaths (middle tercile)				-0.117*		-0.109
				(0.052)		(0.072)
Excess deaths (top tercile)				-0.069		0.040
				(0.072)		(0.108)
Confidence in government index tercile (ref.- top tercile)						
Confidence in government					0.048	0.070
					(0.053)	(0.057)
Confidence in government					0.024	0.053
					(0.097)	(0.103)
Pseudo R-squared	0.0745	0.0748	0.0794	0.0813	0.0869	0.1067
N	65088	65088	65088	65088	65088	65088

Notes: Weighted logit regressions. Marginal effects reported. Standard errors clustered at country level. Ref=reference group; EAP= East Asia and the Pacific; ECA= Europe and Central Asia; LAC=Latin America and the Caribbean; MNA= Middle East and North Africa; SSA= Sub-Saharan Africa; LIC=Low-income countries; LMIC=Lower middle-income countries, UMIC=Upper middle-income countries.

*, **, *** indicates significance at the 95%, 99%, and 99.9% level.

Source: Authors' calculations.

For peer review only

Supplemental Table S5b: Correlates of vaccine hesitancy (Ordinal logit regression, marginal effects)

Dep. Var.:	Would you get the vaccine?		
	No	Not sure	Yes
Male	-0.025**	-0.007**	0.036**
	(0.01)	(0.002)	(0.011)
Head of HH	0.011	0.001	-0.006
	(0.012)	(0.002)	(0.011)
Education of respondent (ref – Tertiary)			
No education	0.119***	0.022**	-0.156***
	-0.028	(0.007)	(0.038)
Any primary	0.057**	0.013**	-0.080***
	-0.018	(0.004)	(0.022)
Any secondary	0.058***	0.012***	-0.068***
	(0.009)	(0.003)	(0.010)
Age group (ref. -- 34 and younger)			
Working age (35-64)	-0.040***	-0.009***	0.049***
	(0.010)	(0.002)	(0.012)
Retirement age (65+)	-0.079***	-0.018***	0.097***
	(0.020)	(0.005)	(0.025)
Rural area	0.010	0.002	-0.012
	(0.009)	(0.002)	(0.011)
Region (ref – LAC)			
EAP	0.149	0.034	-0.183
	(0.093)	(0.021)	(0.113)
ECA	0.238***	0.055***	-0.293***
	(0.044)	(0.016)	(0.058)
MNA	0.288***	0.066***	-0.354***
	(0.042)	(0.011)	(0.049)
SSA	0.015	0.004	-0.019
	(0.082)	(0.019)	(0.101)
Income group (ref. – LIC)			
LMIC	-0.074	-0.017	0.091
	(0.055)	(0.012)	(0.067)
UMIC	-0.109	-0.025	0.134
	(0.069)	(0.017)	(0.085)
HIC	-0.241***	-0.056**	0.297***
	(0.071)	(0.019)	(0.088)
Survey month (ref -- Nov 2020 - Jan 2021)			
March - May 2021	-0.054	-0.011	0.065
	(0.081)	(0.016)	(0.098)
June - August 2021	-0.046	-0.010	0.055
	(0.065)	(0.012)	(0.077)
New COVID-19 cases per million, terciles (ref. – top tercile)			
Cases (middle tercile)	0.016	0.003	-0.019
	(0.048)	(0.010)	(0.058)
Cases (bottom tercile)	-0.028	-0.006	0.034
	(0.037)	(0.008)	(0.045)
Oxford stringency index terciles (ref. – top tercile)			
Stringency (middle tercile)	0.089	0.020	-0.110
	(0.057)	(0.013)	(0.070)
Stringency (bottom tercile)	0.012	0.003	-0.015
	(0.041)	(0.011)	(0.052)

For peer review only

Dep. Var.:	Would you get the vaccine?		
	No	Not sure	Yes
WHO excess deaths due to COVID-19 terciles (ref.- bottom tercile)			
Excess deaths (middle tercile)	-0.075	-0.019	0.094
	(0.057)	(0.015)	(0.071)
Excess deaths (top tercile)	0.057	0.010	-0.067
	(0.096)	(0.015)	(0.112)
Confidence in government index tercile (ref.- top tercile)			
Confidence in government (middle tercile)	0.052	0.016	-0.069
	(0.041)	(0.013)	(0.054)
Confidence in government (bottom tercile)	0.040	0.013	-0.053
	(0.070)	(0.023)	(0.093)
R-squared		0.0866	
N		65088	

Notes: Weighted ordinal logit regressions. Categories ranked from no to not sure to yes. Marginal effects reported. Standard errors clustered at country level. Ref=reference group; EAP= East Asia and the Pacific; ECA= Europe and Central Asia; LAC=Latin America and the Caribbean; MNA= Middle East and North Africa; SSA= Sub-Saharan Africa; LIC=Low-income countries; LMIC=Lower middle-income countries, UMIC=Upper middle-income countries.

*, **, *** indicates significance at the 95%, 99%, and 99.9% level.

Source: Authors' calculations.

For peer review only

Supplemental Table S6: Percent of population non-hesitant to take the COVID-19 vaccine, 14 countries with multiple rounds of survey results (95% confidence intervals in parentheses).

country	**Oct-2020**	**Nov-2020**	**Dec-2020**	**Jan-2021**	**Feb-2021**	**Mar-2021**	**Apr-2021**	**May-2021**	**Jun-2021**	**Jul-2021**	**Aug-2021**
Burkina Faso			0.79 (0.73, 0.86)						0.76 (0.67, 0.86)		
Congo, Rep.			0.84 (0.75, 0.94)			0.87 (0.81, 0.93)					
Ethiopia	0.98 (0.91, 1.00)				0.97 (0.89, 1.00)						
Gambia, The			0.65 (0.58, 0.71)				0.55 (0.49, 0.60)				0.67 (0.60, 0.74)
Georgia				0.36 (0.33, 0.38)		0.29 (0.27, 0.32)			0.35 (0.32, 0.38)		
Indonesia		0.79 (0.73, 0.85)				0.79 (0.72, 0.85)					
Iraq			0.66 (0.61, 0.72)	0.50 (0.45, 0.54)					0.45 (0.40, 0.50)	0.47 (0.42, 0.52)	0.53 (0.47, 0.58)
Kazakhstan					0.23 (0.17, 0.28)			0.32 (0.27, 0.37)	0.25 (0.21, 0.29)		
Malawi		0.83 (0.76, 0.89)				0.52 (0.47, 0.58)	0.71 (0.64, 0.78)				
Nigeria	0.86 (0.80, 0.93)				0.83 (0.77, 0.90)						
Philippines			0.44 (0.40, 0.48)					0.46 (0.42, 0.51)			
Tajikistan								0.73 (0.61, 0.84)	0.79 (0.67, 0.92)	0.80 (0.65, 0.95)	0.73 (0.57, 0.90)
Uganda		0.84 (0.79, 0.90)			0.88 (0.83, 0.94)						
Uzbekistan							0.55 (0.51, 0.60)	0.50 (0.46, 0.54)	0.45 (0.41, 0.49)		

Source: Authors' calculations.

For peer review only

Supplemental Table S7: Comparison of results on vaccine hesitancy from the HFPS and other studies

**Comparison with de Figueiredo and Larson (2021)**		
	**HFPS**	**de Figueiredo and Larson**
Argentina	10.1%	24.3%
Brazil	3.1%	17.0%
Chile	3.4%	27.9%
Croatia	33.2%	58.5%
Ecuador	19.4%	20.3%
Indonesia	21.3%	17.1%
Lebanon	32.2%	55.9%
Malaysia	25.8%	13.9%
Mexico	6.2%	18.0%
Nigeria	15.2%	35.9%
Paraguay	15.3%	48.5%
Peru	10.5%	28.3%
Vietnam	15.9%	3.2%
Average	*16.3%*	*28.4%*
**Comparison with Wouters et al. 2021**		
	**HFPS**	**Wouters et al.**
Argentina	10.1%	24.0%
Brazil	3.1%	12.0%
Chile	3.4%	28.0%
Croatia	33.2%	59.0%
Ecuador	19.4%	20.0%
Indonesia	42.6%	17.0%
Lebanon	32.2%	56.0%
Mexico	6.2%	18.0%
Nigeria	30.3%	36.0%
Paraguay	15.3%	49.0%
Peru	10.5%	28.0%
Vietnam	15.9%	2.0%
Average	*18.5%*	*29.1%*
**Comparison with Gallup (2021)**		
**Country**	**HFPS Hesitancy**	**Gallup**
Argentina	10.1%	37.0%
Bolivia	24.2%	35.0%
Brazil	3.1%	30.0%
Burkina Faso	22.2%	44.0%
Chile	3.4%	40.0%
Colombia	11.2%	30.0%
Congo, Dem. Rep.	61.2%	61.2%
Congo, Rep.	14.4%	48.0%

For peer review only

Costa Rica	11.9%	26.0%
Croatia	33.2%	57.0%
Dominican Republic	5.0%	35.0%
Ecuador	19.4%	28.0%
El Salvador	8.1%	25.0%
Ethiopia	2.8%	16.0%
Georgia	66.8%	44.0%
Guatemala	29.6%	29.0%
Guinea	20.1%	46.0%
Honduras	13.9%	30.0%
Indonesia	21.3%	30.0%
Iraq	47.8%	39.0%
Jamaica	50.6%	68.0%
Kenya	17.9%	27.0%
Lao PDR	12.9%	16.0%
Lebanon	32.2%	57.0%
Malaysia	25.8%	28.0%
Mali	21.1%	51.0%
Mexico	6.2%	25.0%
Mongolia	19.2%	39.0%
Nicaragua	18.8%	13.0%
Nigeria	15.2%	42.0%
Paraguay	15.3%	47.0%
Peru	10.5%	27.0%
Philippines	54.9%	49.0%
Tajikistan	23.7%	36.0%
Thailand	36.6%	39.0%
Uganda	13.6%	38.0%
Uruguay	9.1%	39.0%
Uzbekistan	49.8%	30.0%
Vietnam	15.9%	19.0%
Zimbabwe	15.8%	27.0%
Average	*22.4%*	*36.2%*
**Comparison with Facebook (2021)**		
**Country**	**HFPS**	**FB**
Argentina	10.1%	21.5%
Belize	28.4%	48.0%
Brazil	3.1%	25.8%
Chile	3.4%	43.0%
Colombia	11.2%	15.7%
Costa Rica	11.9%	17.1%
Ecuador	19.4%	17.1%

For peer review only

El Salvador	8.1%	15.8%
Guatemala	29.6%	18.6%
Guyana	20.0%	54.4%
Honduras	13.9%	13.7%
Indonesia	21.3%	19.2%
Iraq	47.8%	52.8%
Lebanon	32.2%	22.3%
Mexico	6.2%	13.0%
Nicaragua	18.8%	37.4%
Panama	13.3%	24.9%
Paraguay	15.3%	16.2%
Peru	10.5%	12.2%
Uruguay	9.1%	50.8%
Average	*16.7%*	*27.0%*

For peer review only

Supplemental Figure S1: Vaccination rates by income group

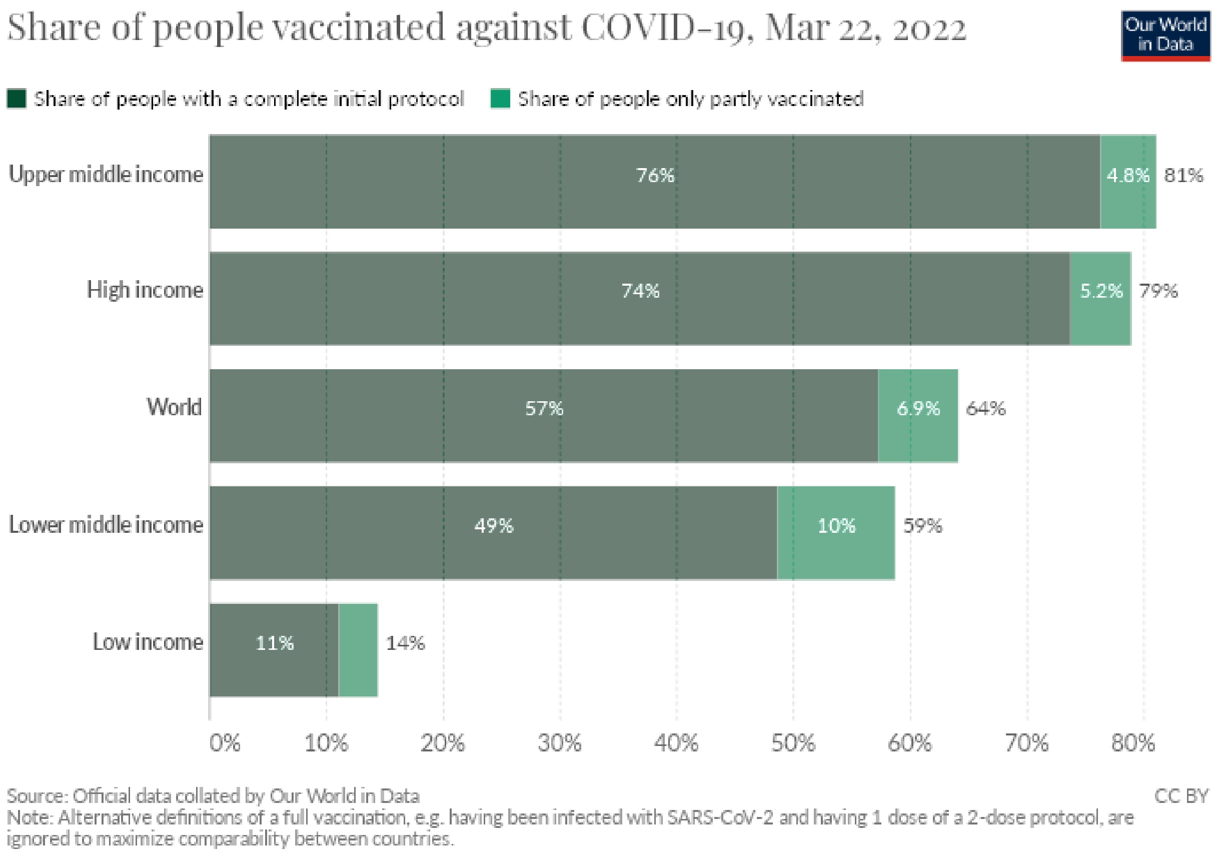

Source: Ritchie et al. 2022 as of March 22, 2022.

Supplemental Figure S2: Vaccine rollout rates by income group

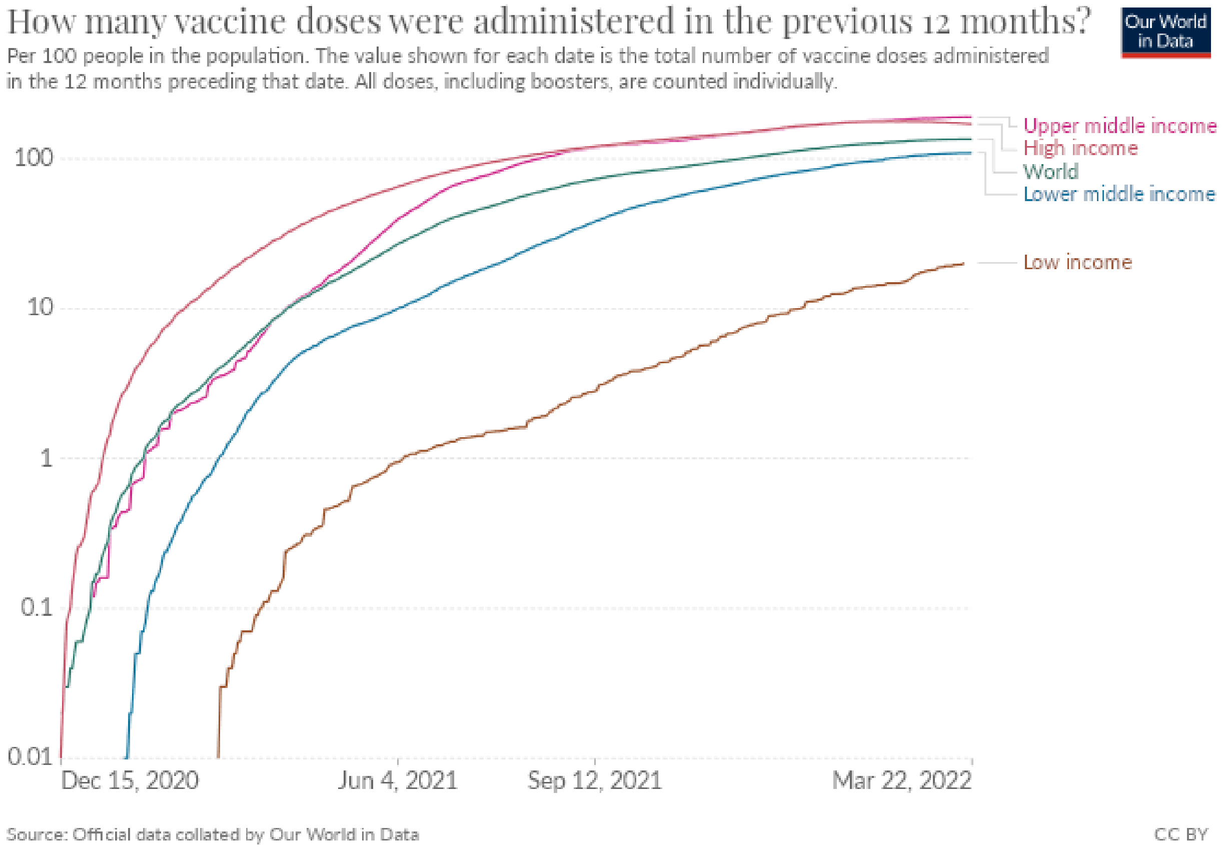

Source: Ritchie et al. 2022 as of March 22, 2022.

Supplemental Figure S3: Strength of sentiment of COVID-19 vaccine hesitancy: share of households in 46 countries reporting “yes,” “not sure,” or “no” when asked if they will take the COVID-19 vaccination when available

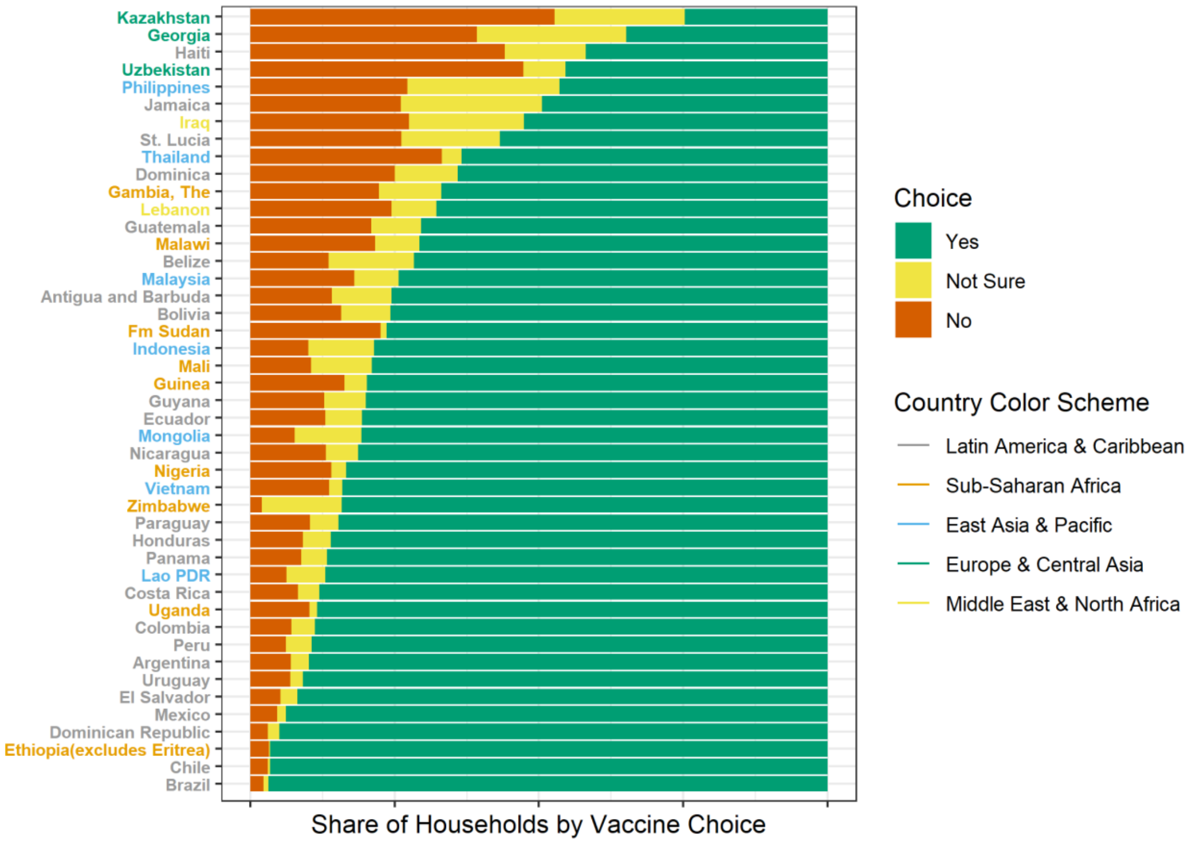

Source: Authors’ calculations.

w only

Supplemental Figure S4: Self-reported reason for vaccine hesitancy in countries with multiple answers allowed, HFPS

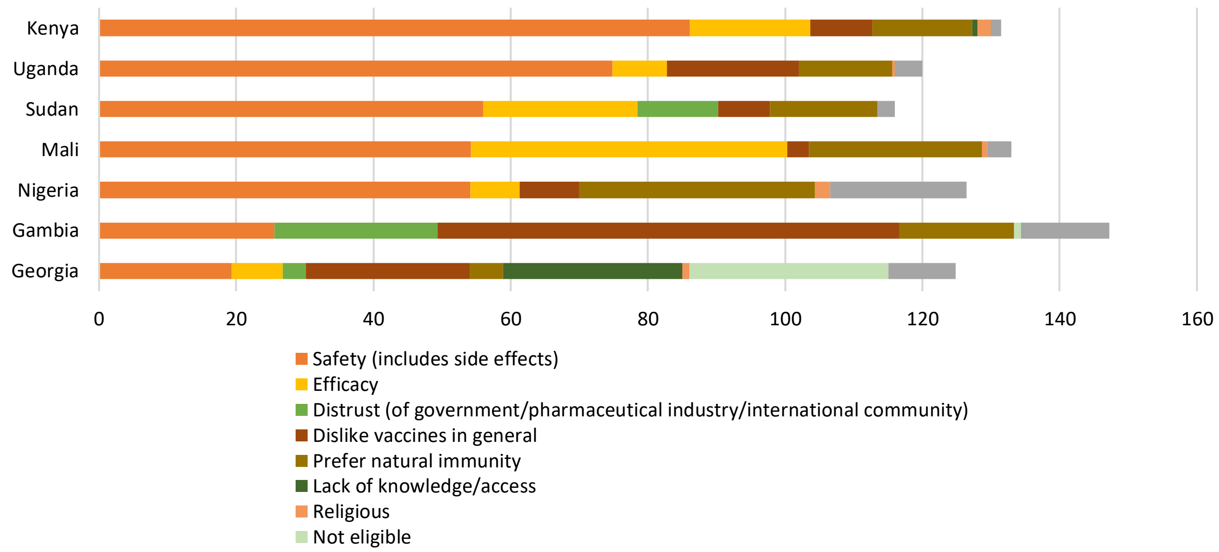

Notes: The X axis represents share of vaccine hesitant households. In some cases, the share is larger than 100% because multiple responses were allowed. For the construction of harmonized response categories, see Supplemental Table S2b.

Source: Authors' calculations.

Supplemental Figure S5: Comparison of share of population COVID-19 that is hesitant to take the COVID-19 vaccine, HFPS with other studies

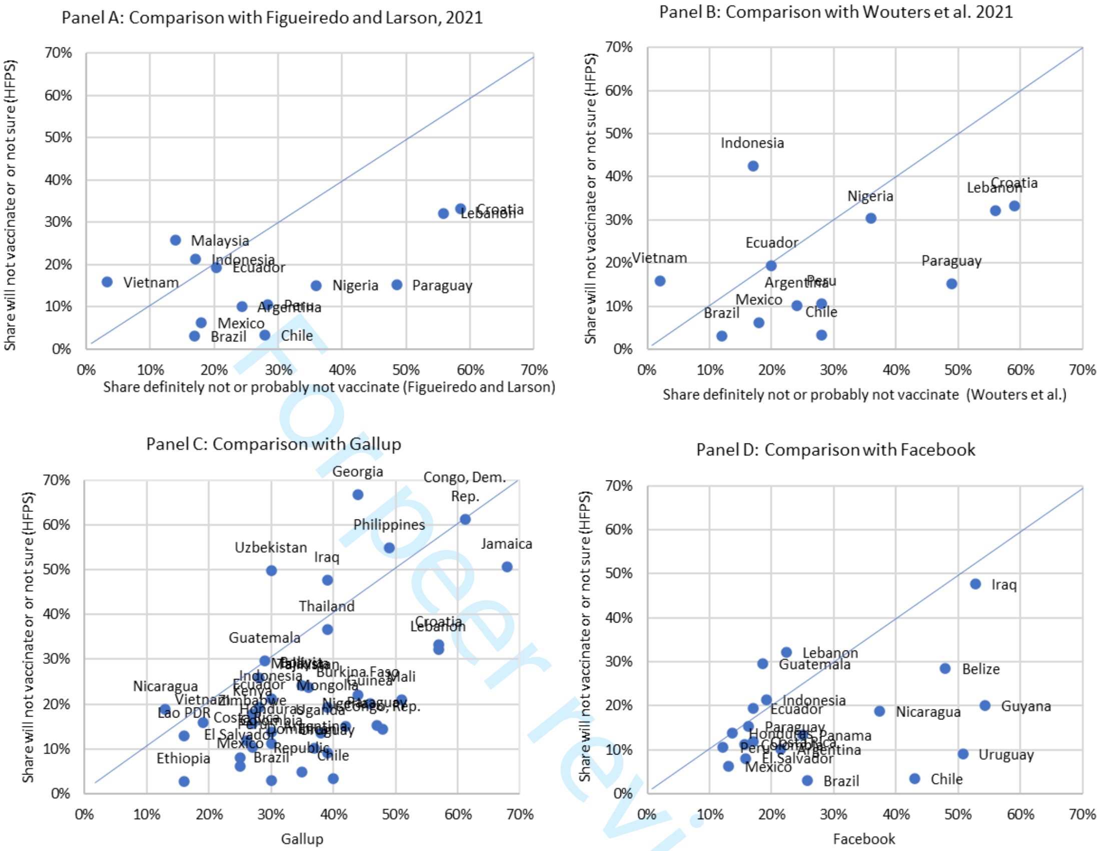

Notes: The y-axes indicate the share of the sample who reported not being willing or not sure if they will take the COVID-19 vaccine, as reported in the HFPS. The x-axes indicate the share of households in the survey that will definitely or probably not be vaccinated against COVID-19 from the following sources. Panel A: Figueiredo and Larson (2021). Panel B: Wouters et al. (2021). Panel C: Gallup (2021). Panel D: Facebook (2021).

For peer review only

Supplemental Figure S6: Comparison of COVID-19 Vaccine Hesitancy with pre-COVID sentiment about vaccines from 2019 Global Monitor, 45 countries

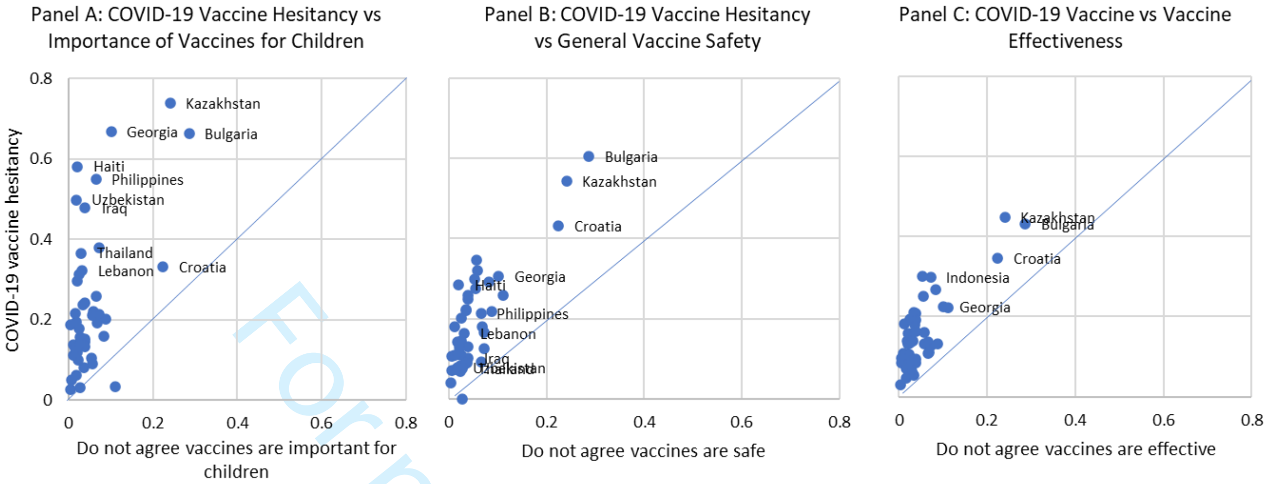

Notes: The y-axes indicate the share of the sample who report either not willing or not sure if they will vaccinate, as reported in the HFPS. The x-axes indicate the share of the 2019 Global Monitor Report sample that reported they do not agree vaccines are important for children (Panel A), do not agree that vaccines are safe (Panel B), and do not agree vaccines are effective.

For peer review only

Supplemental Figure S7: Reason for "definitely not" (upper panel) or "probably not" (lower panel) taking the COVID-19 vaccine, Facebook data for month of HFPS)

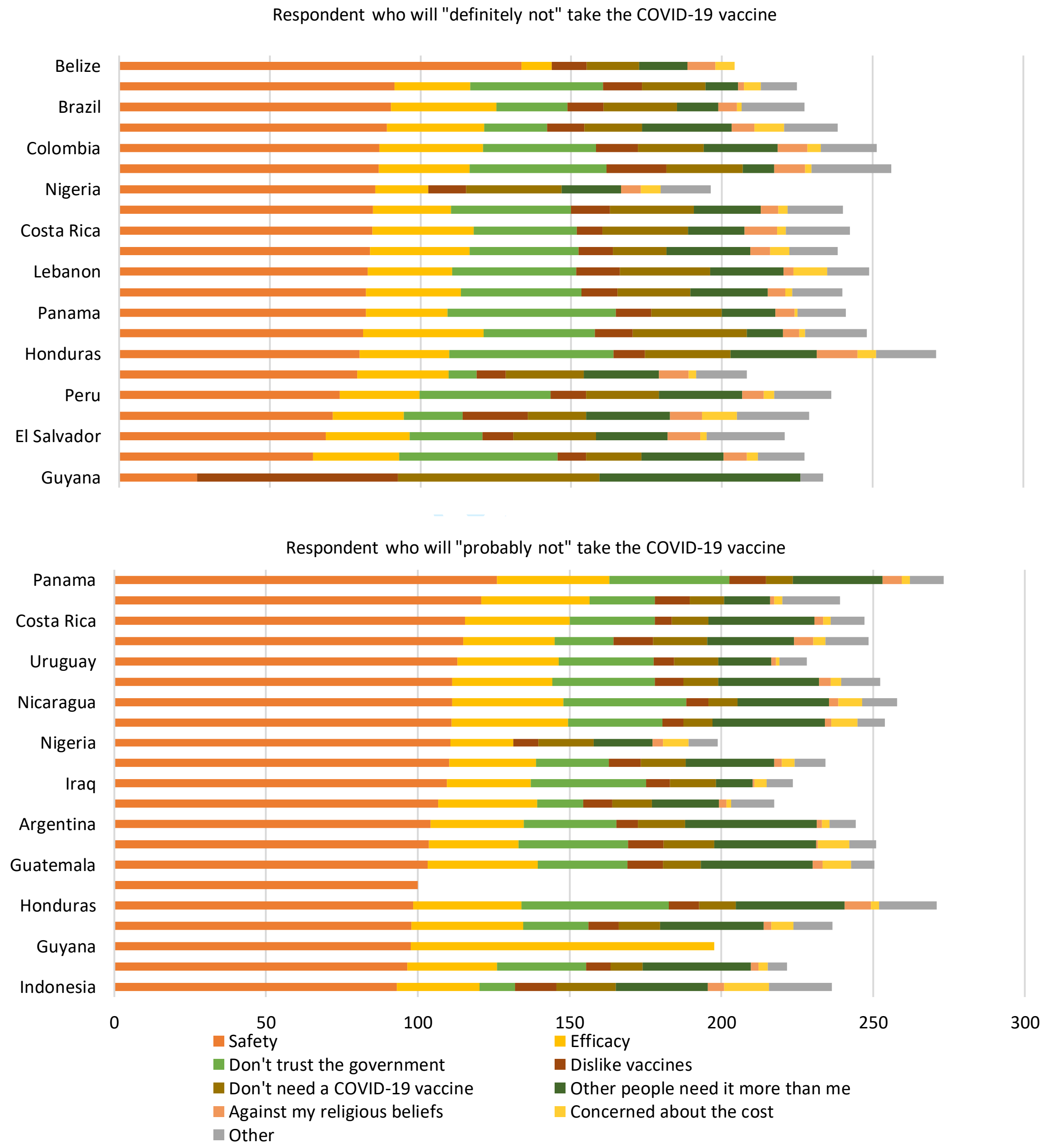

Notes: Y-axes indicate the share of survey respondents; multiple answers were allowed. Data source: Facebook (2021) for same month as reported in the HFPS. The month for each country is reported in Supplemental Table S1.

References- Supplemental Materials

Facebook. 2021. COVID-19 Trends and Impact Survey.

de Figueiredo A, Larson HJ. Exploratory study of the global intent to accept COVID-19 vaccinations. Communications medicine. 2021 Sep 9;1(1):1-0.

Gallup. 2019. Wellcome Global Monitor – First Wave Findings. https://wellcome.org/reports/wellcome-global-monitor/2018#downloads-4d1c.

Gallup. 2021. Gallup World Poll. https://www.gallup.com/178667/gallup-world-poll-work.aspx.

Hale T, Angrist N, Goldszmidt R, et al. 2022. A global panel database of pandemic policies (Oxford COVID-19 government response Tracker). Nat Hum Behav 2021;5:529–38. Database accessed Feb 28, 2022.

Inglehart, R, C. Haerpfer, A. Moreno et al. 2022. Would Values Survey: Round Seven – Country Pooled Datafile Version: https://www.worldvaluessurvey.org/WVSOnline.jsp. (accessed April 21, 2022) Madrid: JD Systems Institute.

Lazarus, JV, Ratzan SC , and Palayew A et al. 2021. A global survey of potential acceptance of a COVID-19 vaccine. Brief Communication. Nature Medicine 27: 225–228. https://doi.org/10.1038/s41591-020-1124-9.

Sallam M. COVID-19 Vaccine Hesitancy Worldwide: A Concise Systematic Review of Vaccine Acceptance Rates. Vaccines (Basel). 2021 Feb 16;9(2):160. doi: 10.3390/vaccines9020160. PMID: 33669441; PMCID: PMC7920465.

Solís Arce, J.S., Warren, S.S., Meriggi, N.F. *et al.* COVID-19 vaccine acceptance and hesitancy in low- and middle-income countries. *Nat Med* **27,** 1385–1394 (2021). https://doi.org/10.1038/s41591-021-01454-y.

World Bank. 2020. High Frequency Mobile Phone Surveys of Households to Assess the Impacts of COVID-19: Overview (English). Washington, D.C.: World Bank Group. http://documents.worldbank.org/curated/en/703571588695361920/Overview

World Bank. 2021. World Development Indicators. Washington, D.C.: The World Bank (producer and distributor). http://data.worldbank.org/data-catalog/world-development-indicators. Accessed March 12, 2022.

World Health Organization. 2022. Global Excess Deaths Associated with COVID-19 (Modelled Estimates). Downloaded on September 8, 2022 at https://www.who.int/data/sets/global-excess-deaths-associated-with-covid-19-modelled-estimates.

Wouters, O.J., Shadlen, K.C., Salcher-Konrad, M., Pollard, A.J., Larson, H.J., Teerawattananon, Y. and Jit, M., 2021. Challenges in ensuring global access to COVID-19 vaccines: production, affordability, allocation, and deployment. The Lancet, 397(10278), pp.1023-1034.

For peer review only

How prevalent is COVID-19 Vaccine Hesitancy in Low- and Middle-Income Countries and What Are the Key Drivers of Hesitancy? Results from 53 Countries

Journal:	*BMJ Open*
Manuscript ID	bmjopen-2022-069152.R2
Article Type:	Original research
Date Submitted by the Author:	15-Sep-2023
Complete List of Authors:	Dayton Eberwein, Julia; World Bank Group, HNP GP<br>Edochie, Ifeanyi; World Bank Group<br>Newhouse, David; World Bank Group<br>Cojocaru, Alexandru; World Bank Group<br>Bopahbe, Gildas; World Bank Group<br>Kakietek, Jakub; World Bank Group<br>Kim, Yeon Soo; World Bank Group<br>Montes, Jose; World Bank Group
**Primary Subject Heading**:	Global health
Secondary Subject Heading:	Health policy, Infectious diseases, Public health
Keywords:	COVID-19, Health policy < HEALTH SERVICES ADMINISTRATION & MANAGEMENT, Public health < INFECTIOUS DISEASES

SCHOLARONE™
Manuscripts

I, the Submitting Author has the right to grant and does grant on behalf of all authors of the Work (as defined in the below author licence), an exclusive licence and/or a non-exclusive licence for contributions from authors who are: i) UK Crown employees; ii) where BMJ has agreed a CC-BY licence shall apply, and/or iii) in accordance with the terms applicable for US Federal Government officers or employees acting as part of their official duties; on a worldwide, perpetual, irrevocable, royalty-free basis to BMJ Publishing Group Ltd ("BMJ") its licensees and where the relevant Journal is co-owned by BMJ to the co-owners of the Journal, to publish the Work in this journal and any other BMJ products and to exploit all rights, as set out in our licence.

The Submitting Author accepts and understands that any supply made under these terms is made by BMJ to the Submitting Author unless you are acting as an employee on behalf of your employer or a postgraduate student of an affiliated institution which is paying any applicable article publishing charge ("APC") for Open Access articles. Where the Submitting Author wishes to make the Work available on an Open Access basis (and intends to pay the relevant APC), the terms of reuse of such Open Access shall be governed by a Creative Commons licence – details of these licences and which Creative Commons licence will apply to this Work are set out in our licence referred to above.

Other than as permitted in any relevant BMJ Author's Self Archiving Policies, I confirm this Work has not been accepted for publication elsewhere, is not being considered for publication elsewhere and does not duplicate material already published. I confirm all authors consent to publication of this Work and authorise the granting of this licence.

For peer review only

How prevalent is COVID-19 Vaccine Hesitancy in Low- and Middle-Income Countries and What Are the Key Drivers of Hesitancy? Results from 53 Countries

Julia Dayton Eberwein,[1*] Ifeanyi Edochie,[1] David Newhouse, [1] Alexandru Cojocaru, [1] Gildas Deudibe, [1] Jakub Kakietek,[1] Yeon Soo Kim, [1] and Jose Montes[1]

September 2022

Abstract

Objectives: To estimate the levels of COVID-19 vaccine hesitancy in 53 low- and middle-income countries, differences across population groups in hesitancy, and self-reported reasons for being hesitant to take the COVID-19 vaccine.

Methods: This paper presents new evidence on levels and trends of vaccine hesitancy in low- and middle-income countries based on harmonized high-frequency phone surveys from more than 120,000 respondents in 53 low- and middle-income countries collected between October 2020 August 2021. These countries represent a combined 53 percent of the population of low- and middle-income countries excluding India and China.

Results: On average across countries, one in five adults reported being hesitant to take the COVID-19 vaccine, with the most cited reasons for hesitancy being concerns about the safety of the vaccine, followed by concerns about its efficacy. Between late 2020 and the first half of 2021, there tended to be little change in hesitancy rates in 11 of the 14 countries with available data, while hesitancy increased in Iraq, Malawi, and Uzbekistan. COVID-19 vaccine hesitancy was higher among female, younger adults, and less educated respondents, after controlling for selected observable characteristics.

Conclusions: Country estimates of vaccine hesitancy from the high-frequency phone surveys are correlated with but lower than those from earlier studies, which often relied on less representative survey samples. The results suggest that vaccine hesitancy in low- and middle-income countries, while less prevalent than previously thought, will be an important and enduring obstacle to recovery from the pandemic.

[1] The World Bank, 1818 H. Street NW, Washington, DC 20433

* Corresponding author: jdayton@worldbank.org.

For peer review only

Strengths and limitations of this study

- The greatest strength of the study is that is provides nationally representative and comparable estimates of COVID-19 vaccine hesitancy for a very large sample (over 50) of low- and middle-income countries, filling a critical evidence gap.
- The study is the first to explore correlates and self-reported reasons for COVID-19 vaccine hesitancy in such a large and comparable sample of low-and middle-income countries.
- One potential limitation of this study is that it is based on phone survey data which exclude respondents who did not have access to a phone, although access to mobile phones was high in the countries in our sample and where possible sampling weights were used to correct for this bias.
- Another limitation is that the majority of the countries are located in two regions, Latin America and the Caribbean and Sub-Saharan Africa, making results less representative of other regions.

For peer review only

1. Introduction

The world is entering the third year of the global COVID-19 pandemic, which has caused enormous devastation to both people's health (450 million cases and 6 million deaths as of March 2022) and national economies, in the form of a global recession that has pushed millions into poverty (1,2). With the continued emergence of new variants and limited treatments available, it is commonly accepted that widespread vaccination is the world's best bet to contain the virus and it is also expected to play an important role in economic recovery (3,4). As of early March 2022, over 60 percent of the world's population had received at least one dose of a COVID-19 vaccine. However, there are stark disparities in vaccination rates across countries: only 14 percent of people in low-income countries had received at least one dose as of March 22, 2022, compared to 79 percent in high-income countries and 81 percent in upper-middle income countries (5) (supplemental figures S1 and S2). The lag in vaccine distribution in lower income countries had until recently put the focus mainly on supply-side constraints. However, with vaccine production now exceeding demand, it is increasingly important to understand the extent of vaccine hesitancy and the nature of the concerns.

Vaccine hesitancy refers to the delay in acceptance or refusal to vaccinate despite availability of vaccination services (6). It is not a new phenomenon, and some level of hesitancy exists for most vaccines; even before the COVID-19 pandemic began, the World Health Organization (WHO) identified vaccine hesitancy as one of the top threats to global health (7). Brewer and colleagues established a conceptual framework of behavioral and social drivers (BeSD) of vaccination that has been widely adopted, including by the WHO (8). These drivers of vaccination can be grouped and measured in four domains: thinking and feeling about vaccines (including confidence in vaccine effectiveness and concern about safety); social processes that drive or inhibit vaccination; motivation (or hesitancy) to seek vaccination; and practical issues involved in seeking and receiving vaccination. This framework is focused on proximal factors affecting decisions to vaccinate, which are thought to be potentially changeable by programs. Several aspects of the thinking and feeling domain are thought to contribute substantially to COVID-19 vaccine hesitancy: the accelerated speed of development of the COVID-19 vaccine, its newness, concerns about longer-term side-effects, uncertainty about the duration of the vaccine's effectiveness, and the type of vaccine (e.g., mRNA) and country of origin of the vaccine (9,10-12).

The multi-country studies that exist show relatively high levels of hesitancy to take the COVID-19 vaccine, but mainly focus on high-income countries (13-14). Less is understood about COVID-19 vaccine hesitancy in low- and middle-income countries. A few studies reported multi-country survey results from developing countries (15-20) or systematic reviews of individual country surveys (21), but few of the studies were based on nationally representative samples of respondents or included comparable results for many countries. Given this limitation, an important contribution of this analysis will be to validate these findings.

This paper extends the existing literature by providing estimates of and reasons for vaccine hesitancy for 53 low- and middle-income countries, which are comparable across countries and largely based on household surveys that are more representative of the national population. The 53 countries represent approximately 30% of the population of all low- and middle-income countries and 53% excluding India and China.

For peer review only - http://bmjopen.bmj.com/site/about/guidelines.xhtml

2. Data and Methods

2a. Data

This study describes the levels of vaccine hesitancy and its reasons in 53 low- and middle-income countries between October 2020 August 2021 using data from the high-frequency phone surveys (HFPS) implemented to monitor the impact of COVID-19 (22, 23). Data are available for one survey round from 39 countries and two or more survey rounds for 14 countries (supplemental table S1). The sampling frame was drawn from pre-existing nationally representative household surveys in 19 countries, random digit dialing (RDD) in 29 countries, and a list of phone numbers typically obtained from mobile phone operators in 5 countries (see supplement 1 for detailed description of methods).

2b. Outcome measures

The question about vaccine hesitancy, "When a vaccine to prevent COVID-19 is available to you, are you planning to be vaccinated?" varied slightly across surveys depending on whether a vaccine was available in the country at the time of the survey (See supplement 1). In the sample, 47 countries offered three answer options (yes, not sure, and no), while the other six offered two categories (yes and no). We combine the "no" and "not sure" answers to obtain the measure of vaccine hesitancy.

To obtain the respondent's reason for vaccine hesitancy, survey respondents who answered "no" or "not sure" were asked "What is your (main) reason/concern for not wanting to be vaccinated / not being sure if you want to be vaccinated?" The answer categories varied widely across surveys (supplemental table S2a). To make these more comparable across countries, answers were remapped into the nine most common categories (supplemental table 2b). The surveys also differed across countries in terms of whether a single concern or multiple concerns were collected, and to account for this, the results are presented separately.

2c. Contextual data

Country-level contextual data were drawn from other sources: new COVID-19 cases per million, measured as a 7-day rolling average prior to the midpoint of the month prior to survey data collection in each country (1); and the Oxford Stringency Index, ranging from 0–100, that indicates the degree to which restrictions were put in place by governments to control the pandemic (24); confidence in the press andgovernment (25); and estimated excess deaths due to COVID-19 (26).

2d. Analytical strategy

Data from 53 countries were pooled into a single data set. Sampling weights were scaled such that countries are weighted equally; in other words, the estimates of vaccine hesitancy are unweighted averages of population-weighted country averages. (Using population weights when aggregating across countries would have resulted in the results being driven by a small number of large countries.) First, we report point estimates and 95% confidence intervals for each country based on standard errors clustered at the state or province level within each country. We also report the simple average across all countries and stratified by: World Bank region; World Bank country income group; urban vs. rural residence; gender of respondent; whether respondent is head of household; age of the respondent; and educational attainment of the respondent. Tukey's test of multiple comparisons was used to test

For peer review only

differences in rates of vaccine hesitancy across groups. Second, we used multivariate regression analysis to assess the relative association of vaccine hesitancy and the correlates and contextual variables. Trends over time in levels of vaccine hesitancy were examined in countries with more than one wave of results. Finally, the analysis describes the reasons for vaccine hesitancy. Descriptive statistics were produced using R Software and regressions were estimated using STATA.

2e. Patient and public involvement

No patients were involved in this study. The results of the study will be disseminated to key policy- makers and relevant stakeholders involved in COVID- 19 vaccine deployment.

3. Results

3a. Levels of vaccine hesitancy across 53 countries

Overall, the average level of vaccine hesitancy across the most recent survey round in 53 countries was 20.0% (Confidence Intervals (CI) 17.2-22.7%). Across regions, average levels of hesitancy were highest in Europe and Central Asia (58.8%, CI 55.0-62.6%), followed by the Middle East and North Africa (47.4%, CI 38.8-56.0%), East Asia and Pacific (26.2%, CI 21.4-31.0%), Sub-Saharan Africa (15.5%, CI 11.8-19.2%), and Latin America and the Caribbean (8.0%, CI 6.5-9.5%) (Figure 1 and supplemental table S3). Differences between all regions were statistically significant at the 5% level (supplemental table S4). When considered by country income group, the highest average level of hesitancy was in lower middle-income countries (27.7%, CI 23.8-31.7%), followed by low-income countries (14.6%, CI 7.8-21.4%), upper middle-income countries (12.7%, CI 9.8-15.6%), and high-income countries (5.9%, CI 3.4-8.4). Differences in hesitancy across country income groups were significant at the 5% level across all pairs except for the comparison between low-income and upper-middle income groups.

3b. Bivariate correlates of vaccine hesitancy

Rural households reported significantly higher levels of vaccine hesitancy than urban households (23.2% vs. 17.7%, p-value=0.018), and female respondents (22.5%) were significantly more likely than male respondents (17.3%) to be vaccine hesitant (p-value=0.021). Among households in the 33 surveys with information on the educational attainment of the respondent, respondents with lower levels of education had on average higher levels of hesitancy. Younger respondents reported higher levels of hesitancy than older respondents, but the differences were not significant.

3c. Vaccine hesitancy by country

The highest levels of vaccine hesitancy were reported in Kazakhstan (75.3%), Bulgaria (66.2%) and Georgia (65.2%), and the lowest levels in Chile (3.4%), Ethiopia (3.4%), and Brazil (3.1%) (Figure 2). Strength of vaccine hesitancy was available for the 46 countries that included three answer options (yes, not sure, and no). In four countries (Philippines, Georgia, Jamaica, and Kazakhstan), over 20% of respondents reported being unsure of whether they would get vaccinated, and in another 11 countries, between 10% and 20% of the sample was unsure whether they would be willing to be vaccinated against COVID-19 (supplemental figure S3).

3c. Multivariate analysis of correlates of vaccine hesitancy

For peer review only

Results from a multivariate regression generally confirmed the bivariate results. Table 1 shows ordinary least squares (OLS) regression results: a baseline model with the correlates described above plus month of survey (expressed in terciles),[1] and extended models with additional contextual variables (expressed in terciles). Results were consistent across models, as were the marginal effects from logit regressions (supplemental Table S5a). Men were less COVID-19 vaccine hesitant than women, although the magnitude was small. Having more formal education was associated with being less hesitant and the differences were more substantial. Age was inversely correlated with hesitancy. There were no statistically significant differences between hesitancy among rural and urban respondents. Differences across regions were statistically significant and, for some regions, quite large, but there were no significant differences by country income group.

Table 1: Multivariate analysis of vaccine hesitancy in 53 low- and middle-income countries (Ordinary least squares)

Dep. Var: Hesitancy	(1)	(2)	(3)	(4)	(5)	(6)
Male	-0.028*	-0.027*	-0.028	-0.027*	-0.031*	-0.038**
	(0.013)	(0.013)	(0.014)	(0.013)	(0.013)	(0.012)
Head of HH	0.012	0.009	0.019	0.000	0.011	0.007
	(0.015)	(0.014)	(0.013)	(0.017)	(0.015)	(0.013)
Education of respondent (ref – No education)						
Any primary	-0.062	-0.062*	-0.056	-0.067*	-0.073*	-0.073*
	(0.031)	(0.031)	(0.030)	(0.032)	(0.034)	(0.032)
Any secondary	-0.059	-0.060	-0.053	-0.069	-0.067	-0.082*
	(0.035)	(0.036)	(0.032)	(0.037)	(0.039)	(0.035)
Any tertiary	-0.123***	-0.125***	-0.116***	-0.134***	-0.125**	-0.143***
	(0.033)	(0.035)	(0.032)	(0.037)	(0.040)	(0.036)
Age group (ref. – 34 and younger)						
Working age (35-64)	-0.047**	-0.048***	-0.042**	-0.050***	-0.045**	-0.049***
	(0.014)	(0.013)	(0.015)	(0.013)	(0.015)	(0.012)
Retirement age (65+)	-0.103***	-0.102***	-0.096***	-0.103***	-0.099***	-0.099***
	(0.023)	(0.024)	(0.023)	(0.022)	(0.024)	(0.023)
Rural area	0.016	0.014	0.013	0.010	0.012	0.011
	(0.019)	(0.018)	(0.018)	(0.015)	(0.015)	(0.011)
Region (ref – LAC)						
EAP	0.211**	0.218*	0.241**	0.127	0.299	0.249
	(0.076)	(0.086)	(0.084)	(0.076)	(0.155)	(0.137)
ECA	0.363***	0.364***	0.347***	0.376***	0.369***	0.345***
	(0.075)	(0.076)	(0.073)	(0.076)	(0.056)	(0.068)
MNA	0.231***	0.230***	0.262***	0.240***	0.288***	0.393***
	(0.043)	(0.043)	(0.062)	(0.036)	(0.059)	(0.050)
SSA	0.082	0.089	0.078	-0.005	0.072	0.057
	(0.075)	(0.079)	(0.074)	(0.101)	(0.086)	(0.103)
Country income group (ref. – LIC)						
LMIC	-0.073	-0.071	-0.052	-0.090	-0.049	-0.053
	(0.127)	(0.124)	(0.122)	(0.129)	(0.113)	(0.081)
UMIC	-0.052	-0.050	-0.071	-0.096	-0.026	-0.130
	(0.154)	(0.154)	(0.146)	(0.154)	(0.135)	(0.107)

[1] Time of survey was split into the following 3 groups: (i) November 2020 – January 2021; (ii) March – May 2021; and (iii) June – August 2021. There are no observations in the data for February and April of 2021.

For peer review only

Dep. Var: Hesitancy	(1)	(2)	(3)	(4)	(5)	(6)
HIC	-0.161	-0.160	-0.183	-0.208	-0.175	-0.280*
	(0.159)	(0.162)	(0.153)	(0.162)	(0.136)	(0.108)
Survey month (ref – Nov 2020 – Jan 2021)						
March – May 2021	-0.073	-0.062	-0.102	-0.056	-0.113	-0.110
	(0.095)	(0.108)	(0.104)	(0.101)	(0.112)	(0.095)
June – August 2021	-0.018	-0.012	-0.018	-0.028	-0.049	-0.026
	(0.090)	(0.099)	(0.099)	(0.089)	(0.097)	(0.079)
New COVID-19 cases per million, terciles						
Cases (middle tercile)		-0.022				0.023
		(0.065)				(0.057)
Cases (top tercile)		-0.009				-0.011
		(0.054)				(0.049)
Oxford stringency index terciles (ref. – bottom tercile)						
Stringency (middle tercile)			0.061			0.148
			(0.073)			(0.077)
Stringency (top tercile)			-0.005			0.028
			(0.060)			(0.059)
WHO excess deaths due to COVID-19 (ref.- bottom tercile)						
Excess deaths (middle tercile)				-0.110*		-0.123
				(0.050)		(0.080)
Excess deaths (top tercile)				-0.059		0.045
				(0.067)		(0.106)
Confidence in government index tercile (ref.- top tercile)						
Confidence in government (middle tercile)					0.073	0.105
					(0.054)	(0.064)
Confidence in government (top tercile)					0.016	0.055
					(0.131)	(0.135)
Constant	0.405**	0.411**	0.381*	0.529***	0.331*	0.333*
	(0.138)	(0.143)	(0.146)	(0.142)	(0.157)	(0.153)
R-squared	0.090	0.090	0.095	0.097	0.102	0.123
N	65088	65088	65088	65088	65088	65088

Notes: Weighted OLS regressions. Marginal effects reported. Standard errors clustered at country level. Ref=reference group; EAP= East Asia and the Pacific; ECA= Europe and Central Asia; LAC=Latin America and the Caribbean; MNA= Middle East and North Africa; SSA= Sub-Saharan Africa; LIC=Low-income countries; LMIC=Lower middle-income countries; UMIC=Upper middle-income countries.*, **, *** indicates significance at the 95%, 99%, and 99.9% level.

In the extended regression models, we tested two additional hypotheses. First, we expected that the severity of the pandemic (for which we use three different proxies: number of new cases of COVID-19 in the country in column 2, the stringency of policy measures as captured by the Oxford Stringency index in column 3, and estimated COVID-19 related excess deaths in column 4) would be negatively correlated with hesitancy. In our data, none of the three proxies for pandemic severity was statistically associated

For peer review only

with vaccine hesitancy exceptfor COVID-19 excess deaths, where in countries in the second tercile of the excess deaths distribution vaccine hesitancy was, on average, lower, relative to the bottom tercile baseline. We also tested whether trust in the government in general was associated with hesitancy, but this association was not statistically significant (column 5). We also considered a model with vaccine hesitance as an ordinal variable in case the underlying characteristics of those who answered “No” and “Not sure” differed. The pattern of the results was similar, but the associations were weaker for the “Not sure” group (supplemental table S5b).

3d. Changes in vaccine hesitancy over time

In the 14 countries with estimates for two or more survey rounds (figure 3 and supplemental table S6), there were no clear patterns in terms of the changes in vaccine hesitancy. COVID-19 vaccine hesitancy declined in half of the countries and increased in the other half, and changes in either direction were less than five percentage points in all but three countries (Iraq, Malawi, Uzbekistan). No patterns of change over time were observed across sub-populations either.

3e. Reasons for COVID-19 vaccine hesitancy

Overall, the most common concern pertained to the safety of the vaccine, including concerns about side effects (43% of respondents in counties with the single answer option) (figure 4). The second most common concern related to efficacy (19%) and all other reasons accounted for less than 10 percent overall. These averages mask significant variation across countries. Concerns about vaccine efficacy were greater or equal to concerns about safety in five Latin American countries (Argentina, Chile, Dominica, Dominican Republic, and Mexico). Respondents in the Democratic Republic of Congo and the Republic of Congo were mainly hesitant because of dislike for vaccines in general and (distrust of the international community). In Georgia, the only country in Europe and Central Asia with information on reasons for hesitancy, the most cited reason was “not eligible” (supplemental figure S4). Only one country, the Republic of Congo, reported reasons for two waves, and only small changes in reasons were observed.

4. Discussion

4a. Principal findings

On average across 53 countries, one in five adults were hesitant about getting a COVID-19 vaccine. The highest levels were observed in Eastern European and Central Asian countries and the lowest levels in Latin American and Caribbean countries. Female respondents, younger adults, and those with less formal education reported higher levels of COVID-19 vaccine hesitancy than their respective counterparts. Between October 2020 and August 2021, there tended to be little change in levels of hesitancy except in Iraq, Malawi, and Uzbekistan, where hesitancy increased. The main self-reported reason for being hesitant was concerns over safety, especially worries about side effects.

4b. Strengths and comparisons with other studies

This study has several advantages over existing literature. It is based on a very large sample of respondents from national surveys in 53 low- and middle-income countries, a part of the world that is under-represented in the literature. Over half of the surveys used random digit dialing, typically in upper middle-income countries in which a large share of the population uses mobile phones. In lower income contexts, most surveys, 35 percent overall, sampled based on previous face-to-face surveys which were in turn

For peer review only

drawn from a census frame. In the remaining 10 percent of cases, sampling was carried out from lists provided by mobile phone operators. In each case, surveys drawn from pre-existing face-to-face surveys were reweighted using the baseline data to become more representative, while many random digit dialing surveys were also reweighted to make those samples more representative of the universe of phone numbers. While it is impossible to eliminate issues of representativeness in phone surveys, the HFPSs were typically carried out by National Statistics Offices and are more plausibly representative than convenience web surveys.

Overall, the estimates of COVID-19 vaccine hesitancy from the HFPSs were more conservative than other published estimates (supplemental figures S5). There are several possible explanations for the differences. The first relates to the timing of data collection. Most of the existing literature is based on surveys carried out in mid to late 2020 (14, 15, 17, 19-20), whereas the HFPS data included in our sample were collected from end-2020 through August 2021. It is plausible to believe COVID-19 vaccine hesitancy generally declined during this period as multiple vaccines became available, and widespread vaccination was safely rolled out in high-income countries. Another possible reason for the differences is the variation in how the vaccine hesitancy question was framed and the response options available. For example, some studies used a four- or five-point Likert scale of agreement (14-16, 20, 27), whereas the HFPS relied on two or three answer options. Finally, variations in survey modality may have created biases. Among the published studies included for comparison, many studies were based on data from commercial online sample providers, often using quotas sampling to ensure an appropriate distribution in terms of gender, age, and region (14-17,20, 27), while others were based on convenience samples (11, 28-30), or a mix of methods (19, 21). There was also wide variety in the survey mode, including online, computer-assisted telephone, and face-to-face surveys.

It is not possible to know the extent to which each of the above-mentioned potential sources of bias affect the comparisons of the findings from the HFPS with the others. For example, it was possible to match the Facebook survey results for the month and year of the HFPS for 20 countries, thus taking away any differences in the timing of the survey. Nevertheless, the HFPS estimates were still lower than those reported by Facebook (supplemental figure S5, panel D), and this could be due to differences in question-and-answer wording and/or the sample frame.

Even though the HFPS estimates are lower than other sources, the levels of COVID-19 vaccine hesitancy reported here are nonetheless higher than the levels of vaccine hesitancy reported for childhood vaccines. We compared the average country levels of COVID-19 vaccine hesitancy from this study with results from 2019 Global Monitor for 45 countries with observations in both studies (31). Overall, the levels of COVID-19 vaccine hesitancy reported here are higher than concerns about vaccines in general (supplemental figure S6). However, there are also similarities in the pattern: countries in Europe and Central Asia and some Sub-Sahara African countries reported the highest levels of vaccine hesitancy even before the pandemic, and the biggest reason for pre-COVID-19 vaccine hesitancy was concerns about safety.

Nevertheless, the higher levels of hesitancy reported for the COVID-19 vaccine suggest that respondents are more concerned about the COVID-19 vaccine than about childhood vaccines. The result is even more striking considering that the COVID-19 vaccine hesitancy is estimated rather conservatively in the HFPS, when compared against estimates from other sources. Possible reasons for the higher rates of COVID-19 vaccine hesitancy include the newness of a vaccine that employs an innovative technology

(mRNA), it's rapid development and streamlined approval process, and unknown long-term effects. This is consistent with the reasons given by respondents, which are mainly around safety.

Our findings that women, younger adults, and those with less education are more vaccine hesitant are largely consistent with results presented elsewhere (15, 19). Although Lazarus and colleagues found that men were slightly more hesitant than women, the gender difference was small (14). The findings with respect to age were also similar (14-15). The low levels of vaccine hesitancy reported among the older adults provide an important opportunity for vaccine campaigns to target this demographic, especially given recent research that targeting vaccines to older age groups saves the most lives and is highly cost-effective (32-33). The findings on education are also consistent with other studies (14-15), although Solis-Arce and colleagues reported mixed results (19). Knowing that citizens who are younger and with less formal education are the most likely to be vaccine hesitant can help vaccination campaigns target these population groups. Although we hypothesized that confidence in the government in general would be negatively associated with vaccine hesitancy, this association was not statistically significant in the regression analysis. This is likely because trust indicators were only available at the national level, making the estimates imprecise. Previous studies have shown mixed results (14-15, 18, 21), and future analysis is needed to investigate this relationship, preferably using an individual level measure of trust in government.

Our results showed that changes over time were minimal in all countries except Iraq, Malawi, and Uzbekistan, where we report relatively large increases. It is difficult to identify the factors that led to increased hesitancy in these cases. The larger body on changes over time in vaccine hesitancy during the same timeframe as the HFPS data were collected is mostly from high-income countries and showed mixed results (34-38). Furthermore, this period was relatively short.

The main reasons reported in the HFPS regarding COVID-19 vaccine hesitancy, which mainly revolve around safety and to a lesser degree efficacy suggest that factors in the 'thinking and feeling' domain of the BeSD framework were predominant (8). This is consistent with the few studies that report reasons for COVID-19 vaccine hesitancy in low- and middle-income countries and with the main reasons for vaccine hesitancy historically (16, 19, 31).

The variation in COVID-19 vaccine hesitancy across regions, and especially in Eastern Europe and Central Asia where rates are highest, is not fully explained by results in this study. Self-reported reasons for being hesitant were only available for one country in Eastern Europe and Central Asia (Georgia). Although our findings from Georgia that counterindication was a common barrier to vaccination are consistent with results published elsewhere (39), more research is needed to understand differences in vaccine hesitancy across regions.

Although concerns have been raised about supply constraints being the greatest barrier to COVID-19 vaccination scale-up in low- and middle-income countries, our study did not find this to be a major concern for most respondents. However, with only 1 in 5 individuals in low-income countries vaccinated to date, there was still a large share of the population who would get vaccinated if vaccines were made available to them, and alleviating access constraints will remain a policy priority in such contexts.

For peer review only

4c. Limitations of the study

The high-frequency surveys used in this analysis were designed to be nationally representative; however, there are potential limitations to phone surveys (40-41). Phone surveys exclude respondents without access to a phone. This method of data collection was necessary to collect information quickly during the early months of the COVID-19 pandemic while respecting local movement restrictions and minimizing the risk of COVID-19 transmission. Given that having a phone may be non-random, this might have created a risk that the results are representative only of the population with access to a phone. However, in the countries in this sample, access to mobile phones was high, and in countries where the sample was based on an existing nationally representative (pre-pandemic) survey, sampling weights were used in the analysis to correct for the biases resulting from non-random access to phones. Studies using phone survey from Africa also included in our sample have shown that although phone survey respondents tended to be older and better educated, the weighting procedures successfully minimized the selection bias in the phone surveys for a wide range of indicators (42-43). Another study on labor market participation during COVID-19 using the same phone survey data found that reweighting did not alter the main results (44).

Another limitation is that the majority of the countries are located in two regions -- Latin America and the Caribbean (45% of countries in sample) and Sub-Saharan African (26%), making results less representative of other regions.

The uneven rollout of the COVID-19 vaccine, which occurred earlier in higher-income countries than in lower-income countries, may have affected the reported levels of hesitancy. Most of the survey results reported here were collected after rollout began in high-income countries but before they were locally available, and this might have led to higher levels of hesitancy than post-rollout. In addition, influential events occurred during the data collection period, including news about adverse side effects of certain vaccines, which could have changed respondents' intentions. The type of vaccine or its country of origin available or anticipated to become available in each country may have influenced levels of hesitancy.

5. Conclusions

Between late 2020 and the first half of 2021, the level of COVID-19 vaccine hesitancy was on average about 20 percent across 53 low- and middle-income countries. Although less prevalent than previously thought, COVID-19 vaccine hesitancy was higher than levels of hesitancy reported towards other vaccines, indicating the challenges in scaling up COVID-19 vaccination campaigns may be even greater than for other diseases.

This study provides a rich set of results which can aid policy makers in the design of vaccination campaigns in low- and middle-income countries. Overall, knowing that hesitancy is less prevalent than previous estimates may bolster the case for expanding vaccination interventions. Specifically, knowing that people over the age of 65 years are the least likely to be hesitant provides an important rationale for scaling up vaccine rollout in this population group, which is at highest risk of severe disease and mortality from COVID-19. In many countries, a sizeable share of the hesitant reported being "not sure" about getting vaccinating (rather than definitely not willing), likely providing greater opportunity to influence vaccination decisions in these countries. In addition, the findings suggest the importance of designing vaccination campaigns that address concerns about safety, especially about side-effects and effectively reach the most hesitant, including women, younger adults, and less educated adults.

For peer review only

Contributors All authors had full access to all the data in the study and take responsibility for the integrity of the data and the accuracy of the data analysis. All authors were responsible for its conception and design. IE, AC and GD were responsible for the preparation and analysis of the data and JDE, DN, AC, YK, JM and JK contributed to their interpretation. JDE drafted the manuscript, and AC, DN and YK made critical revision of the manuscript for important intellectual content. The corresponding author attests that all listed authors meet authorship criteria and that no others meeting criteria have been omitted.

Competing interests None declared.

Funding. This paper was funded by the World Bank (Project AA-P177656-GPLG-BB) and the Bill and Melinda Gates Foundation through the Umbrella Facility for Poverty and Equity (TF0B7242), with support from the Global Financing Facility for Women, Children, and Adolescents (GFF).

Ethics approval statement: This study involves human participants. Each phone survey was implemented by the respective national statistical office or, in a few cases, a private firm. Informed consent was received from all survey respondents in each country. The World Bank does not require institutional ethics approval for household surveys that are partly or fully financed by the World Bank, including these national phone surveys that inform our research.

Data availability statement Data for most countries are available in a public, open access repository available from the World Bank's Microdata Library, High Frequency Phone Survey Catalog (https://microdata. Worldbank.org/index.php/catalog/hfps).

For peer review only

References

(1) Dong E, Du H, Gardner L. An interactive web-based dashboard to track COVID-19 in real time. Lancet Infect Dis 2020; 20(5): 533-4. https://coronavirus.jhu.edu/ accessed March 1, 2022.

(2) World Bank. 2022. Global Economic Prospects, January 2022. Washington, DC: World Bank. Doi: 10.1596/978-1-4648-1758-8

(3) International Monetary Fund. 2021. World Economic Outlook Update. July 2021. Fault Lines Widen in the Global Recovery.

(4) Hoogeveen, JG and G. Lopez-Acevedo, eds. 2021. Distributional Impacts of COVID-19 in the Middle East and North Africa Region. MENA Development Report;. Washington, DC: World Bank. © World Bank. https://openknowledge.worldbank.org/handle/10986/36618 License: CC BY 3.0 IGO.

(5) Ritchie, H., E. Mathieu, L. Rodés-Guirao, et al.2020. – "Coronavirus Pandemic (COVID-19)". Published online at OurWorldInData.org. Retrieved from: 'https://ourworldindata.org/coronavirus' on March 8, 2022.

(6) MacDonald, NE, and SAGE Working Group on Vaccine Hesitancy. Vaccine hesitancy: Definition, Scope, and determinants. Vaccine. Volume 33, Issue 34, 2015, Pages 4161-4164 https://doi.org/10.1016/j.vaccine.2015.04.036

(7) World Health Organization. 2019. Ten threats to global health in 2019. https://www.who.int/newsroom/spotlight/ten-threats-to-global-health-in-2019.

(8) Brewer, N.T., Chapman, G.B., Rothman, A.J., Leask, J. and Kempe, A., 2017. Increasing vaccination: putting psychological science into action. *Psychological Science in the Public Interest*, *18*(3), pp.149-207.

(9) Gramacho WG, Turgeon M. 2021. When politics collides with public health: COVID-19 vaccine country of origin and vaccination acceptance in Brazil. Vaccine. May 6;39(19):2608-2612. Doi: 10.1016/j.vaccine.2021.03.080.

(10) Bucci EM, Berkhof J, Gillibert A, Gopalakrishna G, Calogero RA, Bouter LM, Andreev K, Naudet F, Vlassov V. Data discrepancies and substandard reporting of interim data of Sputnik V phase 3 trial. Lancet. 2021 May 22;397(10288):1881-1883.

(11) Wong, L.P., Alias, H., Danaee, M., Ahmed, J., Lachyan, A., Cai, C.Z., Lin, Y., Hu, Z., Tan, S.Y., Lu, Y. and Cai, G., 2021. COVID-19 vaccination intention and vaccine characteristics influencing vaccination acceptance: a global survey of 17 countries. Infectious diseases of poverty, 10(1), pp.1-14.

(12) Husby, A. and Køber, L., 2022. COVID-19 mRNA vaccination and myocarditis or pericarditis. The Lancet, 399(10342), pp.2168-2169.

(13) Robinson, E., Jones, A., Lesser, I. and Daly, M., 2021. International estimates of intended uptake and refusal of COVID-19 vaccines: A rapid systematic review and meta-analysis of large nationally representative samples. Vaccine.

(14) Lazarus, JV, Ratzan SC , and Palayew A et al. 2021. A global survey of potential acceptance of a COVID-19 vaccine. Brief Communication. Nature Medicine 27: 225–228. https://doi.org/10.1038/s41591-020-1124-9.

(15) de Figueiredo A, Larson HJ. Exploratory study of the global intent to accept COVID-19 vaccinations. Communications medicine. 2021 Sep 9;1(1):1-0.

(16) Facebook. 2021. COVID-19 Trends and Impact Survey.

For peer review only - http://bmjopen.bmj.com/site/about/guidelines.xhtml

(17) Gallup. 2021. Gallup World Poll. https://www.gallup.com/178667/gallup-world-poll-work.aspx.

(18) Kanyanda, S., Markhof, Y., Wollburg, P. and Zezza, A., 2021. Acceptance of COVID-19 vaccines in sub-Saharan Africa: Evidence from six national phone surveys. BMJ open, 11(12), p.e055159.

(19) Solís Arce, J.S., Warren, S.S., Meriggi, N.F. et al. COVID-19 vaccine acceptance and hesitancy in low- and middle-income countries. Nat Med **27,** 1385–1394 (2021). https://doi.org/10.1038/s41591-021-01454-y.

(20) Wouters, O.J., Shadlen, K.C., Salcher-Konrad, M., Pollard, A.J., Larson, H.J., Teerawattananon, Y. and Jit, M., 2021. Challenges in ensuring global access to COVID-19 vaccines: production, affordability, allocation, and deployment. The Lancet, 397(10278), pp.1023-1034.

(21) Sallam M. COVID-19 Vaccine Hesitancy Worldwide: A Concise Systematic Review of Vaccine Acceptance Rates. Vaccines (Basel). 2021 Feb 16;9(2):160. Doi: 10.3390/vaccines9020160. PMID: 33669441; PMCID: PMC7920465.

(22) World Bank. 2020. High Frequency Mobile Phone Surveys of Households to Assess the Impacts of COVID-19: Overview (English). Washington, D.C.: World Bank Group. http://documents.worldbank.org/curated/en/703571588695361920/Overview

(23) [dataset] World Bank. 2020. High Frequency Mobile Phone Surveys Microdata. https://microdata.worldbank.org/index.php/catalog/hfps/?page=1&ps=15&repo=hfps.

(24) [dataset] Hale T, Angrist N, Goldszmidt R, et al. 2022. A global panel database of pandemic policies (Oxford COVID-19 government response Tracker). Nat Hum Behav 2021;5:529–38. Database accessed Feb 28, 2022.

(25) [dataset] Inglehart, R, C. Haerpfer, A. Moreno et al. 2022. Would Values Survey: Round Seven – Country Pooled Datafile Version: https://www.worldvaluessurvey.org/WVSOnline.jsp. (accessed April 21, 2022) Madrid: JD Systems Institute.

(26) [dataset] World Health Organization. 2022. Global Excess Deaths Associated with COVID-19 (Modelled Estimates). Downloaded on September 8, 2022 at https://www.who.int/data/sets/global-excess-deaths-associated-with-covid-19-modelled-estimates.

(27) Africa CDC. 2021. COVID-19 vaccine perceptions: a 15-country study, 2021. Available: https://africacdc.org/download/covid-19-vaccineperceptions-a-15-country-study/ [Accessed 18 May 2021].

(28) Anjorin, A.A., Odetokun, I.A., Abioye, A.I., Elnadi, H., Umoren, M.V., Damaris, B.F., Eyedo, J., Umar, H.I., Nyandwi, J.B., Abdalla, M.M. and Tijani, S.O., 2021. Will Africans take COVID-19 vaccination? PloS one, 16(12), p.e0260575.

(29) Faezi, N.A., Gholizadeh, P., Sanogo, M., Oumarou, A., Mohamed, M.N., Cissoko, Y., Sow, M.S., Keita, B.S., Baye, Y.A.M., Pagliano, P. and Akouda, P., 2021. Peoples' attitude toward COVID-19 vaccine, acceptance, and social trust among African and Middle East countries. Health Promotion Perspectives, 11(2), p.171.

(30) Bono, S.A., Faria de Moura Villela, E., Siau, C.S., Chen, W.S., Pengpid, S., Hasan, M.T., Sessou, P., Ditekemena, J.D., Amodan, B.O., Hosseinipour, M.C. and Dolo, H., 2021. Factors affecting COVID-19 vaccine acceptance: an international survey among low-and middle-income countries. Vaccines, 9(5), p.515.Gallup. 2019. Wellcome Global Monitor – First Wave Findings. https://wellcome.org/reports/wellcome-global-monitor/2018#downloads-4d1c.

For peer review only

(31) Gallup. 2019. Wellcome Global Monitor – First Wave Findings. https://wellcome.org/reports/wellcomeglobal-monitor/2018#downloads-4d1c.

(32) Cheikh, N., J. Spitz and D. Wilson. 2022. Increase Vaccine Targeting and Uptake: lessons learned from Global COVID-19 Vaccine Implementation. Considerations for World Bank Task Teams Managing COVID-19 Fast Track Facility Operations. Washington, D.C.: World Bank Group.

(33) Orangi S, Ojal J, Brand SP, et al. 2022. Epidemiological impact and cost-effectiveness analysis of COVID-19 vaccination in Kenya. BMJ Global Health **7:**e009430.

(34) To QG, Stanton R, Khalesi S, Williams SL, Alley SJ, Thwaite TL, Fenning AS, Vandelanotte C. Willingness to Vaccinate against COVID-19 Declines in Australia, Except in Lockdown Areas. Vaccines (Basel). 2021 May 10;9(5):479. doi: 10.3390/vaccines9050479. PMID: 34068479; PMCID: PMC8150580.

(35) Lavoie K, Gosselin-Boucher V, Stojanovic J, Gupta S, Gagné M, Joyal-Desmarais K, Séguin K, Gorin SS, Ribeiro P, Voisard B, Vallis M, Corace K, Presseau J, Bacon S; iCARE Study Team. Understanding national trends in COVID-19 vaccine hesitancy in Canada: results from five sequential cross-sectional representative surveys spanning April 2020-March 2021. BMJ Open. 2022 Apr 5;12(4):e059411. doi: 10.1136/bmjopen-2021-059411. PMID: 35383087; PMCID: PMC8983402.

(36) Szilagyi PG, Thomas K, Shah MD, Vizueta N, Cui Y, Vangala S, Kapteyn A. National Trends in the US Public's Likelihood of Getting a COVID-19 Vaccine-April 1 to December 8, 2020. JAMA. 2020 Dec 29;325(4):396–8. doi: 10.1001/jama.2020.26419. Epub ahead of print. PMID: 33372943; PMCID: PMC7772743.

(37) Biasio LR, Bonaccorsi G, Lorini C, Mazzini D, Pecorelli S. Italian Adults' Likelihood of Getting COVID-19 Vaccine: A Second Online Survey. Vaccines (Basel). 2021 Mar 17;9(3):268. doi: 10.3390/vaccines9030268. PMID: 33802873; PMCID: PMC8002675.

(38) Sypsa V, Roussos S, Engeli V, Paraskevis D, Tsiodras S, Hatzakis A. Trends in COVID-19 Vaccination Intent, Determinants and Reasons for Vaccine Hesitancy: Results from Repeated Cross-Sectional Surveys in the Adult General Population of Greece during November 2020-June 2021. Vaccines (Basel). 2022 Mar 18;10(3):470. doi: 10.3390/vaccines10030470. PMID: 35335102; PMCID: PMC8950863.

(39) World Bank, UNIFEF and JICA 2021. Counterindication: now the key driver of COVID-19 vaccine hesitancy in Central Asia.

(40) Gourlay, S., Kilic, T., Martuscelli, A., Wollburg, P. and Zezza, A., 2021. High-frequency phone surveys on COVID-19: good practices, open questions. *Food Policy*, *105*, p.102153.

(41) Bundervoet, T., Dávalos, M.E. and Garcia, N., 2022. The short-term impacts of COVID-19 on households in developing countries: An overview based on a harmonized dataset of high-frequency surveys. *World development*, p.105844.

(42) Brubaker J, Kilic T, Wollburg P. 2021. Representativeness of individual-level data in COVID-19 phone surveys: Findings from SubSaharan Africa. PLoS ONE 16(11): e0258877. https://doi.org/10.1371/journal.pone.0258877

(43) Ambel AA, Mcgee KR, Tsegay AH. 2021. Reducing Bias in Phone Survey Samples: Effectiveness of Reweighting Techniques using Face-to-Face Surveys as Frames. World Bank: Washington, DC.

For peer review only

(44) Kugler, M., Viollaz, M., Duque, D., Gaddis, I., Newhouse, D.L., Palacios-Lopez, A. and Weber, M., 2021. How Did the COVID-19 Crisis Affect Different Types of Workers in the Developing World?. IZA Discussion Paper No. 14519, Available at SSRN: https://ssrn.com/abstract=3883795 or http://dx.doi.org/10.2139/ssrn.3883795

For peer review only

Figure Legends and Notes

Figure 1

Title: Share of households that were hesitant to be vaccinated against COVID-19 in 53 countries

Notes: Weighted estimates, with weights scales such that 53 countries are given equal weights, except for results by education which include only results from the 33 country surveys with information on the educational attainment of the respondent.

Figure 2

Title: Share of households that were hesitant to be vaccinated against COVID-19 (answering either "no" or "not sure"), by country

Notes: Weighted estimates, with weights scales such that 53 countries are given equal weights. Black bars indicate confidence intervals. Point estimates are given in supplemental table S3.

Source: Authors' calculations.

Figure 3

Title: Changes in levels of vaccine hesitancy in 14 countries, October 2020 to April 2021

Note: The shaded area around the line represents the confidence bands.

Source: Authors' calculations.

Figure 4

Title: Self-reported reason for vaccine hesitancy in countries with single answer response, HFPS

Notes: The X axis represents share of vaccine hesitant households. In some cases, the share is larger than 100% because multiple responses were allowed. For the construction of harmonized response categories, see supplemental table S2b. Results for countries will multiple answer options are shown in supplemental figure S4.

Source: Authors' calculations.

For peer review only

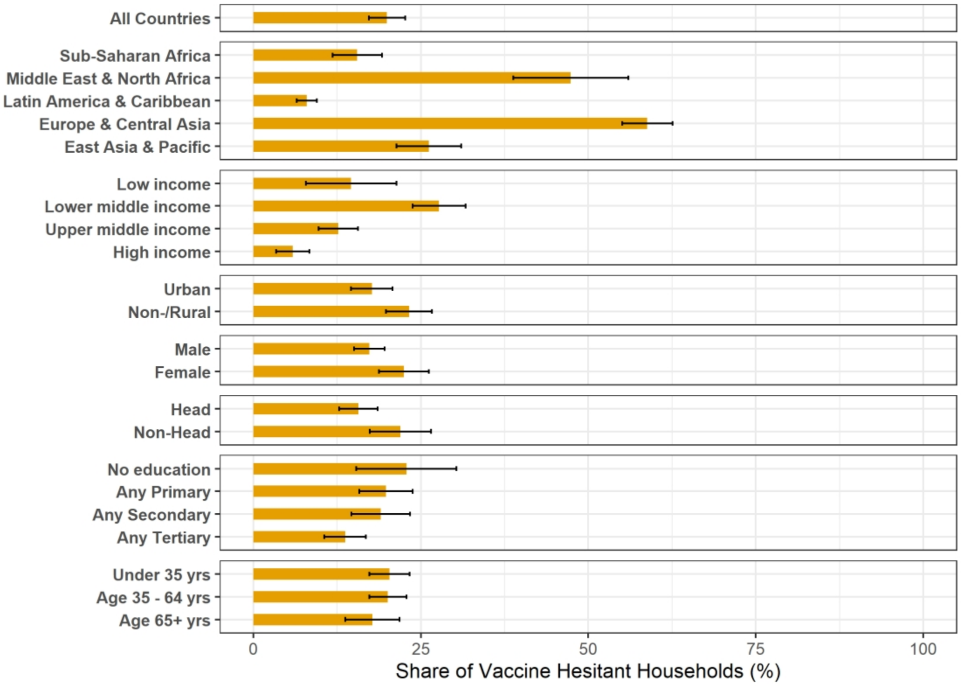

Figure 1: Share of households that were hesitant to be vaccinated against COVID-19 in 53 countries

490x342mm (118 x 118 DPI)

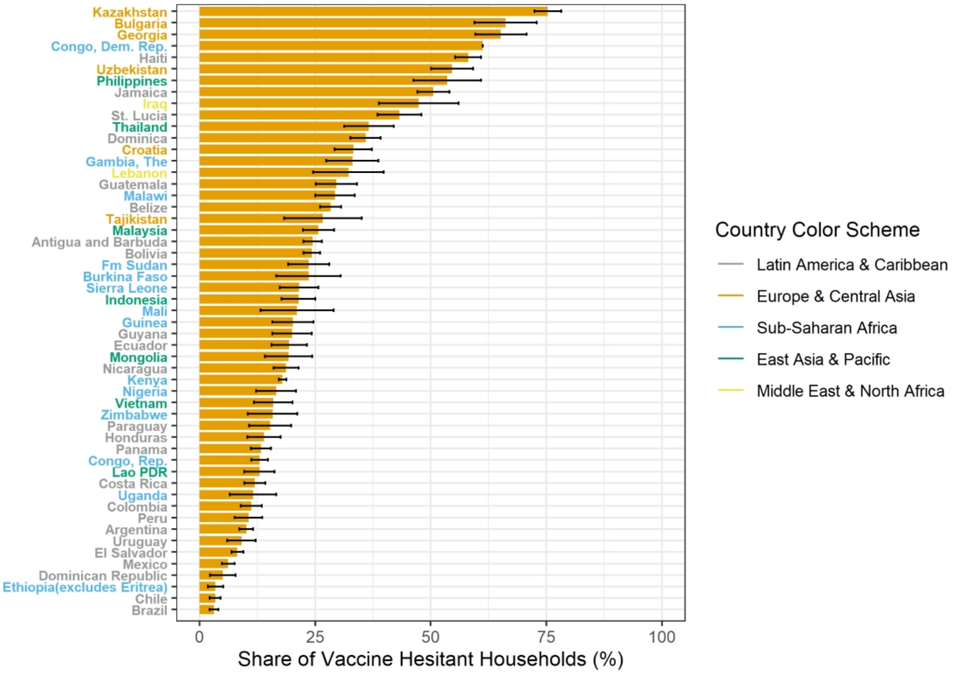

Share of households that were hesitant to be vaccinated against COVID-19 (answering either “no” or “not sure”), by country

490x336mm (118 x 118 DPI)

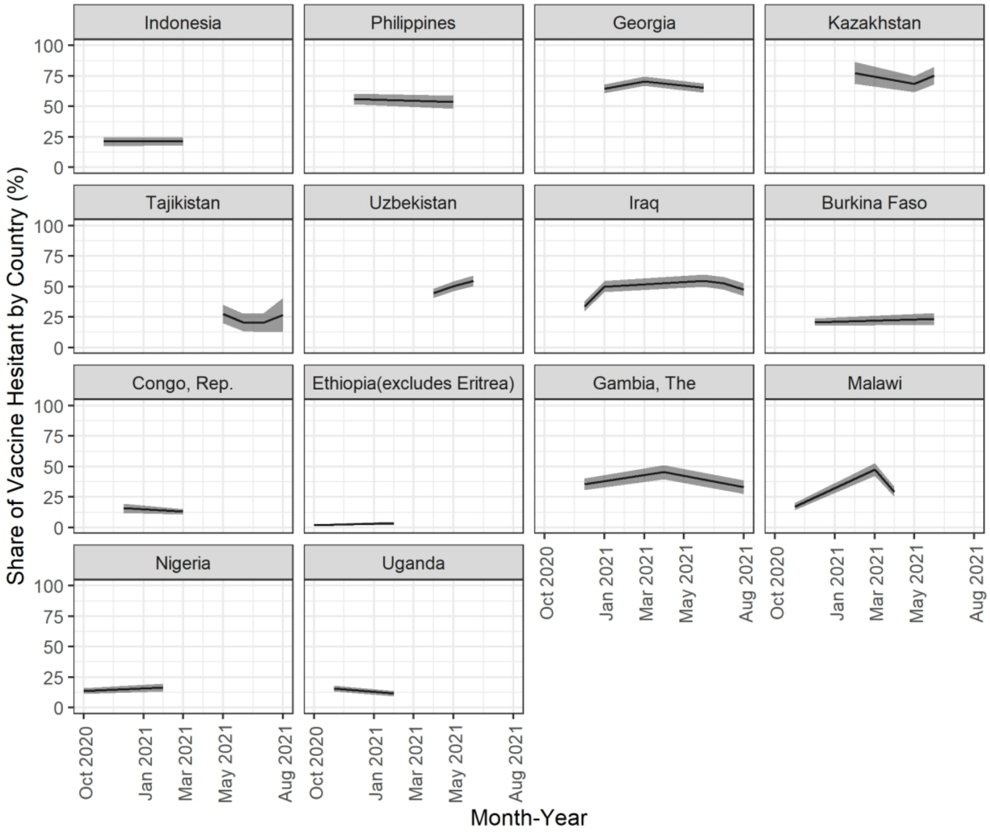

Figure 3: Changes in levels of vaccine hesitancy in 14 countries, October 2020 to April 2021

441x373mm (118 x 118 DPI)

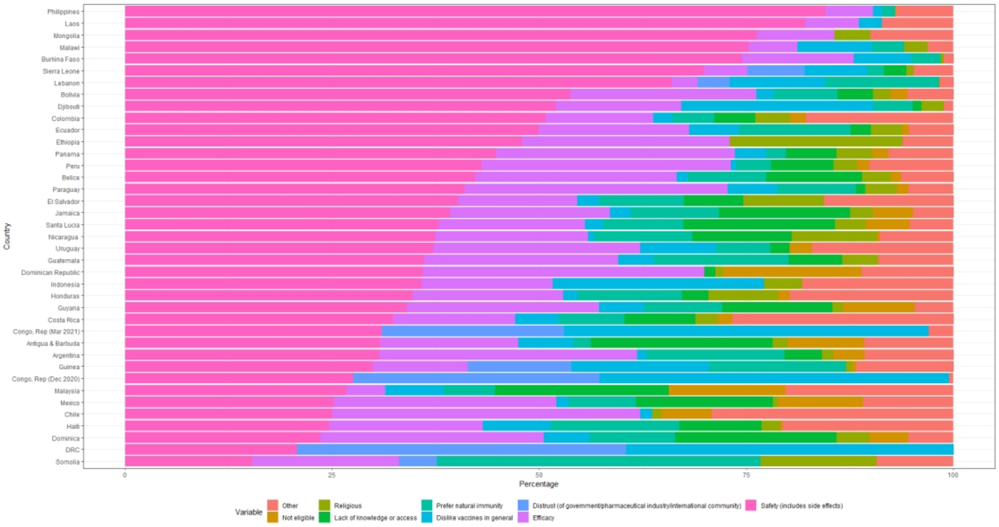

Self-reported reason for vaccine hesitancy in countries with single answer response, HFPS

1283x679mm (38 x 38 DPI)

Supplemental materials, COVID-19 Vaccine Hesitancy in 53 Countries

1. Supplements

Supplement 1: Detailed Description of Methods

2. Supplemental Tables

Supplemental Table S1: Description of 53 HFPS surveys

Supplemental Table S2a: Survey response options for COVID-19 vaccine hesitancy: distribution of countries by questionnaire type

Supplemental Table S2b: Survey response options for COVID-19 vaccine hesitancy: Harmonized answers by questionnaire type

Supplemental Table S3: Percent of population hesitant to take the COVID-19 vaccine, HFPS

Supplemental Table S4: Tukey's tests of significance of differences across groups in percent of population hesitant to take the COVID-19 vaccine

Supplemental Table S5a: Correlates of vaccine hesitancy (Logit model, marginal effects)

Supplemental Table S5b: Correlates of vaccine hesitancy (Ordinal logit regression, marginal effects)

Supplemental Table S6: Percent of population hesitant to take the COVID-19 vaccine, 14 countries with multiple rounds of survey results (95% confidence intervals in parentheses).

Supplemental Table S7: Comparison of results on vaccine hesitancy from the HFPS and other studies

3. Supplemental Figures

Supplemental Figure S1: Vaccination rates by income group

Supplemental Figure S2: Vaccine rollout rates by income group

Supplemental Figure S3: Comparison of share of population COVID-19 that is hesitant to take the COVID-19 vaccine, HFPS with other studies

Supplemental Figure S4: Comparison of COVID-19 Vaccine Hesitancy with pre-COVID sentiment about vaccines from 2019 Global Monitor, 45 countries

Supplemental Figure S5: Reason for "definitely not" (upper panel) or "probably not" (lower panel) taking the COVID-19 vaccine, Facebook data for month of HFPS)

4. References

For peer review only

For peer review only - http://bmjopen.bmj.com/site/about/guidelines.xhtml

Supplement 1: Detailed Description of Methods

Data

This analysis describes the levels of vaccine hesitancy and its reasons in 53 developing countries between October 2020 August 2021 using data from the World Bank's COVID-19 high-frequency phone surveys (HFPS) which were implemented to monitor the impact of COVID-19 on households around the world (World Bank 2020). Data are available for one survey round from 39 countries and two or more survey rounds for 14 countries. The countries in the pooled database represent five out of six regions defined by the World Bank: Latin America and the Caribbean (24 countries), Sub-Saharan Africa (14), East Asia and Pacific (7), Europe and Central Asia (6) and the Middle East and North Africa (2).[1] By income group, the sample includes 12 low-income countries, 13 lower-middle income countries, 23 upper middle-income countries and five high-income countries (Supplemental Table 1 provides a list of countries with survey month, sample size, region and country income group).

A global core questionnaire served as the basis for each country's survey but was then customized to fit the local context. As a result, the exact questions may vary across countries, but topics typically included knowledge and concerns about COVID-19, access to food, health care and education, employment and income loss, and safety nets and coping strategies. The survey data was then harmonized ex-post to enable cross-country comparability. The questionnaire was flexible and adapted over time to the pandemic context. The vaccine module, for example, was included more recently.

The sampling frame was drawn from pre-existing nationally representative household surveys in 19 countries, random digit dialing (RDD) in 29 countries, and a list of phone numbers typically obtained from mobile phone operators in 5 countries. The RDD design samples from all active mobile and landline phone numbers, such that RDD surveys are representative of the population aged 18 and over with an active phone number, conditional on response and survey completion. Sampling weights were then constructed to correct for selection bias due to the inability to contact households that did not participate in the survey either due to non-response or lack of access to a phone, with the goal of obtaining estimates that are as close to being nationally representative as possible. Information was collected from one respondent per household. In the case of countries where the sampling frame was derived from a previous survey, this was typically the household head. In other cases, such as when the sample utilized RDD, the respondent was more representative of individuals within the household. While the non-random selection of individuals in the former cases is not ideal, in an earlier analysis of labor market indicators derived from phone surveys, Kugler et al. (2021) find little evidence of differences in employment outcomes or trends over time being affected by the oversampling of household heads with respect to variables such as level of education, gender or location, the main source of bias being for age comparisons. Within the pooled data set, the household sampling weights are scaled such that countries are weighted equally, such that estimates of vaccine hesitancy reported in this study are averages of country averages.[2]

Outcome measures

The questions asked about vaccine hesitancy in the surveys varied depending on whether a vaccine was available in the country at the time of the survey. In surveys that took place before the vaccine rollout had begun in that country, the question asked was "*If an approved vaccine to prevent COVID-19 was to become*

[1] South Asia is not represented due to lack of available data. The Middle East and North Africa includes two countries—Iraq and Lebanon.

[2] Using population weights would have resulted in the results being driven by a small number of large countries.

For peer review only - http://bmjopen.bmj.com/site/about/guidelines.xhtml

available at no cost, would you agree to be vaccinated?" In surveys that took place after the vaccine rollout had begun, the wording of the question was either "*Are you planning to be vaccinated?*" or "*When a vaccine to prevent COVID-19 is available to you, are you planning to be vaccinated?"* In the sample, 47 countries offered three answer options (yes, not sure, and no), while the other six offered only two categories (yes and no). For this analysis, we combine the "no" and "not sure" answers to obtain the measure of vaccine hesitancy.

To obtain the respondent's reason for vaccine hesitancy, survey respondents who answered "no" or "not sure" were asked "*What is your (main) reason/concern for not wanting to be vaccinated / not being sure if you want to be vaccinated*?" The answer categories varied widely across surveys. To make these more comparable across countries, answers were remapped into the following nine most common categories (see Supplemental Tables 2a and 2b for the original answer options in each survey and a mapping of how they were harmonized):

1. Safety, which includes concerns about side effects
2. Efficacy
3. Distrust of government, pharmaceutical industry, international community
4. Dislike of vaccines in general
5. Preference for natural immunity, which included perceiving self as low risk and already having had COVID-19 infection
6. Lack of knowledge or access, which included "I do not have enough information about the vaccine," "too hard to get," "health facility to far" and "I don't have the time"
7. Religious reasons
8. Not eligible, which included "counter-indication," "recent medical discharge, "have an underlying health condition and believe taking the vaccine will make it worse"
9. Other

The surveys also differed across countries in terms of whether a single concern or multiple concerns were collected. To account for the difference in the number of response options, the results are presented separately.

Contextual data

Country-level contextual data were drawn from other sources. New COVID-19 cases per million, measured as a 7-day rolling average prior to the midpoint of the month household survey data was collected in each country, were obtained from Dong and colleagues (2022), and the Oxford Stringency Index was drawn from Hale et al. 2022. The latter is an index, ranging from 0–100, that indicates the degree to which various restrictions were put in place by governments to control the pandemic (for example, school closures and shelter-in-place requirements). Confidence in the press, the government, and the WHO was drawn from the World Values Survey Wave 7, fielded in 2017-2020, and aggregated up to the country level (Inglehart 2022). Excess deaths due to COVID-19 draw on WHO data (WHO 2022). Country geographic region and income group are based on the World Bank classifications.

Analytical strategy

Data from 53 countries was pooled into a single data set. First, we report point estimates and 95% confidence intervals for each country based on standard errors clustered at the state or province level within each country. We also report the simple average across all countries and stratified by: (i) World Bank region, (ii) World Bank country income group, (iii) urban vs. rural residence, (iv) gender of the respondent, (v) whether the respondent is head of household, (vi) age of the respondent (under 35 years,

For peer review only - http://bmjopen.bmj.com/site/about/guidelines.xhtml

35-64 years, and 65 years and older, and (vii) educational attainment of the respondent (some primary school, some secondary school, some tertiary). Tukey's test of multiple comparisons is used to test the significance of differences in rates of vaccine hesitancy across the above-mentioned groups. Second, we use multivariate regression analysis to assess the relative association of vaccine hesitancy and the correlates and contextual variables. Trends over time in levels of vaccine hesitancy are examined in 14 countries with more than one wave of results available. Finally, the analysis describes the reasons for vaccine hesitancy.

For peer review only

For peer review only - http://bmjopen.bmj.com/site/about/guidelines.xhtml

Supplemental Table S1: Description of 53 HFPS surveys

Country	Survey Months	Total Number of households	Sample frame
**Antigua and Barbuda**	06/2021	790	RDD
**Argentina**	06/2021	1216	RDD
**Belize**	06/2021	816	RDD
**Bolivia**	05/2021	1272	RDD
**Brazil**	08/2021	2166	RDD
**Bulgaria**	07/2021	1000	RDD
**Burkina Faso**	12/2020	1944	Previous survey
**Chile**	06/2021	1212	RDD
**Colombia**	06/2021	1221	RDD
**Congo, Dem. Rep.**	12/2020	986	Previous Survey
**Congo, Rep.**	12/2020, 03/2021	578, 1495	Previous Survey
**Costa Rica**	06/2021	802	RDD
**Croatia**	03/2021	1217	Non-survey list
**Dominica**	06/2021	861	RDD
**Dominican Republic**	06/2021	1205	RDD
**Ecuador**	05/2021	1352	RDD
**El Salvador**	06/2021	816	RDD
**Ethiopia**	10/2020, 02/2021	2704, 2178	Previous Survey
**The Gambia**	12/2020, 04/2021, 08/2021	1334, 1287, 1059	Previous Survey
**Georgia**	01/2021, 03/2021, 06/2021	2033, 2100, 1936	RDD
**Guinea**	11/2020	1334	Previous Survey
**Guatemala**	06/2021	1206	RDD
**Guyana**	06/2021	785	RDD
**Haiti**	07/2021	2813	Non-Survey list
**Honduras**	07/2021	1021	RDD
**Indonesia**	11/2020, 03/2021	3953, 3555	Previous Survey
**Iraq**	12/2020, 01/2021,06/2021, 07/2021, 08/2021	1614,1651,1378, 1297,1141	Non-Survey list
**Jamaica**	06/2021	828	RDD
**Kazakhstan**	02/2021, 05/2021, 06/2021	917, 1732, 1610	Previous Survey
**Kenya**	03/2021	6730	Previous Survey

For peer review only - http://bmjopen.bmj.com/site/about/guidelines.xhtml

Country	Survey Months	Total Number of households	Sample frame
**Lao PDR**	03/2021	2153	RDD
**Lebanon**	03/2021	5113	RDD
**Mali**	01/2021	1884	Previous Survey
**Malawi**	11/2020, 03/2021, 04/2021	1589, 1549,1338	Previous Survey
**Malaysia**	06/2021	2210	RDD
**Mexico**	06/2021	2624	RDD
**Mongolia**	12/2020	1147	Previous Survey
**Nicaragua**	06/2021	833	RDD
**Nigeria**	02/2021, 10/2020	1699, 1762	Previous Survey
**Panama**	06/2021	815	RDD
**Paraguay**	06/2021	1076	RDD
**Peru**	06/2021	1210	RDD
**Philippines**	12/2020, 05/2021	1805, 2122	Non-survey list
**Saint Lucia**	06/2021	835	RDD
**Sierra Leone**	11/2020	1198	Previous Survey
**Sudan**	03/2021	2545	Non-Survey list
**Tajikistan**	05-08/2021	232	Previous Survey
**Thailand**	05/2021	1786	RDD
**Uganda**	11/2020, 02/2021	2135, 2121	Previous Survey
**Uruguay**	06/2021	816	RDD
**Uzbekistan**	04-06/2021	1496, 1356, 1300	Previous Survey
**Vietnam**	01/2021	3940	Previous Survey
**Zimbabwe**	12/2020	1227	Previous Survey

For peer review only

For peer review only - http://bmjopen.bmj.com/site/about/guidelines.xhtml

Supplemental Table S2a: Survey response options for COVID-19 vaccine hesitancy: distribution of countries by questionnaire type

**Questionnaire type 1**	**Questionnaire type 2**	**Questionnaire type 3**	**Questionnaire type 4**	**Questionnaire type 5**	**Questionnaire type 6**	**Questionnaire type 7**	**Questionnaire type 8**	**Questionnaire type 9**	**Questionnaire type 10**	**Questionnaire LAC**	**Questionnaire type 11**	**Questionnaire type 12**
Kenya	*Philippines*	*Lebanon*	*Congo, Democratic Republic*	*Guinea*	*Malaysia*	*Mali*	*Gambia, The*	*Lao PDR*	*Cambodia*	*Argentina*	*Kazakhstan*	*Croatia*
Thailand	*Indonesia*		*Republic of Congo*	*Sudan*		*Burkina Faso*		*Mongolia*		*Antigua and Barbuda*	*Kyrgyzstan*	
	Djibouti			*Sierra Leone*		*Uganda*				*Belize*	*Uzbekistan*	
						Ethiopia				*Bolivia*		
						Malawi				*Brazil*		
						Nigeria				*Chile*		
										Colombia		
										Costa Rica		
										Dominica		
										Dominican Republic		
										Ecuador		
										Guatemala		
										Guyana		
										Honduras		
										Haiti		
										Jamaica		
										Mexico		
										Nicaragua		
										Panama		
										Peru		
										Paraguay		
										El Salvador		
										Uruguay		

For peer review only - http://bmjopen.bmj.com/site/about/guidelines.xhtml

For peer review only

Supplemental Table S2b: Survey response options for COVID-19 vaccine hesitancy: Harmonized answers by questionnaire type

Answer categories	Quest type 1	Quest type 2	Quest type 3	Quest type 4	Quest type 5	Quest type 6	Quest type 7	Quest type 8	Quest type 9	Quest type 10	Quest LAC	Quest type 11	Quest type 12
**1. Efficacy**	I don't think it will work	I don't think COVID-19 vaccines effective/wo rk	I don't think coronavirus vaccines are effective/wo rk		I don't think vaccines work	I don't think it will work	I don't think it will work	I do not think the vaccine would work against COVID	I don't think COVID-19 vaccines effective/wo rk		I do not think they are effective, that they work		It may not work
						I don't think the vaccines available in my country are effective							
**2 Safety (includes side effects)**	I don't think it will be safe	I am worried about the safety of the vaccine		Safety of the vaccine	I am worried about the vaccine safety		I don't think it will be safe	I fear the unforeseen future negative side effects from the COVID-19 vaccine	I am worried about the safety of the vaccine	I don't think it is safe		The risk of vaccinating is higher than the benefits	Health risks
	I am worried about the side effects	I am worried about the side effects	I am worried about the side effects of the vaccine			I am worried about the side effects	I'm worried about side effects	I have heard the vaccine has negative side effects	I am worried about the side effects		I don't think it's safe, because of the side effects		
**3. Perceive self as low risk**	I am not enough at risk of contracting COVID-19	I am strong, I never got any disease in the past	I am not enough at risk of contracting coronavirus			I am not enough at risk of contracting COVID-19	I am not at enough risk of contracting covid-19	I believe I do not need to be vaccinated			I am not at risk of getting covid-19		I am not worried about COVID-19
**4.Dislike vaccines in general**	I am against vaccines in general	I am against vaccines in general	I am against vaccines in general	General distrust of vaccines	I don't trust the vaccines in general	In general, I don't trust vaccines	I am against the vaccine in general		I am against vaccines in general		I am against vaccines in general	Negative past experiences with vaccines	I am against vaccines in general
**5 Religious**	It is against my religion	Religious reasons	Religious reasons		I have religious reasons/con cerns	It is against my religion.	It's against my religion		Religious reasons		Religious reasons	Personal or religious beliefs	
		I am concerned about its											

For peer review only

Answer categories	Quest type 1	Quest type 2	Quest type 3	Quest type 4	Quest type 5	Quest type 6	Quest type 7	Quest type 8	Quest type 9	Quest type 10	Quest LAC	Quest type 11	Quest type 12
		halal certification											
**6 Concerned about getting covid at facility**	I'm worried to get infected with COVID-19 at the health facility	I am worried of getting infected with COVID-19 at the health facility	I am worried of getting infected with coronavirus at the health facility			I am worried to get infected with COVID-19 at the health facility.			I am worried to get infected with COVID-19 at the health facility		I am concerned of being infected with covid-19 at the health center		
**7 Supply /access barrier**	Health facility is too far or too hard to get to		Health facility too far or too hard to get to		It does not suit me (will be given too far or difficult to find)	Health facility too far or too hard to get to		There is shortage of vaccines in the country		I don't know how to access the vaccine	Health center too far or hard to reach		
	I don't have time to get vaccinated/ It will take too long to get vaccinated					It will take too long to get vaccinated/ I don't have time to get vaccinated		There is no vaccination center near my place		I am not the priority group to get the vaccine	I don't have time to go to get vaccinated		
**8 Prefer natural immunity (includes already had COVID-19)**			I already had coronavirus		I believe that the remedies natural or traditional	I already had COVID-19		I prefer to build immunity against COVID-19 naturally by having the disease			I already had covid-19 / I no longer need it		I already had COVID-19 and do''t need a vaccine
					It is better to leave nature take its course; the COVID symptoms are mostly light								

For peer review only

Answer categories	Quest type 1	Quest type 2	Quest type 3	Quest type 4	Quest type 5	Quest type 6	Quest type 7	Quest type 8	Quest type 9	Quest type 10	Quest LAC	Quest type 11	Quest type 12
**9 Distrust (of Government/pharmaceutical industry/international community)**				Be wary of international community	I do''t trust the government/ to the organization who gives the vaccine			I do not trust pharmaceutical companies				Lack of trust to producers of vaccines	I do''t trust the pharmaceutical industry
								I do not trust the government				Lack of trust to heath system or healthcare providers	I do''t trust the government
								I do not trust the COVID-19 vaccines					
								I heard the vaccine is meant to control population growth					
								I do not believe in COVID-19					
**10 Not eligible / conterindication**								I have underlying health conditions and I believe taking the vaccine will make it worse		I am not eligible to get the vaccine			Counter-indication or prolonged medical discharge
**11 Lack of knowledge about COVID 19 Vaccine**								I do not have enough information about the vaccine					

For peer review only

Answer categories	Quest type 1	Quest type 2	Quest type 3	Quest type 4	Quest type 5	Quest type 6	Quest type 7	Quest type 8	Quest type 9	Quest type 10	Quest LAC	Quest type 11	Quest type 12
								I did not know that a vaccine exists against COVID-19					
**12 Other**	Other (specify)	Other (specify)	Some other reason	other	Others	Other (Specify)	other, specify)	Everyday stresses are overwhelming to think about getting vaccinated	Others (specify)	Other, specify	Other (specify)	Other	
		I will wait till more people are vaccinated											
		No one in my neighborhood got it				I am not sure I will get the vaccine I want							

For peer review only

Supplemental Table S3: Percent of population hesitant to take the COVID-19 vaccine, HFPS

Average across countries	Percent (%) hesitant to take the COVID-19 vaccine	Confidence Intervals
All countries	20.0	[17.24 , 22.67]
East Asia & Pacific	26.2	[21.38 , 31.02]
Europe & Central Asia	58.8	[55.04 , 62.56]
Latin America & Caribbean	8.0	[6.45 , 9.47]
Middle East & North Africa	47.4	[38.80 , 55.96]
Sub-Saharan Africa	15.5	[11.81 , 19.19]
Low income countries	14.6	[7.82 , 21.39]
Lower middle income countries	27.7	[23.78 , 31.65]
Upper middle income countries	12.7	[9.77 , 15.61]
High income countries	5.9	[3.40 , 8.36]
Urban	17.6	[14.60 , 20.75]
Rural	23.2	[19.83 , 26.64]
Male	17.3	[15.02 , 19.63]
Female	22.5	[18.77 , 26.21]
Head of household	10.8	[12.82 , 18.53]
Non-Head of household	9.4	[17.37 , 26.54]
No education	22.8	[15.36 , 30.31]
Any Primary	19.8	[15.80 , 23.78]
Any Secondary	19.0	[14.64 , 23.42]
Any Tertiary	13.7	[10.56 , 16.83]
Age 34 and younger	20.3	[17.29 , 23.34]
Age 35— 64	20.1	[17.34 , 22.84]
Ages 65 and older	17.8	[13.70 , 21.80]
Antigua and Barbuda	24.5	[22.51 , 26.49]
Argentina	10.1	[8.66 , 11.53]
Belize	28.4	[26.10 , 30.64]
Bolivia	24.3	[22.47 , 26.04]
Brazil	3.1	[2.15 , 4.04]
Bulgaria	66.2	[59.48 , 72.90]
Burkina Faso	23.6	[16.60 , 30.53]
Chile	3.4	[2.22 , 4.57]
Colombia	11.2	[8.91 , 13.51]
Congo, Dem. Rep.	61.2	[61.23 , 61.23]
Congo, Rep.	13.0	[11.20 , 14.79]

For peer review only

For peer review only - http://bmjopen.bmj.com/site/about/guidelines.xhtml

Average across countries	Percent (%) hesitant to take the COVID-19 vaccine	Confidence Intervals
Costa Rica	11.9	[9.66 , 14.22]
Croatia	33.2	[29.21 , 37.25]
Dominica	35.9	[32.63 , 39.14]
Dominican Republic	5.0	[2.25 , 7.81]
Ecuador	19.4	[15.54 , 23.21]
El Salvador	8.2	[6.91 , 9.44]
Ethiopia (excludes Eritrea)	3.5	[1.81 , 5.09]
Fm Sudan	23.7	[19.19 , 28.11]
Gambia, The	33.1	[27.44 , 38.70]
Georgia	65.2	[59.62 , 70.72]
Guatemala	29.6	[25.09 , 34.05]
Guinea	20.2	[15.74 , 24.61]
Guyana	20.0	[15.75 , 24.23]
Haiti	58.1	[55.31 , 60.86]
Honduras	13.9	[10.31 , 17.51]
Indonesia	21.4	[17.71 , 25.07]
Iraq	47.4	[38.81 , 55.99]
Jamaica	50.6	[47.11 , 54.03]
Kazakhstan	75.3	[72.39 , 78.18]
Kenya	17.9	[17.11 , 18.74]
Lao PDR	13.0	[9.70 , 16.25]
Lebanon	32.2	[24.56 , 39.84]
Malawi	29.3	[25.03 , 33.54]
Malaysia	25.7	[22.37 , 29.10]
Mali	21.1	[13.14 , 29.03]
Mexico	6.2	[4.85 , 7.55]
Mongolia	19.3	[14.13 , 24.38]
Nicaragua	18.7	[16.04 , 21.40]
Nigeria	16.6	[12.27 , 20.84]
Panama	13.3	[11.12 , 15.49]
Paraguay	15.3	[10.71 , 19.79]
Peru	10.6	[7.55 , 13.59]
Philippines	53.6	[46.28 , 60.83]
Sierra Leone	21.5	[17.31 , 25.66]
St. Lucia	43.2	[38.46 , 47.93]
Tajikistan	26.7	[18.32 , 35.03]
Thailand	36.6	[31.25 , 42.00]
Uganda	11.6	[6.51 , 16.61]
Uruguay	9.1	[5.94 , 12.17]
Uzbekistan	54.6	[50.02 , 59.18]
Vietnam	15.9	[11.72 , 20.14]
Zimbabwe	15.8	[10.42 , 21.14]

For peer review only - http://bmjopen.bmj.com/site/about/guidelines.xhtml

For peer review only

Supplemental Table S4: Tukey's tests of significance of differences across groups in percent of population hesitant to take the COVID-19 vaccine

Group	Comparison	Difference	Std. Error	t-score	p-value	Signi-ficance
Gender	Female vs Male	0.052	0.022	2.316	0.021	**
Household Status	Head vs Non-Head	-0.063	0.026	-2.398	0.016	**
Age	Ages 65 and older vs Age 35 - 64	-0.023	0.025	-0.937	0.349	
	Ages 65 and older vs Age 34 and younger	-0.026	0.026	-0.994	0.320	
	Age 35 - 64 vs Age 34 and younger	-0.002	0.021	-0.107	0.543	
Education	Any Primary vs Any Secondary	0.008	0.030	0.250	0.599	
	Any Primary vs Any Tertiary	0.061	0.026	2.355	0.019	**
	Any Primary vs No Education	-0.030	0.043	-0.705	0.481	
	Any Secondary vs Any Tertiary	0.053	0.028	1.941	0.052	*
	Any Secondary vs No Education	-0.038	0.044	-0.860	0.390	
	Any Tertiary vs No Education	-0.091	0.041	-2.211	0.027	**
Sector	Rural vs Urban	0.056	0.023	2.375	0.018	**
Region	EAP vs ECA	-0.326	0.031	-10.450	0.000	***
	EAP vs LAC	0.182	0.026	7.077	0.000	***
	EAP vs MENA	-0.212	0.050	-4.219	0.000	***
	EAP vs SSA	0.107	0.031	3.454	0.001	***
	ECA vs LAC	0.508	0.021	24.579	0.000	***
	ECA vs MENA	0.114	0.048	2.390	0.017	**
	ECA vs SSA	0.433	0.027	16.103	0.000	***
	LAC vs MENA	-0.394	0.044	-8.870	0.000	***
	LAC vs SSA	-0.075	0.020	-3.705	0.000	***
	MENA vs SSA	0.319	0.048	6.691	0.000	***

For peer review only - http://bmjopen.bmj.com/site/about/guidelines.xhtml

Income Group	HIC vs LIC	-0.087	0.037	-2.368	0.018	**
	HIC vs LMIC	-0.218	0.024	-9.202	0.000	***
	HIC vs UMIC	-0.068	0.020	-3.481	0.001	***
	LIC vs LMIC	-0.131	0.037	-3.559	0.000	***
	LIC vs UMIC	0.019	0.024	0.808	0.419	
	LMIC vs UMIC	0.150	0.025	6.010	0.000	***

Significance levels: *** for p-values <= 0.01, ** for 0.01 < p-values <= 0.05, * for 0.05 < p-values <= 0.1

For peer review only

Supplemental Table S5a: Correlates of vaccine hesitancy (Logit model, marginal effects)

Dep. Var: Hesitancy	(1)	(2)	(3)	(4)	(5)	(6)
Male	-0.027*	-0.028*	-0.027*	-0.027*	-0.033**	-0.040***
	(0.013)	(0.013)	(0.013)	(0.012)	(0.012)	(0.011)
Head of HH	0.013	0.009	0.021	-0.001	0.014	0.007
	(0.014)	(0.014)	(0.013)	(0.016)	(0.015)	(0.012)
Education of respondent (ref – no education)						
No education	-0.062	-0.062	-0.055	-0.068*	-0.074*	-0.075*
	(0.032)	(0.032)	(0.031)	(0.034)	(0.036)	(0.035)
Any primary	-0.061	-0.060	-0.054	-0.072	-0.069	-0.086*
	(0.037)	(0.038)	(0.033)	(0.040)	(0.042)	(0.038)
Any secondary	-0.130***	-0.131***	-0.122***	-0.142***	-0.133**	-0.153***
	(0.036)	(0.037)	(0.033)	(0.040)	(0.044)	(0.040)
Age group (ref. -- 34 and younger)						
Working age (35-64)	-0.047***	-0.048***	-0.042**	-0.050***	-0.046**	-0.049***
	(0.014)	(0.013)	(0.015)	(0.013)	(0.015)	(0.012)
Retirement age (65+)	-0.103***	-0.102***	-0.096***	-0.101***	-0.103***	-0.102***
	(0.024)	(0.025)	(0.024)	(0.023)	(0.025)	(0.024)
Rural area	0.016	0.014	0.015	0.008	0.012	0.011
	(0.019)	(0.018)	(0.017)	(0.015)	(0.015)	(0.011)
Region (ref – LAC)						
EAP	0.189**	0.192*	0.222**	0.095	0.264*	0.188
	(0.067)	(0.078)	(0.077)	(0.069)	(0.130)	(0.130)
ECA	0.313***	0.315***	0.295***	0.328***	0.313***	0.295***
	(0.055)	(0.057)	(0.056)	(0.059)	(0.040)	(0.062)
MNA	0.208***	0.212***	0.245***	0.216***	0.282***	0.369***
	(0.037)	(0.040)	(0.062)	(0.032)	(0.059)	(0.051)
SSA	0.074	0.081	0.070	-0.022	0.047	0.022
	(0.075)	(0.077)	(0.070)	(0.105)	(0.083)	(0.104)
Income group (ref. – LIC)						
LMIC	-0.068	-0.065	-0.045	-0.088	-0.054	-0.071
	(0.122)	(0.119)	(0.112)	(0.123)	(0.097)	(0.069)
UMIC	-0.047	-0.038	-0.066	-0.095	-0.035	-0.137
	(0.149)	(0.148)	(0.130)	(0.150)	(0.116)	(0.087)
HIC	-0.173	-0.164	-0.190	-0.223	-0.202	-0.309***
	(0.163)	(0.160)	(0.143)	(0.164)	(0.130)	(0.090)
Survey month (ref -- Nov 2020 - Jan						
March - May 2021	-0.069	-0.060	-0.094	-0.056	-0.095	-0.083
	(0.095)	(0.108)	(0.103)	(0.098)	(0.104)	(0.095)
June - August 2021	-0.029	-0.025	-0.024	-0.052	-0.057	-0.055
	(0.088)	(0.098)	(0.100)	(0.087)	(0.092)	(0.075)
New COVID-19 cases per million, terciles (ref. – bottom tercile)						
Cases (middle tercile)		-0.017				0.026
		(0.067)				(0.060)
Cases (top tercile)		-0.019				-0.016
		(0.057)				(0.047)
Oxford stringency index terciles (ref. – bottom tercile)						
Stringency (middle tercile)			0.065			0.134
			(0.072)			(0.070)
Stringency (top tercile)			-0.008			0.027
			(0.076)			(0.068)
WHO excess deaths due to COVID-19 terciles (ref.- bottom tercile)						

For peer review only - http://bmjopen.bmj.com/site/about/guidelines.xhtml

Dep. Var: Hesitancy	(1)	(2)	(3)	(4)	(5)	(6)
Excess deaths (middle tercile)				-0.117*		-0.109
				(0.052)		(0.072)
Excess deaths (top tercile)				-0.069		0.040
				(0.072)		(0.108)
Confidence in government index tercile (ref.- top tercile)						
Confidence in government					0.048	0.070
					(0.053)	(0.057)
Confidence in government					0.024	0.053
					(0.097)	(0.103)
Pseudo R-squared	0.0745	0.0748	0.0794	0.0813	0.0869	0.1067
N	65088	65088	65088	65088	65088	65088

Notes: Weighted logit regressions. Marginal effects reported. Standard errors clustered at country level. Ref=reference group; EAP= East Asia and the Pacific; ECA= Europe and Central Asia; LAC=Latin America and the Caribbean; MNA= Middle East and North Africa; SSA= Sub-Saharan Africa; LIC=Low-income countries; LMIC=Lower middle-income countries, UMIC=Upper middle-income countries. *, **, *** indicates significance at the 95%, 99%, and 99.9% level.

For peer review only

Supplemental Table S5b: Correlates of vaccine hesitancy (Ordinal logit regression, marginal effects)

Dep. Var.:	Would you get the vaccine?		
	No	Not sure	Yes
Male	-0.025**	-0.007**	0.036**
	(0.01)	(0.002)	(0.011)
Head of HH	0.011	0.001	-0.006
	(0.012)	(0.002)	(0.011)
Education of respondent (ref – Tertiary)			
No education	0.119***	0.022**	-0.156***
	-0.028	(0.007)	(0.038)
Any primary	0.057**	0.013**	-0.080***
	-0.018	(0.004)	(0.022)
Any secondary	0.058***	0.012***	-0.068***
	(0.009)	(0.003)	(0.010)
Age group (ref. -- 34 and younger)			
Working age (35-64)	-0.040***	-0.009***	0.049***
	(0.010)	(0.002)	(0.012)
Retirement age (65+)	-0.079***	-0.018***	0.097***
	(0.020)	(0.005)	(0.025)
Rural area	0.010	0.002	-0.012
	(0.009)	(0.002)	(0.011)
Region (ref – LAC)			
EAP	0.149	0.034	-0.183
	(0.093)	(0.021)	(0.113)
ECA	0.238***	0.055***	-0.293***
	(0.044)	(0.016)	(0.058)
MNA	0.288***	0.066***	-0.354***
	(0.042)	(0.011)	(0.049)
SSA	0.015	0.004	-0.019
	(0.082)	(0.019)	(0.101)
Income group (ref. – LIC)			
LMIC	-0.074	-0.017	0.091
	(0.055)	(0.012)	(0.067)
UMIC	-0.109	-0.025	0.134
	(0.069)	(0.017)	(0.085)
HIC	-0.241***	-0.056**	0.297***
	(0.071)	(0.019)	(0.088)
Survey month (ref -- Nov 2020 - Jan 2021)			
March - May 2021	-0.054	-0.011	0.065
	(0.081)	(0.016)	(0.098)
June - August 2021	-0.046	-0.010	0.055
	(0.065)	(0.012)	(0.077)
New COVID-19 cases per million, terciles (ref. – top tercile)			
Cases (middle tercile)	0.016	0.003	-0.019
	(0.048)	(0.010)	(0.058)
Cases (bottom tercile)	-0.028	-0.006	0.034
	(0.037)	(0.008)	(0.045)
Oxford stringency index terciles (ref. – top tercile)			
Stringency (middle tercile)	0.089	0.020	-0.110
	(0.057)	(0.013)	(0.070)
Stringency (bottom tercile)	0.012	0.003	-0.015
	(0.041)	(0.011)	(0.052)

For peer review only - http://bmjopen.bmj.com/site/about/guidelines.xhtml

Dep. Var.:	Would you get the vaccine?		
	No	Not sure	Yes
WHO excess deaths due to COVID-19 terciles (ref.- bottom tercile)			
Excess deaths (middle tercile)	-0.075	-0.019	0.094
	(0.057)	(0.015)	(0.071)
Excess deaths (top tercile)	0.057	0.010	-0.067
	(0.096)	(0.015)	(0.112)
Confidence in government index tercile (ref.- top tercile)			
Confidence in government (middle tercile)	0.052	0.016	-0.069
	(0.041)	(0.013)	(0.054)
Confidence in government (bottom tercile)	0.040	0.013	-0.053
	(0.070)	(0.023)	(0.093)
R-squared		0.0866	
N		65088	

Notes: Weighted ordinal logit regressions. Categories ranked from no to not sure to yes. Marginal effects reported. Standard errors clustered at country level. Ref=reference group; EAP= East Asia and the Pacific; ECA= Europe and Central Asia; LAC=Latin America and the Caribbean; MNA= Middle East and North Africa; SSA= Sub-Saharan Africa; LIC=Low-income countries; LMIC=Lower middle-income countries, UMIC=Upper middle-income countries. *, **, *** indicates significance at the 95%, 99%, and 99.9% level.

For peer review only

Supplemental Table S6: Percent of population non-hesitant to take the COVID-19 vaccine, 14 countries with multiple rounds of survey results (95% confidence intervals in parentheses).

country	**Oct-2020**	**Nov-2020**	**Dec-2020**	**Jan-2021**	**Feb-2021**	**Mar-2021**	**Apr-2021**	**May-2021**	**Jun-2021**	**Jul-2021**	**Aug-2021**
Burkina Faso			0.79 (0.73, 0.86)						0.76 (0.67, 0.86)		
Congo, Rep.			0.84 (0.75, 0.94)			0.87 (0.81, 0.93)					
Ethiopia	0.98 (0.91, 1.00)				0.97 (0.89, 1.00)						
Gambia, The			0.65 (0.58, 0.71)				0.55 (0.49, 0.60)				0.67 (0.60, 0.74)
Georgia				0.36 (0.33, 0.38)		0.29 (0.27, 0.32)			0.35 (0.32, 0.38)		
Indonesia		0.79 (0.73, 0.85)				0.79 (0.72, 0.85)					
Iraq			0.66 (0.61, 0.72)	0.50 (0.45, 0.54)					0.45 (0.40, 0.50)	0.47 (0.42, 0.52)	0.53 (0.47, 0.58)
Kazakhstan					0.23 (0.17, 0.28)			0.32 (0.27, 0.37)	0.25 (0.21, 0.29)		
Malawi		0.83 (0.76, 0.89)				0.52 (0.47, 0.58)	0.71 (0.64, 0.78)				
Nigeria	0.86 (0.80, 0.93)				0.83 (0.77, 0.90)						
Philippines			0.44 (0.40, 0.48)					0.46 (0.42, 0.51)			
Tajikistan								0.73 (0.61, 0.84)	0.79 (0.67, 0.92)	0.80 (0.65, 0.95)	0.73 (0.57, 0.90)
Uganda		0.84 (0.79, 0.90)			0.88 (0.83, 0.94)						
Uzbekistan							0.55 (0.51, 0.60)	0.50 (0.46, 0.54)	0.45 (0.41, 0.49)		

For peer review only

For peer review only - http://bmjopen.bmj.com/site/about/guidelines.xhtml

Supplemental Table S7: Comparison of results on vaccine hesitancy from the HFPS and other studies

**Comparison with de Figueiredo and Larson (2021)**		
	**HFPS**	**de Figueiredo and Larson**
Argentina	10.1%	24.3%
Brazil	3.1%	17.0%
Chile	3.4%	27.9%
Croatia	33.2%	58.5%
Ecuador	19.4%	20.3%
Indonesia	21.3%	17.1%
Lebanon	32.2%	55.9%
Malaysia	25.8%	13.9%
Mexico	6.2%	18.0%
Nigeria	15.2%	35.9%
Paraguay	15.3%	48.5%
Peru	10.5%	28.3%
Vietnam	15.9%	3.2%
Average	*16.3%*	*28.4%*
**Comparison with Wouters et al. 2021**		
	**HFPS**	**Wouters et al.**
Argentina	10.1%	24.0%
Brazil	3.1%	12.0%
Chile	3.4%	28.0%
Croatia	33.2%	59.0%
Ecuador	19.4%	20.0%
Indonesia	42.6%	17.0%
Lebanon	32.2%	56.0%
Mexico	6.2%	18.0%
Nigeria	30.3%	36.0%
Paraguay	15.3%	49.0%
Peru	10.5%	28.0%
Vietnam	15.9%	2.0%
Average	*18.5%*	*29.1%*
**Comparison with Gallup (2021)**		
**Country**	**HFPS Hesitancy**	**Gallup**
Argentina	10.1%	37.0%
Bolivia	24.2%	35.0%
Brazil	3.1%	30.0%
Burkina Faso	22.2%	44.0%
Chile	3.4%	40.0%
Colombia	11.2%	30.0%
Congo, Dem. Rep.	61.2%	61.2%
Congo, Rep.	14.4%	48.0%

For peer review only - http://bmjopen.bmj.com/site/about/guidelines.xhtml

Costa Rica	11.9%	26.0%
Croatia	33.2%	57.0%
Dominican Republic	5.0%	35.0%
Ecuador	19.4%	28.0%
El Salvador	8.1%	25.0%
Ethiopia	2.8%	16.0%
Georgia	66.8%	44.0%
Guatemala	29.6%	29.0%
Guinea	20.1%	46.0%
Honduras	13.9%	30.0%
Indonesia	21.3%	30.0%
Iraq	47.8%	39.0%
Jamaica	50.6%	68.0%
Kenya	17.9%	27.0%
Lao PDR	12.9%	16.0%
Lebanon	32.2%	57.0%
Malaysia	25.8%	28.0%
Mali	21.1%	51.0%
Mexico	6.2%	25.0%
Mongolia	19.2%	39.0%
Nicaragua	18.8%	13.0%
Nigeria	15.2%	42.0%
Paraguay	15.3%	47.0%
Peru	10.5%	27.0%
Philippines	54.9%	49.0%
Tajikistan	23.7%	36.0%
Thailand	36.6%	39.0%
Uganda	13.6%	38.0%
Uruguay	9.1%	39.0%
Uzbekistan	49.8%	30.0%
Vietnam	15.9%	19.0%
Zimbabwe	15.8%	27.0%
Average	*22.4%*	*36.2%*
**Comparison with Facebook (2021)**		
**Country**	**HFPS**	**FB**
Argentina	10.1%	21.5%
Belize	28.4%	48.0%
Brazil	3.1%	25.8%
Chile	3.4%	43.0%
Colombia	11.2%	15.7%
Costa Rica	11.9%	17.1%
Ecuador	19.4%	17.1%

For peer review only - http://bmjopen.bmj.com/site/about/guidelines.xhtml

El Salvador	8.1%	15.8%
Guatemala	29.6%	18.6%
Guyana	20.0%	54.4%
Honduras	13.9%	13.7%
Indonesia	21.3%	19.2%
Iraq	47.8%	52.8%
Lebanon	32.2%	22.3%
Mexico	6.2%	13.0%
Nicaragua	18.8%	37.4%
Panama	13.3%	24.9%
Paraguay	15.3%	16.2%
Peru	10.5%	12.2%
Uruguay	9.1%	50.8%
Average	*16.7%*	*27.0%*

For peer review only

For peer review only - http://bmjopen.bmj.com/site/about/guidelines.xhtml

Supplemental Figure S1: Vaccination rates by income group

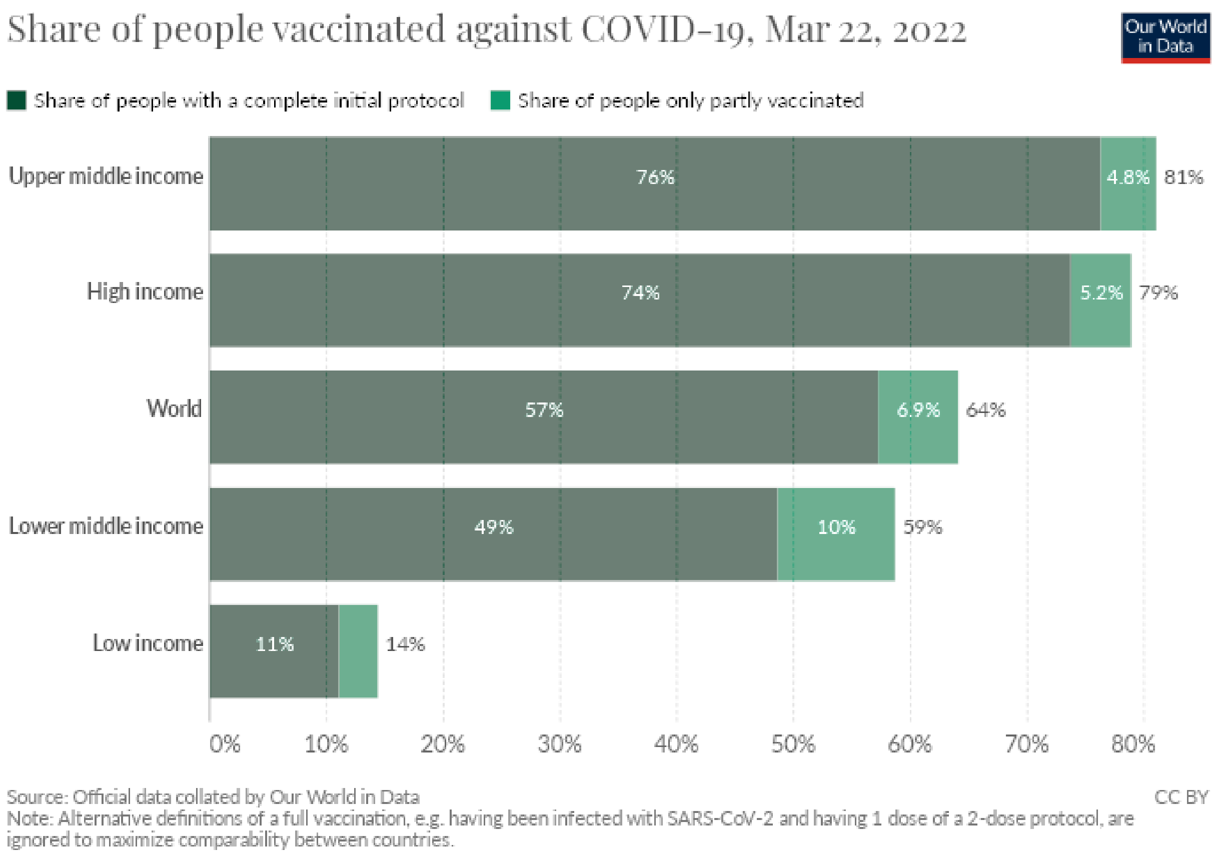

w only

Data source: Ritchie et al. 2022 as of March 22, 2022.

For peer review only - http://bmjopen.bmj.com/site/about/guidelines.xhtml

Supplemental Figure S2: Vaccine rollout rates by income group

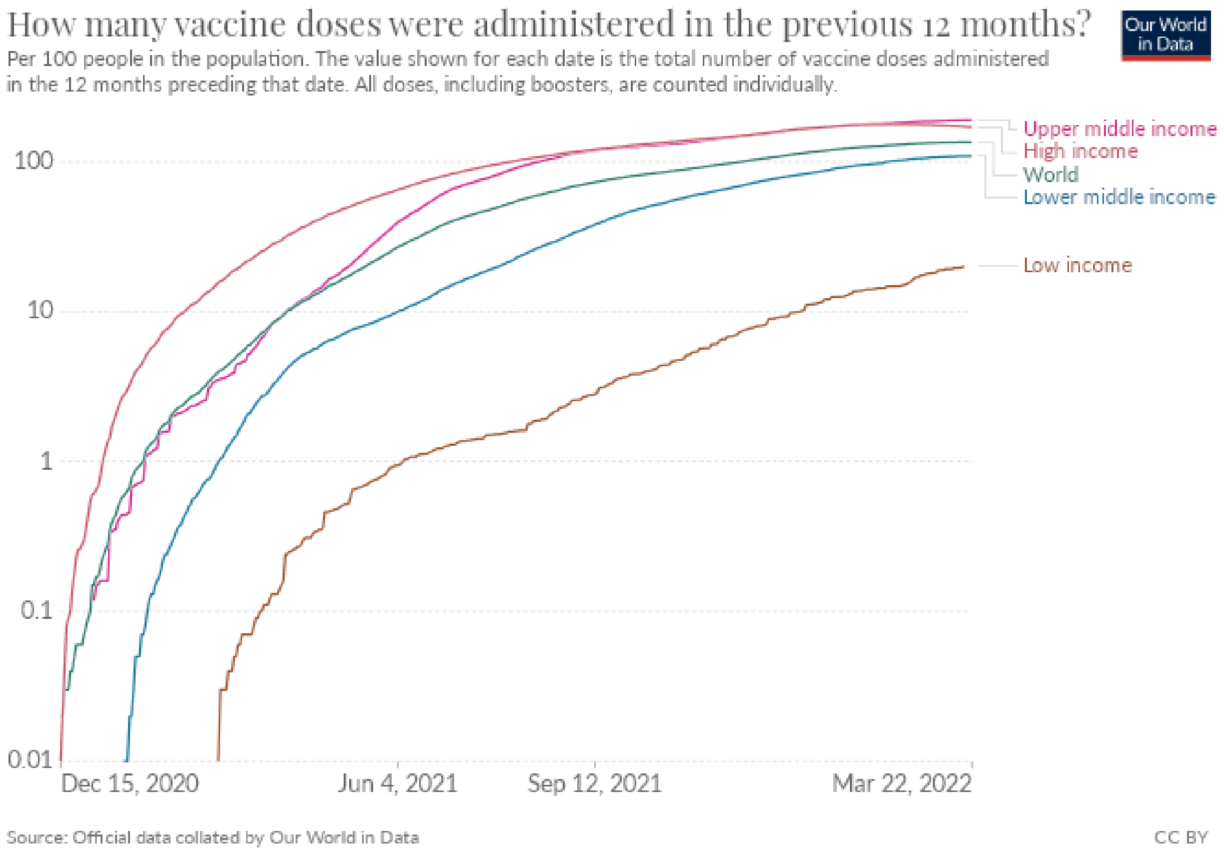

Data source: Ritchie et al. 2022 as of March 22, 2022.

For peer review only - http://bmjopen.bmj.com/site/about/guidelines.xhtml

Supplemental Figure S3: Comparison of share of population COVID-19 that is hesitant to take the COVID-19 vaccine, HFPS with other studies

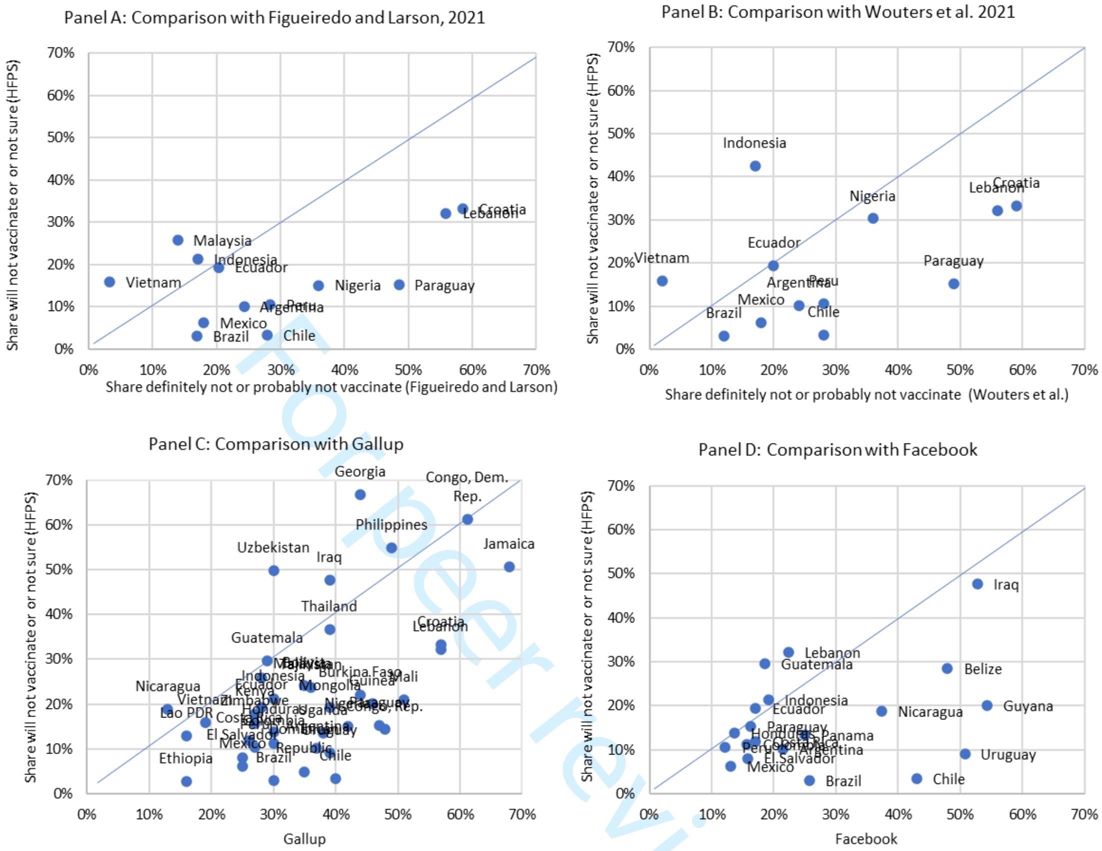

Notes: The y-axes indicate the share of the sample who reported not being willing or not sure if they will take the COVID-19 vaccine, as reported in the HFPS. The x-axes indicate the share of households in the survey that will definitely or probably not be vaccinated against COVID-19 from the following sources. Panel A: Figueiredo and Larson (2021). Panel B: Wouters et al. (2021). Panel C: Gallup (2021). Panel D: Facebook (2021).

For peer review only - http://bmjopen.bmj.com/site/about/guidelines.xhtml

Supplemental Figure S4: Comparison of COVID-19 Vaccine Hesitancy with pre-COVID sentiment about vaccines from 2019 Global Monitor, 45 countries

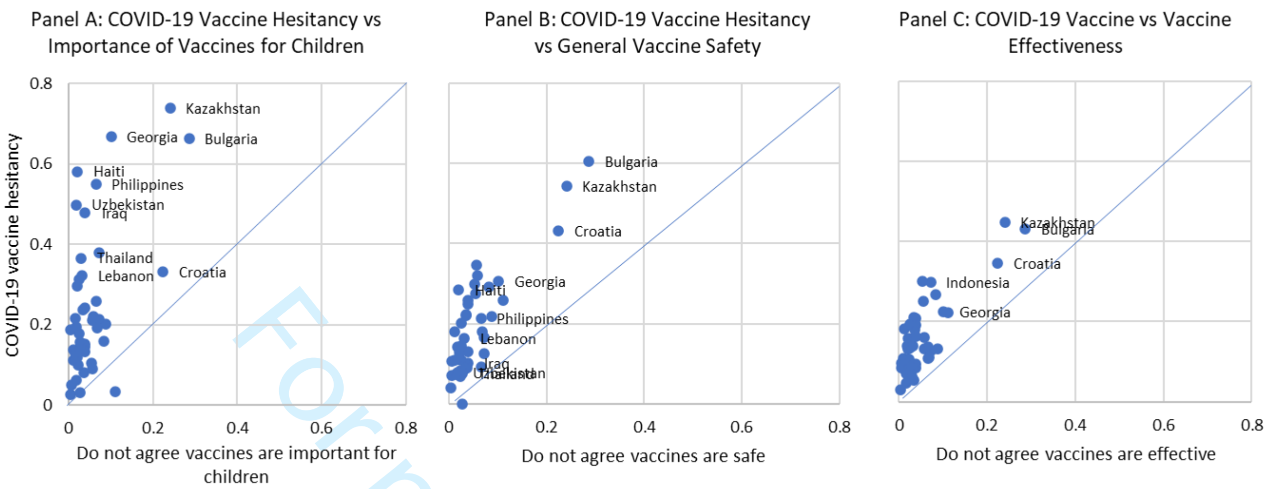

Notes: The y-axes indicate the share of the sample who report either not willing or not sure if they will vaccinate, as reported in the HFPS. The x-axes indicate the share of the 2019 Global Monitor Report sample that reported they do not agree vaccines are important for children (Panel A), do not agree that vaccines are safe (Panel B), and do not agree vaccines are effective.

For peer review only - http://bmjopen.bmj.com/site/about/guidelines.xhtml

Supplemental Figure S5: Reason for "definitely not" (upper panel) or "probably not" (lower panel) taking the COVID-19 vaccine, Facebook data for month of HFPS)

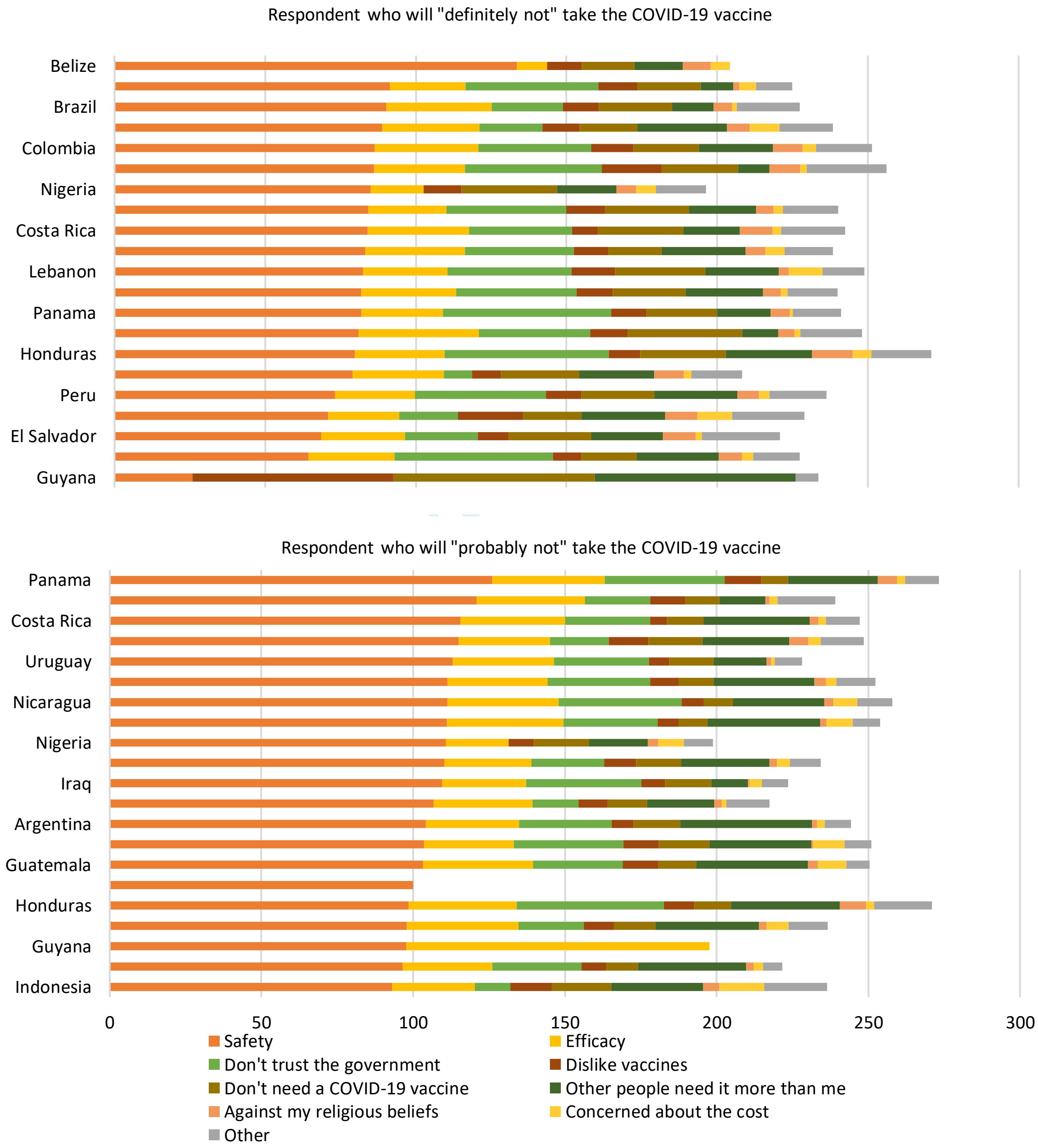

Notes: Y-axes indicate the share of survey respondents; multiple answers were allowed. Data source: Facebook (2021) for same month as reported in the HFPS. The month for each country is reported in Supplemental Table S1.

For peer review only - http://bmjopen.bmj.com/site/about/guidelines.xhtml

References- Supplemental Materials

de Figueiredo A, Larson HJ. Exploratory study of the global intent to accept COVID-19 vaccinations. Communications medicine. 2021 Sep 9;1(1):1-0.

Facebook. 2021. COVID-19 Trends and Impact Survey.

Gallup. 2019. Wellcome Global Monitor – First Wave Findings. https://wellcome.org/reports/wellcome-global-monitor/2018#downloads-4d1c.

Gallup. 2021. Gallup World Poll. https://www.gallup.com/178667/gallup-world-poll-work.aspx.

Hale T, Angrist N, Goldszmidt R, et al. 2022. A global panel database of pandemic policies (Oxford COVID-19 government response Tracker). Nat Hum Behav 2021;5:529–38. Database accessed Feb 28, 2022.

Inglehart, R, C. Haerpfer, A. Moreno et al. 2022. Would Values Survey: Round Seven – Country Pooled Datafile Version: https://www.worldvaluessurvey.org/WVSOnline.jsp. (accessed April 21, 2022) Madrid: JD Systems Institute.

Lazarus, JV, Ratzan SC , and Palayew A et al. 2021. A global survey of potential acceptance of a COVID-19 vaccine. Brief Communication. Nature Medicine 27: 225–228. https://doi.org/10.1038/s41591-020-1124-9.

Sallam M. COVID-19 Vaccine Hesitancy Worldwide: A Concise Systematic Review of Vaccine Acceptance Rates. Vaccines (Basel). 2021 Feb 16;9(2):160. doi: 10.3390/vaccines9020160. PMID: 33669441; PMCID: PMC7920465.

Solís Arce, J.S., Warren, S.S., Meriggi, N.F. *et al.* COVID-19 vaccine acceptance and hesitancy in low- and middle-income countries. *Nat Med* **27,** 1385–1394 (2021). https://doi.org/10.1038/s41591-021-01454-y.

World Bank. 2020. High Frequency Mobile Phone Surveys of Households to Assess the Impacts of COVID-19: Overview (English). Washington, D.C.: World Bank Group. http://documents.worldbank.org/curated/en/703571588695361920/Overview

World Bank. 2021. World Development Indicators. Washington, D.C.: The World Bank (producer and distributor). http://data.worldbank.org/data-catalog/world-development-indicators. Accessed March 12, 2022.

World Health Organization. 2022. Global Excess Deaths Associated with COVID-19 (Modelled Estimates). Downloaded on September 8, 2022 at https://www.who.int/data/sets/global-excess-deaths-associated-with-covid-19-modelled-estimates.

Wouters, O.J., Shadlen, K.C., Salcher-Konrad, M., Pollard, A.J., Larson, H.J., Teerawattananon, Y. and Jit, M., 2021. Challenges in ensuring global access to COVID-19 vaccines: production, affordability, allocation, and deployment. The Lancet, 397(10278), pp.1023-1034.

For peer review only

For peer review only - http://bmjopen.bmj.com/site/about/guidelines.xhtml
